# Helixer: ab initio prediction of primary eukaryotic gene models combining deep learning and a hidden Markov model

**DOI:** 10.1038/s41592-025-02939-1

**Published:** 2025-11-24

**Authors:** Felix Holst, Anthony M. Bolger, Felicitas Kindel, Christopher Günther, Janina Maß, Sebastian Triesch, Niklas Kiel, Nima Saadat, Oliver Ebenhöh, Björn Usadel, Rainer Schwacke, Andreas P. M. Weber, Marie E. Bolger, Alisandra K. Denton

**Affiliations:** 1 Institute of Plant Biochemistry, Heinrich Heine University, Düsseldorf, Germany; 2 IBG-4 Bioinformatics, Forschungszentrum Jülich, Jülich, Germany; 3 Institute of Quantitative and Theoretical Biology, Heinrich Heine University, Düsseldorf, Germany; 4 Cluster of Excellence on Plant Sciences, Heinrich Heine University, Düsseldorf, Germany; 5 Institute for Biological Data Science, Heinrich Heine University, Düsseldorf, Germany; 6 Recursion, Valence Labs, Montreal, Quebec Canada

**Keywords:** Computational biology and bioinformatics, Machine learning, Genome informatics

## Abstract

The accurate identification of genes is vital for understanding biological function, yet this remains challenging across many newly sequenced or less-studied species. Here we present Helixer, an artificial intelligence-based tool for ab initio gene prediction that delivers highly accurate gene models across fungal, plant, vertebrate and invertebrate genomes. Unlike traditional methods, Helixer operates without requiring additional experimental data such as RNA sequencing, making it broadly applicable to diverse species. We show that Helixer’s pretrained models achieve accuracy on par with or exceeding current tools, producing gene annotations that closely match expert-curated references across multiple evaluation metrics. Its design enables immediate use on genomes without retraining, providing an efficient, accessible solution for genome annotation in both research and applied settings. The tool is available as an open-source software for local installation via GitHub. An online web interface is also available as well as through the Galaxy ToolShed.

## Abstract (web summary)

By leveraging both deep learning and hidden Markov models, Helixer achieves broad taxonomic coverage for ab initio gene annotation of eukaryotic genomes from fungi, plants, vertebrates and invertebrates.

## Main

Major advances in genome sequencing and assembly^1^ have led to a rapid increase in genomics data. Accurate and fast in silico models are needed to analyze these data, extract biological knowledge and thereby accelerate research progress in biology and bioengineering. Gene calling, or structural gene annotation, plays a critical role but has not seen comparable advances in recent years.

Historically, eukaryotic gene calling relied primarily on (generalized) hidden Markov models (HMMs) such as GeneMark-ES^2,3^, FGENESH^4^ or AUGUSTUS^5,6^. However, these models lack the capacity to fully model biological complexity on their own. As a result, they are currently used as parts of data integration pipelines such as MAKER2^7^, PASA^8^, TOGA^9^ and BRAKER3^10^. These pipelines perform best with wet laboratory data such as RNA sequencing, other extrinsic evidence such as homologous proteins or databases with repetitive elements, and require substantial computational resources, often becoming the bottleneck in genome projects. The inconsistent availability of such resources leads to a heterogeneous quality of the resulting gene models. Errors are still being identified in gene models of extensively studied species such as human and mouse^11^, while in less-studied species the lower overall annotation quality is disruptive in large-scale analyses and can necessitate project-specific reannotation^12^. Illustrating this further, many newly sequenced species lack any annotation, with only circa 24% of the latest eukaryotic assemblies on the National Center for Biotechnology Information (NCBI) database having an accompanying annotation, lagging far behind the 89% annotated in prokaryotes. There is a pressing need for improved tools than can easily produce consistent high-quality annotations and strengthen the foundation for downstream applications such as target-gene characterization, transcriptomics, proteomics, genome-wide association studies and more.

Deep learning is a transformative technology where massive networks with the capacity to make extraordinarily complex and nonlinear fits are trained on large amounts of data. Deep learning has demonstrated remarkable performance across diverse fields, including language models (for example, GPT-3^13^), playing strategy games (for example, AlphaGo^14^), deciphering mathematical equations^15,16^ and protein folding^17^. In recent years, deep learning networks have been employed with promising outcomes for some elements of gene calling such as predicting genes in prokaryotes^18^, differentiating coding from noncoding RNAs^19^, differentiating exons and introns in human sequences^20^, assessing splice site type and strength^21,22^ and predicting gene model elements such as start codons, splice sites and poly-adenylation sites^23,24^. Building on these developments, a recent addition to the field is Tiberius, a deep neural network specifically designed for annotating mammalian genomes^25^. In our proof-of-principle version of Helixer, we used deep learning to classify the genic class of each base pair^26^, achieving substantial performance gains compared to an existing ab initio predictor and even exceeding the quality of some references in consistency with independent RNA-sequencing data.

Deep learning has already shown promising results in tasks related to eukaryotic gene annotation. This work advances the field by achieving finalized eukaryotic gene models. Here, we present Helixer as an application-ready tool that includes various improvements to our previous work, along with a hidden Markov model-based postprocessing tool named HelixerPost. This is integrated, and in a single command, the Helixer.py script takes the genome sequence as input (in FASTA format) and outputs structural annotations for primary gene models (in GFF3 format). Pretrained models are now provided for fungal, invertebrate and mammalian genomes, in addition to the previously available plant and vertebrate models. Helixer does not require extrinsic data nor species-specific retraining.

## Results

### Overview of Helixer

Helixer is a deep learning-based framework for eukaryotic gene annotation directly from genomic DNA. It uses a sequence to label neural network that predicts base-wise genomic features including coding regions, untranslated regions (UTRs) and intron–exon boundaries based solely on nucleotide sequence. The architecture integrates convolutional and recurrent layers to capture both local sequence motifs and long-range dependencies, followed by a biologically informed decoding step that assembles coherent gene models. Trained end-to-end on high-quality reference annotations, Helixer generalizes across species without requiring transcriptomic or homology-based evidence. This design enables consistent annotations while minimizing manual curation, providing a scalable solution for annotating newly sequenced genomes and supporting large-scale comparative genomics.

### The released Helixer models show state-of-the-art performance compared to existing ab initio gene calling tools and previous Helixer models

As our previous work^26^ set the state of the art for base-wise predictions, we first compared the latest models to models trained with the hand-selected six (vertebrate) or nine (plant) species used previously, as well as to all intermittently released models (Supplementary Figs. 1–8 and Supplementary Tables 1–4). The best models released here (vertebrate_v0.3_m_0080, invertebrate_v0.3_m_0100, land_plant_v0.3_a_0080 and fungi_v0.3_a_0100), had the highest median Genic F1 for their phylogenetic target range, and showed a more balanced performance across said range, compared to other Helixer models. Nevertheless, no single model was consistently better for all species, so all plotted models are released to allow researchers to select the model likely to perform best for their species of interest.

While promising, the high base-wise performance of raw HelixerBW predictions does not necessarily imply that the performance is maintained through postprocessing. Therefore, we additionally computed the F1 for the phases (phase F1) (Supplementary Figs. 9–13 and Supplementary Table 5), the F1 for the subgenic classes (subgenic F1) (Supplementary Table 6) and the F1 for the genic classes (genic F1) (Supplementary Table 7) scores for the final gene models output by HelixerPost on the selected test species. Importantly, we see that predictions postprocessed via HelixerPost have very similar performance to the HelixerBW predictions, with a slight increase in 43 of 45 test species.

Additionally, we inferred gene models using two existing HMM tools, GeneMark-ES and—where trained models were available for the test species or a closely related species—AUGUSTUS. GeneMark-ES was used without repeat masking, while AUGUSTUS was used both with and without repeat masking.

The phase F1 of HelixerPost (Table 1 and Supplementary Table 5) was notably higher than GeneMark-ES and Augustus across both plants and vertebrates and still somewhat higher for invertebrates generally, although not for all species. All three tools showed similar performance for fungi, with HelixerPost having a slight margin of 0.007 overall. Similar patterns were found with subgenic and genic F1 metrics (Supplementary Figs. 14 and 15 and Supplementary Tables 6 and 7).

**Table 1:** Base-wise phase F1 statistics for test species, summarized as the mean value per group

Group	HelixerBW	HelixerPost	GeneMark-ES	AUGUSTUS^a^
Fungi	0.9514	**0.9540**	0.9466	0.9221
Plants	0.8001	**0.8099**	0.4089	0.6300
Vertebrates	0.8641	**0.8829**	0.2326	0.6372
Invertebrates	0.8458	**0.8562**	0.7172	0.7914

The highest performing tool for each group is highlighted in bold.

^a^For AUGUSTUS, pretrained models were only available for 4 of the 13 plant, 1 of the 11 vertebrate and 5 of the 15 invertebrate test species. All reported summary statistics reflect only those species.

**Fig. 1:**
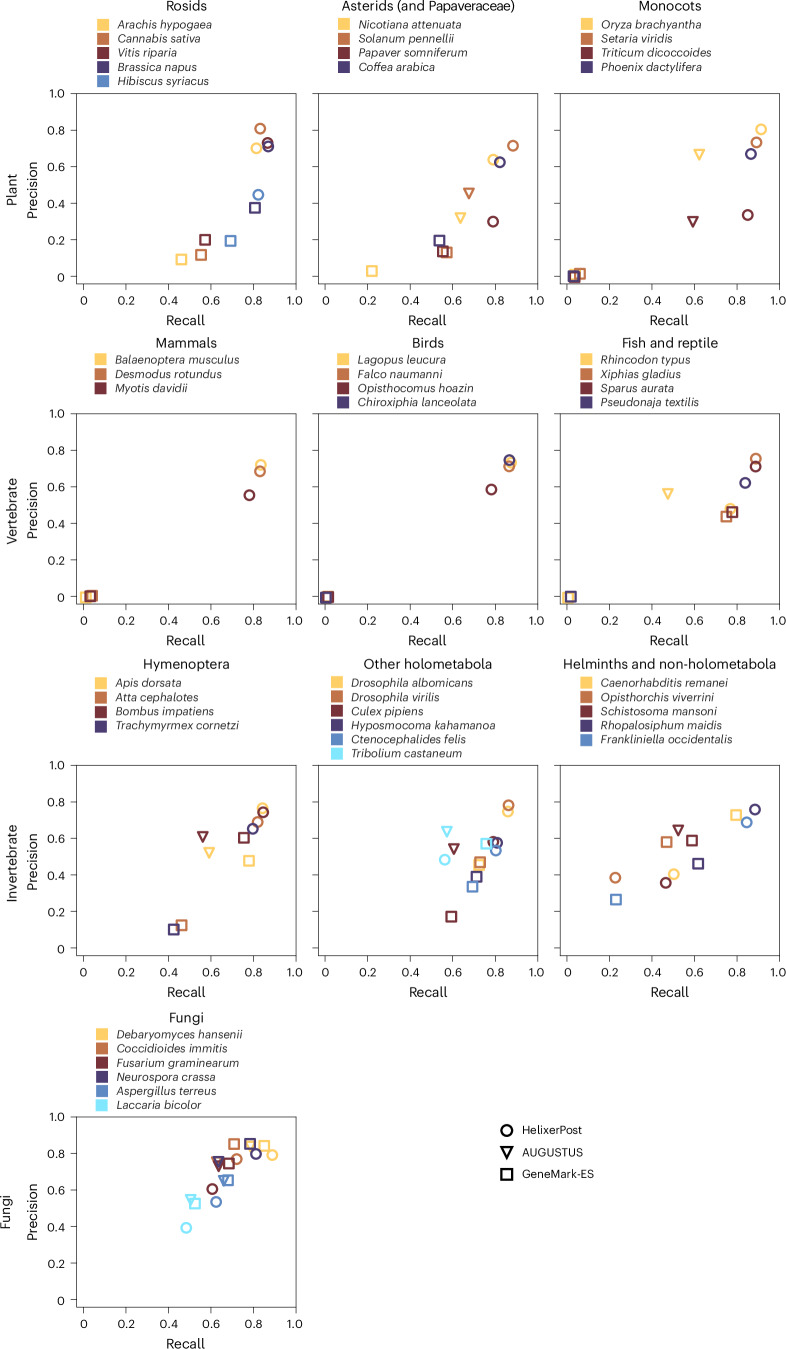
Exon precision and recall comparison. A comparison of precision and recall between HelixerPost (circles), AUGUSTUS (triangles) and GeneMark-ES (squares). The first row shows plants, the second vertebrates, the third invertebrates and the fourth fungi.

**Extended Data Fig. 1:**
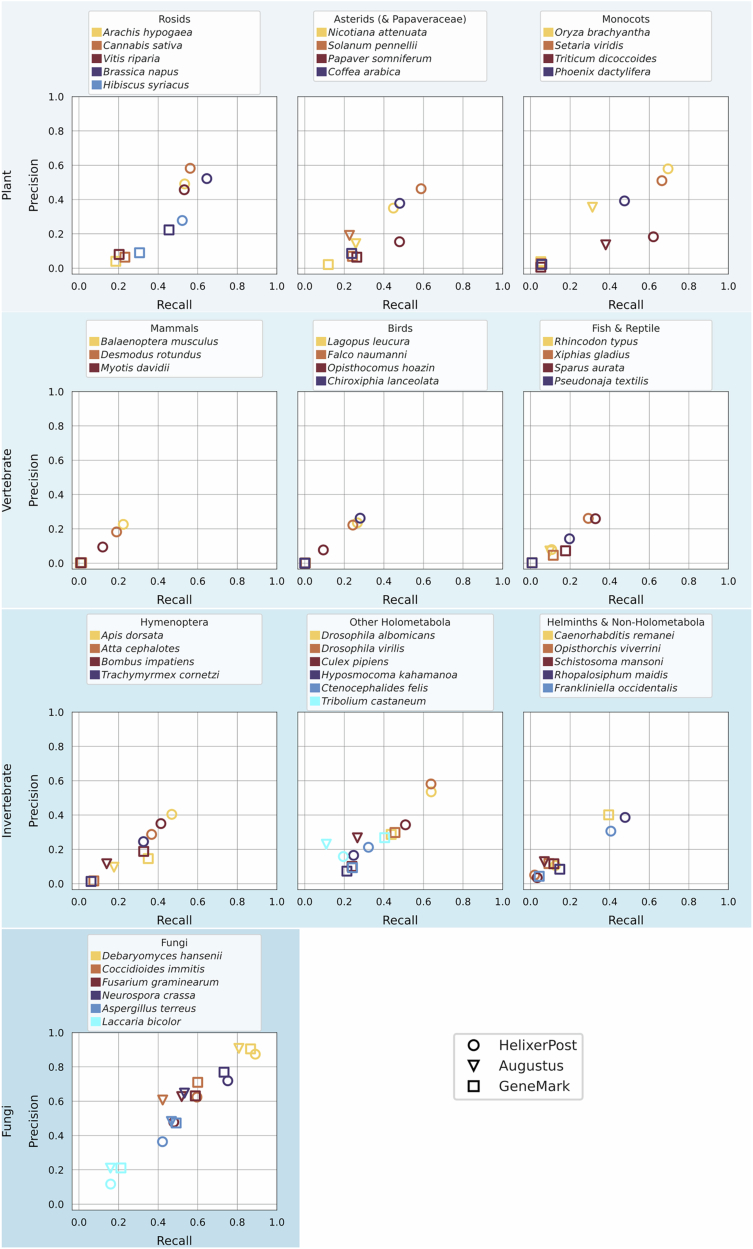
Gene precision and recall. Gene precision and recall comparison between HelixerPost (circle), Augustus (triangle) and GeneMark (square). The first row shows plants, the second vertebrates, the third invertebrates and the fourth fungi.

Moving from base-wise to feature- or protein-level evaluation, we found lower absolute precision, recall and F1 scores (Fig. 1, Table 2, Extended Data Fig. 1, Supplementary Fig. 16 and Supplementary Tables 8–11). Helixer scored highest for plants and vertebrates, but both HMM tools gained an edge in fungi. Helixer maintained a small advantage in invertebrates overall, although AUGUSTUS and GeneMark-ES were again strongest in some species. Overall, the gene precision and recall scores were lower for every tool than the exon scores, which is expected for the harder task. For most species, Helixer tends to have a higher recall score than precision.

**Table 2:** Feature F1 statistics for test species at the exon, intron, intron chain and transcript levels, summarized as the mean value per group and level

Group	Exon			Intron			Intron chain			transcript		
	Helixer	GeneMark-ES	AUGUSTUS^a^	Helixer	GeneMark-ES	AUGUSTUS^a^	Helixer	GeneMark-ES	AUGUSTUS^a^	Helixer	GeneMark-ES	AUGUSTUS^a^
Fungi	0.6678	**0.7240**	0.6743	0.6061	**0.7053**	0.6812	0.4431	**0.5210**	0.4615	0.5386	**0.5981**	0.5269
Plant	**0.7143**	0.1810	0.5044	**0.7232**	0.1861	0.5251	**0.4338**	0.0554	0.1792	**0.4618**	0.0934	0.2310
Vertebrate	**0.7405**	0.1078	0.5145	**0.6912**	0.1059	0.4966	**0.1740**	0.0130	0.0656	**0.1977**	0.0170	0.0823
Invertebrate	**0.6608**	0.4872	0.5786	**0.6416**	0.5142	0.6187	**0.2939**	0.1581	0.1475	**0.3066**	0.1783	0.1513

The highest F1 score per row is highlighted in bold. ^a^For AUGUSTUS, pretrained models were only available for 4 of the 13 plant, 1 of the 11 vertebrate and 5 of the 15 invertebrate test species. All reported summary statistics reflect only those species.

When comparing Helixer and AUGUSTUS with softmasking enabled, Helixer outperforms AUGUSTUS in exon F1 score for 9 species, gene F1 score for 10 species and intron F1 score for 8 species out of a total of 16 tested (Supplementary Fig. 17 and Supplementary Tables 8–11).

The completeness of predicted proteomes^27^ was quantified for the reference annotation and the three prediction tools (Extended Data Table 1, Supplementary Figs. 18–22 and Supplementary Table 12).

The reference annotations generally had the highest performance (39 of 45 species), which is unsurprising since they typically benefit from additional data sources and manual curation. The results of the prediction tools were broadly similar to the base-wise and feature assessments above, with HelixerPost leading strongly within plants and vertebrates, where it even approached the result of the reference. Invertebrates again varied by species, with Helixer leading by a smaller margin overall but GeneMark-ES performing best on several species. Fungi was the most competitive clade, with Helixer leading by a small margin, and interestingly all tools outperformed the reference.

Interestingly, the three invertebrates where HelixerPost scored behind GeneMark-ES in phase F1 and Benchmarking Universal Single-Copy Orthologs (BUSCO) count had the overall lowest BUSCO count in the reference annotations. This hints at larger challenges related to annotating these genomes, be it exceptional divergence or simply a paucity of well-annotated genomes in close phylogenetic proximity to use either for training or for the homology mapping step of an annotation pipeline. However, it may also simply reflect that the invertebrate prediction models are less optimized.

### Comparison with Tiberius

**Fig. 2:**
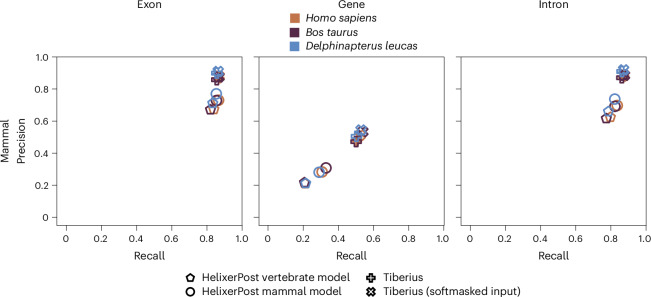
Precision and recall comparison. A comparison of precision and recall between the HelixerPost vertebrate model (pentagons), HelixerPost mammal model (circles), Tiberius (pluses) and Tiberius with softmasked input (crosses). The first column depicts exon, the second gene and the third intron metrics.

To compare Helixer to Tiberius, we trained a model focused on the mammalian clade. We used the three test species *Homo sapiens*, *Bos taurus* and *Delphinapterus leucas*. Even though we could improve Helixers prediction quality compared to our vertebrate model, Tiberius still outperforms Helixer (Fig. 2 and Supplementary Table 13). Especially gene recall and precision is consistently 20% higher. Regarding exon recall, Helixer and Tiberius are almost on par, with Tiberius still taking the lead. For exon precision Helixer shows 10–15% lower values. While we acknowledge that Tiberius outperforms Helixer in the Mammalia clade, we offer a more phylogenetically diverse range of models, especially for the often-underrepresented plant species.

### Ablation analyses

To validate the importance of major changes made during development, we performed an ablation analysis by individually excluding each of the major changes, training a new model, then predicting and comparing prediction quality to the final model (Supplementary Fig. 23).

While none of the changes had a major effect on the overall genic F1 score (Supplementary Fig. 23a), this was not the target of most of the changes. Without transition weights (Supplementary Fig. 23e), the network displays high uncertainty immediately around start and stop codons, as well as acceptor and donor splice sites. All of the low, medium and final transition weights push the networks toward sharper transitions in an example gene, both right at the start codon (Supplementary Fig. 23e) and more widely helping to clear up uncertainty throughout the UTR (Supplementary Fig. 23d), with the final transition weights being most effective. To quantify this on a larger scale, we calculated the genic F1 specifically for the base pairs immediately before or after transitions. Higher transition weights resulted in the expected increase in transition genic F1, with the largest difference between low transition weights and none (Supplementary Fig. 23c). Moreover, the models with reduced transition weights show markedly more internal phase mistakes, that is, where both reference and predictions have a 0–2 codon phase, but the phases do not match (Supplementary Fig. 23b).

Interestingly, including the phase also improved the performance around transitions in the example (Supplementary Fig. 23d,e). In addition, phase predictions are useful signals for postprocessing into final gene models. The long short-term memory (LSTM) model and the hybrid model have similar performances. We acknowledge that this analysis could be made more conclusive by addressing the effect of random starting parameters by adding replicates or, where applicable (the number and arrangement of trainable parameters is only constant for the transition weight ablations), recording and reusing a random seed.

### Helixer’s annotations approach reference quality

The F1 metrics shown so far treat the references as the ‘ground truth’; however, the provided references themselves are ultimately the output of a gene annotation pipeline, that is. data-supported predictions. Measuring performance against such references is particularly limited for understanding how good the absolute performance is as it approaches that of the provided reference. In the BUSCO analysis above we already observed that Helixer’s performance was close to that of the reference. Therefore, we used two additional homology-based methods to evaluate in more detail the quality of Helixer and GeneMark-ES predictions versus the reference for the plant test species. AUGUSTUS predictions were omitted since they covered only four plant species.

**Extended Data Fig. 2:**
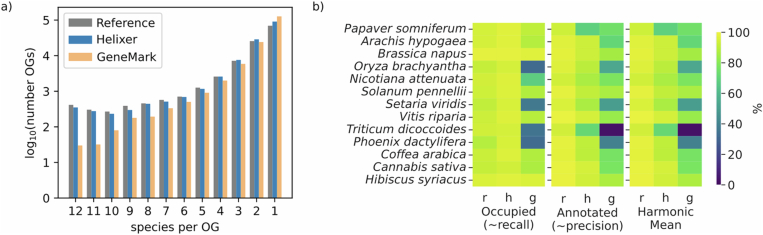
Homology based evaluation of annotations. **a**) Orthogroup occupancy (number of species represented out of 12) for orthogroups based on the reference, Helixer’s, and GeneMark’s annotations (each clustered individually). *Triticum dicoccoides* was excluded from this analysis. **b**) Comparison of proteomes to curated Mapman4 protein annotations. r=reference, h=Helixer (post processed), g=GeneMark. The harmonic mean is that of the %Annotated and the %Occupied, and here is conceptually a proxy for the F1.

First, we used the ability of a set of proteomes to form orthogroups as a reference-free indicator for quality, which is particularly relevant when considering annotation applicability for comparative genomic tasks. The more accurate annotation is expected to have more orthogroups with all 12 species represented and generally more orthogroups with higher numbers of species represented. The results are shown in Extended Data Fig. 2 and Supplementary Figs. 24–28.

Here, the reference performs best, with 0.38% of orthogroups containing all 12 species, and 36.5% containing two or more species, followed by Helixer at 0.26% and 31.1% for the same statistics, respectively, and GeneMark-ES at 0.019% and 21.2%, respectively. The reference has the most orthogroups with four or more species, followed by Helixer with two or three species and GeneMark-ES with only one species (Extended Data Fig. 2a). Notably, Helixer’s performance by these metrics was more like that of the reference, that is, to extrinsic data-supported predictions, than to GeneMark-ES’s predictions, which, comparable to Helixer’s are made from the DNA sequence alone. Moreover, all of the reference annotations were respectable (minimum complete BUSCOs of 96.5%, and eight of the species above 99%).

Second, given that the orthogroup-based numbers could potentially be skewed by consistent cross-species mistakes, such as misannotation of transposons, we used the Mapman4 protein annotations (curated plant protein-coding gene families) to evaluate annotation quality. This further gives us a proxy for precision (the percentage of proteins that could be annotated) and recall (the percentage of gene families occupied by a protein). Measured this way, the reference had an average precision, recall and harmonic mean (of precision and recall) of 0.966, 0.931 and 0.948, followed by Helixer (0.878, 0.958 and 0.914, respectively) and GeneMark-ES (0.719, 0.742 and 0.724, respectively) (Extended Data Fig. 2b and Supplementary Table 14).

Thus, the ab initio annotations from Helixer show higher recall than the references for the plant species test set but are behind the references on precision, particularly for the specific species *Papaver somniferum* and *Triticum dicoccoides* (Extended Data Fig. 2b, Supplementary Figs. 24, 26 and 28, and Supplementary Table 14), which we note have the most fragmented assemblies, leading to an idea on how to further improve Helixer’s predictions (Supplementary Information).

### Filling gaps in *Arabidopsisthaliana*

**Extended Data Fig. 3:**
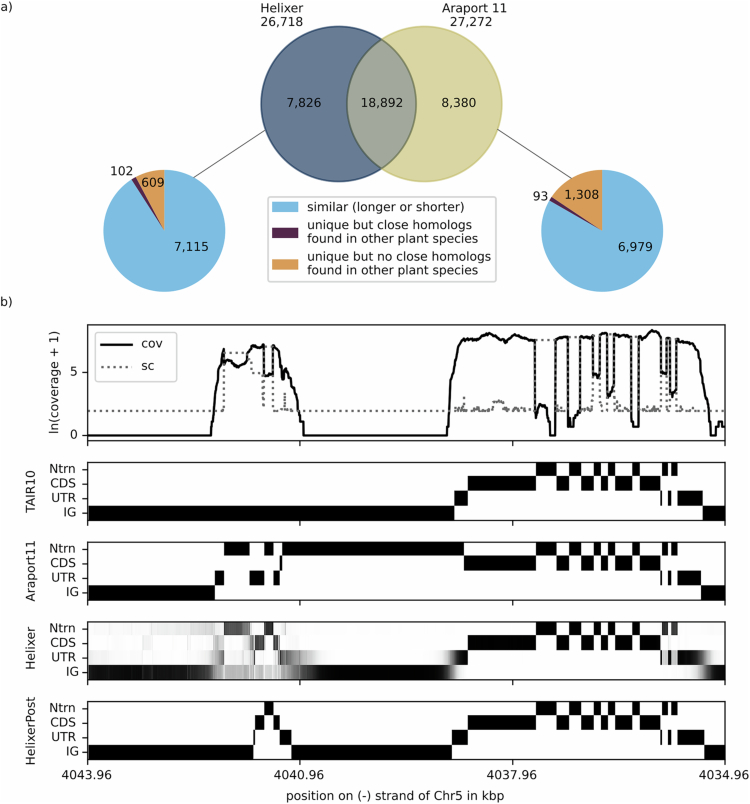
Comparison of the *Arabidopsis thaliana* proteome predicted by Helixer to the existing Araport11 annotation. **a**) Venn diagram and pie charts of the overlaps and differences between predicted proteomes. The pie charts categorize the unique sections from the Venn diagram further into three categories: ‘similar (longer or shorter)’, ‘unique with homologs in other plant species’ and ‘unique without homologs in other plant species. The second category encompasses likely legitimate annotations not found in the other proteome. The third category encompasses likely artefacts not found in the other proteome. **b**) visualization of RNAseq expression (cov=coverage; sc=spliced coverage) and available annotations at the genomic loci of the Phosphatidylinositol N-acetylglucosaminyltransferase *γ*-subunit (Chr5, - strand, from 4043960 to 4034960 bp, via HelixerPost).

We compared the annotations produced by Helixer for the model plant *A. thaliana*, to the established high-quality reference annotations TAIR10 and Araport11 (newest versions available in November 2022). When comparing Helixer to Araport11 (Extended Data Fig. 3a), the majority (~70%) of gene loci were found to have an exact match in Helixer. From the remaining ~30%, around 27% are highly similar but varied in their length. The unmatched sequences that remained from Helixer and Araport11 were considered as true annotations if the sequences were found to be conserved in at least 20 streptophyte species. For the Araport11 annotations, 93 of 1,401 were found to be true annotations, while 102 of 711 Helixer annotations were identified as true. Similar results were found when comparing Helixer to TAIR10 annotations (Supplementary Figs. 29 and 30).

From these 102 Helixer annotations not found in the Araport11 reference annotation, a notable example is the Phosphatidylinositol *N*-acetylglucosaminyltransferase γ subunit. This complex is known to be active in *A. thaliana*^28,29^ and the γ subunit locus identified by Helixer shows expression (Extended Data Fig. 3b and Supplementary Fig. 31). However, this γ subunit is entirely missing from TAIR10, and has a chimeric annotation with the next gene in Araport11, resulting in a mere 4 bp of out-of-frame overlap between the resulting protein annotations. While the final postprocessed annotation from Helixer has truncations in at least UTR and introns relative to both the RNA-sequencing data and the raw predictions,the resulting protein was long enough for homology-based identification. This highlights the power of Helixer to complement and improve even the most polished reference annotations.

### Benchmarking

**Extended Data Fig. 4:**
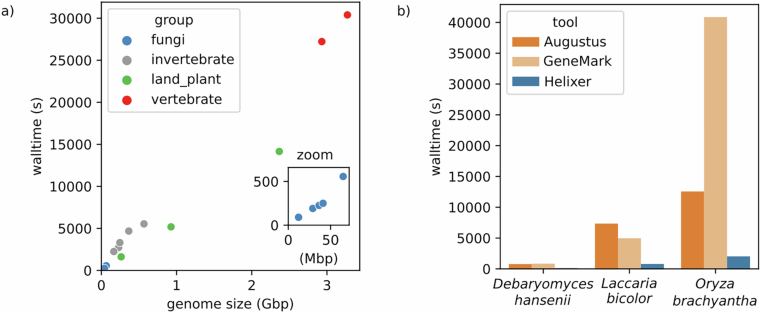
Benchmarking. **a**) Helixer walltime on test genomes and *Homo sapiens* on ‘workstation A’; **b**) comparison in single-threaded walltime between ab initio tools.

As shown in Extended Data Fig. 4, Helixer is faster than the state-of-the-art tools AUGUSTUS and GeneMark-ES, and scales approximately linearly with genome size within phylogenetic groups. When comparing to GeneMark-ES and Augustus on the smallest and largest fungus test genomes and smallest test plant genome, Helixer required 6.2–20.1 fold lower wall time in single-threaded mode. Both AUGUSTUS and Helixer can be parallelized by splitting the genome into multiple fasta files. However, we acknowledge that the other tools make use of more complete multithreading than Helixer does. In single-threaded mode, Helixer.py can annotate the 263-Mbp *Oryza brachyantha* genome in 27 min and the 3.3-Gbp human genome in just under 8.5 h (Supplementary Methods). The exact speed will vary by system, particularly in regards to the speed of the disk storage and the GPU (Supplementary Fig. 32). A speed comparison to Tiberius was performed by the authors of Tiberius^25^. In their setup, Tiberius annotated the human genome in 1 h and 39 min and Helixer in 8 h and 54 min.

## Discussion

Helixer represents a substantial advancement as a deep learning-based tool for producing full eukaryotic gene models and demonstrates what can be accomplished with a state-of-the-art ab initio gene caller. The primary gene models predicted by Helixer approach the quality of references produced via data-supported pipelines. This is achieved using a single tool, requiring no additional data beyond the DNA sequence, and only modest compute time.

Helixer performs a predictive task that no pre-existing tool is currently optimized for, namely going from an unmasked genome sequence to primary gene models for a phylogenetically diverse range of species, and all comparisons to existing tools were performed aligned to Helixer’s task. We acknowledge that if comparative tools had been consistently selected and configured for optimal performance in a different scenario, such as gene prediction without project-specific RNA-sequencing data but incorporating repeat masking and protein homology, they would probably have achieved higher performances. However, such approaches also entail substantially greater computational costs and require more user expertise. While this does not constitute a conceptual difference in the type of input information, since Helixer’s weights implicitly capture the extrinsic data, domain knowledge and compute resources used to construct the training references, it represents a major practical distinction owing to the greatly reduced requirements during inference.

In a comparison between Helixer and BRAKER2 on 54 crop plant species, Helixer matched BRAKER2 in overall coding sequence accuracy and outperforms it in detecting exon-containing regions and complete single-copy BUSCO genes, showing strength in identifying coding regions even under high GC content^30^. However, it was less precise at defining gene boundaries, particularly in fragmented genomes, and while BRAKER2 has higher gene-level recall, Helixer proved more consistent across plant clades, especially outperforming BRAKER2 on monocots.

While Helixer’s overall prediction quality may not yet match predictions generated with extrinsic data-supported pipelines, the standardized output format enables easy incorporation in such pipelines. This integration has the potential to immediately improve the final quality of predictions. Notably, our research has demonstrated that Helixer’s predictions can identify areas for improvement even in well-studied model species. Moreover, these annotations have been successfully applied for comparative genomics^31^. Helixer was also used for the ab initio genome annotation, either alone or in combination with other tools, of plants such as *Ribes nigrum* L.^32^, *Camellia sinensis*^33^, *Oxalis articulata*^34^ and the arctic moss *Ptychostomum knowltonii*^35^, as well as invertebrates such as *Coenonympha arcania*^36^, two *Frankliniella* species^37,38^ and the three economically harmful invasive species *Cydalima perspectalis*, *Leptoglossus occidentalis* and *Tecia solanivora*^39^. The combined attributes of sufficiently high quality and ease of generation make the structural annotations produced by Helixer valuable for many projects, especially for nonexperts.

The deep neural network Tiberius^25^ is very similar to Helixer in approach and concept. Although it shows higher performance in mammals overall, including more balanced exon precision and recall values, it lacks the phylogenetic diversity of the Helixer models. While Tiberius’ current focus is on mammals only (also predicting the longest gene isoform only), Helixer also provides fungi, plant, vertebrate and invertebrate models, which have already been adopted by the scientific community. Although Helixer represents a notable milestone as a fully applicable deep learning-based gene caller, it is not the pinnacle of what can be achieved by applying deep learning to structural gene annotation. We identify two primary areas for improvement: modeling and data handling.

In terms of modeling, there are several promising options that warrant exploration in future research. One such approach involves leveraging end-to-end prediction to harness the full potential of deep learning. Previous studies have successfully predicted transitions such as splice sites^22,40,41^. It is conceivable to encode a full gene structure as a series of transition tokens and positions, and predict structure with a many-to-many model architecture, similar to those used for large-scale language modeling^13,42^. This approach offers the advantage of being readily extensible for alternative splicing, but brings with it the challenge of an extremely sparse encoding. Sparse encoding coupled with erroneous or arbitrary labels (see below) may result in difficulties to get the model to converge during training. However, encoding the data in this manner would simplify the application of powerful techniques from language modeling, such as unsupervised pretraining^43,44^. Additionally, this would shorten the output length compared to base-wise encoding, thus reducing the memory required for low-bias transformer architectures that have demonstrated exceptional results across various domains^42,43,45^.

Regarding data, there are both extensive challenges and opportunities for improvement. While there is generally no shortage of total data, obtaining high-quality data that represent a diverse and well-balanced selection of species across phylogenetic groups remains a major issue. We strongly believe that data quality is currently a limiting factor in network performance. This observation is supported by our findings (in ref. ^26^) and the present study, where we observed the lowest scoring predictions in the UTR, which is the class where the highest discrepancy between references and independent RNA-sequencing data was found, probably indicating errors in the reference annotations.

There are several options available to address data quality issues. The conceptually simplest—but poorly scaling—approach is to invest time, money and expertise to improve the quality of reference annotation for the training genomes. Indeed, ongoing efforts to improve annotations can be leveraged by retraining Helixer, with the automated selection of training species enabling Helixer to adapt to even large changes in available input data. An intermediate option would be to use public extrinsic data to identify and differentially weight regions of higher or lower quality in the reference data. The network then learns more from the former than the latter, thereby reducing the noise the network sees while training.

Finally, there are additional modeling options that can be explored such as the unsupervised pretraining mentioned above^43,44^ or pseudo-labeling^46^. These approaches could be combined and used in an iterative approach, where state-of-the-art ab initio predictions from Helixer are incorporated into data-supported pipelines. The resulting state-of-the-art references could, in turn, be used to train a deep learning ab initio caller. Although this approach would be effort intensive, it offers a highly reliable route to achieve major improvement. Another option is to train deep learning models to use RNA-sequencing, CAGE or other extrinsic information as input. This would probably boost performance when both training and inference are performed with the highest-quality extrinsic data. However, handling the potentially high variability of the extrinsic data at inference time will be a challenge.

The above ideas are neither exhaustive nor tested but they highlight the potential for performance gains. It is the hope of the authors that the power of deep learning techniques demonstrated here, and also in other genomics tasks^17,22,45,47^, will promote research interest and improve available resources to the point where they reach a critical mass where modeling tasks previously considered untenable, such as achieving reference quality ab initio annotations, can be accomplished. This will free up time and resources that were previously consumed on using more cumbersome tools, extensive troubleshooting and manual validation and consequently accelerate research progress.

**Fig. 3:**
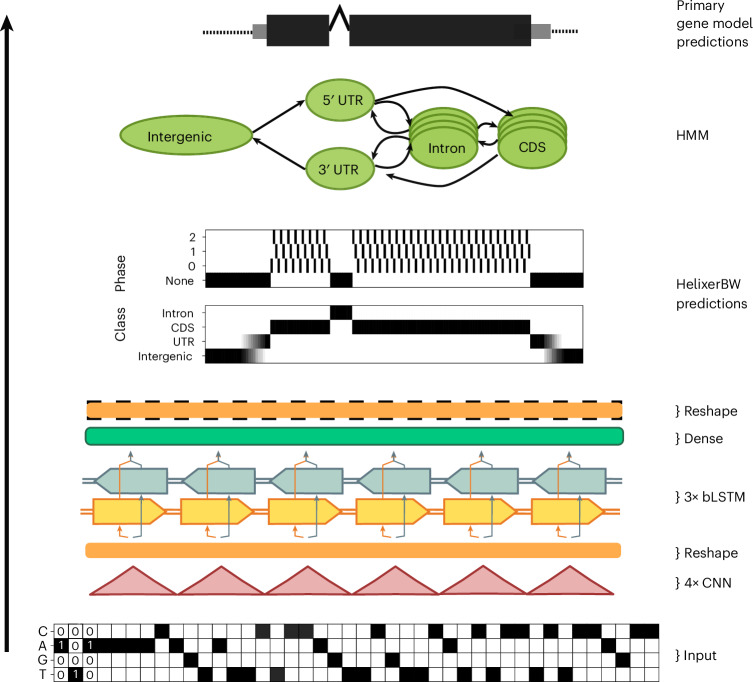
Visual summary of Helixer’s predictive process. The depicted HMM state CDS is further broken down into 10 substates by phase and codon type (start, stop or regular), and the depicted intron state is further broken down into 60 substates by start or continuation of intron, splice motif and outer state. Not to scale. Hyperparameters are examples and can vary.

## Methods

### Data

The foundation of every deep learning application is the data used to train it. Here, we expand the set of genomes to train and evaluate with from 237 in our previous work to 936, including many more plants and vertebrates as well as the new target groups, fungi and invertebrates (Supplementary Table 15). We acquired this data from RefSeq^48^ and Phytozome13^49^ and optimized the assignment of species to training or validation sets in each group (Supplementary Table 16 and Supplementary Methods). We reserved and set aside test set species, only using them for the final evaluation (Supplementary Methods). The exact accessions of all species used during development of Helixer are listed in the Supplementary Tables 17–21 and the different training–validation splits tested are listed in Supplementary Tables 22–25.

GeenuFF (https://github.com/weberlab-hhu/GeenuFF) is a tool to preprocess genome FASTA and annotation GFF3 files and store the cleaned annotations in a SQLite3 database. With GeenuFF, we identify partial gene models and other errors, before generating numerical encodings for the genomic sequence (C, A, T and G), the genic class (intergenic, UTR, coding DNA sequence (CDS) and intron) and coding phase (none, phase 0, phase 1 and phase 2 (indicating the number of base-pairs until the start of the next codon)). We use one-hot encodings, apart from the genomic sequence encoding, which supports ambiguity codes (Supplementary Table 26). Further, for training, we split both input and target data into subsequences of 21,384 bases and apply padding as necessary for shorter contigs or sequence ends. Finally, to prevent padded bases or regions GeenuFF identified as erroneous (Supplementary Methods) from affecting training or evaluation, we mask these base pairs from both the loss and metric calculations.

### Architecture

Helixer (v0.3.0 used here) takes the genomic sequence as input and makes deep learning-based base-wise predictions (referred to as HelixerBW) for the genic class and coding phase. Next, our new HMM, HelixerPost, processes the HelixerBW predictions to generate primary gene models (Fig. 3). This combination allows us to benefit from the powerful pattern recognition of neural networks alongside the structured modeling of eukaryotic gene grammar enabled by HMMs. The probabilistic output of the deep learning stack is leveraged in this approach, capturing the inherent uncertainty associated with genetic structure and used to predict precise gene models.

The deep learning stack takes genomic sequence as input into a convolutional neural network followed by a bidirectional LSTN (bLSTM) hybrid model. This combines the powerful recognition of local patterns of convolutional neural networks with the ability of bLSTM to compress and conserve state over many thousands of inputs. This capability of bLSTMs also allowed us to use longer subsequences of 106,920 bp for plants and 213,840 bp for vertebrates and invertebrates compared to our standard length of 21,384 bp during inference, enabling the inclusion of most genes within a single subsequence (Supplementary Table 27).

The deep learning stack produces two simultaneous outputs: one for the genic class and the other for the coding phase (Fig. 3). We train the model to minimize by base-pair categorical cross-entropy loss but select both hyperparameters and the best model in each training run to maximize genic F1 (‘Metrics’ section) on the validation species. During inference, we predict (by default) on a sliding window of subsequences and use a mean ensemble to combine overlaps, as described in our earlier work^26^.

### HelixerPost

We implemented a postprocessing HMM tool, HelixerPost, that takes the genomic sequence and HelixerBW predictions of genic class and coding phase as input. Using this information, HelixerPost determines the most biologically plausible gene model consistent with these genic class and coding phase predictions for each locus, and outputs a final GFF3 file of primary gene models. HelixerPost first identifies candidate genic regions with consistent low intergenic probability with a sliding window. Within each candidate region, HelixerPost finds the gene model(s) that minimize a penalty for (1) discrepancy between the state of the HMM and Helixer’s predictions and (2) for transitions at sequences conflicting with biological knowledge, such as a start codon that is not at ATG. For a more detailed description, see Supplementary Methods.

It should be noted that the role of the HMM in HelixerPost is relatively limited compared to established gene-finding HMMs. In these cases, the HMM or generalized HMM includes various trained state and state transition likelihoods. In contrast, HelixerPost aims to ensure that each state transition in HelixerBW is resolved at a biologically plausible point, and thus the HMM within HelixerPost needs to encode just well-established biological knowledge.

### Metrics

#### Base wise

We use base-wise F1 metrics^26^. These F1 metrics are calculated as the weighted average of the F1 score for the genic classes UTR, CDS and intron (genic F1), the genic classes CDS and intron (subgenic F1) or the coding phase classes 0, 1 and 2 (phase F1). We calculate the base-wise metrics only on bases that are not padding and, additionally in this work, not masked by GeenuFF (for details, see Supplementary Methods).

For each training and validation genome, we calculate the F1 scores on a random sample of 800 subsequences, while for test genomes, we calculate F1 scores genome wide

1 $${\rm{Precision}}=\frac{{\mathrm{TP}}}{{\mathrm{TP}}+{\mathrm{FP}}}$$

2 $${\rm{Recall}}=\frac{{\mathrm{TP}}}{{\mathrm{TP}}+{\mathrm{FN}}}$$

3 $${\rm{F1}}=2\times \frac{{\rm{precision}}\times {\rm{recall}}}{{\rm{precision}}+{\rm{recall}}},$$

where TP is true positive, FP is false positive and FN is false negative.

#### Feature or full model

Complementary to the base-wise metrics, we employ both reference-centric and homology-based metrics to capture performance at the level of full features and gene models. With GffCompare (0.12.8)^50^, we calculate feature and feature combination-level precise matches against the reference for the primary (longest protein) transcripts with all UTRs removed. We removed UTRs because GffCompare does score a true positive if every feature is exactly identical to the reference. All tools approached 0% exactly identical UTRs and therefore we excluded them.

The homology-based methods also support evaluation of the reference. We estimate proteome completeness with BUSCO (5.2.2)^27^. For the plant test genomes, we further assigned Mapman4 protein categories^51^ with Mercator4 (5.0)^52^ to quantify both completeness and annotatability by comparison to curated gene families. Additionally, we assessed the comparability between proteomes by clustering test plant proteomes into orthogroups with OrthoFinder (2.5.4)^53^. We focused on 12 plant test genomes (those that finished in 1 week for GeneMark-ES), and double-checked consistency for all 13 species with only the references and Helixer’s predictions. Finally, we performed a detailed analysis for *A. thaliana* (for details, see Supplementary Methods).

### Species selection

While developing Helixer, we found that the generalization performance of a trained model is highly dependent on both the quality and the quantity of the training genomes. Finding the optimal tradeoff proved to be difficult and often a bit counterintuitive. To automatically obtain more generalizable, higher-quality and consistent models, we implemented a custom hyperparameter optimization for splitting training and validation genomes. This comprises selecting promising random sets of training genomes via twofold cross-validation and then remixing the best genomes from the folds for a final evaluation (Supplementary Methods and Supplementary Table 16).

### Incorporation of biologically important features into the loss function

The loss function is the optimization criteria for the network and must thus be as close to the actual goal of a project as possible. Vanilla base-wise categorical cross-entropy treats all bases equally and does not proportionally reflect the importance of some mistakes (for example, those inducing frame shifts). We tuned the loss function to push the network toward more biologically plausible predictions. First, we implemented different weights for each class to be computed in the loss function. This counteracted the fact that the intergenic class is the most common, but also the one we care the least about. We then moved to increase these weights if they lie directly before or after one of four class transition sites (start codon, stop codon, donor splice site or acceptor splice site). This was done as the model had previously little incentive to output very sharp predictions around these transitions, which occur very infrequently in the data (see parameters class_weights and transition_weights in Supplementary Methods and Supplementary Tables 28–32). Finally, we added the phase output described above to capture phase directly in the loss and to support HMM postprocessing (see below). The overall loss is the weighted sum of the primary loss (weight of 0.8) and the categorical cross-entropy loss of the phase predictions (weight or 0.2).

### Annotation quality comparison

We employed two established HMM tools to set baseline expectations for an ab initio gene calling tool. Where trained models were available, we used AUGUSTUS (3.3.2)^6^ as a high-performance baseline (Supplementary Table 33), but for feasibility reasons, we did not retrain here. We did however perform an additional comparison running AUGUSTUS (3.5.0) with softmasking enabled and softmasked input genomes (Supplementary Fig. 17 and Supplementary Tables 8–11). Therefore, for the complete test set, we employed GeneMark-ES (4.71_lic)^2,3^, which uses unsupervised training. Furthermore, we compared a Helixer to the similar DNN Tiberius version 1.1.4^25^ with and without softmasking enabled. For this purpose, a mammal only model was trained using the same species as Tiberius (Supplementary Table 21). Helixer inference details can be found in Supplementary Tables 27 and 33 and Supplementary Methods. For comparing Helixer’s prediction to alternative tools and the reference, we use only the splice variants producing the longest protein, unless otherwise specified.

### Ablations

During ablations, we compared the best plant model to plant models trained without phase, with lower transition weights or with an LSTM instead of hybrid model. All training parameters differing from land_plant_v0.3_a_0080 as well as ablation-specific inference parameters are listed (Supplementary Tables 35 and 36, respectively). Ablations were evaluated on all plant test genomes except the very large *T. dicoccoides*.

### Model releases

For this paper, we evaluated and documented all models released during development, including the training parameters (Supplementary Tables 28–32) and training species (Supplementary Tables 21–25). Released models are named as lineage_release_key_rank, where the lineage indicates the appropriate application lineage of the trained model; the release indicates the code version with which it is compatible; the key is a brief marker of ‘a’ for automatic species selection and ‘m’ for manual, which can be either entirely manual or automatic followed by manual tuning; and rank indicates relative model performance within the aforementioned categories (lower is better).

### Reporting summary

Further information on research design is available in the Nature Portfolio Reporting Summary linked to this article.

## Online content

Any methods, additional references, Nature Portfolio reporting summaries, source data, extended data, supplementary information, acknowledgements, peer review information; details of author contributions and competing interests; and statements of data and code availability are available at 10.1038/s41592-025-02939-1.

## Supplementary information

Supplementary Information Supplementary Methods, Figures and Tables.

Reporting Summary

## Figures and Tables

**Extended Data Table 1:**
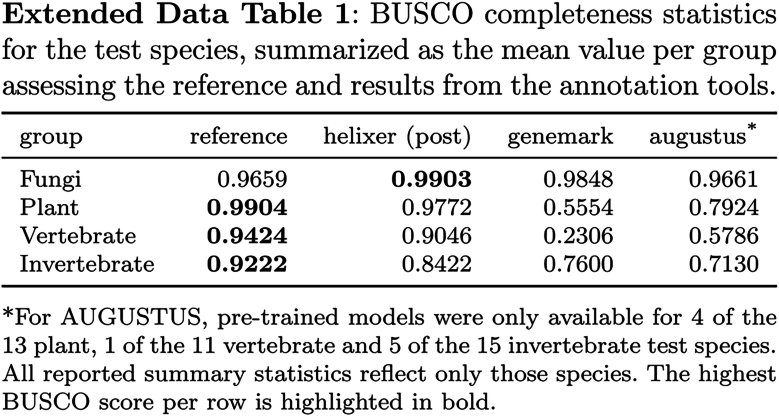
BUSCO completeness statistics

BUSCO completeness statistics for the test species, summarized as the mean value per group assessing the reference and results from the annotation tools.

## Data Availability

The data used in this study were acquired from public databases. Accession numbers can be found in Supplementary Tables 17–20. The pretrained models are available via GitHub at https://github.com/usadellab/Helixer or via Zenodo at 10.5281/zenodo.10836346 (ref. ^54^).

## Code availability

Helixer’s source code is available via GitHub at https://github.com/usadellab/Helixer. The code is available via Zenodo at 10.5281/zenodo.17404832 (ref. ^55^). The source code for HelixerPost is available via GitHub at https://github.com/Usadellab/HelixerPost and also via Zenodo at 10.5281/zenodo.17414354 (ref. ^56^). Helixer’s online web interface is available at https://www.plabipd.de/helixer_main.html.
