# Supplementary material for: Helixer: ab initio prediction of primary eukaryotic gene models combining deep learning and a hidden Markov model
Source: Nat Methods. 2025 Nov 24;23(4):732–9. doi: 10.1038/s41592-025-02939-1 (PMC13076211; doi:10.1038/s41592-025-02939-1)
Supplement: Supplementary file 1 — Supplementary Methods, Figures and Tables. [file 41592_2025_2939_MOESM1_ESM.pdf]

nature methods

Supplementary information

https://doi.org/10.1038/s41592-025-02939-1

Helixer: ab initio prediction of primary eukaryotic gene models combining deep learning and a hidden Markov model

In the format provided by the authors and unedited

Contents

S1 Supplemental Methods **4**
S1.1 Supplemental Data 4
S1.1.1 Data origin and splits 4
S1.2 Supplemental Architecture 5
S1.2.1 HelixerPost 5
S1.3 Supplemental Metrics 7
S1.3.1 GeenuFF masking 7
S1.3.2 *A. thaliana*-annotation comparison 7
S1.4 Supplemental Species Selection 8
S1.5 Supplemental Incorporation of biologically important features into the loss function 8
S1.5.1 Transition Weights 8
S1.6 Supplemental Annotation quality comparison 8
S1.6.1 Masking genomes for AUGUSTUS and Tiberius predictions 8
S1.6.2 Running gene prediction tools 9
S1.6.3 Cleaning predictions 10
S1.6.4 Running comparison 10
S1.7 Benchmarking 12

S2 Supplemental Figures **13**
S2.1 Vertebrate, Invertebrate, Plant and Fungi Model Comparison (Genic F1) 13
S2.2 Plant Performance With Species Labels 18
S2.3 Relative Performance of Annotations measured with F1 20
S2.4 Intron precision and recall 25
S2.5 Comparison to Augustus using softmasking 26
S2.6 Relative Performance of Annotations measured with BUSCO 27
S2.7 Ablations 30
S2.8 Orthogroup based quality control 31
S2.9 *Arabidopsis thaliana* 34

S3 Supplemental Tables **37**
S3.1 Tabular Results 37
S3.2 Data 59
S3.2.1 Full Datasets 59
S3.2.2 Training species sets 79
S3.2.3 Encoding 84
S3.3 Training Parameters 85
S3.3.1 Model parameters 85
S3.3.2 Additional Information 88

S4 Supplemental Discussion **88**

S1 Supplemental Methods

S1.1 Supplemental Data

S1.1.1 Data origin and splits

Due to the iterative nature of development, datasets were acquired from different sources at different times, as listed below.

Fungi Fungus data for training, validation, and testing were acquired from RefSeq on March 4th, 2022. Test genomes were assigned randomly *within* genomes that had a matching or close AUGUSTUS trained model available and *within* those that did not.

All species assigned to test that had a close AUGUSTUS model were actually used as test species in this manuscript, all other test species remain reserved.

Exact species, versions, and set assignments are listed in Tab. S17.

Plants Plant training and validation genomes were acquired from Phytozome13 on June 7th 2021.

The plant test genomes were acquired from RefSeq on July 14th 2022. They exclude any species present in the training validation set, include genomes with matching or close AUGUSTUS models and several more that were expected to have acceptable quality genomes based upon crop impact and researcher experience.

Thus, these genomes were selected for comparability with other tools, as well as to have acceptable quality for analyses that rely on the reference (the ablation analyses).

Selection was performed prior to downloading, thus other RefSeq genomes meeting e.g. just the first criteria above are not listed, in contrast to the other phylogenetic groups.

Exact species, versions, and set assignments are listed in Tab. S18.

Vertebrates Vertebrate training, validation, and test genomes were acquired from RefSeq on May 6th, 2022; and are the combined RefSeq sets of 'vertebrate_mammalian' and 'vertebrate_other'.

Species previously used in Stiehler et al. (2020) were assigned to the training and validation set, after that test genomes were assigned randomly *within* genomes that had a matching or close AUGUSTUS trained model available and *within* those that did not.

All species assigned to test that had a close AUGUSTUS model were actually used as test species in this manuscript, additional used test species were selected arbitrarily (every Nth).

Exact species, versions, and set assignments are listed in Tab. S19.

Invertebrates Invertebrate training, validation and test genomes were acquired from RefSeq on May 6th, 2022.

Test genomes were assigned randomly *within* genomes that had a matching or close AUGUSTUS trained model available and *within* those that did not.

All species assigned to test that had a close AUGUSTUS model were actually used as test species in this manuscript, additional used test species were selected arbitrarily (every Nth).

Exact species, versions, and set assignments are listed in Tab. S20.

Mammals Mammal training, validation and test genomes were acquired from RefSeq on March 13th, 2025.

The same species selection and partition into training, validation and test set was used as for the Tiberius mammal model [Gabriel et al., 2024].

Exact species, versions, and set assignments are listed in Tab. S21.

S1.2 Supplemental Architecture

S1.2.1 HelixerPost

HelixerPost makes a double pass on each strand of the genome. In the first pass, probable genic regions are identified from the Helixer base-wise (HelixerBW) predictions. These are regions where the average non-intergenic prediction of a sliding window (recommended 100bp) stays above a given threshold (recommended 0.1) and peaks at or above a higher threshold (recommended 0.8).

In the second pass, the primary gene model(s) most consistent with the HelixerBW predictions in each genic region are determined using a Markov Model which encodes biologically plausible states and transitions. The Markov Model states include those representing intergenic, 5' UTR, CDS, and 3' UTR sequence. Specific CDS states are used to represent start and stop codons. Coding phase is also encoded within the states for start, regular and stop codons, thus requiring 3 start codon states, 3 regular codon states and, perhaps surprisingly, 4 stop codon states, with the additional stop state needed to distinguish between TAx and TGx partial stop codons. Intron state is encoded using an additional set of substates, for both UTR states and 8 of the 10 CDS states. The Start2 and Stop2 states do not need additional intron states since introns starting immediately after start and stop codons are functionally equivalent to introns starting immediately after regular codons, and anywhere in the 3' UTR respectively.

Three different forms of intron splicing are represented, GT-AG, GC-AG, both of which are mediated by U2, and AC-AT as mediated by U12. Two states are used for each intron form, representing the start and continuation of each intron form, thus requiring 6 additional states for each primary state. Thus, in total, the HMM uses 73 states, with 1 intergenic, 2 UTR states, 10 CDS states, and 6 additional intron substates each for 10 of the 12 UTR/CDS states.

Minimal intron lengths are implemented by pausing the HMM state for additional bases when an intron start state is detected (49 additional bases for U2 mediated introns and 29 for U12 mediated introns). In all other cases, consumption of each base is associated with a HMM state transition, according to set of possible valid state transitions.

HMM transition and emission probabilities are implemented by accruing penalties when less likely state transitions / emissions are evaluated, i.e. when the HMM state doesn't match HelixerBW genic class or phase predictions, or when biologically implausible state transitions occur.

HMM emission penalties are based on the relationship between the HelixerBW predictions and the HMM state, and in general multiple HMM states correspond to one HelixerBW prediction class and phase. If the HMM state corresponds to the most likely genic class and phase in HelixerBW's prediction, no emission penalty is accrued. Otherwise, the probability difference between the HMM inferred genic class / phase and HelixerBW's most likely prediction genic class / phase is used to calculate the penalty.

HMM transition penalties were hand picked based on existing biological knowledge and are, in many cases, conditional the surrounding sequence context. These transition penalties may either reward or penalize certain transitions, or permit any of several different transitions without penalty. An example of the latter is transitioning from a UTR 5' state to a coding state which has no penalty at a start codon, but remaining in the UTR 5' state is also permitted without penalty - this is biologically valid since the presence of a start codon is necessary but not sufficient for translation. Intron splicing is similar, where a valid splice boundary allows splicing, but does not require it. On the other hand, in-phase stop codons are generally respected (ignoring their role in non-standard translation), therefore transitioning from a coding to the UTR 3' state is encouraged at stop codons by penalizing remaining in a coding state.

HelixerPost processes each strand independently and concatenates the results.

HelixerPost as an Optimization Problem The optimal scoring path of states s through this Markov Model for a given underlying sequence window and HelixerBW predictions is determined with the Viterbi algorithm [Viterbi, 1967].

More precisely, HelixerPost determines a sequence of states across the base positions which minimizes the penalty P, for

$$P = \sum_{w=1}^{W} (normpen_{w,s} + C_{w,s})$$

where W is the length of the candidate window, $normpen_{w,s}$ is a penalty score based on Helixer's base-wise prediction at base w for the class (and for coding state, also the phase) corresponding to state s and $C_{w,s}$ is a penalty for discrepancy between states and/or state transitions vs those expected based on the sequence context.

Normalized Prediction Penalty Matrix The normalized penalty score, $normpen_{w,s}$, referenced above, is a per-base penalty matrix derived from Helixer's predictions by applying the following process at each base position w:

First, Helixer's state (Intergenic, UTR, Coding or Intron) and phase (0,1,2) predictions are combined into a single 6-way prediction (Intergenic, UTR, Coding0, Coding1, Coding2 or Intron) using a 0.8 state, 0.2 phase weighting for the input prediction matrices, as follows:

The input is the Helixer state prediction matrix $hstate$ and phase prediction matrix $hphase$

$$hstate = \begin{Bmatrix} hstate\left[intergenic\right] \\ hstate\left[utr\right] \\ hstate\left[coding\right] \\ hstate\left[intron\right] \end{Bmatrix} \qquad hphase = \begin{Bmatrix} hphase\left[noncoding\right] \\ hphase\left[phase0\right] \\ hphase\left[phase1\right] \\ hphase\left[phase2\right] \end{Bmatrix}$$

Scale the phase predictions to ensure their total matches $hstate\left[coding\right]$:

$$phaseScaleFactor = hstate\left[coding\right] / (hphase\left[phase0\right] + hphase\left[phase1\right] + hphase\left[phase2\right])$$

$$scalephase\left[phase0\right] = hphase\left[phase0\right] * phaseScaleFactor$$

$$scalephase\left[phase1\right] = hphase\left[phase1\right] * phaseScaleFactor$$

$$scalephase\left[phase2\right] = hphase\left[phase2\right] * phaseScaleFactor$$

Calculate the weighted predictions for phase-specific coding, with $StateWeight = 0.8$ and $PhaseWeight = 0.2$

$$phase\left[phase0\right] = hstate\left[coding\right] * StateWeight + scalephase\left[phase0\right] * PhaseWeight$$

$$phase\left[phase1\right] = hstate\left[coding\right] * StateWeight + scalephase\left[phase1\right] * PhaseWeight$$

$$phase\left[phase2\right] = hstate\left[coding\right] * StateWeight + scalephase\left[phase2\right] * PhaseWeight$$

Then the negative log is calculated for each of the 6 possibilities to create an associated penalty score, and the minimal penalty score determined.

$$pen\left[intergenic\right] = -log_2(hstate\left[intergenic\right])$$

$$pen\left[utr\right] = -log_2(hstate\left[utr\right])$$

$$pen\left[phase0\right] = -log_2(phase\left[phase0\right])$$

$$pen\left[phase1\right] = -log_2(phase\left[phase1\right])$$

$$pen\left[phase2\right] = -log_2(phase\left[phase2\right])$$

$$pen\left[intron\right] = -log_2(hstate\left[intron\right])$$

$$minPen = min(pen\left[intergenic\right], pen\left[utr\right], pen\left[phase0\right], pen\left[phase1\right], pen\left[phase2\right], pen\left[intron\right])$$

The minimal penalty is then subtracted from all penalty scores, to form a normalized penalty matrix where the highest probability Helixer prediction has a penalty of 0.

$$normpen = \begin{Bmatrix} pen\left[intergenic\right] - minPen \\ pen\left[utr\right] - minPen \\ pen\left[phase0\right] - minPen \\ pen\left[phase1\right] - minPen \\ pen\left[phase2\right] - minPen \\ pen\left[intron\right] - minPen \end{Bmatrix}$$

Sequence Context Penalty The second component of the HelixerPost scoring function is C, a pre-defined penalty based on the biological plausibility of the HMM state and/or state transition, given the sequence context. In most cases, this serves to penalize transitions when the expected context sequence is absent, such as a start codon without the expected ATG. Similar penalties are applied for stop codons and intron donor / acceptor sites. In addition, failure to take the expected stop codon transitions in the presence of in frame TAA / TAG / TGA sequences is penalized.

S1.3 Supplemental Metrics

S1.3.1 GeenuFF masking

GeenuFF v0.3.1 is used to intermittently store annotation features in a consistent, and explicit fashion from which one can readily generate various encodings. Moreover, it generates error masks for erroneous or ambiguous regions from a GFF3 file. More information can be found here: https://weberlab-hhu.github.io/GeenuFF/.

The majority of GeenuFF error masks are placed in the region up- or downstream from an apparently partial gene model. These masks are used for both the loss function, so that the models do not learn from these suspected errors, and the calculation of all base-wise metrics, so that neither the Helixer models nor the comparative tools are penalized for the most obvious and detectable errors in the reference.

The following errors in the reference cause a mask going from the error source into the (implied) intergenic region of length M, where

$$M = \min(l/2, \text{int}(\sqrt(l)) \cdot 10)$$

and l is the length of the (implied) intergenic region until the next gene. If there is no gene between the error and the sequence end, then the mask extends until the end of the sequence.

We say '(implied) intergenic' because although classical parsing of the gff3 file would interpret the space between two genes as intergenic. The apparently partial gene models indicate this region is likely partially genic, and is ultimately ambiguous.

- **missing_utr_5p**–Inserted when the 5' most exon coordinate matches the 5' most CDS coordinate. Masks into the upstream (implied) intergenic
- **missing_utr_3p**–Inserted when the 3' most exon coordinate matches the 3' most CDS coordinate. Masks into the downstream (implied) intergenic
- **missing_start_codon**–Inserted when the 5' most 3bp of the CDS are not ATG. Masks from the 5' CDS end into the upstream (implied) intergenic
- **missing_stop_codon**–Inserted when the 3' most 3bp of the CDS are not a TAA TGA or TAG. Masks from the 3' CDS end into the downstream (implied) intergenic
- **wrong_starting_phase**–Inserted when the 5' most bp of the CDS is not phase 0. Masks from the 5' CDS end into the upstream (implied) intergenic
- **mismatched_ending_phase**–Inserted when the phase listed in the gff features at CDS 3' end, does not match expectations based on starting phase and cumulative CDS length. Masks from the 3' CDS end into the downstream (implied) intergenic
- **truncated_intron**–Inserted when the 5' or 3' most bp of an intron matches the 5' or 3' end of a gff transcript feature. Masks from the 5' or 3' end into the upstream or downstream (implied) intergenic, respectively.

Additionally the following errors are masked as described below.

- **overlapping_exons**–Inserted when the coordinates of two exons in one transcript overlap. The overlapping region is masked.
- **mismatching_strands**–Inserted when two transcripts within one gene have different strands. The entire gene feature is masked.
- **too_short_intron**–Inserted when an intron of less than the minimum length (20bp here, configurable) is detected. The whole short intron is masked.
- **super_loci_overlap_error**–This is technically not a new error, but an extension of any of the 5' or 3' errors above. Inserted when two genic loci overlap, and have errors on the overlapping end. Errors are extended into the (implied) intergenic as above, except that l is calculated based on the distance the next *non*-overlapping gene.

S1.3.2 *A. thaliana*-annotation comparison

We performed a detailed comparison of the structural annotations from Helixer to TAIR10 and Araport11 for the model species *A. thaliana*; the results are reported in section 'Filling gaps in *A. thaliana*' of the main text.

As Helixer predicts only a single splice variant per locus, the number of gene loci was used for counting purposes. First, identical protein sequences were identified from each reference annotation versus Helixer. If any splice variant from

the comparison reference genome was present in the Helixer set, it was labelled as ‘identical’. The remaining set of sequences were tested using BLAST (E-value 10^{-8}, Altschul et al. [1997]) to find homologous sequences between the reference and Helixer. This identified similar protein-coding sequences between each pairwise comparison. Finally, the remaining sequences were compared to sequences from 420 genome projects representing all streptophytes clades, to identify orthologous sequences. Orthologous candidates were verified by triangles of Reciprocal Best Hits [Tatusov et al., 1997].

For the main text figure, the same RNAseq data and processing was used as in [Stiehler et al., 2020].

S1.4 Supplemental Species Selection

Details on automated selection of training species from a given group of non-test genomes are as follows.

At first during species selection, the available training and validation genomes were divided randomly into two folds. Second, within each fold, a number (Tab.S16) of models were trained each on randomized divisions of the fold into training and validation genomes. Third, the performance (genic F1) of each model was evaluated on all the genomes in the other fold. Fourth, the best two divisions of training and validation in each fold were recombined. So given the best two splits in fold A as A1 and A2, and the best two splits in fold B as B1 and B2, models were then trained on A1 + B1, A1 + B2, A2 + B1, A2 + B2. All remaining non-test species were used for validation. Fifth, final evaluation was performed on the intersect of all validation sets from step four, and this was then used for the final ranking of the models. The top models can be used for ensembling, but here the best model was used alone unless otherwise specified.

This was implemented here: `https://github.com/alisandra/SpeciesSelector`

As necessary, a last round of human expertise and hyperparameter optimization was employed to finalize the selection.

S1.5 Supplemental Incorporation of biologically important features into the loss function

S1.5.1 Transition Weights

Previously, we weighted all non-masked base pairs in an annotation class equally, and the network could achieve a low loss by predicting the broad regions correctly and confidently, while accumulating only a small penalty for comparatively few uncertain predictions around class transitions. However, biologically speaking, four of the class transitions (start codon, stop codon, donor splice site and acceptor splice site) can cause a non-sense prediction if they are off by even a single base pair by introducing a frame shift in the final protein sequence. To better reflect the biological importance of these transitions in the loss function, we implemented transition weighting; which up-weighted the loss around start and stop codons, as well as donor and acceptor splice sites. The transcript start and end were not up-weighted as preliminary analyses (not shown) indicated this was not beneficial, potentially due to the extreme noise in the transcript start and end labels be it from errors, biological variation, or both.

In more detail: For all transitions transcription start site, start codon, donor splice site, poly-adenylation site, stop codon, acceptor splice site the indices of last base pair of the proceeding class, and the first base pair of the next class were identified. Where *any* transition occurred in a pool, the whole pool was up-weighted (generally $12\times$ for start & stop codons, and $3\times$ for splice sites). Where two transitions occurred in the same pool (e.g. start codon and donor splice site), weights were summed. Non-transition pools received a weight of 1. The resulting weights were used to multiply the sample weights (which are additionally adjusted according to error-masks as previously described.)

S1.6 Supplemental Annotation quality comparison

S1.6.1 Masking genomes for AUGUSTUS and Tiberius predictions

We performed a comparison between Helixer and the two *ab initio* gene annotation tools AUGUSTUS and Tiberius (Fig. S17, Tab. S8 - S11). Both tools support the input of a softmasked genome to improve prediction quality. Softmasking was performed with RepeatModeler2 version 2.0.6 [Flynn et al., 2020] and RepeatMasker version 4.1.7-p1 [Smit et al., 2013–2025] for the comparison with both tools. The commands were as follows:

```
# RepeatModeler2
BuildDatabase -name "$(basename $fasta .fna)_db" $fasta
RepeatModeler -database "$(basename $fasta .fna)_db" -threads 56 -LTRStruct

# RepeatMasker
RepeatMasker -pa 56 -lib "$(basename $fasta .fna)_db-families.fa" -xsmall $fasta
```

In the Tiberius paper [Gabriel et al., 2024], an extra masking step with Tandem Repeats Finder (TRF) [Benson, 1999] was performed. So, the input genomes for Tiberius were additionally masked with TRF version 4.10.0 as described in the Tiberius paper, additionally utilizing BedTools version 2.31.1 [Quinlan and Hall, 2010] and the scripts 'parseTrfOutput.py' from the BRAKER2 pulication [Brůna et al., 2021] (https://github.com/gatech-genemark/BRAKER2-exp/blob/master/bin/trf-scripts/parseTrfOutput.py) and 'splitMfasta.py' from AUGUSTUS version 3.5.0. The commands were as follows:

```
genome="<genome_name>.fna.masked"
bs="<genome_name>_final.fna.masked"

mkdir -p trf
cd trf
perl ../splitMfasta.pl --minsize=25000000 $genome
ls $bs.split.*.fa | parallel 'TRF/build/src/trf {} 2 7 7 80 10 50 500 -d -m -h &> \
{}.log'
ls $bs.split.*.fa.2.7.7.80.10.50.500.dat | parallel 'parseTrfOutput.py {} \
--minCopies 1 --statistics {}.STATS > {}.raw.gff 2> {}.parsedLog'
ls $bs.split.*.fa.2.7.7.80.10.50.500.dat.raw.gff | parallel \
'sort -k1,1 -k4,4n -k5,5n {} \
> {}.sorted 2> {}.sortLog'
FILES=$bs.split.*.fa.2.7.7.80.10.50.500.dat.raw.gff.sorted
for f in $FILES; do
bedtools-2.31.1/bin/mergeBed -i $f | awk 'BEGIN{OFS="\t"} \
{print $1,"trf","repeat",$2+1,$3,".",".",".","."}' \
> $f.merged.gff 2> $f.bedtools_merge.log
done
ls $bs.split.*.fa | parallel 'bedtools-2.31.1/bin/maskFastaFromBed \
-fi {} -bed {}.2.7.7.80.10.50.500.dat.raw.gff.sorted.merged.gff \
-fo {}.combined.masked \
-soft &> {}.bedools_mask.log'
cat $bs.split.*.fa.combined.masked > $bs.fa.combined.masked
```

S1.6.2 Running gene prediction tools

AUGUSTUS: AUGUSTUS was installed from GitHub (https://github.com/Gaius-Augustus/Augustus) and run with the following command:

```
augustus --species=<augustus_species> <genome_assembly.fa> --softmasking=1 \
--noInFrameStop=true --stopCodonExcludedFromCDS=false \
--gff3=on --UTR=on > <output>.gff3
```

The input genomes were softmasked previously like described in Sec. S1.6.1 in one instance and not for the other, producing two sets of predictions per species. The UTR mode was only on if the given AUGUSTUS model suppoted it.

GeneMark: GeneMark was downloaded and installed from the official website (http://topaz.gatech.edu/GeneMark/license_download.cgi) and run with the following command:

```
gmes_petap.pl --sequence <genome_assembly.fa>  --ES --cores 24
```

The option `--fungus` was added for fungal genomes.

Tiberius: Tiberius was installed from GitHub (https://github.com/Gaius-Augustus/Tiberius) and run with the following command:

```
tiberius.py --genome <genome_assembly.fa> --out <output>.gtf --seq_len 259992 \
--protseq <output>.faa --batch_size 4
```

The input genomes were softmasked previously like described in Sec. S1.6.1 in one instance and not for the other, producing two sets of predictions per species. For the non-softmasking runs the flag `--no_softmasking` was added.

Helixer: Helixer was intsalled from GitHub (https://github.com/weberlab-hhu/Helixer) alongside Helixer-Post (https://github.com/TonyBolger/HelixerPost) and run with the following command:

```
Helixer.py --model <model> --fasta-path <genome_assembly.fa> \
--species <species> --subsequence-length <length> \
--gff-output-path <output>.gff3  --temporary-dir <tmp>
```

For the subsequence length and subsequently calculated overlap parameters see Tab. **??**. The models used were vertebrate_v0.3_m_0080, invertebrate_v0.3_m_0100, land_plant_v0.3_a_0080, fungi_v0.3_a_0100 and mammal_v0.3_a_0400. The default parameters for HelixerPost were used (see Tab. S34).

S1.6.3 Cleaning predictions

Both GeneMark and Tiberius produced GTF files that needed to be converted to GFF files before further comparisons could be performed. We used GffRead [Pertea and Pertea, 2020] and two custom scripts. The script `fix_gm_names.py` (`https://github.com/weberlab-hhu/helixer_scratch/blob/master/data_scripts/fix_gm_names.py`) for GeneMark and the script `correct_tiberius_gtf_format.py` (`https://github.com/weberlab-hhu/helixer_scratch/blob/master/data_scripts/correct_tiberius_gtf_format.py`) for Tiberius. We used the following commands:

GeneMark:

```
gffread genemark.gtf -o genemark.gff3

# fix ID names
python3 fix_gm_names.py genemark.gff3 > <species>_genemark.gff3
```

Tiberius:

```
# fix transcript names
correct_tiberius_gtf_format.py -i tiberius.gtf -o tiberius_fixed.gtf

gffread tiberius_fixed.gtf -o tiberius.gff3
```

The predictions from AUGUSTUS created with UTRs=off and gff3=on were corrected by inserting exons for every CDS and changing transcript features to mRNA. We used the script `clean\_UTRoff\_gff3.py` (`https://github.com/weberlab-hhu/helixer_scratch/blob/master/method_comp/clean_UTRoff_gff3.py`).

```
python3 clean_UTRoff_gff3.py --gff-in <augustus_annotation_utr_off>.gff3 \
--out <cleaned_annotation>.gff3
```

S1.6.4 Running comparison

Inference for test species was performed with an ensemble (element-wise average of raw predictions) of the best two models for fungi (fungi_v0.3_a_0100 and fungi_v0.3_a_0200), and due to time constraints for developing and evaluating the models with only the single best model (default) for the other three groups.

BUSCO was run as follows:

```
bash busco_local.sh <genome_assembly.fa> <genome_annotation.gff> <busco_lineage>
```

Contents of `busco_local.sh`:

```
genome_fa=$1
gff=$2
lineage=$3

# activate python environment where BUSCO is installed
source <busco_virtual_environment>/bin/activate
# path to the busco_downloads folder where all BUSCO datasets are located
# when BUSCO is installed locally
bp=<path_to_BUSCO>/busco_downloads

# find source files
annodir=‘echo $gff |sed ’s@/[^/]*gff3@@g’‘
```

```
proteins=$annodir/protein.fa

# extract protein sequences from the GFF file
gffread $gff -g $genome_fa -y $proteins

# create output directory and remove a potential old one
busco_out=$annodir/busco
rm -r $busco_out
mkdir -p $busco_out

# run BUSCO
busco --in $fa_in --out $busco_out --offline --mode "prot" -l $lineage \
-f --download_path $bp
```

See also Tab. S34 for details on BUSCO lineages used.

GffCompare: Since Helixer only predicts the splice variants producing the longest protein, we first filter out all other splice variants from annotations of other gene callers and the reference with the script `lala_longest.py` (`https://github.com/weberlab-hhu/helixer_scratch/blob/master/misc_scripts/lala_longest.py`).

```
lala_longest.py --gff-file <gff_to_filter> > <filtered_gff>
```

Then GffCompare was run as follows:

```
./gffcompare.sh <refrence.gff3> <alternate.gff3> <output_directory>
```

Contents of `gffcompare.sh`:

```
#! /bin/bash
ref=$1
gff=$2
outdir=$3

mkdir -p $outdir
mkdir -p tmp_gffcompare_cleaner
cat $ref|awk '$3 != "exon"' |awk '$3 != "five_prime_UTR"'| \
awk '$3 != "three_prime_UTR"' > tmp_gffcompare_cleaner/reference.gff3
cat $gff|awk '$3 != "exon"' |awk '$3 != "five_prime_UTR"'| \
awk '$3 != "three_prime_UTR"' > tmp_gffcompare_cleaner/alternate.gff3
gffcompare -r tmp_gffcompare_cleaner/reference.gff3 \
-o $outdir/gffcompare \
tmp_gffcompare_cleaner/alternate.gff3

rm tmp_gffcompare_cleaner/reference.gff3*
rm tmp_gffcompare_cleaner/alternate.gff3*
```

Base-wise Metrics: We compared the three different annotation categories genic (UTR, CDS, Intron), subgenic (CDS and Intron) and phase (None, phase 0, phase 1, phase 2). We used precision, recall and F1 to evaluate the accuracy of the gene predictions of AUGUSTUS, GeneMark and Helixer versus the reference. To calculate these metrics base-wise, we followed a three-step workflow. Converting the genome annotations to sqlite3 with GeenuFF (`https://github.com/weberlab-hhu/GeenuFF`):

```
import2geenuff.py --gff3 <genome_annotation.gff> --fasta <genome_assembly.fa> \
--db-path <species.sqlite3> --log-file import.log --species <species>
```

Importing the sqlite3 databases to h5 files with Helixer's `geenuff2h5.py`:

```
geenuff2h5.py --h5-output-path <species>/test_data.h5 \
--input-db-path <species.sqlite3> \
--subsequence-length 21384 --write-by 2138400
```

Computing accuracy metrics files with `accs_genic_intergenic.py` (`https://github.com/weberlab-hhu/Helixer/blob/main/scripts/accs_genic_intergenic.py`):

```
python Helixer/scripts/accs_genic_intergenic.py \
--data <species>.h5 --predictions <species>/test_data.h5 \
--h5_prediction_dataset 'data/y' \
--stats_dir summary/<lineage>/<species>/vs_ref/<gene_caller>
```

Mercator4: Mercator4 was run online `https://www.plabipd.de/mercator_main.html` for all plant test species. The selected sequence type was protein and the databases Prot-scriber and Swissprot were included in the analysis.

S1.7 Benchmarking

The computational load for Helixer comprises three main parts, namely conversion of input sequence into numerical form, prediction with the neutral network, and the HMM for post-processing of the predictions. The first two are expected to scale linearly with genomic sequence length with the qualifier that here, the length includes any padding. The HMM is expected to scale linearly with the cumulative length of candidate gene regions.

Benchmarking was performed on the following machines. A) Workstation with an AMD Ryzen 9 5950X CPU, GeForce GTX 1080 Ti graphics card (11GB memory), 32GiB 2133MHz DDR4 RAM, and a Samsung 980 PRO 2TB NVMe SSD. B) Workstation with Intel(R) Xeon(R) CPU E5-2623 v4 @ 2.60GHz, GeForce GTX 1080 Ti graphics card (11GB memory), 64GiB 2400MHz DDR4 ECC RAM, and an INTEL SSDSC2BB24 SSD. C) for comparison between Helixer and the other tools on a workstation with Intel(R) Xeon(R) W-2125 CPU @ 4.00GHz, 32GB (4x 8GB) DDR4 PC2666 ECC RAM, GeForce GTX 1080 graphics card (8GB memory), and an Intel D3-S4510 240GB SSD. All benchmarks were performed single-threaded. All machines used lzf compression and a temporary dir to a path on an SSD, which improves performance where the installation and hardware, respectively, allow. Respective batch sizes were 64 for machines A and B and 58 for machine C; the highest batch size that fits in GPU memory should be fastest. No multiprocess was set for interpretability of the resulting numbers and a more fair comparison between tools.

S2 Supplemental Figures

S2.1 Vertebrate, Invertebrate, Plant and Fungi Model Comparison (Genic F1)

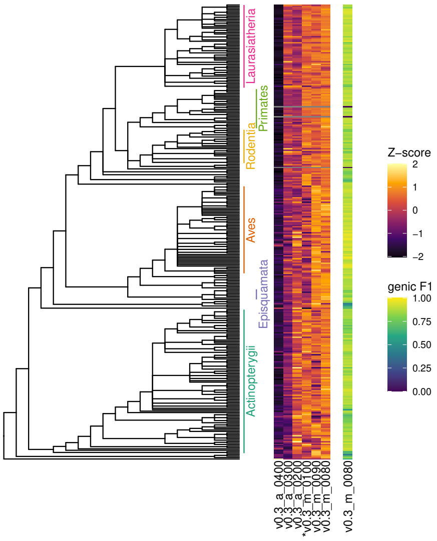

Figure S1: Illustrative performance of candidate and best vertebrate models across vertebrates. The absolute performance is measured with genic F1 on a random selection of 800 subsequences of each species and displayed for the overal best v0.3_m_0080 model (right). For peceptibility, differences between the genic F1 of models are displayed as a Z-score (middle). Models marked with * used the same training species as in Stiehler et al. [2020]. Grey indicates data is not available. Supplemental Figure S5 provides a finer grained view with species names included. Further details about the genomes, training sets and training parameters can be found in Supplementary tables S19, S24 and S30, respectively

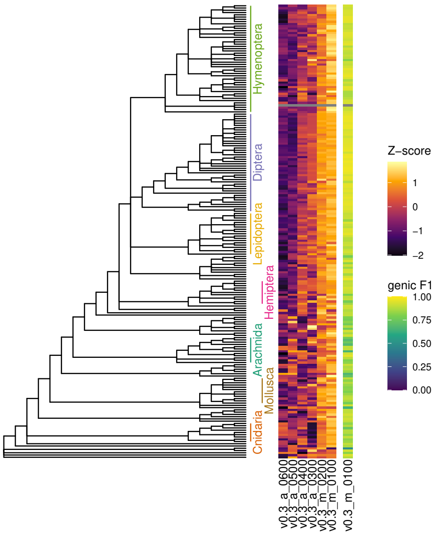

Figure S2: Illustrative performance of candidate and best invertebrate models across invertebrates. The absolute performance is measured with genic F1 on a random selection of 800 subsequences of each species and displayed for the overall best v0.3_m_0100 model (right). For perceptibility, differences between the genic F1 of models are displayed as a Z-score (middle). Supplemental Figure S6 provides a finer grained view with species names included. Further details about the species included, training sets and training parameters can be found in Supplementary tables S20, S25 and S31 respectively

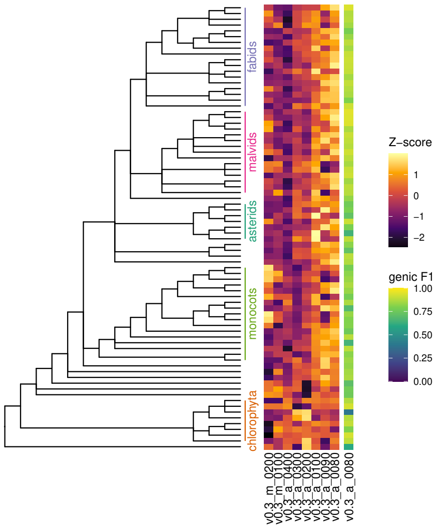

Figure S3: Illustrative performance of candidate and best plant models across plants. The absolute performance is measured with genic F1 on a random selection of 800 subsequences of each species and displayed for the overall best v0.3_a_0080 model (right). For perceptibility, differences between the genic F1 of models are displayed as a Z-score (middle). Models marked with * used the same training species as in Stiehler et al. (2020). Supplemental Figure S7 provides a finer grained view with species names included. Further details about the species included, training sets and training parameters can be found in Supplementary tables S18, S23 and S29 respectively

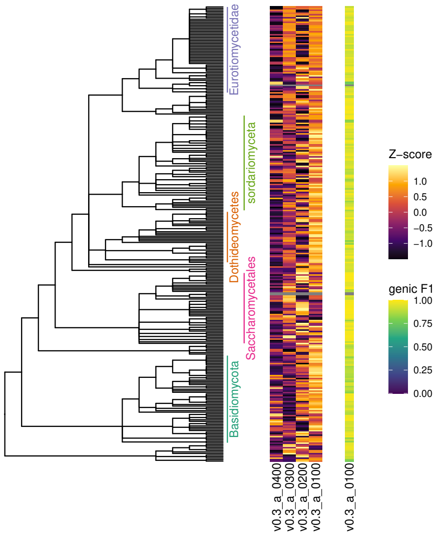

Figure S4: Illustrative performance of candidate and best fungus models across fungi. The absolute performance is measured with genic F1 on a random selection of 800 subsequences of each species and displayed for the overall best v0.3_a_0100 model (right). For perceptibility, differences between the genic F1 of models are displayed as a Z-score (middle). Supplemental Figure S8 provides a finer grained view with species names included. Further details about the species, training sets and training parameters can be found in Supplementary tables S17, S22 and S28 respectively

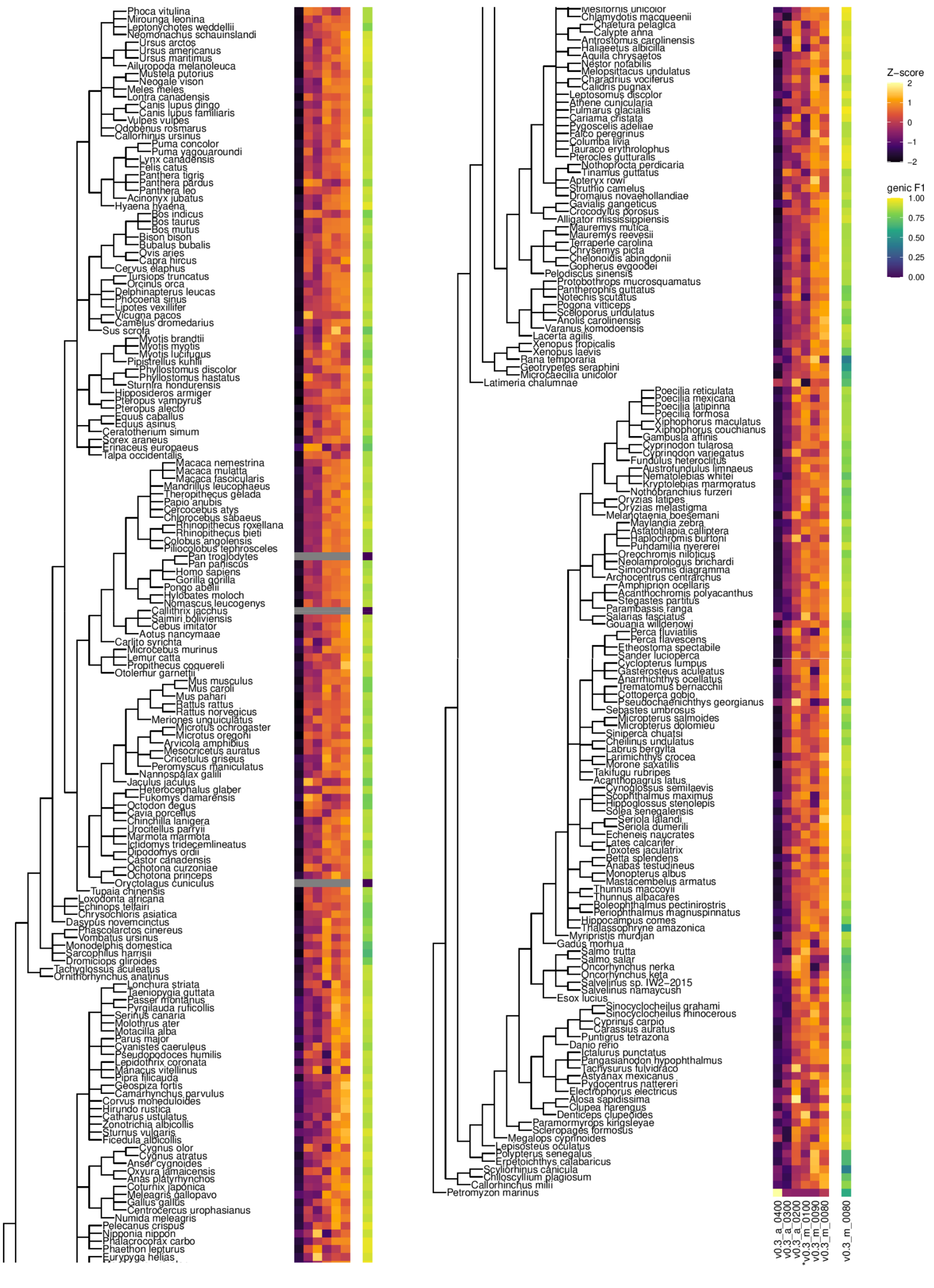

Figure S5: Illustrative performance of candidate and best vertebrate models across vertebrates. The absolute performance is measured with genic F1 on a random selection of 800 subsequences of each species and displayed for the overall best v0.3_m_0080 model (right). For perceptibility, differences between the genic F1 of models are displayed as a Z-score (middle). Models marked with * used the same training species as in Stiehler et al. (2020).

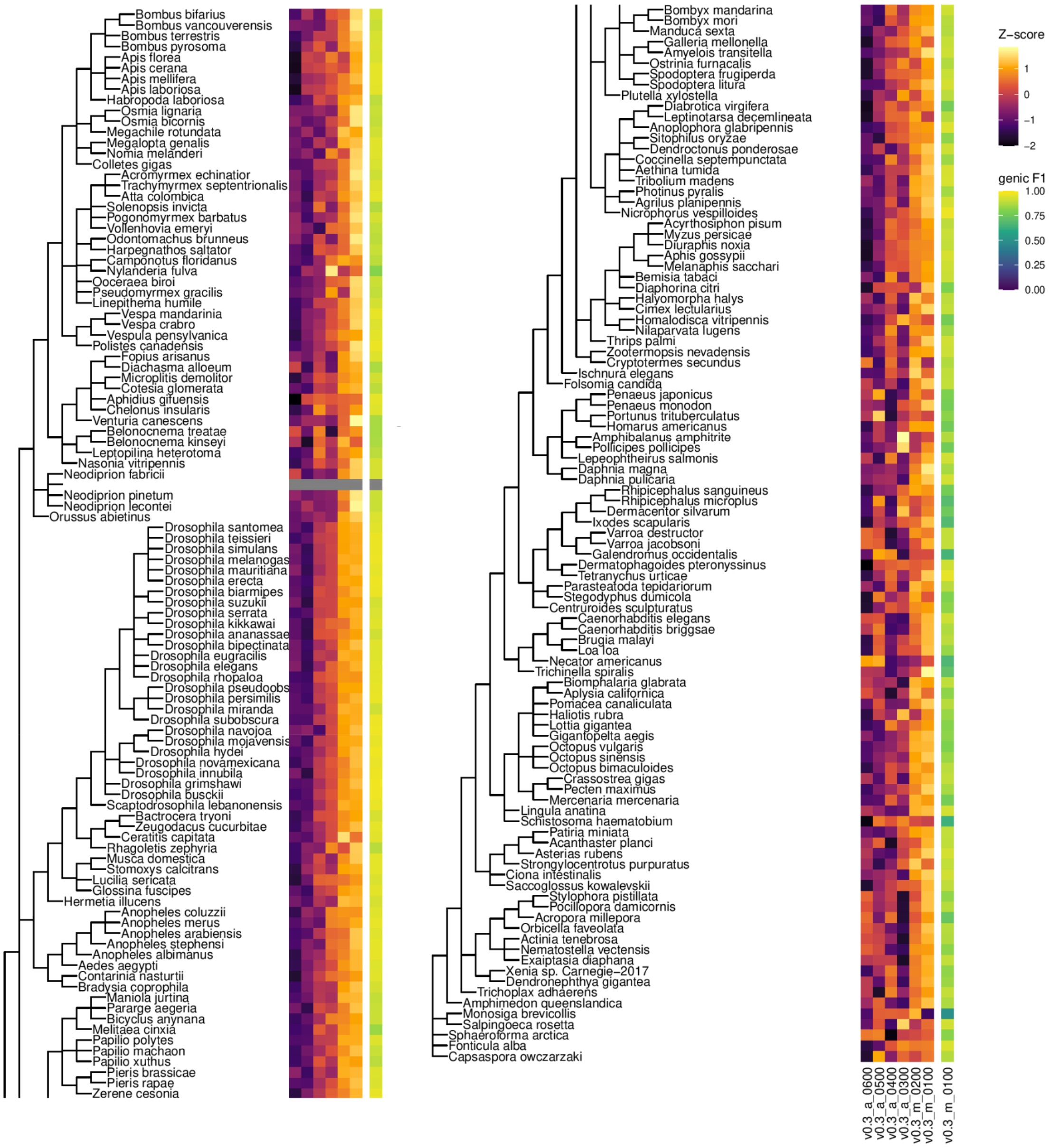

Figure S6: Performance of candidate and best invertebrate models across invertebrates. The absolute performance is measured with genic F1 on a random selection of 800 subsequences of each species and displayed for the overall best v0.3_m_0100 model (right). For perceptibility, differences between the genic F1 of models are displayed as a Z-score (middle).

S2.2 Plant Performance With Species Labels

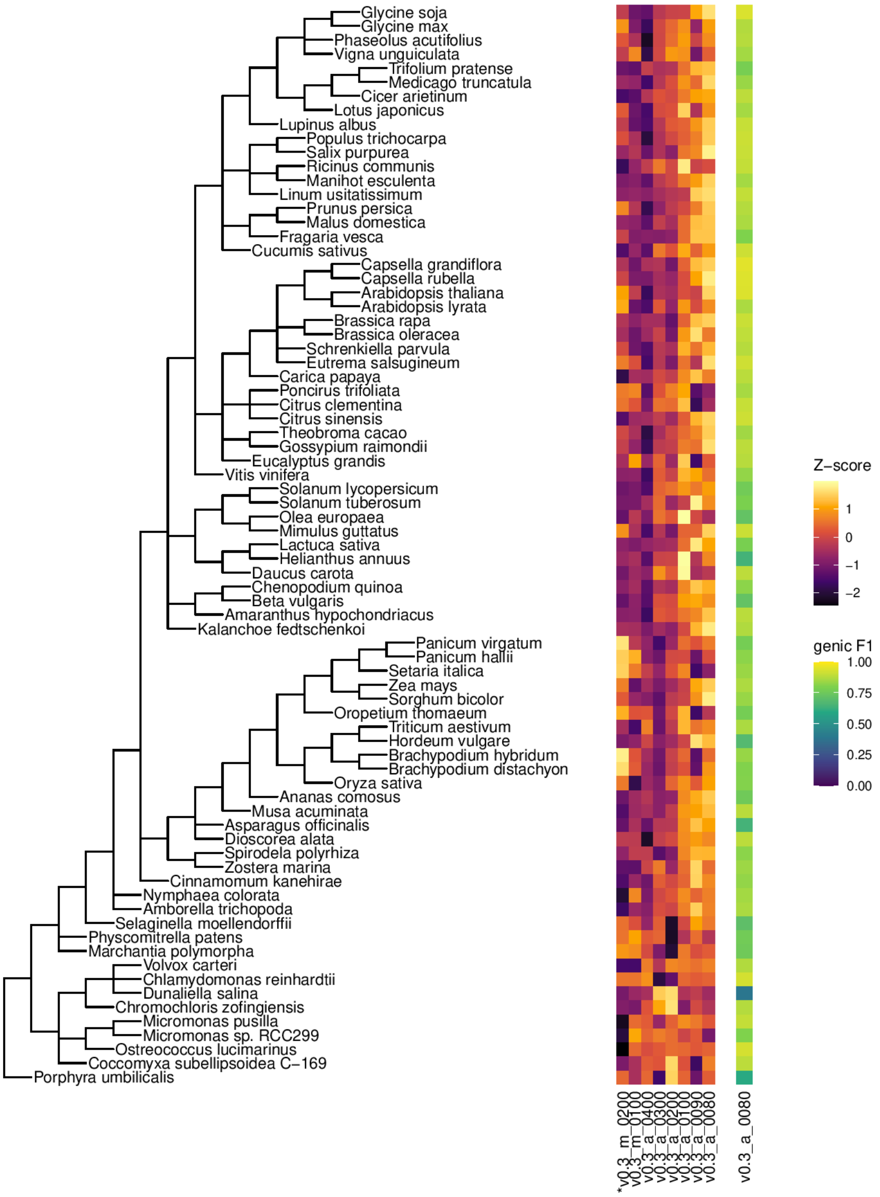

Figure S7: Performance of candidate and best plant models across plants. The absolute performance is measured with genic F1 on a random selection of 800 subsequences of each species and displayed for the overall best v0.3_m_0080 model (right). For perceptibility, differences between the genic F1 of models are displayed as a Z-score (middle). Models marked with * used the same training species as in Stiehler et al. (2020).

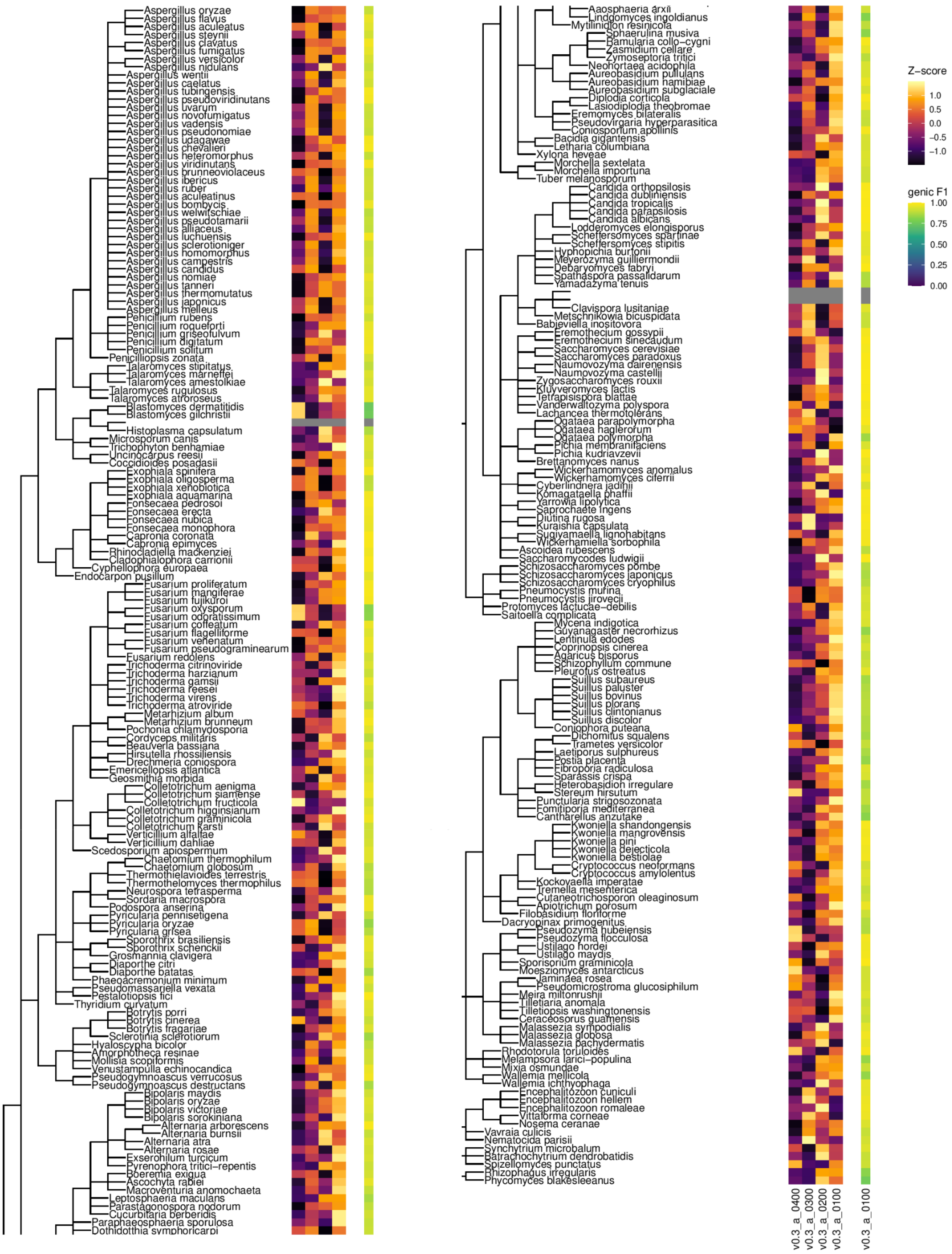

Figure S8: Performance of candidate and best fungus models across fungi. The absolute performance is measured with genic F1 on a random selection of 800 subsequences of each species and displayed for the overall best v0.3_a_0100 model (right). For perceptibility, differences between the genic F1 of models are displayed as a Z-score (middle).

S2.3 Relative Performance of Annotations measured with F1

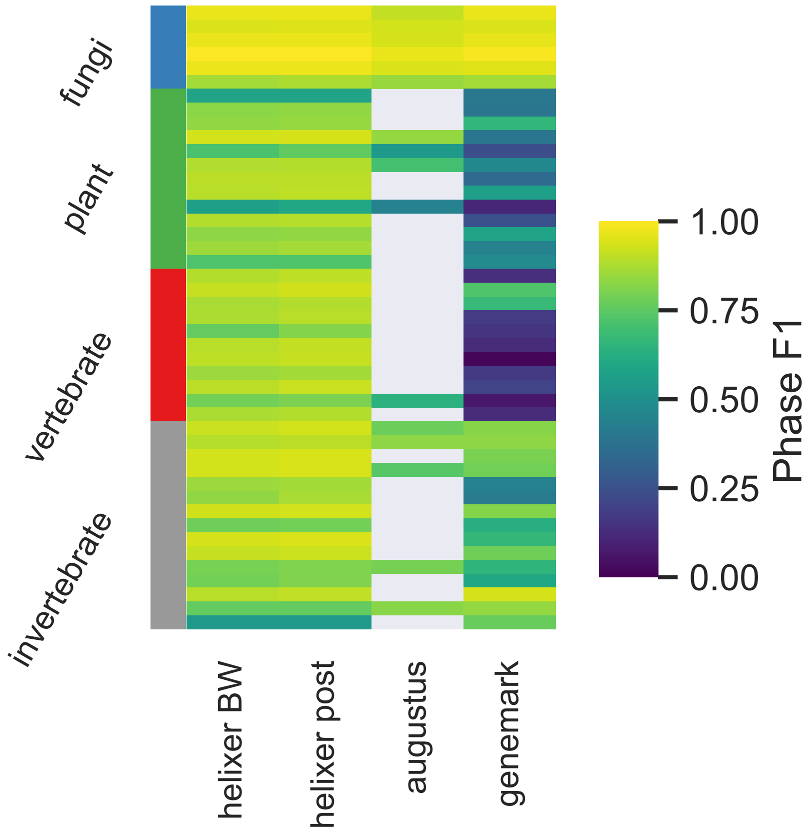

Figure S9: Test set annotation quality measured as Phase F1 compared to the reference. The labels 'helixer' and 'helixer post' indicate raw predictions from the neural network, and those post-processed by HelixerPost, respectively. Grey indicates data is not available.

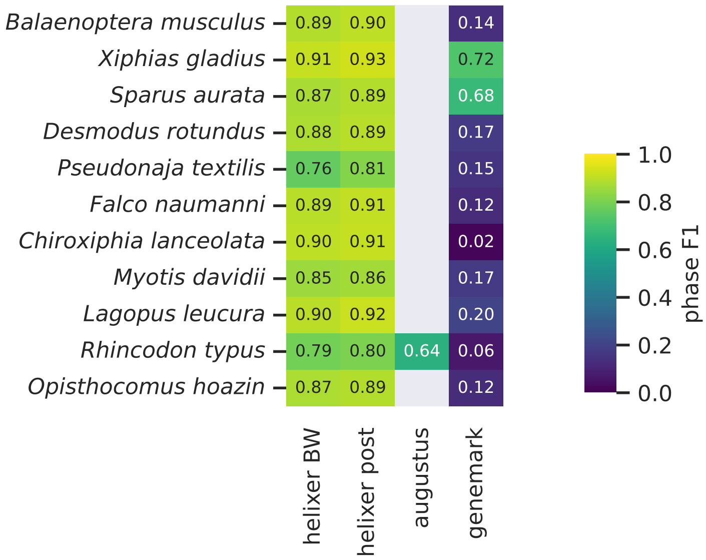

Figure S10: Test set annotation quality for vertebrates measured as Phase F1 compared to the reference. Grey indicates data is not available.

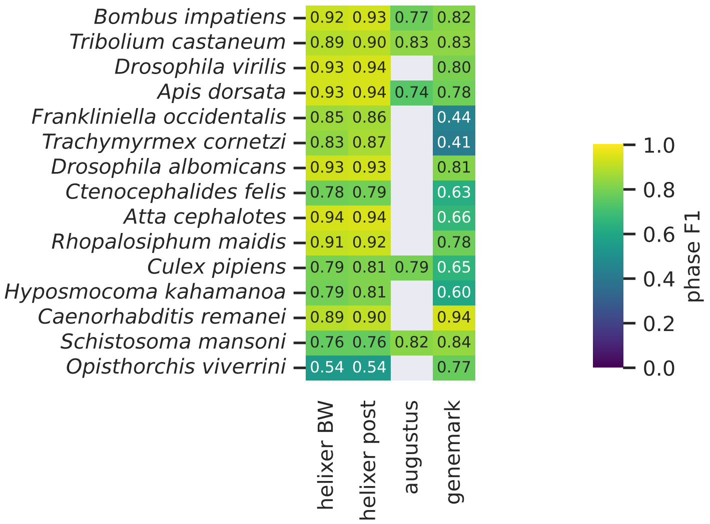

Figure S11: Test set annotation quality for invertebrates measured as Phase F1 compared to the reference. Grey indicates data is not available.

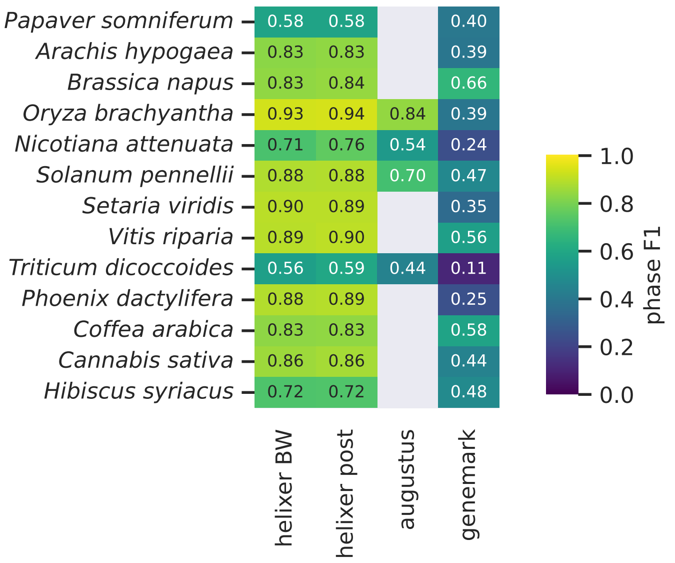

Figure S12: Test set annotation quality for plants measured as Phase F1 compared to the reference. Grey indicates data is not available.

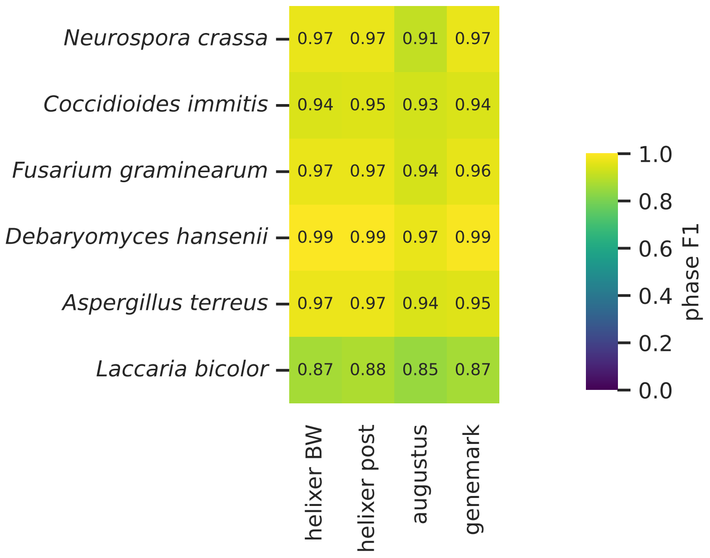

Figure S13: Test set annotation quality for fungi measured as Phase F1 compared to the reference

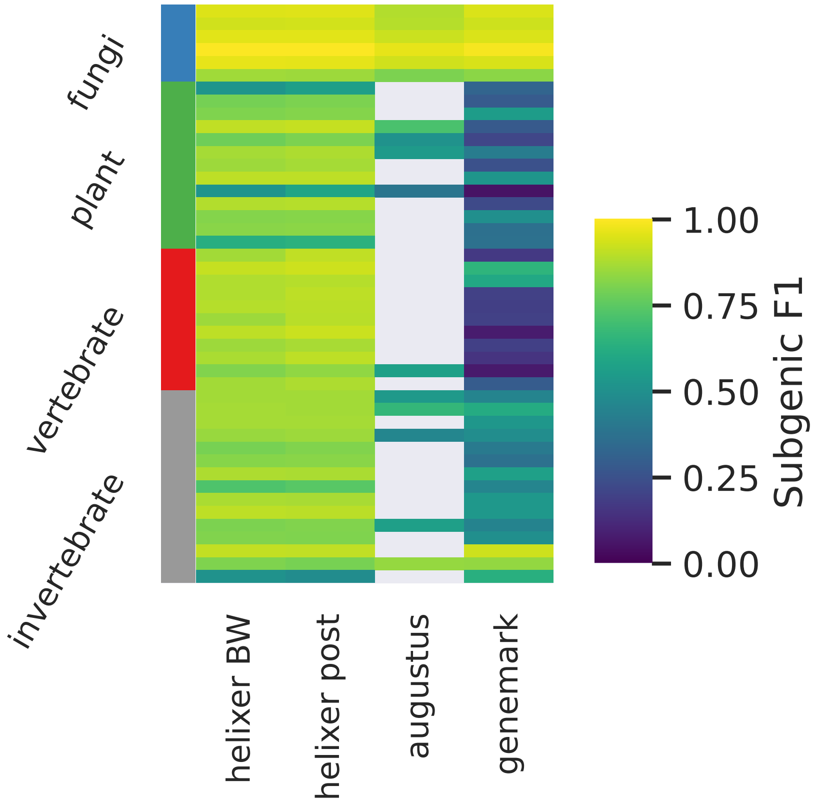

Figure S14: Test set annotation quality measured with Subgenic F1. Grey indicates data is not available.

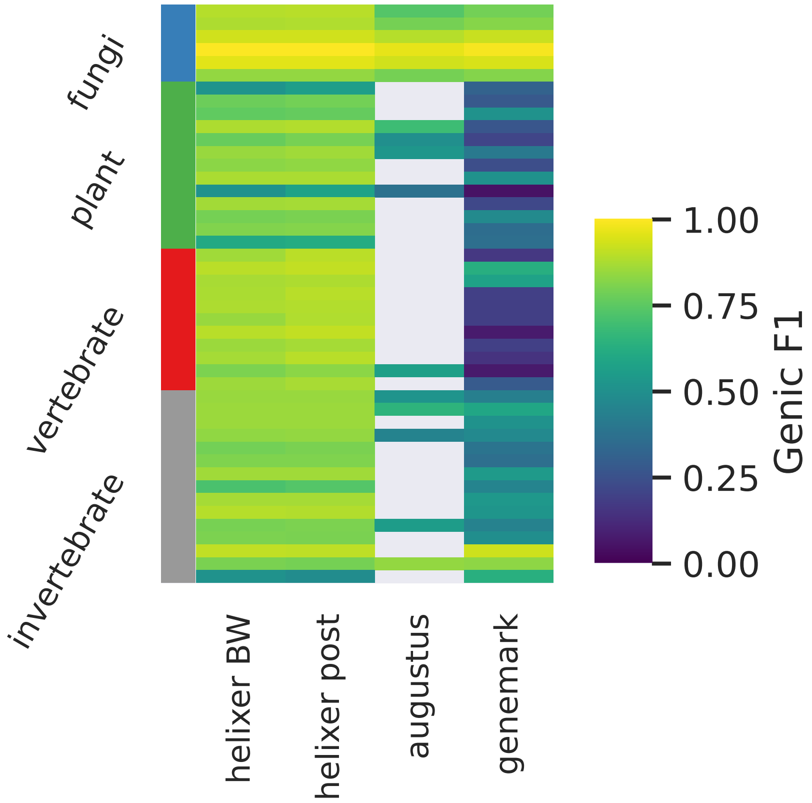

Figure S15: Test set annotation quality measured with Genic F1. Grey indicates data is not available.

S2.4 Intron precision and recall

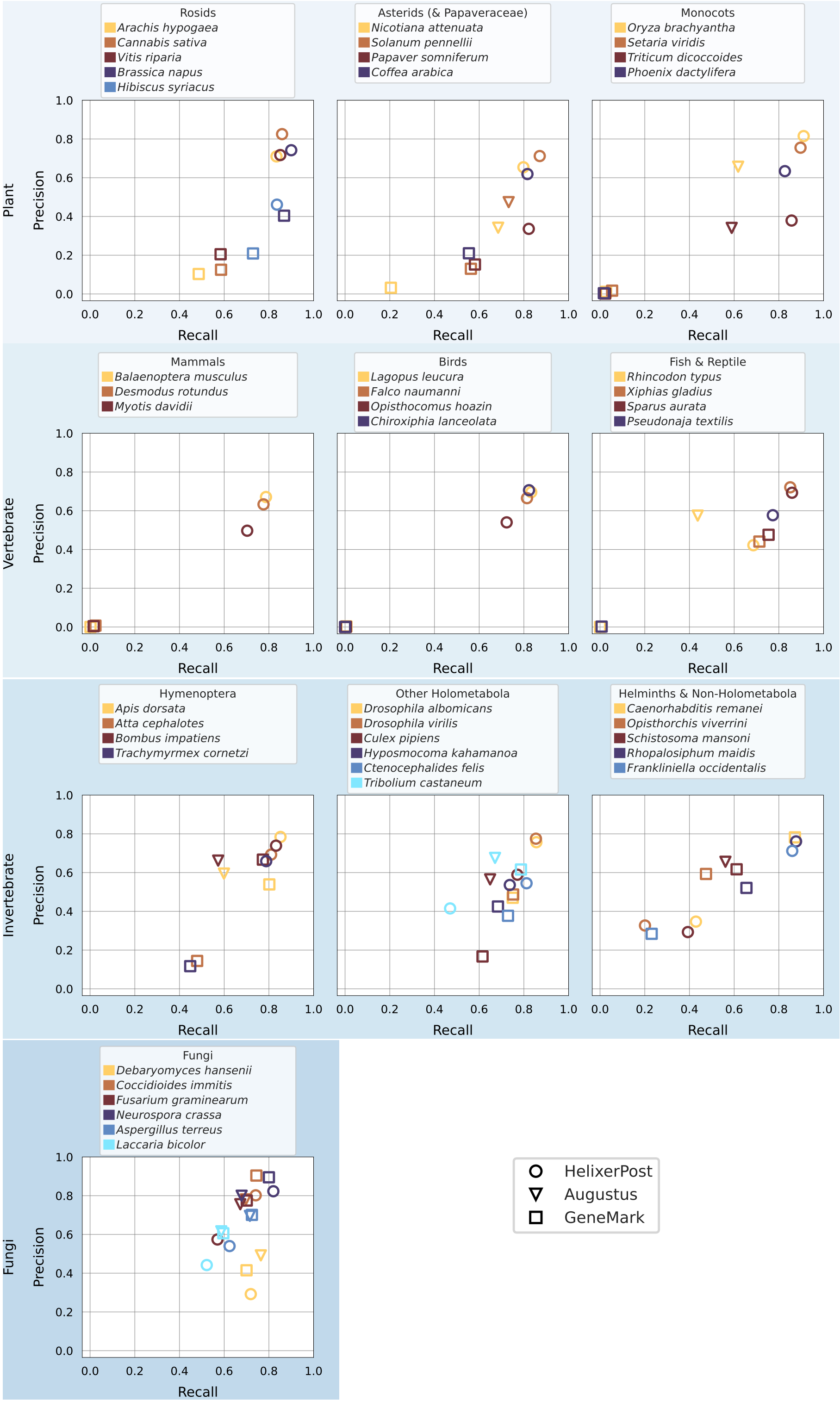

Figure S16: Intron precision and recall comparison between HelixerPost (circle), Augustus (triangle) and GeneMark (square). The first row shows plants, the second vertebrates, the third invertebrates and the fourth fungi.

S2.5 Comparison to Augustus using softmasking

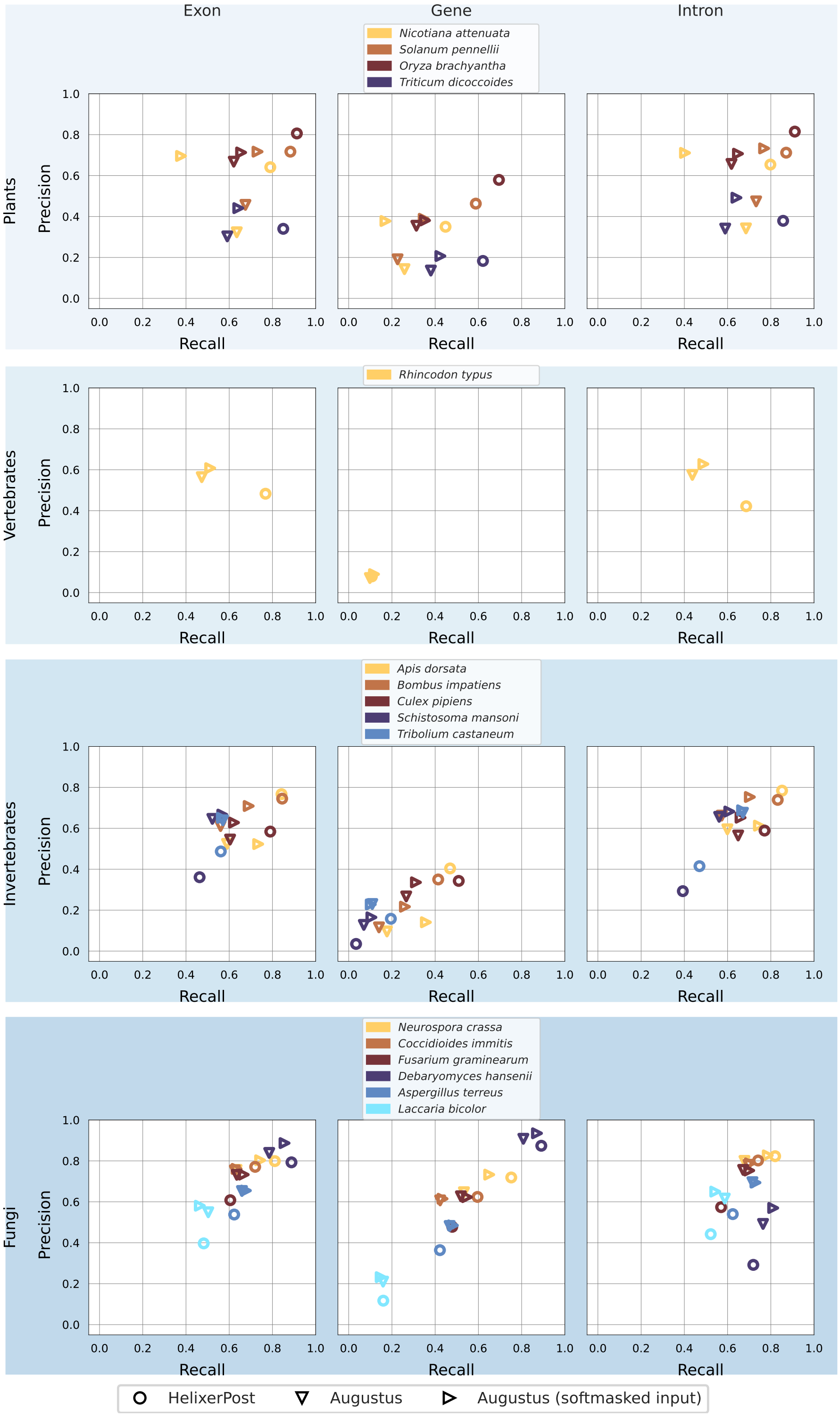

Figure S17: Precision and recall comparison between HelixerPost (circle), Augustus (triangle pointing down) and Augustus with softmasked genomes as input (triangle pointing right). The first column depicts exon, the second gene and the third intron metrics.

S2.6 Relative Performance of Annotations measured with BUSCO

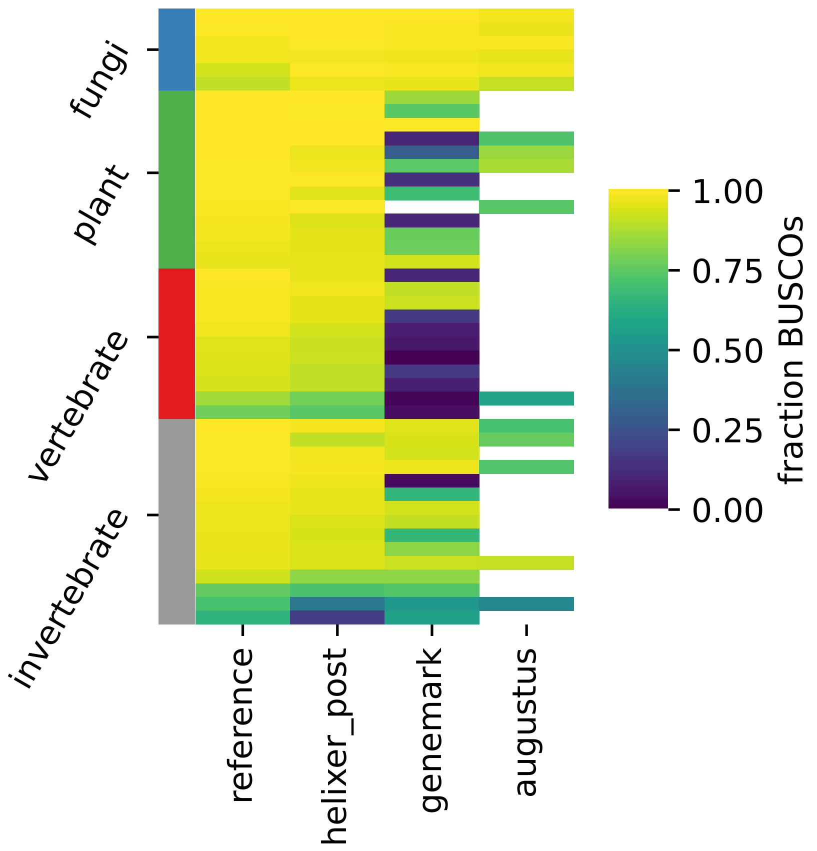

Figure S18: Test set annotation quality measured as a fraction of complete BUSCOs found in the final annotation. The labels 'helixer' and 'helixer post' indicate raw predictions from the neural network, and those post-processed by HelixerPost, respectively. Grey indicates data is not available.

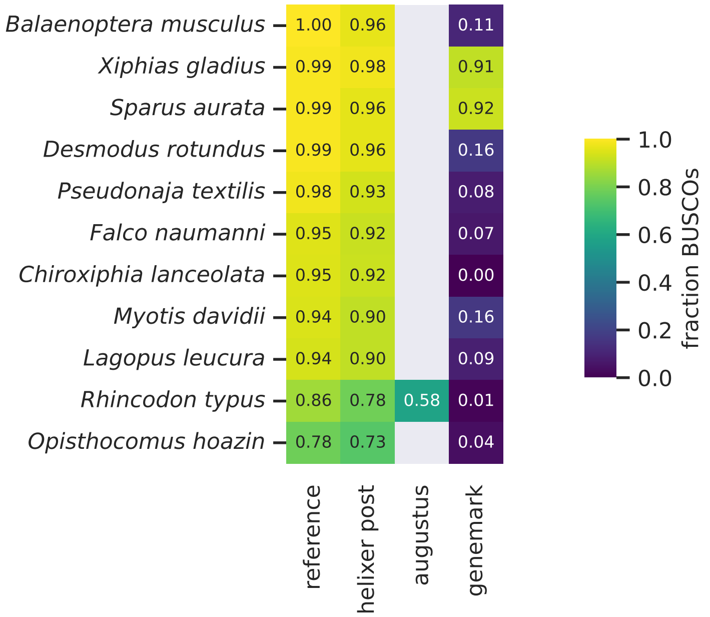

Figure S19: Test set annotation quality for vertebrates measured as fraction of complete BUSCOs found in final annotation. Grey indicates data is not available.

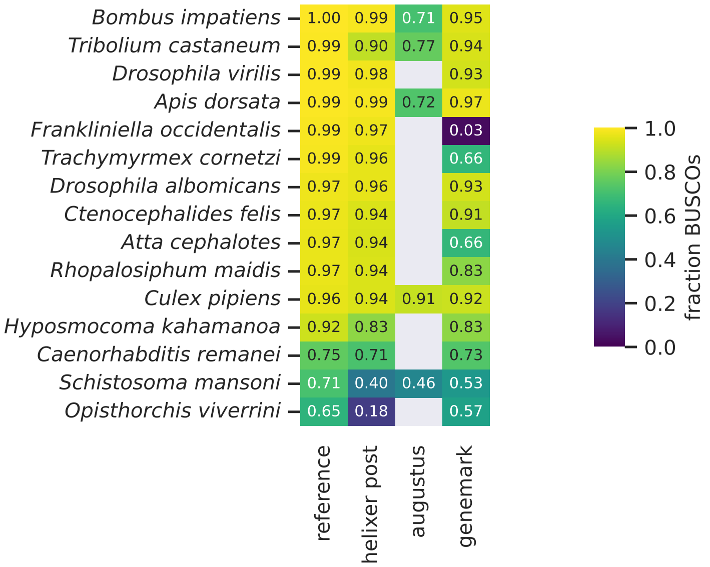

Figure S20: Test set annotation quality for invertebrates measured as fraction of complete BUSCOs found in final annotation. Grey indicates data is not available.

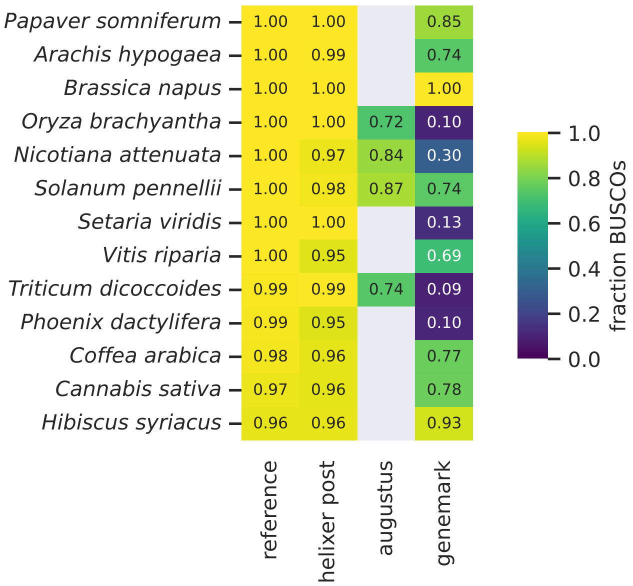

Figure S21: Test set annotation quality for plants measured as fraction of complete BUSCOs found in final annotation. Grey indicates data is not available.

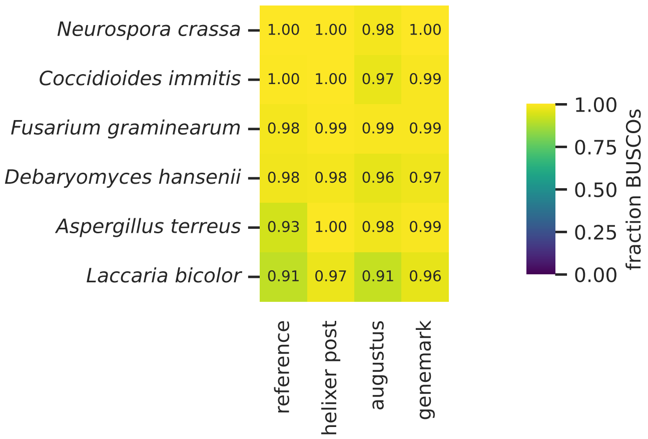

Figure S22: Test set annotation quality for fungi measured as fraction of complete BUSCOs found in final annotation.

S2.7 Ablations

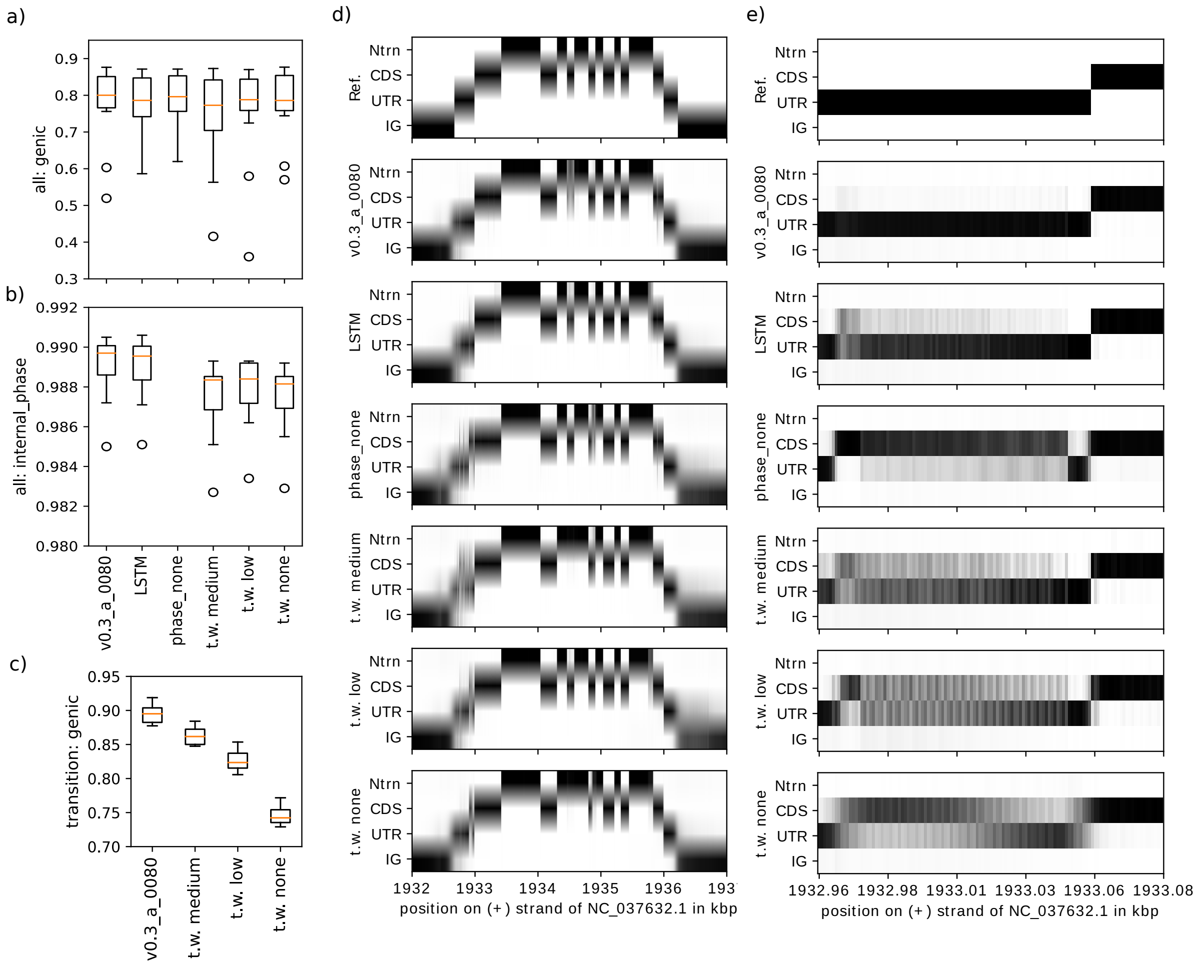

Figure S23: Performance of models with or without various optimizations to the models (phase, transition weights, Hybrid vs LSTM) on the 12 test plant species (*Triticum dicoccoides* was excluded). v0.3_a_0080 is the final model and contains all optimizations; LSTM has phase and the final transition weighting, but uses the pure LSTM architecture used in Stiehler et. al 2020; the phase_none model was trained without the phase matrix, the t.w. medium, low, and none models use progressively lower transition weights compared to v0.3_a_0080 (see methods). a) genic F1 for all positions, b) accuracy of internal phase predictions, i.e. accuracy as fraction of phase prediction when both the reference and the predictions had a non-none phase. This captures only frame shift errors. c) effect of transition weights on the genic F1 score *only* at the base pairs before or after a transition. d) example prediction of the genic class matrix compared to the reference 1-hot encoding of *Arachis hypogaea*, white=0, black=1 with grays being intermediate values. e) a zoom in of specifically the same gene, specifically around the start codon.

S2.8 Orthogroup based quality control

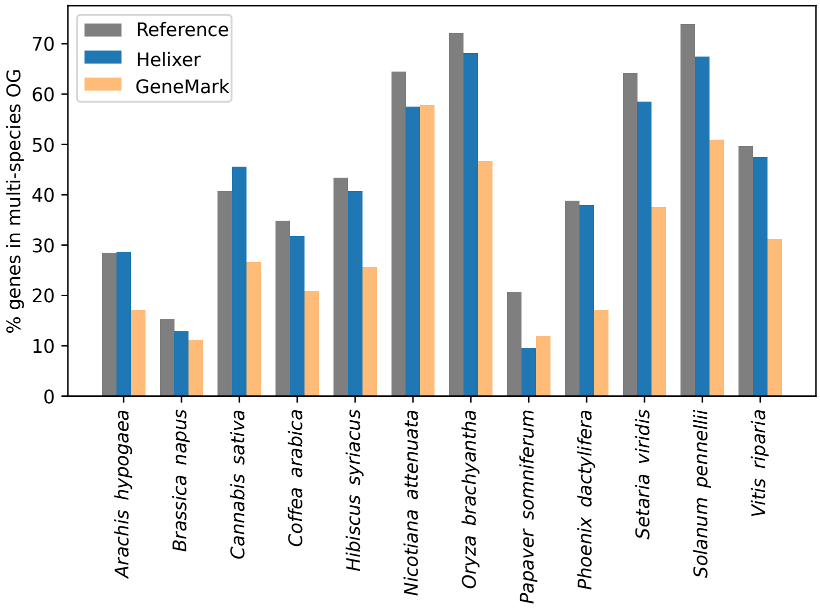

Figure S24: Precision proxy of proteome annotations as estimated with orthogroups. Specifically, the percentage of genes from each species that were assigned to an orthogroup containing at least one other species.Here with all 13 plant test species, but without GeneMarkES (missing annotation for *T. dicoccoides*).

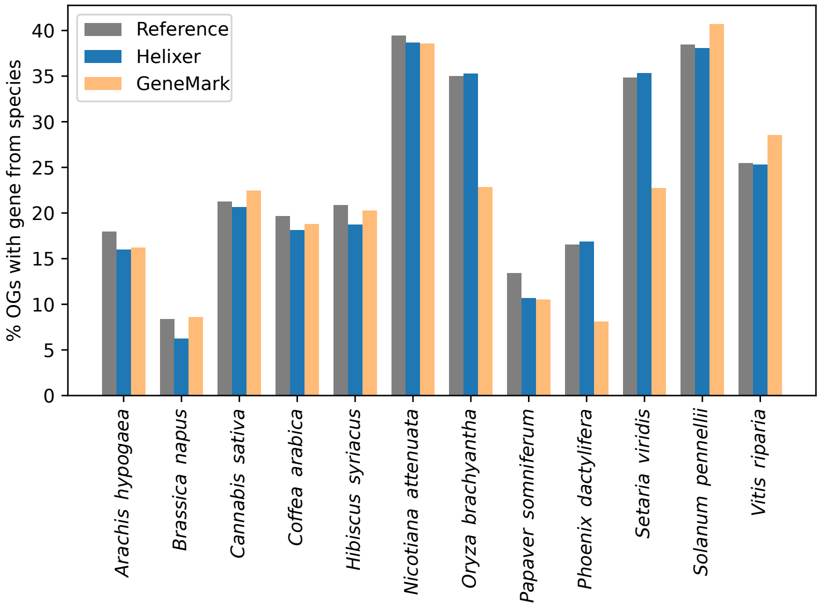

Figure S25: Recall proxy of proteome annotations as estimated with orthogroups. Specifically the percentage of orthogroups that contain a gene from each given species. Here with all 13 plant test species, but without GeneMarkES (missing annotation for *T. dicoccoides*).

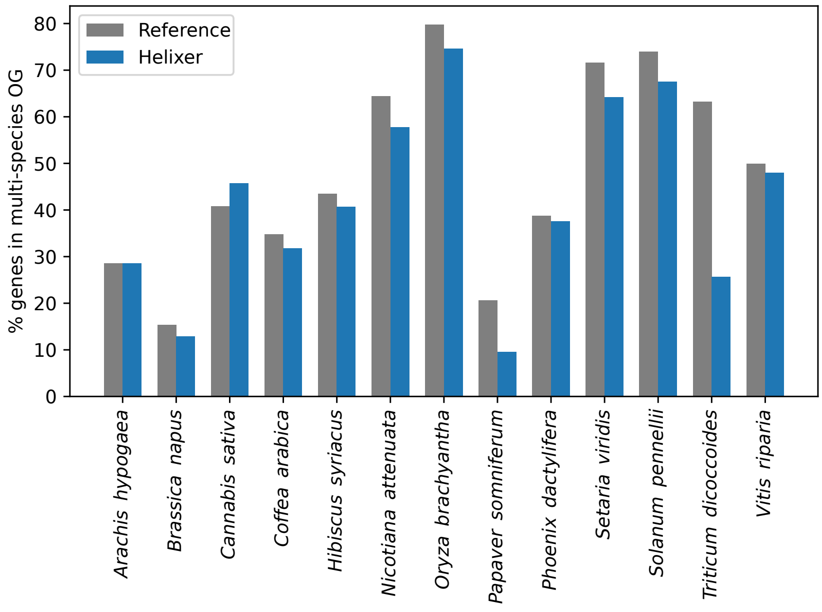

Figure S26: Precision proxy of proteome annotations as estimated with orthogroups. Specifically, the percentage of genes from each species that were assigned to an orthogroup containing at least one other species.

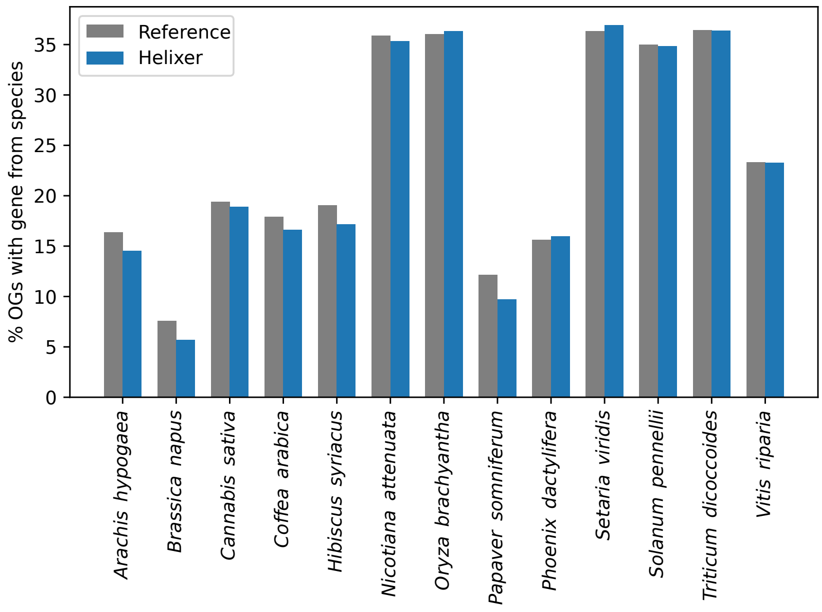

Figure S27: Recall proxy of proteome annotations as estimated with orthogroups. Specifically the percentage of orthogroups that contain a gene from each given species.

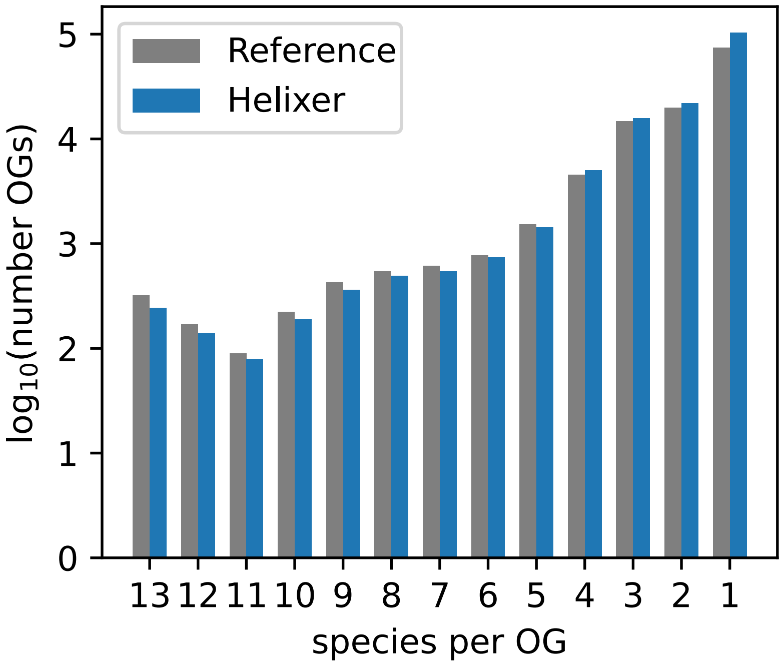

Figure S28: Orthogroup occupancy (number of species represented out of 13) for orthogroups based on the reference and Helixer's annotations (each clustered individually)

S2.9 *Arabidopsis thaliana*

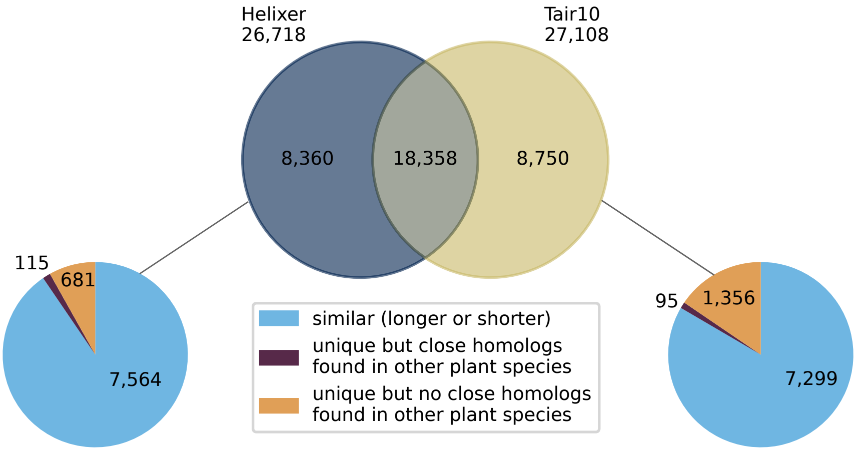

Figure S29: Comparison of the *A. thaliana* proteome predicted by Helixer to the existing TAIR10 annotation. Venn diagram and pie charts of the overlaps and differences between predicted proteomes. The pie charts categorize the unique sections from the Venn diagram further into three categories: 'similar (longer or shorter)', 'unique with homologs in other plant species' and 'unique without homologs in other plant species'. The second category encompasses likely legitimate annotations not found in the other proteome. The third category encompasses likely artefacts not found in the other proteome.

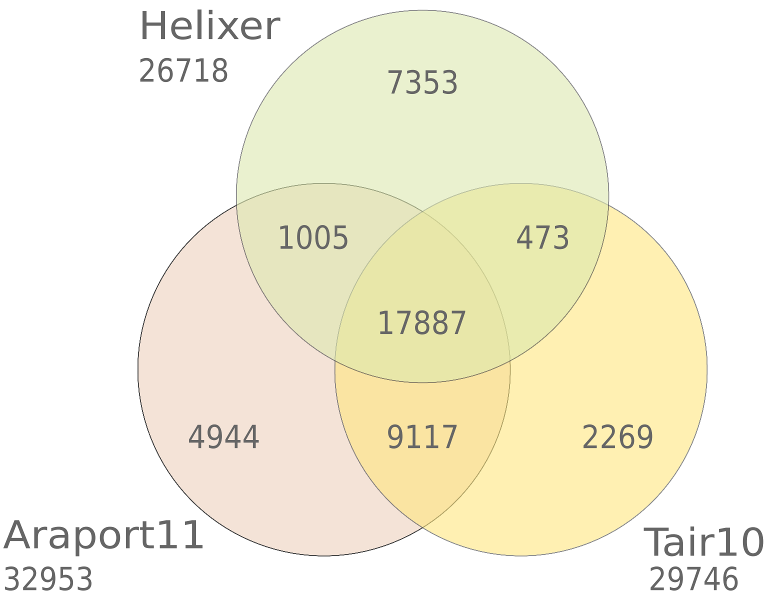

Figure S30: Comparison of the *A. thaliana* proteome predicted by Helixer to the existing TAIR10 and Araport11 annotations. Venn diagram of *identical* proteins. Note that here all splice variants, not loci, are counted.

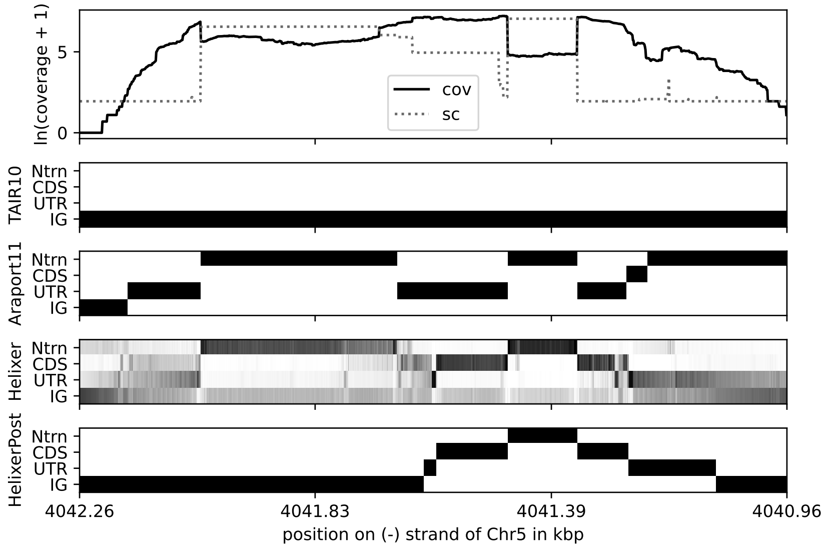

Figure S31: Comparison of the *A. thaliana* proteome predicted by Helixer to the existing TAIR10 and Araport11 annotations. Visualization of RNA-seq expression (cov=coverage; sc=spliced coverage) and available annotations zoomed into the genomic locus of the Phosphatidylinositol N-acetylglucosaminyltransferase γ subunit (Chr5, - strand, from 4041091 to 4041627, via HelixerPost).

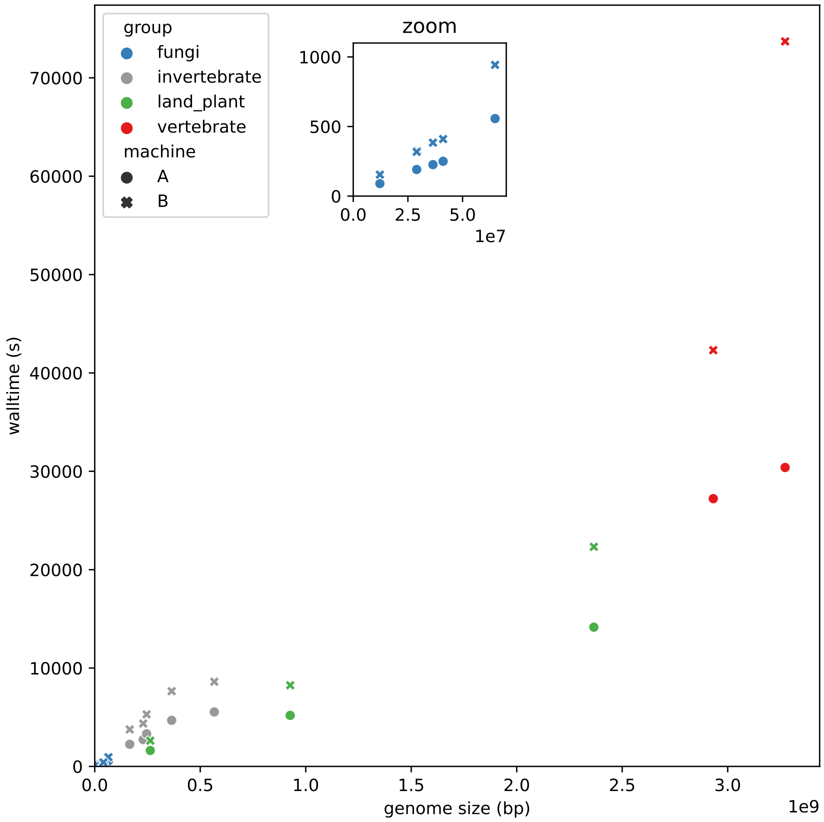

Figure S32: Benchmarking. Helixer walltime on some test genomes and *H. sapiens*. Comparison between two different workstations (see methods).

S3 Supplemental Tables

S3.1 Tabular Results

Table S1: Genic F1 for 800 randomly selected subsequences of each training and validation fungus species and for each model released during development. These results should be considered as illustrative only since the genomes were used as part of the training process.

	v0.3_a_0400	v0.3_a_0300	v0.3_a_0200	v0.3_a_0100
Candida auris	0.9911	0.9927	0.9915	0.9922
Candida haemuloni	0.9646	0.9671	0.9681	0.9679
Aaosphaeria arxii	0.9150	0.9171	0.9134	0.9180
Agaricus bisporus	0.8932	0.8916	0.8964	0.8995
Alternaria arborescens	0.9857	0.9869	0.9883	0.9882
Alternaria atra	0.9341	0.9353	0.9406	0.9382
Alternaria burnsii	0.9020	0.9039	0.9110	0.9070
Alternaria rosae	0.9254	0.9264	0.9225	0.9289
Amorphotheca resinae	0.9186	0.9222	0.9188	0.9243
Apiotrichum porosum	0.9659	0.9653	0.9664	0.9681
Ascochyta rabiei	0.9489	0.9555	0.9524	0.9584
Ascoidea rubescens	0.8745	0.8825	0.8844	0.8922
Aspergillus aculeatinus	0.9177	0.9179	0.9138	0.9179
Aspergillus aculeatus	0.9499	0.9506	0.9453	0.9491
Aspergillus alliaceus	0.8912	0.8990	0.8900	0.9005
Aspergillus bombycis	0.9672	0.9698	0.9697	0.9698
Aspergillus brunneoviolaceus	0.9153	0.9160	0.9100	0.9158
Aspergillus caelatus	0.9169	0.9229	0.9148	0.9249
Aspergillus campestris	0.9278	0.9302	0.9262	0.9305
Aspergillus candidus	0.9237	0.9261	0.9175	0.9238
Aspergillus chevalieri	0.9828	0.9843	0.9849	0.9853
Aspergillus clavatus	0.9780	0.9800	0.9799	0.9802
Aspergillus flavus	0.9603	0.9631	0.9635	0.9639
Aspergillus fumigatus	0.9592	0.9610	0.9609	0.9604
Aspergillus heteromorphus	0.8979	0.8999	0.8941	0.9011
Aspergillus homomorphus	0.9157	0.9181	0.9151	0.9189
Aspergillus ibericus	0.9181	0.9220	0.9145	0.9217
Aspergillus japonicus	0.9315	0.9335	0.9281	0.9335
Aspergillus luchuensis	0.9773	0.9803	0.9807	0.9816
Aspergillus melleus	0.9547	0.9565	0.9521	0.9556
Aspergillus nidulans	0.9046	0.9058	0.9103	0.9072
Aspergillus nomiae	0.9613	0.9644	0.9654	0.9660
Aspergillus novofumigatus	0.9055	0.9094	0.9032	0.9099
Aspergillus oryzae	0.9032	0.9054	0.9051	0.9052
Aspergillus pseudonomiae	0.9028	0.9097	0.9000	0.9088
Aspergillus pseudotamarii	0.9025	0.9063	0.8980	0.9061
Aspergillus pseudoviridinutans	0.9793	0.9809	0.9804	0.9807
Aspergillus ruber	0.9166	0.9235	0.9172	0.9236
Aspergillus sclerotioniger	0.9091	0.9128	0.9073	0.9127
Aspergillus steynii	0.9214	0.9247	0.9179	0.9248
Aspergillus tanneri	0.9381	0.9429	0.9448	0.9437
Aspergillus thermomutatus	0.9335	0.9387	0.9409	0.9397
Aspergillus tubingensis	0.9826	0.9839	0.9846	0.9843
Aspergillus udagawae	0.9803	0.9821	0.9829	0.9822
Aspergillus uvarum	0.9141	0.9160	0.9121	0.9161
Aspergillus vadensis	0.9166	0.9191	0.9137	0.9190
Aspergillus versicolor	0.9304	0.9333	0.9284	0.9336
Aspergillus viridinutans	0.9814	0.9832	0.9833	0.9836
Aspergillus welwitschiae	0.8828	0.8889	0.8811	0.8892
Aspergillus wentii	0.9339	0.9382	0.9310	0.9378
Aureobasidium namibiae	0.9598	0.9628	0.9642	0.9644
Aureobasidium pullulans	0.9330	0.9360	0.9323	0.9371
Aureobasidium subglaciale	0.9266	0.9292	0.9248	0.9310
Babjeviella inositovora	0.9062	0.9123	0.9024	0.9082
Bacidia gigantensis	0.9283	0.9314	0.9364	0.9327
Batrachochytrium dendrobatidis	0.9044	0.8959	0.9252	0.9088
Beauveria bassiana	0.9589	0.9600	0.9611	0.9607
Bipolaris maydis	0.9194	0.9211	0.9216	0.9238
Bipolaris oryzae	0.9414	0.9437	0.9460	0.9473
Bipolaris sorokiniana	0.9182	0.9212	0.9168	0.9253
Bipolaris victoriae	0.9327	0.9362	0.9388	0.9409
Blastomyces dermatitidis	0.7719	0.7222	0.7403	0.7521
Blastomyces gilchristii	0.7894	0.7320	0.7570	0.7650
Boeremia exigua	0.9251	0.9261	0.9201	0.9284
Botrytis cinerea	0.9099	0.9002	0.8955	0.9084
Botrytis fragariae	0.9267	0.9364	0.9401	0.9430
Botrytis porri	0.9333	0.9355	0.9429	0.9454
Brettanomyces nanus	0.9673	0.9682	0.9687	0.9678
Candida albicans	0.9932	0.9930	0.9948	0.9937
Candida dubliniensis	0.9949	0.9956	0.9954	0.9957
Candida orthopsilosis	0.9923	0.9922	0.9940	0.9921
Candida parapsilosis	0.9935	0.9944	0.9955	0.9945
Candida tropicalis	0.9921	0.9922	0.9930	0.9922
Cantharellus anzutake	0.7938	0.8016	0.8083	0.8195
Capronia coronata	0.9900	0.9910	0.9904	0.9907
Capronia epimyces	0.9892	0.9895	0.9890	0.9901
Ceraceosorus guamensis	0.9012	0.8982	0.8955	0.9068

	v0.3_a_0400	v0.3_a_0300	v0.3_a_0200	v0.3_a_0100
Chaetomium globosum	0.8735	0.8785	0.8691	0.8824
Chaetomium thermophilum	0.9448	0.9455	0.9458	0.9479
Cladophialophora carrionii	0.9895	0.9895	0.9888	0.9900
Clavispora lusitaniae	0.9497	0.9516	0.9479	0.9504
Coccidioides posadasii	0.9472	0.9441	0.9313	0.9489
Colletotrichum aenigma	0.9667	0.9671	0.9678	0.9677
Colletotrichum fructicola	0.9340	0.9283	0.9301	0.9299
Colletotrichum graminicola	0.9757	0.9766	0.9773	0.9768
Colletotrichum higginsianum	0.9537	0.9532	0.9537	0.9555
Colletotrichum karsti	0.9184	0.9176	0.9198	0.9187
Colletotrichum siamense	0.9736	0.9733	0.9736	0.9739
Coniophora puteana	0.8758	0.8725	0.8733	0.8757
Coniosporium apollinis	0.9795	0.9819	0.9820	0.9837
Coprinopsis cinerea	0.9335	0.9342	0.9360	0.9369
Cordyceps militaris	0.8893	0.8873	0.8917	0.8900
Cryptococcus amylolentus	0.9472	0.9460	0.9469	0.9479
Cryptococcus neoformans	0.9209	0.9181	0.9166	0.9214
Cucurbitaria berberidis	0.9201	0.9217	0.9211	0.9242
Cutaneotrichosporon oleaginosum	0.9192	0.9138	0.9173	0.9199
Cyberlindnera jadinii	0.9288	0.9364	0.9251	0.9333
Cyphellophora europaea	0.9904	0.9904	0.9901	0.9905
Dacryopinax primogenitus	0.8767	0.8760	0.8785	0.8799
Debaryomyces fabryi	0.9968	0.9976	0.9976	0.9972
Diaporthe batatas	0.8505	0.8516	0.8400	0.8529
Diaporthe citri	0.9238	0.9240	0.9251	0.9263
Dichomitus squalens	0.8547	0.8513	0.8540	0.8573
Diplodia corticola	0.9855	0.9858	0.9831	0.9867
Diutina rugosa	0.9922	0.9936	0.9913	0.9914
Dothidotthia symphoricarpi	0.9120	0.9124	0.9103	0.9123
Drechmeria coniospora	0.9208	0.9224	0.9400	0.9427
Emericellopsis atlantica	0.9282	0.9318	0.9240	0.9317
Encephalitozoon cuniculi	0.9503	0.9641	0.9562	0.9603
Encephalitozoon hellem	0.9729	0.9773	0.9746	0.9719
Encephalitozoon romaleae	0.9778	0.9780	0.9852	0.9754
Endocarpon pusillum	0.9421	0.9465	0.9523	0.9496
Eremomyces bilateralis	0.9047	0.9087	0.9059	0.9106
Eremothecium gossypii	0.9849	0.9855	0.9842	0.9835
Eremothecium sinecaudum	0.9931	0.9950	0.9955	0.9938
Exophiala aquamarina	0.9771	0.9799	0.9794	0.9801
Exophiala oligosperma	0.8967	0.8989	0.8843	0.8964
Exophiala spinifera	0.9828	0.9851	0.9842	0.9852
Exophiala xenobiotica	0.8985	0.9013	0.8840	0.8982
Exserohilum turcicum	0.9315	0.9330	0.9323	0.9352
Fibroporia radiculosa	0.9399	0.9411	0.9444	0.9442
Filobasidium floriforme	0.9132	0.9063	0.9162	0.9159
Fomitiporia mediterranea	0.8437	0.8404	0.8520	0.8499
Fonsecaea erecta	0.9728	0.9731	0.9751	0.9742
Fonsecaea monophora	0.9608	0.9633	0.9632	0.9638
Fonsecaea nubica	0.9688	0.9696	0.9701	0.9701
Fonsecaea pedrosoi	0.9842	0.9851	0.9852	0.9847
Fusarium coffeatum	0.9660	0.9677	0.9698	0.9690
Fusarium flagelliforme	0.9439	0.9438	0.9401	0.9448
Fusarium fujikuroi	0.9802	0.9814	0.9828	0.9830
Fusarium mangiferae	0.9857	0.9871	0.9884	0.9885
Fusarium odoratissimum	0.8341	0.8235	0.8111	0.8217
Fusarium oxysporum	0.8313	0.8186	0.8041	0.8177
Fusarium proliferatum	0.9822	0.9844	0.9841	0.9850
Fusarium pseudograminearum	0.9666	0.9690	0.9698	0.9696
Fusarium redolens	0.9204	0.9231	0.9180	0.9241
Fusarium venenatum	0.9613	0.9652	0.9647	0.9656
Geosmithia morbida	0.9419	0.9400	0.9446	0.9429
Grosmannia clavigera	0.9312	0.9309	0.9357	0.9354
Guyanagaster necrorhizus	0.8136	0.8226	0.8284	0.8327
Heterobasidion irregulare	0.8508	0.8442	0.8539	0.8561
Hirsutella rhossiliensis	0.9009	0.9068	0.9048	0.9142
Histoplasma capsulatum	0.8663	0.8636	0.8763	0.8703
Histoplasma mississippiense nom. inval.	0.8394	0.8374	0.8531	0.8486
Hyaloscypha bicolor	0.8903	0.8966	0.8929	0.8973
Hyphopichia burtonii	0.9430	0.9488	0.9469	0.9525
Jaminaea rosea	0.9283	0.9256	0.9185	0.9274
Kluyveromyces lactis	0.9896	0.9917	0.9922	0.9909
Kockovaella imperatae	0.9341	0.9332	0.9369	0.9378
Komagataella phaffii	0.9893	0.9898	0.9909	0.9891
Kuraishia capsulata	0.9852	0.9871	0.9853	0.9859
Kwoniella bestiolae	0.9776	0.9763	0.9799	0.9796
Kwoniella dejecticola	0.9724	0.9682	0.9790	0.9771
Kwoniella mangrovensis	0.9776	0.9753	0.9783	0.9788

	v0.3_a_0400	v0.3_a_0300	v0.3_a_0200	v0.3_a_0100
Kwoniella pini	0.9741	0.9757	0.9829	0.9844
Kwoniella shandongensis	0.9831	0.9817	0.9869	0.9860
Lachancea thermotolerans	0.9870	0.9885	0.9848	0.9860
Laetiporus sulphureus	0.8529	0.8496	0.8610	0.8639
Lasiodiplodia theobromae	0.9666	0.9672	0.9666	0.9673
Lentinula edodes	0.8948	0.8979	0.8980	0.9073
Leptosphaeria maculans	0.8527	0.8535	0.8616	0.8616
Letharia columbiana	0.9200	0.9267	0.9331	0.9323
Lindgomyces ingoldianus	0.8020	0.8094	0.8040	0.8089
Lodderomyces elongisporus	0.9848	0.9855	0.9858	0.9860
Macroventuria anomochaeta	0.8959	0.8993	0.8970	0.9022
Malassezia globosa	0.9760	0.9787	0.9793	0.9778
Malassezia pachydermatis	0.9640	0.9642	0.9664	0.9621
Malassezia sympodialis	0.9533	0.9546	0.9569	0.9544
Meira miltonrushii	0.9361	0.9397	0.9352	0.9463
Melampsora larici-populina	0.8023	0.7954	0.8419	0.8365
Metarhizium album	0.9709	0.9701	0.9692	0.9719
Metarhizium brunneum	0.9839	0.9847	0.9846	0.9851
Metschnikowia bicuspidata	0.9115	0.9170	0.9048	0.9137
Meyerozyma guilliermondii	0.9789	0.9814	0.9774	0.9791
Microsporum canis	0.9365	0.9382	0.9439	0.9413
Mixia osmundae	0.9031	0.8975	0.9129	0.9133
Moesziomyces antarcticus	0.9351	0.9200	0.9168	0.9269
Mollisia scopiformis	0.9205	0.9242	0.9192	0.9241
Morchella importuna	0.9137	0.9130	0.9242	0.9221
Morchella sextelata	0.9285	0.9289	0.9367	0.9375
Mycena indigotica	0.9288	0.9288	0.9356	0.9384
Mytilinidion resinicola	0.8889	0.8908	0.8853	0.8959
Naumovozyma castellii	0.9950	0.9950	0.9964	0.9954
Naumovozyma dairenensis	0.9924	0.9946	0.9960	0.9935
Nematocida parisii	0.9265	0.9598	0.9355	0.9279
Neohortaea acidophila	0.9324	0.9340	0.9293	0.9363
Neurospora tetrasperma	0.8753	0.8775	0.8764	0.8791
Nosema ceranae	0.8916	0.9433	0.9286	0.9417
Ogataea haglerorum	0.9585	0.9582	0.9580	0.9567
Ogataea parapolymorpha	0.9927	0.9931	0.9920	0.9910
Ogataea polymorpha	0.7584	0.8152	0.7564	0.8485
Paraphaeosphaeria sporulosa	0.9012	0.9025	0.8998	0.9066
Parastagonospora nodorum	0.8943	0.8972	0.8976	0.8983
Penicilliopsis zonata	0.9269	0.9292	0.9236	0.9294
Penicillium digitatum	0.9724	0.9741	0.9740	0.9735
Penicillium griseofulvum	0.9742	0.9749	0.9757	0.9748
Penicillium roqueforti	0.9591	0.9602	0.9621	0.9621
Penicillium rubens	0.9067	0.9102	0.9101	0.9107
Penicillium solitum	0.9741	0.9752	0.9754	0.9759
Pestalotiopsis fici	0.9894	0.9899	0.9901	0.9912
Phaeoacremonium minimum	0.9520	0.9526	0.9533	0.9532
Phycomyces blakesleeanus	0.7514	0.7472	0.7820	0.7706
Pichia kudriavzevii	0.9922	0.9921	0.9940	0.9924
Pichia membranifaciens	0.9313	0.9366	0.9252	0.9381
Pleurotus ostreatus	0.9527	0.9505	0.9576	0.9567
Pneumocystis jirovecii	0.9139	0.7579	0.9562	0.9349
Pneumocystis murina	0.9033	0.7167	0.9445	0.9276
Pochonia chlamydosporia	0.9475	0.9508	0.9510	0.9529
Podospora anserina	0.9702	0.9710	0.9697	0.9723
Postia placenta	0.8190	0.8243	0.8243	0.8340
Protomyces lactucae-debilis	0.9181	0.9249	0.9145	0.9268
Pseudogymnoascus destructans	0.8572	0.8621	0.8535	0.8632
Pseudogymnoascus verrucosus	0.9763	0.9774	0.9802	0.9791
Pseudomassariella vexata	0.9101	0.9154	0.9105	0.9157
Pseudomicrostroma glucosiphilum	0.9305	0.9274	0.9246	0.9301
Pseudovirgaria hyperparasitica	0.8994	0.9027	0.9005	0.9056
Pseudozyma flocculosa	0.9836	0.9805	0.9778	0.9804
Pseudozyma hubeiensis	0.8809	0.8675	0.8739	0.8744
Punctularia strigosozonata	0.8977	0.8969	0.8983	0.9013
Pyrenophora tritici-repentis	0.9103	0.9095	0.9160	0.9153
Pyricularia grisea	0.8845	0.8882	0.8699	0.8829
Pyricularia oryzae	0.8395	0.8470	0.8142	0.8358
Pyricularia pennisetigena	0.9019	0.9032	0.9047	0.9036
Ramularia collo-cygni	0.9844	0.9854	0.9867	0.9868
Rhinocladiella mackenziei	0.9719	0.9762	0.9750	0.9768
Rhizophagus irregularis	0.7646	0.7593	0.8215	0.8199
Rhodotorula toruloides	0.9843	0.9808	0.9790	0.9826
Saccharomyces cerevisiae	0.9828	0.9846	0.9865	0.9849
Saccharomyces paradoxus	0.9934	0.9946	0.9953	0.9940
Saccharomycodes ludwigii	0.9909	0.9909	0.9921	0.9912
Saitoella complicata	0.9415	0.9428	0.9403	0.9445

	v0.3_a_0400	v0.3_a_0300	v0.3_a_0200	v0.3_a_0100
Saprochaete ingens	0.9749	0.9762	0.9812	0.9825
Scedosporium apiospermum	0.9473	0.9481	0.9517	0.9496
Scheffersomyces spartinae	0.9558	0.9568	0.9581	0.9569
Scheffersomyces stipitis	0.9548	0.9567	0.9530	0.9571
Schizophyllum commune	0.9167	0.9162	0.9120	0.9169
Schizosaccharomyces cryophilus	0.8076	0.8184	0.8608	0.8836
Schizosaccharomyces japonicus	0.8106	0.8259	0.8278	0.8675
Schizosaccharomyces pombe	0.7928	0.8043	0.8534	0.8836
Sclerotinia sclerotiorum	0.8278	0.8324	0.8527	0.8465
Sordaria macrospora	0.9676	0.9691	0.9696	0.9696
Sparassis crispa	0.9135	0.9252	0.9304	0.9365
Spathaspora passalidarum	0.8297	0.8502	0.8340	0.8711
Sphaerulina musiva	0.8144	0.8190	0.8124	0.8237
Spizellomyces punctatus	0.9177	0.9141	0.9139	0.9171
Sporisorium graminicola	0.9939	0.9922	0.9936	0.9932
Sporothrix brasiliensis	0.9548	0.9563	0.9571	0.9567
Sporothrix schenckii	0.9575	0.9555	0.9566	0.9592
Stereum hirsutum	0.9003	0.8972	0.8972	0.8990
Sugiyamaella lignohabitans	0.9800	0.9791	0.9780	0.9807
Suillus bovinus	0.8703	0.8741	0.8779	0.8840
Suillus clintonianus	0.8747	0.8803	0.8794	0.8904
Suillus discolor	0.8602	0.8616	0.8689	0.8750
Suillus paluster	0.8488	0.8603	0.8632	0.8748
Suillus plorans	0.8811	0.8847	0.8875	0.8926
Suillus subaureus	0.8390	0.8427	0.8522	0.8546
Synchytrium microbalum	0.9468	0.9316	0.9554	0.9452
Talaromyces amestolkiae	0.9311	0.9318	0.9344	0.9318
Talaromyces atroroseus	0.9563	0.9572	0.9600	0.9588
Talaromyces marneffei	0.9355	0.9355	0.9378	0.9415
Talaromyces rugulosus	0.9533	0.9546	0.9557	0.9556
Talaromyces stipitatus	0.8878	0.8861	0.8971	0.8964
Tetrapisispora blattae	0.9954	0.9964	0.9966	0.9962
Thermothelomyces thermophilus	0.8813	0.8817	0.8727	0.8810
Thermothielavioides terrestris	0.8973	0.8978	0.8876	0.8989
Thyridium curvatum	0.9504	0.9497	0.9509	0.9517
Tilletiaria anomala	0.9057	0.9048	0.8997	0.9102
Tilletiopsis washingtonensis	0.9254	0.9259	0.9140	0.9290
Trametes versicolor	0.9067	0.9058	0.9005	0.9054
Tremella mesenterica	0.8030	0.8184	0.9058	0.9027
Trichoderma atroviride	0.8907	0.8896	0.8824	0.8913
Trichoderma citrinoviride	0.9395	0.9386	0.9397	0.9405
Trichoderma gamsii	0.9623	0.9612	0.9629	0.9628
Trichoderma harzianum	0.9102	0.9102	0.9093	0.9122
Trichoderma reesei	0.9341	0.9338	0.9335	0.9356
Trichoderma virens	0.9310	0.9304	0.9296	0.9328
Trichophyton benhamiae	0.9395	0.9392	0.9390	0.9423
Tuber melanosporum	0.9111	0.9096	0.9220	0.9189
Uncinocarpus reesii	0.9222	0.9253	0.9268	0.9262
Ustilago hordei	0.9664	0.9476	0.9732	0.9747
Ustilago maydis	0.9806	0.9794	0.9839	0.9821
Vanderwaltozyma polyspora	0.9875	0.9831	0.9882	0.9818
Vavraia culicis	0.8805	0.9084	0.8946	0.9073
Venustampulla echinocandica	0.9644	0.9681	0.9687	0.9686
Verticillium alfalfae	0.9217	0.9195	0.9157	0.9213
Verticillium dahliae	0.9279	0.9278	0.9255	0.9294
Vittaforma corneae	0.9730	0.9781	0.9756	0.9711
Wallemia ichthyophaga	0.9585	0.9572	0.9704	0.9624
Wallemia mellicola	0.8177	0.8251	0.8305	0.8334
Wickerhamiella sorbophila	0.9790	0.9811	0.9806	0.9812
Wickerhamomyces anomalus	0.9465	0.9484	0.9488	0.9530
Wickerhamomyces ciferrii	0.9841	0.9845	0.9858	0.9854
Xylona heveae	0.9386	0.9379	0.9321	0.9414
Yamadazyma tenuis	0.8518	0.8721	0.8461	0.8759
Yarrowia lipolytica	0.9801	0.9830	0.9844	0.9851
Zasmidium cellare	0.9551	0.9558	0.9572	0.9578
Zygosaccharomyces rouxii	0.9959	0.9960	0.9968	0.9958
Zymoseptoria tritici	0.8827	0.8806	0.8836	0.8879

Table S2: Genic F1 for 800 randomly selected subsequences of each training and validation plant species and for each model released during development. These results should be considered as illustrative only since the genomes were used as part of the training process.

	*v0.3_m_0200	v0.3_m_0100	v0.3_a_0400	v0.3_a_0300	v0.3_a_0200	v0.3_a_0100	v0.3_a_0090	v0.3_a_0080
Ananas comosus	0.6471	0.6726	0.6835	0.6617	0.6659	0.7338	0.7505	0.7639
Amaranthus hypochondriacus	0.8169	0.8183	0.7900	0.8520	0.8483	0.8628	0.8687	0.8895
Arabidopsis lyrata	0.8678	0.8507	0.8485	0.8612	0.8542	0.8663	0.8601	0.8653
Asparagus officinalis	0.5395	0.5775	0.5403	0.5844	0.5851	0.6402	0.6655	0.6497
Arabidopsis thaliana	0.9402	0.9332	0.9246	0.9318	0.9311	0.9349	0.9338	0.9430
Amborella trichopoda	0.6978	0.7701	0.7553	0.8238	0.8139	0.8625	0.9219	0.8652
Brachypodium distachyon	0.8192	0.7974	0.7820	0.7740	0.7902	0.7960	0.7723	0.8076
Brachypodium hybridum	0.8514	0.8344	0.8266	0.8207	0.8312	0.8366	0.8180	0.8369
Brassica oleracea	0.8827	0.8746	0.8816	0.8881	0.8758	0.9013	0.9104	0.8969
Brassica rapa	0.8905	0.8852	0.8887	0.8886	0.8797	0.8990	0.9109	0.9128
Beta vulgaris	0.6640	0.6777	0.6592	0.7054	0.7032	0.7316	0.7347	0.7264
Cicer arietinum	0.8167	0.8217	0.8510	0.8660	0.8619	0.8782	0.8902	0.8927
Citrus clementina	0.9143	0.9122	0.9044	0.9141	0.9137	0.9193	0.8999	0.9075
Capsella grandiflora	0.9358	0.9293	0.9355	0.9361	0.9297	0.9425	0.9509	0.9525
Cinnamomum kanehirae	0.6930	0.7425	0.7141	0.8017	0.7849	0.8066	0.8771	0.8308
Carica papaya	0.8397	0.8659	0.8649	0.8765	0.8721	0.8909	0.9022	0.8883
Chenopodium quinoa	0.7762	0.7827	0.7641	0.7863	0.7833	0.8122	0.8272	0.8221
Chlamydomonas reinhardtii	0.9331	0.9330	0.9402	0.8764	0.8914	0.9299	0.9272	0.9290
Capsella rubella	0.9266	0.9172	0.9170	0.9219	0.9201	0.9281	0.9378	0.9450
Cucumis sativus	0.8936	0.9012	0.8946	0.9139	0.9099	0.9194	0.9116	0.9177
Citrus sinensis	0.8812	0.8916	0.8923	0.8968	0.8918	0.9030	0.9148	0.9188
Coccomyxa subellipsoidea C-169	0.8701	0.8773	0.8872	0.8860	0.9044	0.8854	0.8611	0.8920
Chromochloris zofingiensis	0.8585	0.8546	0.9027	0.9387	0.9612	0.8570	0.8964	0.8802
Dioscorea alata	0.8789	0.8788	0.8571	0.8796	0.8820	0.8903	0.8923	0.8899
Daucus carota	0.8861	0.8904	0.8885	0.9036	0.8982	0.9131	0.8913	0.8933
Dunaliella salina	0.2830	0.3772	0.4168	0.8863	0.9277	0.4367	0.4853	0.3940
Eucalyptus grandis	0.8776	0.8915	0.8763	0.8855	0.8767	0.8951	0.8678	0.8844
Eutrema salsugineum	0.9154	0.9127	0.9033	0.9102	0.9065	0.9149	0.9101	0.9210
Fragaria vesca	0.7717	0.7535	0.7551	0.7542	0.7568	0.7838	0.8042	0.8042
Glycine max	0.8754	0.8504	0.8400	0.8635	0.8690	0.8750	0.8536	0.8769
Gossypium raimondii	0.8701	0.8632	0.8512	0.8690	0.8700	0.8792	0.8777	0.8912
Glycine soja	0.9140	0.9007	0.8992	0.9127	0.9155	0.9158	0.9318	0.9394
Helianthus annuus	0.6017	0.6071	0.5765	0.6209	0.6219	0.6897	0.6116	0.6488
Hordeum vulgare	0.5973	0.6052	0.6336	0.6023	0.6131	0.6338	0.6996	0.6858
Kalanchoe fedtschenkoi	0.8326	0.8321	0.8237	0.8314	0.8273	0.8421	0.8633	0.8727
Lupinus albus	0.8839	0.8690	0.8655	0.8821	0.8900	0.8914	0.8990	0.9068
Lotus japonicus	0.8504	0.8344	0.8300	0.8483	0.8493	0.8646	0.8429	0.8573
Lactuca sativa	0.7079	0.7008	0.7117	0.7178	0.7208	0.7645	0.8198	0.7910
Linum usitatissimum	0.8132	0.8164	0.8116	0.8235	0.8299	0.8340	0.8958	0.9014
Musa acuminata	0.8442	0.8572	0.8591	0.8539	0.8635	0.8814	0.8830	0.8872
Malus domestica	0.8261	0.8242	0.8140	0.8359	0.8299	0.8480	0.8650	0.8663
Manihot esculenta	0.7711	0.7754	0.7607	0.7950	0.8037	0.8251	0.8365	0.8552
Mimulus guttatus	0.9097	0.9003	0.8923	0.9010	0.8959	0.9062	0.9063	0.9145
Marchantia polymorpha	0.7940	0.7887	0.7523	0.7013	0.6485	0.7788	0.7553	0.7629
Micromonas pusilla	0.8537	0.9074	0.9043	0.8856	0.9054	0.9147	0.9104	0.9032
Micromonas sp. RCC299	0.7451	0.9411	0.8949	0.9107	0.9004	0.8676	0.8876	0.8044
Medicago truncatula	0.7990	0.7973	0.7815	0.8045	0.8129	0.8266	0.8184	0.8393
Nymphaea colorata	0.7423	0.8582	0.7865	0.8274	0.8359	0.8598	0.8428	0.8546
Olea europaea	0.7150	0.7297	0.7192	0.7418	0.7376	0.7638	0.7344	0.7261
Ostreococcus lucimarinus	0.5145	0.9390	0.8932	0.9139	0.9451	0.9377	0.9119	0.9294
Oryza sativa	0.8031	0.7682	0.7858	0.7814	0.7999	0.8069	0.7982	0.8095
Oropetium thomaeum	0.7896	0.7827	0.7829	0.7737	0.7815	0.7900	0.7703	0.7793
Phaseolus acutifolius	0.8772	0.8709	0.8526	0.8748	0.8797	0.8848	0.8742	0.8829
Panicum hallii	0.8380	0.8347	0.8117	0.8079	0.8215	0.8219	0.8076	0.8225
Physcomitrella patens	0.7972	0.8135	0.7885	0.7922	0.7120	0.7690	0.7953	0.7695
Prunus persica	0.8732	0.8631	0.8493	0.8593	0.8641	0.8667	0.8724	0.8799
Populus trichocarpa	0.8994	0.8922	0.8736	0.8928	0.8981	0.9018	0.9059	0.9159
Poncirus trifoliata	0.8735	0.8747	0.8535	0.8693	0.8741	0.8783	0.8536	0.8594
Porphyra umbilicalis	0.6174	0.5279	0.6162	0.4096	0.7329	0.6136	0.4557	0.6058
Panicum virgatum	0.7909	0.7704	0.7655	0.7568	0.7666	0.7781	0.7751	0.7796
Ricinus communis	0.8886	0.8962	0.9001	0.9059	0.9017	0.9147	0.9009	0.9022
Sorghum bicolor	0.8017	0.7784	0.7738	0.7632	0.7891	0.7936	0.8164	0.8389
Setaria italica	0.8776	0.8637	0.8516	0.8431	0.8568	0.8656	0.8291	0.8413
Solanum lycopersicum	0.7319	0.7341	0.7260	0.7589	0.7617	0.7691	0.7630	0.7704
Selaginella moellendorffii	0.7068	0.6898	0.6500	0.6974	0.5819	0.6848	0.7328	0.7109
Schrenkiella parvula	0.8742	0.8658	0.8636	0.8717	0.8705	0.8822	0.8776	0.8827
Spirodela polyrhiza	0.7821	0.7928	0.7926	0.7694	0.7783	0.8112	0.8212	0.8210
Salix purpurea	0.8829	0.8864	0.8770	0.8857	0.8812	0.8962	0.8985	0.9100
Solanum tuberosum	0.7382	0.7387	0.7547	0.7852	0.7506	0.7673	0.8304	0.7972
Triticum aestivum	0.8546	0.8455	0.8648	0.8485	0.8569	0.8700	0.8637	0.8659
Theobroma cacao	0.8309	0.8247	0.8041	0.8240	0.8304	0.8373	0.8413	0.8516
Trifolium pratense	0.6771	0.6729	0.7212	0.7090	0.7126	0.7495	0.7820	0.7863
Volvox carteri	0.7378	0.7370	0.8718	0.8127	0.8787	0.8728	0.8637	0.8753
Vigna unguiculata	0.8460	0.8612	0.8187	0.8493	0.8641	0.8627	0.8313	0.8541
Vitis vinifera	0.7987	0.7995	0.7761	0.8269	0.8172	0.8515	0.8476	0.8320
Zostera marina	0.7265	0.7250	0.8099	0.8546	0.7695	0.8458	0.9200	0.8456
Zea mays	0.8387	0.7659	0.7934	0.7759	0.7920	0.8091	0.8555	0.8686

Table S3: Genic F1 for 800 randomly selected subsequences of each training and validation vertebrate species and for each model released during development. These results should be considered as illustrative only since the genomes were used as part of the training process.

	v0.3_a_0400	v0.3_a_0300	v0.3_a_0200	*v0.3_m_0100	v0.3_m_0090	v0.3_m_0080
Acanthochromis polyacanthus	0.7729	0.8100	0.8452	0.9070	0.8831	0.8913
Acanthopagrus latus	0.7963	0.8310	0.8557	0.8635	0.8333	0.8614
Acinonyx jubatus	0.7403	0.8322	0.8124	0.8767	0.8890	0.8978
Ailuropoda melanoleuca	0.7889	0.8789	0.8635	0.8948	0.8686	0.8995
Alligator mississippiensis	0.6945	0.8088	0.8321	0.8790	0.9069	0.9323
Alosa sapidissima	0.8153	0.8341	0.8836	0.8135	0.8237	0.8364
Amphiprion ocellaris	0.7914	0.8397	0.8588	0.8882	0.8631	0.9055
Anabas testudineus	0.8165	0.8572	0.9044	0.8918	0.8819	0.8922
Anarrhichthys ocellatus	0.7974	0.8265	0.8582	0.8912	0.8491	0.9035
Anas platyrhynchos	0.7569	0.8288	0.8266	0.8934	0.9225	0.9094
Anolis carolinensis	0.5957	0.6360	0.7079	0.6606	0.7435	0.7970
Anser cygnoides	0.7532	0.8081	0.8307	0.8930	0.9098	0.9095
Antrostomus carolinensis	0.9402	0.9240	0.9736	0.9427	0.9658	0.9473
Aotus nancymaae	0.7457	0.8328	0.8378	0.8530	0.8626	0.8946
Apteryx rowi	0.8437	0.8728	0.8809	0.8833	0.9215	0.8847
Aquila chrysaetos	0.8344	0.8625	0.8715	0.8802	0.9121	0.8944
Archocentrus centrarchus	0.7289	0.7862	0.8148	0.8390	0.8777	0.8705
Arvicola amphibius	0.7235	0.8493	0.7998	0.8712	0.8879	0.8960
Astatotilapia calliptera	0.7390	0.7624	0.8031	0.8367	0.8204	0.8220
Astyanax mexicanus	0.7465	0.7824	0.8126	0.8357	0.8945	0.8636
Athene cunicularia	0.8345	0.8813	0.8822	0.8639	0.9052	0.9032
Austrofundulus limnaeus	0.7436	0.7641	0.8184	0.8548	0.8111	0.8258
Betta splendens	0.7863	0.7971	0.8273	0.8613	0.8436	0.8602
Bison bison	0.6901	0.8173	0.8332	0.8804	0.8377	0.8674
Boleophthalmus pectinirostris	0.7114	0.7401	0.7832	0.8139	0.7878	0.8221
Bos indicus	0.6925	0.8117	0.8086	0.7800	0.7677	0.7975
Bos mutus	0.7271	0.8334	0.8099	0.9077	0.9094	0.9128
Bos taurus	0.7096	0.7995	0.7845	0.8495	0.8529	0.8568
Bubalus bubalis	0.6715	0.8177	0.7995	0.8572	0.8312	0.8429
Calidris pugnax	0.8577	0.8747	0.8826	0.8803	0.9061	0.8969
Callithrix jacchus	0.0000	0.0000	0.0000	0.0000	0.0000	0.0000
Callorhinchus milii	0.6711	0.6581	0.7376	0.7783	0.7692	0.8273
Callorhinus ursinus	0.7433	0.8738	0.8441	0.8852	0.8770	0.8973
Calypte anna	0.8583	0.8842	0.9141	0.8894	0.8972	0.9159
Camarhynchus parvulus	0.8305	0.8540	0.8541	0.8851	0.9156	0.9274
Camelus dromedarius	0.7704	0.8569	0.8330	0.8769	0.8956	0.8984
Canis lupus dingo	0.6921	0.8054	0.7857	0.9016	0.8786	0.8952
Canis lupus familiaris	0.7343	0.8303	0.8224	0.8582	0.8306	0.8547
Capra hircus	0.7385	0.8479	0.8064	0.8432	0.8257	0.8778
Carassius auratus	0.7233	0.7595	0.8214	0.8380	0.8469	0.8496
Cariama cristata	0.9284	0.9473	0.9718	0.9319	0.9712	0.9527
Carlito syrichta	0.7876	0.8915	0.8042	0.8481	0.9093	0.9116
Castor canadensis	0.7146	0.8392	0.8117	0.8853	0.8790	0.8879
Catharus ustulatus	0.8136	0.8186	0.8419	0.8183	0.8811	0.8684
Cavia porcellus	0.7773	0.8777	0.8718	0.8302	0.8833	0.8875
Cebus imitator	0.7023	0.8088	0.7838	0.8606	0.8728	0.9117
Centrocercus urophasianus	0.8529	0.9099	0.8752	0.9157	0.9492	0.9283
Ceratotherium simum	0.7442	0.8683	0.8417	0.8588	0.8570	0.8888
Cercocebus atys	0.6955	0.7833	0.7864	0.8656	0.8360	0.8733
Cervus elaphus	0.7457	0.8487	0.8006	0.8584	0.8571	0.8327
Chaetura pelagica	0.8560	0.8888	0.9081	0.8712	0.8965	0.9083
Charadrius vociferus	0.8488	0.8787	0.8983	0.8773	0.9086	0.8673
Cheilinus undulatus	0.7347	0.7980	0.8314	0.8070	0.8056	0.8224
Chelonoidis abingdonii	0.6704	0.7654	0.8020	0.7018	0.8333	0.8687
Chiloscyllium plagiosum	0.4726	0.3928	0.5364	0.5829	0.6710	0.7247
Chinchilla lanigera	0.7071	0.7722	0.7511	0.8423	0.8497	0.8763
Chlamydotis macqueenii	0.9075	0.8631	0.9311	0.9659	0.9703	0.9635
Chlorocebus sabaeus	0.6713	0.8050	0.7995	0.8624	0.8965	0.8976
Chrysemys picta	0.6202	0.7283	0.8221	0.7267	0.8451	0.8727
Chrysochloris asiatica	0.5460	0.6958	0.6991	0.7297	0.7872	0.7690
Clupea harengus	0.8608	0.8767	0.9058	0.9051	0.8971	0.9243
Colobus angolensis	0.6348	0.7757	0.7578	0.8659	0.8481	0.8717
Columba livia	0.8243	0.8576	0.8604	0.8656	0.8992	0.9124
Corvus moneduloides	0.8137	0.8371	0.8447	0.8481	0.8797	0.8931
Cottoperca gobio	0.7632	0.8189	0.8550	0.9026	0.8842	0.9202
Coturnix japonica	0.8240	0.8661	0.8618	0.8741	0.9145	0.9183
Cricetulus griseus	0.8098	0.8383	0.8367	0.8934	0.8633	0.8809
Crocodylus porosus	0.7574	0.8629	0.8683	0.8392	0.8641	0.9042
Cyanistes caeruleus	0.7895	0.8224	0.8462	0.8205	0.8510	0.8410
Cyclopterus lumpus	0.8155	0.8574	0.8851	0.8848	0.8654	0.8937
Cygnus atratus	0.8210	0.8804	0.8970	0.9158	0.9337	0.9382
Cygnus olor	0.8468	0.8969	0.8834	0.8583	0.9072	0.8878
Cynoglossus semilaevis	0.7403	0.7962	0.8294	0.8645	0.8605	0.8710
Cyprinodon tularosa	0.6824	0.7311	0.8213	0.8357	0.8316	0.8092
Cyprinodon variegatus	0.7409	0.8035	0.8572	0.8081	0.7994	0.8282
Cyprinus carpio	0.7624	0.7759	0.8079	0.8340	0.8417	0.8125
Danio rerio	0.6925	0.7283	0.7929	0.8016	0.8579	0.8182

	v0.3_a_0400	v0.3_a_0300	v0.3_a_0200	*v0.3_m_0100	v0.3_m_0090	v0.3_m_0080
Dasypus novemcinctus	0.6809	0.7740	0.7690	0.8533	0.7980	0.8106
Delphinapterus leucas	0.7281	0.8364	0.8117	0.8370	0.8668	0.8758
Denticeps clupeoides	0.8457	0.8295	0.8723	0.9010	0.8445	0.8636
Dipodomys ordii	0.6923	0.8142	0.8283	0.8559	0.8807	0.8549
Dromaius novaehollandiae	0.8303	0.9060	0.8778	0.8707	0.8999	0.9088
Dromiciops gliroides	0.5498	0.6577	0.6306	0.8033	0.7189	0.7734
Echeneis naucrates	0.7755	0.8264	0.8449	0.9031	0.8734	0.9043
Echinops telfairi	0.6588	0.7631	0.7739	0.7645	0.7560	0.7745
Electrophorus electricus	0.8761	0.8765	0.9071	0.8771	0.8864	0.9096
Equus asinus	0.7102	0.8228	0.7728	0.8424	0.8236	0.8532
Equus caballus	0.7224	0.8516	0.8353	0.8597	0.8742	0.8809
Erinaceus europaeus	0.6916	0.8013	0.7975	0.7074	0.7567	0.7335
Erpetoichthys calabaricus	0.5177	0.5833	0.6923	0.6446	0.7802	0.6705
Esox lucius	0.6983	0.7077	0.7664	0.8050	0.7730	0.8079
Etheostoma spectabile	0.7779	0.8152	0.8552	0.8674	0.8768	0.8930
Eurypyga helias	0.8972	0.9227	0.9681	0.9286	0.9368	0.9328
Falco peregrinus	0.8550	0.8742	0.8826	0.8611	0.8982	0.8800
Felis catus	0.7151	0.8377	0.8171	0.8673	0.8664	0.8996
Ficedula albicollis	0.8299	0.8513	0.8669	0.8696	0.8869	0.9000
Fukomys damarensis	0.7233	0.8163	0.7995	0.7496	0.7607	0.7729
Fulmarus glacialis	0.8862	0.8983	0.9433	0.9663	0.9645	0.9843
Fundulus heteroclitus	0.6735	0.7135	0.7649	0.7962	0.7993	0.8048
Gadus morhua	0.8047	0.8165	0.8522	0.8646	0.8040	0.8560
Gallus gallus	0.8477	0.8746	0.9009	0.9156	0.9288	0.9073
Gambusia affinis	0.7058	0.7625	0.7953	0.8526	0.8238	0.8607
Gasterosteus aculeatus	0.8055	0.8303	0.8659	0.8816	0.7965	0.8763
Gavialis gangeticus	0.7130	0.7407	0.7996	0.7640	0.8428	0.8538
Geospiza fortis	0.7879	0.8068	0.8179	0.8085	0.8638	0.8875
Geotrypetes seraphini	0.3106	0.4026	0.4621	0.4784	0.5621	0.5263
Gopherus evgoodei	0.6448	0.7320	0.8020	0.7659	0.9021	0.8879
Gorilla gorilla	0.6616	0.8438	0.8177	0.8810	0.8943	0.9017
Gouania willdenowi	0.6980	0.7694	0.8158	0.8162	0.8361	0.8118
Haliaeetus albicilla	0.9232	0.9073	0.9408	0.9062	0.9270	0.9338
Haplochromis burtoni	0.8083	0.8345	0.8636	0.8277	0.8483	0.8346
Heterocephalus glaber	0.6814	0.7717	0.7660	0.8693	0.8636	0.8717
Hippocampus comes	0.6751	0.7246	0.7748	0.8548	0.8344	0.8079
Hippoglossus stenolepis	0.8119	0.8474	0.8657	0.8582	0.8224	0.8815
Hipposideros armiger	0.6856	0.8000	0.8088	0.8688	0.8632	0.8557
Hirundo rustica	0.8267	0.8575	0.8602	0.8629	0.8906	0.9099
Homo sapiens	0.6078	0.7449	0.7283	0.8433	0.8291	0.8630
Hyaena hyaena	0.8026	0.8870	0.8570	0.9404	0.9428	0.9583
Hylobates moloch	0.6727	0.7721	0.7819	0.8465	0.8621	0.8701
Ictalurus punctatus	0.7113	0.7256	0.7958	0.8556	0.8889	0.8905
Ictidomys tridecemlineatus	0.6743	0.7606	0.7492	0.8522	0.8333	0.8174
Jaculus jaculus	0.6658	0.8282	0.7807	0.7271	0.7328	0.7704
Kryptolebias marmoratus	0.7511	0.7810	0.8233	0.8260	0.8362	0.8372
Labrus bergylta	0.8179	0.8792	0.9107	0.8969	0.8777	0.9025
Lacerta agilis	0.7109	0.7657	0.7944	0.8108	0.8454	0.8914
Larimichthys crocea	0.7975	0.8334	0.8441	0.8615	0.8694	0.8988
Lates calcarifer	0.8130	0.8512	0.8722	0.8882	0.9003	0.9136
Latimeria chalumnae	0.6925	0.6751	0.7713	0.6050	0.7121	0.7017
Lemur catta	0.7886	0.8796	0.8949	0.8732	0.8800	0.9232
Lepidothrix coronata	0.8353	0.8639	0.8687	0.8611	0.8850	0.8931
Lepisosteus oculatus	0.7876	0.8131	0.8760	0.8476	0.8968	0.8958
Leptonychotes weddellii	0.6537	0.7513	0.7793	0.7994	0.8094	0.8094
Leptosomus discolor	0.9194	0.9149	0.9486	0.9331	0.9649	0.9591
Lipotes vexillifer	0.7600	0.8596	0.8643	0.8764	0.8927	0.9087
Lonchura striata	0.8371	0.8732	0.8760	0.8495	0.8956	0.8777
Lontra canadensis	0.7819	0.9012	0.8662	0.8956	0.8939	0.9064
Loxodonta africana	0.6455	0.8239	0.7698	0.8269	0.8368	0.8377
Lynx canadensis	0.7750	0.8648	0.8411	0.8941	0.9030	0.9061
Macaca fascicularis	0.6523	0.7769	0.7747	0.8517	0.8777	0.8932
Macaca mulatta	0.6871	0.7959	0.7639	0.9055	0.8851	0.8991
Macaca nemestrina	0.6656	0.7895	0.7922	0.8377	0.8490	0.8655
Manacus vitellinus	0.8629	0.8818	0.9083	0.8473	0.9142	0.8750
Mandrillus leucophaeus	0.6919	0.7844	0.7977	0.8685	0.8743	0.8581
Marmota marmota	0.6938	0.8081	0.8254	0.8955	0.8801	0.9120
Mastacembelus armatus	0.8147	0.8509	0.8725	0.8758	0.8903	0.8838
Mauremys mutica	0.6368	0.7201	0.7558	0.7575	0.8498	0.8596
Mauremys reevesii	0.5762	0.6882	0.7337	0.7048	0.8391	0.8605
Maylandia zebra	0.7761	0.7849	0.8468	0.8667	0.8736	0.8930
Megalops cyprinoides	0.9099	0.8913	0.9228	0.9117	0.9037	0.9269
Melanotaenia boesemani	0.7149	0.7746	0.8213	0.7824	0.7725	0.7899
Meleagris gallopavo	0.8441	0.8494	0.8468	0.8909	0.8806	0.8850
Meles meles	0.7463	0.8142	0.8053	0.8767	0.8809	0.8804
Melopsittacus undulatus	0.8614	0.8980	0.9151	0.9005	0.9502	0.9444
Meriones unguiculatus	0.7991	0.8674	0.8996	0.8929	0.8898	0.8796

	v0.3_a_0400	v0.3_a_0300	v0.3_a_0200	*v0.3_m_0100	v0.3_m_0090	v0.3_m_0080
Mesitornis unicolor	0.8847	0.9015	0.9275	0.9634	0.9757	0.9776
Mesocricetus auratus	0.6791	0.7765	0.7570	0.8142	0.7886	0.7950
Microcaecilia unicolor	0.3986	0.5304	0.5849	0.5688	0.6527	0.6556
Microcebus murinus	0.6365	0.7556	0.7200	0.8590	0.8389	0.8502
Micropterus dolomieu	0.7093	0.7632	0.8052	0.7737	0.7911	0.7973
Micropterus salmoides	0.7497	0.7973	0.8482	0.8008	0.8214	0.8387
Microtus ochrogaster	0.6597	0.7855	0.7594	0.8189	0.7962	0.8330
Microtus oregoni	0.7126	0.8554	0.8401	0.8560	0.8301	0.8394
Mirounga leonina	0.7224	0.8494	0.8163	0.8742	0.8608	0.8684
Molothrus ater	0.8212	0.8498	0.8336	0.8610	0.8875	0.8831
Monodelphis domestica	0.4884	0.6075	0.6208	0.7424	0.6823	0.7136
Monopterus albus	0.7697	0.7961	0.8153	0.8498	0.8473	0.8673
Morone saxatilis	0.8588	0.9116	0.9242	0.9214	0.9117	0.9278
Motacilla alba	0.7975	0.8437	0.8331	0.8585	0.8780	0.8925
Mus caroli	0.6420	0.7333	0.7210	0.8068	0.7468	0.7918
Mus musculus	0.6569	0.7635	0.7465	0.7984	0.8015	0.7972
Mus pahari	0.6912	0.8281	0.8271	0.8875	0.8504	0.8740
Mustela putorius	0.8166	0.8734	0.8788	0.9006	0.8843	0.8922
Myotis brandtii	0.6854	0.8051	0.7625	0.8647	0.8572	0.8626
Myotis lucifugus	0.7199	0.8217	0.8032	0.8219	0.8284	0.7880
Myotis myotis	0.7568	0.8438	0.8266	0.8935	0.8631	0.8349
Myripristis murdjan	0.8092	0.8463	0.8783	0.8793	0.8624	0.8896
Nannospalax galili	0.7046	0.7784	0.8047	0.8422	0.8276	0.8603
Nematolebias whitei	0.6166	0.6024	0.6943	0.7196	0.7484	0.7261
Neogale vison	0.7861	0.8620	0.8361	0.9512	0.9274	0.9335
Neolamprologus brichardi	0.7578	0.7922	0.8480	0.8673	0.8608	0.8613
Neomonachus schauinslandi	0.7900	0.8645	0.8726	0.8823	0.8985	0.9235
Nestor notabilis	0.8734	0.9157	0.9412	0.9462	0.9609	0.9449
Nipponia nippon	0.8965	0.9030	0.9219	0.8926	0.9073	0.9124
Nomascus leucogenys	0.7399	0.8743	0.8391	0.8741	0.8590	0.8876
Notechis scutatus	0.6637	0.7562	0.8188	0.6562	0.7925	0.7987
Nothobranchius furzeri	0.6195	0.6533	0.7168	0.7293	0.7083	0.7430
Nothoprocta perdicaria	0.8927	0.9198	0.9076	0.9297	0.9480	0.9458
Numida meleagris	0.8402	0.8797	0.8698	0.8786	0.9108	0.9020
Ochotona curzoniae	0.8322	0.8962	0.8894	0.8682	0.8844	0.8918
Ochotona princeps	0.8274	0.8504	0.8569	0.8728	0.8896	0.8742
Octodon degus	0.7135	0.7968	0.7902	0.7771	0.7672	0.7829
Odobenus rosmarus	0.7941	0.8743	0.8633	0.8851	0.8838	0.8995
Oncorhynchus keta	0.6718	0.6892	0.7786	0.7647	0.6929	0.7562
Oncorhynchus nerka	0.7198	0.7242	0.7828	0.7649	0.6809	0.7306
Orcinus orca	0.7368	0.8177	0.8301	0.8804	0.9021	0.9018
Oreochromis niloticus	0.7208	0.7543	0.7904	0.8180	0.7975	0.8085
Ornithorhynchus anatinus	0.7466	0.8566	0.8327	0.9461	0.8729	0.9057
Oryctolagus cuniculus	0.0000	0.0000	0.0000	0.0000	0.0000	0.0000
Oryzias latipes	0.6763	0.7151	0.7849	0.8603	0.7864	0.7991
Oryzias melastigma	0.6943	0.7138	0.7826	0.8302	0.7708	0.8128
Otolemur garnettii	0.6703	0.7818	0.7717	0.8653	0.8558	0.8837
Ovis aries	0.7260	0.8624	0.8275	0.8549	0.8733	0.8779
Oxyura jamaicensis	0.8184	0.8725	0.8759	0.8457	0.8956	0.8703
Pan paniscus	0.6713	0.7940	0.7671	0.8375	0.8399	0.8446
Pan troglodytes	0.0000	0.0000	0.0000	0.0000	0.0000	0.0000
Pangasianodon hypophthalmus	0.7973	0.8039	0.8643	0.8667	0.8925	0.8974
Panthera leo	0.7827	0.8930	0.8853	0.8791	0.8963	0.9184
Panthera pardus	0.7626	0.8581	0.8511	0.8133	0.8017	0.8370
Panthera tigris	0.7686	0.8813	0.8469	0.8940	0.8866	0.9095
Pantherophis guttatus	0.5747	0.6495	0.7469	0.6604	0.7602	0.7803
Papio anubis	0.6911	0.8027	0.7883	0.8941	0.9162	0.9150
Parambassis ranga	0.8361	0.8515	0.8759	0.9085	0.9003	0.9117
Paramormyrops kingsleyae	0.7971	0.8123	0.8504	0.8952	0.8698	0.8872
Parus major	0.8088	0.8340	0.8207	0.8562	0.8871	0.8800
Passer montanus	0.8021	0.8412	0.8236	0.8663	0.9216	0.9105
Pelecanus crispus	0.9283	0.9430	0.9674	0.9640	0.9809	0.9609
Pelodiscus sinensis	0.6073	0.7397	0.7546	0.7324	0.8623	0.8677
Perca flavescens	0.7971	0.8236	0.8580	0.8413	0.8559	0.8601
Perca fluviatilis	0.7724	0.8050	0.8420	0.7931	0.7704	0.8373
Periophthalmus magnuspinnatus	0.7736	0.7790	0.8273	0.8632	0.8547	0.8560
Peromyscus maniculatus	0.6787	0.7805	0.7503	0.8651	0.8560	0.8529
Petromyzon marinus	0.7556	0.5831	0.5803	0.5790	0.5777	0.6110
Phaethon lepturus	0.9349	0.9568	0.9650	0.9475	0.9530	0.9606
Phalacrocorax carbo	0.8635	0.8929	0.9277	0.9395	0.9643	0.9639
Phascolarctos cinereus	0.6398	0.7395	0.7381	0.8784	0.8395	0.8530
Phoca vitulina	0.8121	0.8754	0.8723	0.8615	0.8469	0.8693
Phocoena sinus	0.7288	0.8344	0.8451	0.8624	0.8932	0.8906
Phyllostomus discolor	0.7272	0.8400	0.8278	0.9008	0.9030	0.9171
Phyllostomus hastatus	0.7591	0.8600	0.8303	0.8253	0.8186	0.8455
Piliocolobus tephrosceles	0.7129	0.8030	0.8138	0.8920	0.8747	0.8796
Pipistrellus kuhlii	0.7466	0.8222	0.8535	0.8619	0.8476	0.8324

	v0.3_a_0400	v0.3_a_0300	v0.3_a_0200	*v0.3_m_0100	v0.3_m_0090	v0.3_m_0080
Pipra filicauda	0.8339	0.8793	0.8898	0.8886	0.9136	0.9219
Poecilia formosa	0.7538	0.7963	0.8334	0.8709	0.8160	0.8602
Poecilia latipinna	0.7646	0.7969	0.8522	0.8730	0.8577	0.8617
Poecilia mexicana	0.7742	0.8015	0.8607	0.8374	0.7977	0.8458
Poecilia reticulata	0.7356	0.7766	0.8323	0.8862	0.8536	0.8725
Pogona vitticeps	0.6905	0.7704	0.8057	0.7810	0.8519	0.8756
Polypterus senegalus	0.5420	0.6130	0.6766	0.5905	0.7531	0.6710
Pongo abelii	0.6661	0.7900	0.7908	0.8246	0.8385	0.8464
Propithecus coquereli	0.7497	0.7970	0.8187	0.8276	0.8206	0.8782
Protobothrops mucrosquamatus	0.6661	0.7901	0.8709	0.7558	0.8567	0.8690
Pseudochaenichthys georgianus	0.7557	0.7631	0.8167	0.7820	0.7383	0.7641
Pseudopodoces humilis	0.8757	0.9043	0.8991	0.8703	0.9174	0.9025
Pterocles gutturalis	0.8700	0.9103	0.9106	0.9621	0.9863	0.9692
Pteropus alecto	0.7630	0.8287	0.8363	0.8567	0.8876	0.8946
Pteropus vampyrus	0.7849	0.8464	0.8499	0.8563	0.8546	0.8504
Puma concolor	0.7462	0.8270	0.8080	0.8548	0.8281	0.8880
Puma yagouaroundi	0.7639	0.8823	0.8244	0.8694	0.8794	0.8866
Pundamilia nyererei	0.7808	0.8358	0.8721	0.9029	0.8954	0.9160
Puntigrus tetrazona	0.8008	0.8313	0.8803	0.8837	0.8908	0.8727
Pygocentrus nattereri	0.8419	0.8617	0.8770	0.8618	0.8601	0.8776
Pygoscelis adeliae	0.8182	0.8770	0.8566	0.8352	0.8856	0.8690
Pyrgilauda ruficollis	0.8599	0.8859	0.8700	0.9137	0.9532	0.9274
Rana temporaria	0.4151	0.3882	0.4452	0.4833	0.5270	0.4225
Rattus norvegicus	0.6279	0.8062	0.7452	0.8197	0.8105	0.8356
Rattus rattus	0.6681	0.8215	0.8145	0.8379	0.8340	0.8474
Rhinopithecus bieti	0.6593	0.7799	0.7779	0.8871	0.8526	0.8654
Rhinopithecus roxellana	0.6976	0.8199	0.8138	0.9183	0.9146	0.9377
Saimiri boliviensis	0.7678	0.8432	0.8473	0.8593	0.8606	0.8830
Salarias fasciatus	0.7939	0.7699	0.7958	0.8696	0.8156	0.8463
Salmo salar	0.6675	0.6927	0.7704	0.7509	0.6822	0.6803
Salmo trutta	0.6921	0.6773	0.7530	0.7974	0.7413	0.7485
Salvelinus namaycush	0.7228	0.7196	0.7742	0.7887	0.7405	0.7691
Salvelinus sp. IW2-2015	0.6466	0.6592	0.7307	0.7752	0.7339	0.7867
Sander lucioperca	0.7902	0.8173	0.8692	0.8381	0.8613	0.8566
Sarcophilus harrisii	0.5454	0.6652	0.6518	0.6847	0.6352	0.6702
Sceloporus undulatus	0.5918	0.6536	0.7115	0.6855	0.8026	0.8163
Scleropages formosus	0.8639	0.8515	0.8937	0.8806	0.8786	0.8944
Scophthalmus maximus	0.8089	0.8208	0.8587	0.8661	0.8082	0.8585
Scyliorhinus canicula	0.3090	0.2630	0.3380	0.3480	0.4681	0.4307
Sebastes umbrosus	0.7850	0.8409	0.8674	0.8596	0.8510	0.8906
Serinus canaria	0.8526	0.8913	0.8725	0.8818	0.9248	0.9137
Seriola dumerili	0.8789	0.8903	0.9187	0.9093	0.9240	0.9332
Seriola lalandi	0.8258	0.8717	0.8640	0.8966	0.8609	0.9300
Simochromis diagramma	0.7358	0.7795	0.8274	0.8565	0.8395	0.8537
Siniperca chuatsi	0.7620	0.8057	0.8395	0.8342	0.8455	0.8682
Sinocyclocheilus grahami	0.7839	0.7730	0.8559	0.8540	0.8791	0.8456
Sinocyclocheilus rhinocerous	0.8064	0.7963	0.8511	0.8466	0.8889	0.8556
Solea senegalensis	0.7850	0.8185	0.8516	0.8652	0.8516	0.8792
Sorex araneus	0.6878	0.7404	0.7762	0.8056	0.8135	0.8004
Stegastes partitus	0.8440	0.8544	0.8928	0.9073	0.9013	0.9102
Struthio camelus	0.8465	0.8815	0.8681	0.8915	0.8999	0.8812
Sturnira hondurensis	0.7604	0.8821	0.8792	0.8826	0.8627	0.9021
Sturnus vulgaris	0.8830	0.8857	0.8891	0.9020	0.9349	0.9420
Sus scrofa	0.6531	0.7196	0.6824	0.7670	0.8342	0.7483
Tachyglossus aculeatus	0.7532	0.8413	0.8672	0.9202	0.8624	0.8992
Tachysurus fulvidraco	0.8023	0.8300	0.8723	0.8311	0.8405	0.8379
Taeniopygia guttata	0.8238	0.8457	0.8621	0.8707	0.8833	0.8755
Takifugu rubripes	0.7626	0.8294	0.8479	0.8791	0.8428	0.8994
Talpa occidentalis	0.7569	0.8865	0.8806	0.8887	0.8370	0.8640
Tauraco erythrolophus	0.8541	0.9034	0.9505	0.9460	0.9733	0.9581
Terrapene carolina	0.6447	0.7093	0.7861	0.7020	0.8515	0.8665
Thalassophryne amazonica	0.4337	0.4496	0.4950	0.5572	0.5888	0.5529
Theropithecus gelada	0.7000	0.8171	0.7782	0.8713	0.8277	0.8899
Thunnus albacares	0.7660	0.8238	0.8653	0.8748	0.8860	0.8991
Thunnus maccoyii	0.7718	0.8103	0.8597	0.8618	0.8660	0.8771
Tinamus guttatus	0.8191	0.8591	0.8507	0.8293	0.8563	0.8516
Toxotes jaculatrix	0.8514	0.8869	0.9036	0.8948	0.8753	0.8947
Trematomus bernacchii	0.7795	0.8044	0.8502	0.8496	0.8282	0.8640
Tupaia chinensis	0.6393	0.8078	0.7649	0.8242	0.8283	0.8632
Tursiops truncatus	0.6946	0.8297	0.7865	0.8697	0.8896	0.8921
Urocitellus parryii	0.6537	0.7959	0.7810	0.8885	0.8417	0.8676
Ursus americanus	0.7998	0.8937	0.8698	0.9329	0.9085	0.9462
Ursus arctos	0.7846	0.9092	0.8521	0.9086	0.9147	0.9357
Ursus maritimus	0.8140	0.8917	0.8814	0.9187	0.9332	0.9377
Varanus komodoensis	0.6666	0.7462	0.7687	0.7948	0.8798	0.9127
Vicugna pacos	0.7934	0.9035	0.8585	0.8590	0.8551	0.8585
Vombatus ursinus	0.7312	0.8086	0.8275	0.8475	0.7957	0.8256
Vulpes vulpes	0.7509	0.8776	0.8297	0.9437	0.8974	0.9239
Xenopus laevis	0.5822	0.6178	0.7227	0.6470	0.7472	0.7550
Xenopus tropicalis	0.6213	0.7678	0.7911	0.7461	0.8862	0.8565
Xiphophorus couchianus	0.7203	0.7716	0.8176	0.8764	0.8605	0.8778
Xiphophorus maculatus	0.7670	0.8096	0.8545	0.8724	0.8162	0.8608
Zonotrichia albicollis	0.8094	0.8439	0.8287	0.8586	0.8717	0.8763

Table S4: Genic F1 for 800 randomly selected subsequences of each training and validation invertebrate species and for each model released during development. These results should be considered as illustrative only since the genomes were used as part of the training process.

	v0.3_a_0600	v0.3_a_0500	v0.3_a_0400	v0.3_a_0300	v0.3_m_0200	v0.3_m_0100
Acanthaster planci	0.8740	0.8854	0.8523	0.8810	0.8841	0.8969
Acromyrmex echinatior	0.8845	0.8791	0.8886	0.8909	0.9044	0.9180
Acropora millepora	0.7260	0.6809	0.7283	0.6505	0.7198	0.7423
Actinia tenebrosa	0.8480	0.8442	0.8307	0.7931	0.8614	0.8767
Acyrthosiphon pisum	0.7593	0.8012	0.8702	0.8537	0.8688	0.8939
Aedes aegypti	0.8914	0.9060	0.9169	0.9255	0.9406	0.9464
Aethina tumida	0.8301	0.8550	0.8808	0.8797	0.9084	0.9316
Agrilus planipennis	0.8482	0.8517	0.8910	0.8468	0.9078	0.9279
Amphibalanus amphitrite	0.8566	0.8718	0.8600	0.9044	0.8696	0.8762
Amphimedon queenslandica	0.7216	0.7950	0.6782	0.6428	0.8203	0.8690
Amyelois transitella	0.8675	0.8725	0.8818	0.8849	0.9014	0.9162
Anopheles albimanus	0.9148	0.9307	0.9340	0.9479	0.9530	0.9608
Anopheles arabiensis	0.9047	0.9075	0.9227	0.9331	0.9346	0.9476
Anopheles coluzzii	0.9089	0.9262	0.9216	0.9475	0.9500	0.9494
Anopheles merus	0.9315	0.9372	0.9334	0.9572	0.9586	0.9627
Anopheles stephensi	0.9136	0.9236	0.9192	0.9439	0.9458	0.9572
Anoplophora glabripennis	0.9000	0.9032	0.9254	0.9290	0.9384	0.9399
Aphidius gifuensis	0.7875	0.9316	0.9506	0.9420	0.9513	0.9577
Aphis gossypii	0.8220	0.8774	0.9067	0.9123	0.9290	0.9334
Apis cerana	0.8964	0.9359	0.9384	0.9445	0.9394	0.9601
Apis florea	0.8768	0.9146	0.9121	0.9266	0.9130	0.9352
Apis laboriosa	0.8979	0.9338	0.9353	0.9429	0.9513	0.9554
Apis mellifera	0.9002	0.9262	0.9290	0.9377	0.9489	0.9597
Aplysia californica	0.8190	0.8104	0.7854	0.8109	0.8370	0.8235
Asterias rubens	0.9083	0.8982	0.8963	0.9103	0.9200	0.9277
Atta colombica	0.9304	0.9189	0.9379	0.9357	0.9508	0.9557
Bactrocera tryoni	0.8783	0.8865	0.9126	0.9136	0.9267	0.9311
Belonocnema kinseyi	0.8497	0.8065	0.8691	0.8513	0.8892	0.8663
Belonocnema treatae	0.8449	0.8175	0.8577	0.8015	0.8533	0.8617
Bemisia tabaci	0.8712	0.8544	0.9093	0.8696	0.9053	0.9131
Bicyclus anynana	0.8508	0.8605	0.8787	0.9054	0.9069	0.9137
Biomphalaria glabrata	0.8582	0.8437	0.8487	0.8034	0.9044	0.8982
Bombus bifarius	0.8869	0.9004	0.8899	0.9086	0.9117	0.9264
Bombus pyrosoma	0.9051	0.9208	0.9292	0.9176	0.9347	0.9400
Bombus terrestris	0.8756	0.8873	0.9031	0.9030	0.9201	0.9347
Bombus vancouverensis	0.8769	0.8760	0.8737	0.8876	0.8994	0.9145
Bombyx mandarina	0.8557	0.8455	0.9095	0.8765	0.9143	0.9206
Bombyx mori	0.8203	0.8046	0.8595	0.8427	0.8819	0.8842
Bradysia coprophila	0.8100	0.8381	0.8636	0.8611	0.9175	0.9303
Brugia malayi	0.7422	0.8422	0.7778	0.8250	0.8544	0.8896
Caenorhabditis briggsae	0.8019	0.8419	0.7459	0.7544	0.8323	0.8712
Caenorhabditis elegans	0.8727	0.8765	0.8160	0.8102	0.8910	0.9155
Camponotus floridanus	0.8713	0.8691	0.8778	0.8896	0.8953	0.8926
Capsaspora owczarzaki	0.8460	0.8863	0.8611	0.8774	0.8720	0.8794
Centruroides sculpturatus	0.6961	0.7475	0.7936	0.7789	0.8170	0.8242
Ceratitis capitata	0.9273	0.9257	0.9366	0.9453	0.9663	0.9511
Chelonus insularis	0.9160	0.9238	0.9481	0.9364	0.9421	0.9493
Cimex lectularius	0.8267	0.8372	0.8496	0.8160	0.8819	0.9011
Ciona intestinalis	0.8330	0.8424	0.8522	0.8108	0.9038	0.9228
Coccinella septempunctata	0.7932	0.7941	0.8351	0.8111	0.8298	0.8461
Colletes gigas	0.8949	0.8955	0.9107	0.9110	0.9239	0.9364
Contarinia nasturtii	0.9155	0.9295	0.9383	0.9452	0.9476	0.9510
Cotesia glomerata	0.8439	0.8378	0.8642	0.8614	0.8890	0.8914
Crassostrea gigas	0.8472	0.8138	0.8643	0.8116	0.8905	0.9007
Cryptotermes secundus	0.8900	0.8623	0.8889	0.8685	0.8993	0.8751
Daphnia magna	0.7674	0.7721	0.7813	0.7647	0.8249	0.8596
Daphnia pulicaria	0.8698	0.8776	0.8789	0.8545	0.9158	0.9250
Dendroctonus ponderosae	0.8522	0.8796	0.8457	0.8728	0.9082	0.9200
Dendronephthya gigantea	0.7805	0.7355	0.8072	0.7205	0.8190	0.8434
Dermacentor silvarum	0.6937	0.7377	0.6948	0.7784	0.7351	0.7586
Dermatophagoides pteronyssinus	0.7055	0.8907	0.9125	0.9223	0.9094	0.9228
Diabrotica virgifera	0.6844	0.7420	0.7658	0.7491	0.7723	0.7792
Diachasma alloeum	0.8464	0.8188	0.8450	0.8276	0.8633	0.8669
Diaphorina citri	0.6285	0.7340	0.7394	0.7428	0.7077	0.8017
Diuraphis noxia	0.8196	0.8584	0.8823	0.8903	0.9014	0.9024
Drosophila ananassae	0.8782	0.8679	0.9056	0.9062	0.9172	0.9216
Drosophila biarmipes	0.9328	0.9265	0.9510	0.9491	0.9612	0.9658
Drosophila bipectinata	0.9177	0.9102	0.9252	0.9317	0.9455	0.9502
Drosophila busckii	0.9360	0.9389	0.9547	0.9612	0.9715	0.9743
Drosophila elegans	0.9211	0.9075	0.9243	0.9399	0.9478	0.9527
Drosophila erecta	0.9400	0.9366	0.9548	0.9566	0.9714	0.9711
Drosophila eugracilis	0.9124	0.9022	0.9257	0.9233	0.9467	0.9459
Drosophila grimshawi	0.9444	0.9455	0.9557	0.9625	0.9729	0.9774
Drosophila hydei	0.9452	0.9475	0.9558	0.9623	0.9737	0.9770
Drosophila innubila	0.9473	0.9372	0.9560	0.9599	0.9670	0.9716
Drosophila kikkawai	0.9182	0.9116	0.9361	0.9402	0.9416	0.9501
Drosophila mauritiana	0.9348	0.9242	0.9386	0.9435	0.9562	0.9587

	v0.3_a_0600	v0.3_a_0500	v0.3_a_0400	v0.3_a_0300	v0.3_m_0200	v0.3_m_0100
Drosophila melanogaster	0.9372	0.9295	0.9485	0.9524	0.9701	0.9698
Drosophila miranda	0.8743	0.8744	0.8855	0.9017	0.9229	0.9221
Drosophila mojavensis	0.9334	0.9275	0.9452	0.9469	0.9617	0.9621
Drosophila navojoa	0.9510	0.9533	0.9463	0.9618	0.9715	0.9744
Drosophila novamexicana	0.9242	0.9303	0.9434	0.9498	0.9558	0.9639
Drosophila persimilis	0.9140	0.9083	0.9365	0.9405	0.9586	0.9647
Drosophila pseudoobscura	0.9350	0.9337	0.9473	0.9516	0.9621	0.9620
Drosophila rhopaloa	0.9192	0.9178	0.9439	0.9466	0.9558	0.9551
Drosophila santomea	0.9255	0.9206	0.9346	0.9392	0.9506	0.9519
Drosophila serrata	0.8898	0.8932	0.9163	0.9183	0.9370	0.9409
Drosophila simulans	0.9305	0.9188	0.9420	0.9469	0.9645	0.9665
Drosophila subobscura	0.9344	0.9344	0.9506	0.9534	0.9686	0.9697
Drosophila suzukii	0.8889	0.8707	0.9090	0.9089	0.9307	0.9302
Drosophila teissieri	0.9264	0.9317	0.9485	0.9500	0.9677	0.9688
Exaiptasia diaphana	0.8570	0.8616	0.8623	0.8052	0.8842	0.8994
Folsomia candida	0.8580	0.8301	0.8538	0.8189	0.8848	0.9020
Fonticula alba	0.7532	0.7979	0.9050	0.9489	0.9329	0.9374
Fopius arisanus	0.8994	0.8935	0.9082	0.8903	0.9339	0.9448
Galendromus occidentalis	0.6194	0.6802	0.6731	0.6037	0.6533	0.6540
Galleria mellonella	0.8406	0.8772	0.9056	0.9100	0.9260	0.9083
Gigantopelta aegis	0.7722	0.7571	0.7729	0.7832	0.8182	0.8226
Glossina fuscipes	0.8824	0.8725	0.9157	0.9217	0.9503	0.9549
Habropoda laboriosa	0.8414	0.8500	0.8736	0.8675	0.8913	0.8956
Haliotis rubra	0.7497	0.8077	0.7895	0.8553	0.7986	0.8401
Halyomorpha halys	0.8537	0.8187	0.8794	0.8358	0.9129	0.8921
Harpegnathos saltator	0.8761	0.8718	0.8950	0.8925	0.8976	0.9100
Hermetia illucens	0.8111	0.7921	0.8479	0.8338	0.9378	0.9340
Homalodisca vitripennis	0.7267	0.7376	0.7775	0.8077	0.7923	0.7780
Homarus americanus	0.7212	0.7477	0.7335	0.7522	0.7705	0.7700
Ischnura elegans	0.8754	0.8747	0.8955	0.8872	0.9115	0.8978
Ixodes scapularis	0.5738	0.6326	0.6140	0.6501	0.6560	0.6855
Lepeophtheirus salmonis	0.8444	0.7622	0.8937	0.7936	0.8773	0.9222
Leptinotarsa decemlineata	0.8418	0.8732	0.8907	0.8903	0.9040	0.8798
Leptopilina heterotoma	0.8343	0.8407	0.8816	0.8558	0.8846	0.8900
Linepithema humile	0.8887	0.8943	0.9085	0.9088	0.9195	0.9283
Lingula anatina	0.9041	0.8999	0.8921	0.8940	0.9238	0.9252
Loa loa	0.6893	0.7909	0.7173	0.7807	0.7829	0.8368
Lottia gigantea	0.7270	0.7358	0.8043	0.7772	0.8016	0.8143
Lucilia sericata	0.8287	0.8619	0.9179	0.9201	0.9261	0.9394
Manduca sexta	0.8302	0.8483	0.8437	0.8609	0.8702	0.8782
Maniola jurtina	0.8092	0.8215	0.8505	0.8439	0.9019	0.9036
Megachile rotundata	0.9024	0.9075	0.9010	0.9102	0.9245	0.9222
Megalopta genalis	0.9278	0.9245	0.9344	0.9303	0.9489	0.9506
Melanaphis sacchari	0.8129	0.8342	0.8901	0.8884	0.9060	0.9211
Melitaea cinxia	0.7809	0.7841	0.8234	0.8123	0.8367	0.8427
Mercenaria mercenaria	0.7012	0.7028	0.7578	0.7251	0.7867	0.7792
Microplitis demolitor	0.8610	0.8804	0.9048	0.8987	0.9178	0.9244
Monosiga brevicollis	0.5102	0.6402	0.6431	0.6397	0.7082	0.4970
Musca domestica	0.8996	0.9138	0.9314	0.9158	0.9462	0.9571
Myzus persicae	0.7948	0.8217	0.8891	0.8795	0.8989	0.9099
Nasonia vitripennis	0.8979	0.9056	0.9182	0.9124	0.9213	0.9316
Necator americanus	0.7444	0.7529	0.5213	0.5586	0.5964	0.6707
Nematostella vectensis	0.8127	0.8079	0.7829	0.7774	0.8226	0.8244
Neodiprion fabricii	0.9167	0.9003	0.9081	0.9056	0.9269	0.9335
Neodiprion lecontei	0.8759	0.8845	0.8779	0.8792	0.8963	0.9133
Neodiprion pinetum	0.8877	0.8836	0.8857	0.8866	0.9032	0.9129
Neodiprion virginiana	0.9083	0.8922	0.8953	0.8902	0.9236	0.9324
Nicrophorus vespilloides	0.9398	0.9468	0.9527	0.9584	0.9629	0.9691
Nilaparvata lugens	0.8112	0.8187	0.8517	0.8161	0.8532	0.8519
Nomia melanderi	0.9154	0.9203	0.9119	0.9393	0.9301	0.9484
Nylanderia fulva	0.7754	0.7944	0.7910	0.8348	0.7982	0.8122
Octopus bimaculoides	0.6081	0.6985	0.8090	0.7626	0.9130	0.8882
Octopus sinensis	0.7075	0.7370	0.7544	0.7360	0.8247	0.8422
Octopus vulgaris	0.6717	0.6931	0.7049	0.7314	0.7985	0.8012
Odontomachus brunneus	0.8521	0.8420	0.8642	0.8475	0.8707	0.8915
Ooceraea biroi	0.8849	0.8873	0.8907	0.9019	0.9032	0.9146
Orbicella faveolata	0.8485	0.8344	0.8036	0.7851	0.8567	0.8831
Orussus abietinus	0.9061	0.9102	0.9146	0.9112	0.9365	0.9408
Osmia bicornis	0.8693	0.8630	0.8656	0.8797	0.8880	0.9030
Osmia lignaria	0.8662	0.8660	0.8717	0.8675	0.8952	0.9096
Ostrinia furnacalis	0.8299	0.8512	0.8606	0.8721	0.8600	0.8751
Papilio machaon	0.8433	0.8561	0.8690	0.8831	0.8990	0.9097
Papilio polytes	0.9055	0.9060	0.9239	0.9246	0.9439	0.9442
Papilio xuthus	0.8151	0.8262	0.8679	0.8505	0.8942	0.8904
Pararge aegeria	0.8517	0.8326	0.8791	0.8729	0.8949	0.9110
Parasteatoda tepidariorum	0.8185	0.7794	0.8624	0.7863	0.9057	0.8874
Patiria miniata	0.8540	0.8486	0.8574	0.8842	0.8978	0.9000

	v0.3_a_0600	v0.3_a_0500	v0.3_a_0400	v0.3_a_0300	v0.3_m_0200	v0.3_m_0100
Pecten maximus	0.8206	0.8298	0.8339	0.8413	0.8503	0.8601
Penaeus japonicus	0.7940	0.8071	0.7806	0.7865	0.8049	0.7994
Penaeus monodon	0.7454	0.7419	0.7243	0.7581	0.7644	0.7796
Photinus pyralis	0.7122	0.6935	0.7659	0.7279	0.8441	0.8540
Pieris brassicae	0.9046	0.8874	0.9160	0.9070	0.9436	0.9450
Pieris rapae	0.8943	0.8933	0.9090	0.9195	0.9326	0.9404
Plutella xylostella	0.8360	0.8618	0.8563	0.8880	0.8697	0.8903
Pocillopora damicornis	0.8453	0.8211	0.8325	0.7794	0.8551	0.8809
Pogonomyrmex barbatus	0.9055	0.8955	0.9055	0.9076	0.9194	0.9304
Polistes canadensis	0.9429	0.9463	0.9510	0.9546	0.9593	0.9685
Pollicipes pollicipes	0.6390	0.6696	0.6878	0.8159	0.6999	0.7698
Pomacea canaliculata	0.8228	0.8545	0.8253	0.8269	0.8694	0.8835
Portunus trituberculatus	0.8168	0.8385	0.7977	0.8161	0.8253	0.8207
Pseudomyrmex gracilis	0.8776	0.8795	0.8813	0.8951	0.8834	0.8993
Rhagoletis zephyria	0.8519	0.8612	0.8602	0.8794	0.8719	0.8870
Rhipicephalus microplus	0.6435	0.7016	0.6394	0.6970	0.6816	0.6930
Rhipicephalus sanguineus	0.7290	0.7460	0.7487	0.7680	0.7803	0.7770
Saccoglossus kowalevskii	0.8026	0.8401	0.8565	0.8688	0.8711	0.8958
Salpingoeca rosetta	0.9075	0.9221	0.9045	0.9350	0.9165	0.9243
Scaptodrosophila lebanonensis	0.9184	0.9164	0.9338	0.9445	0.9654	0.9642
Schistosoma haematobium	0.3728	0.7168	0.6746	0.7371	0.6570	0.6387
Sitophilus oryzae	0.7633	0.7427	0.8080	0.8110	0.8235	0.8267
Solenopsis invicta	0.8172	0.8068	0.8522	0.8188	0.8513	0.8759
Sphaeroforma arctica	0.8359	0.8443	0.6231	0.7867	0.7976	0.8174
Spodoptera frugiperda	0.8716	0.8868	0.9029	0.9037	0.9055	0.9111
Spodoptera litura	0.8688	0.8692	0.8911	0.9004	0.9179	0.9290
Stegodyphus dumicola	0.7461	0.8138	0.7815	0.7953	0.8262	0.8042
Stomoxys calcitrans	0.8926	0.9198	0.9308	0.9211	0.9550	0.9564
Strongylocentrotus purpuratus	0.8920	0.8796	0.8888	0.9010	0.9119	0.9017
Stylophora pistillata	0.7933	0.7600	0.7762	0.7055	0.7854	0.8285
Tetranychus urticae	0.9088	0.9277	0.9441	0.9265	0.9410	0.9579
Thrips palmi	0.8940	0.9042	0.9115	0.8971	0.9134	0.9224
Trachymyrmex septentrionalis	0.9192	0.9136	0.9230	0.9282	0.9354	0.9424
Tribolium madens	0.8294	0.7831	0.8561	0.7953	0.9270	0.9356
Trichinella spiralis	0.6747	0.6734	0.6471	0.6674	0.6827	0.7247
Trichoplax adhaerens	0.8438	0.7539	0.8888	0.8226	0.8787	0.9028
Varroa destructor	0.8966	0.8876	0.8179	0.8515	0.9106	0.9026
Varroa jacobsoni	0.8789	0.8755	0.8161	0.8324	0.8721	0.8997
Venturia canescens	0.8104	0.8209	0.8212	0.8141	0.8382	0.8651
Vespa crabro	0.9104	0.9191	0.9227	0.9292	0.9331	0.9434
Vespa mandarinia	0.9175	0.9286	0.9308	0.9422	0.9501	0.9608
Vespula pensylvanica	0.9324	0.9393	0.9433	0.9488	0.9531	0.9541
Vollenhovia emeryi	0.8552	0.8474	0.8297	0.8793	0.8817	0.9016
Xenia sp. Carnegie-2017	0.7204	0.7636	0.8061	0.7369	0.8097	0.8272
Zerene cesonia	0.9142	0.9288	0.9385	0.9476	0.9532	0.9610
Zeugodacus cucurbitae	0.9259	0.9329	0.9394	0.9461	0.9554	0.9601
Zootermopsis nevadensis	0.8853	0.8900	0.9059	0.8972	0.9145	0.9151

Table S5: Phase F1 for test species.

species	group	Precision				Recall				F1			
		hel. BW	hel. post	genemark	augustus	hel. BW	hel. post	genemark	augustus	hel. BW	hel. post	genemark	augustus
Neurospora_crassa	fungi	0.9631	0.9631	0.9830	0.9862	0.9706	0.9731	0.9498	0.8447	0.9668	0.9681	0.9661	0.9100
Coccidioides_immitis	fungi	0.9629	0.9635	0.9794	0.9754	0.9258	0.9310	0.9129	0.8914	0.9440	0.9470	0.9449	0.9315
Fusarium_graminearum	fungi	0.9662	0.9672	0.9733	0.9770	0.9666	0.9670	0.9493	0.9006	0.9664	0.9671	0.9612	0.9373
Debaryomyces_hansenii	fungi	0.9976	0.9977	0.9980	0.9990	0.9910	0.9919	0.9817	0.9351	0.9943	0.9948	0.9898	0.9660
Aspergillus_terreus	fungi	0.9719	0.9723	0.9778	0.9788	0.9687	0.9695	0.9261	0.9086	0.9703	0.9709	0.9512	0.9424
Laccaria_bicolor	fungi	0.8845	0.8832	0.9010	0.9135	0.8497	0.8692	0.8346	0.7864	0.8668	0.8762	0.8666	0.8452
Papaver_somniferum	plant	0.4489	0.4473	0.2579	N/A	0.8209	0.8265	0.9017	N/A	0.5804	0.5804	0.4011	N/A
Arachis_hypogaea	plant	0.8359	0.8453	0.2561	N/A	0.8174	0.8233	0.8548	N/A	0.8266	0.8341	0.3941	N/A
Brassica_napus	plant	0.8038	0.8152	0.4996	N/A	0.8654	0.8684	0.9564	N/A	0.8335	0.8410	0.6563	N/A
Oryza_brachyantha	plant	0.9044	0.9036	0.4223	0.8494	0.9578	0.9695	0.3695	0.8278	0.9303	0.9354	0.3941	0.8385
Nicotiana_attenuata	plant	0.6556	0.7288	0.1460	0.4028	0.7825	0.7845	0.6814	0.8083	0.7135	0.7557	0.2404	0.5376
Solanum_pennellii	plant	0.8549	0.8574	0.3178	0.6104	0.9066	0.9140	0.8779	0.8205	0.8800	0.8848	0.4666	0.7000
Setaria_viridis	plant	0.8605	0.8498	0.2959	N/A	0.9334	0.9451	0.4137	N/A	0.8955	0.8949	0.3450	N/A
Vitis_riparia	plant	0.8694	0.8705	0.4070	N/A	0.9183	0.9288	0.9078	N/A	0.8932	0.8987	0.5620	N/A
Triticum_dicoccoides	plant	0.4030	0.4380	0.0638	0.3157	0.9162	0.9268	0.3439	0.7483	0.5598	0.5949	0.1077	0.4441
Phoenix_dactylifera	plant	0.8662	0.8639	0.1963	N/A	0.9034	0.9153	0.3410	N/A	0.8844	0.8888	0.2491	N/A
Coffea_arabica	plant	0.8150	0.8166	0.4290	N/A	0.8418	0.8503	0.8914	N/A	0.8282	0.8331	0.5793	N/A
Cannabis_sativa	plant	0.8909	0.9038	0.2952	N/A	0.8219	0.8269	0.8910	N/A	0.8550	0.8637	0.4434	N/A
Hibiscus_syriacus	plant	0.6004	0.5987	0.3197	N/A	0.9026	0.9130	0.9339	N/A	0.7211	0.7232	0.4763	N/A
Balaenoptera_musculus	vertebrate	0.9076	0.9183	0.0856	N/A	0.8661	0.8814	0.3234	N/A	0.8864	0.8994	0.1353	N/A
Xiphias_gladius	vertebrate	0.8972	0.9109	0.6034	N/A	0.9243	0.9490	0.9011	N/A	0.9106	0.9296	0.7228	N/A
Sparus_aurata	vertebrate	0.8426	0.8455	0.5456	N/A	0.9010	0.9312	0.8862	N/A	0.8708	0.8863	0.6754	N/A
Desmodus_rotundus	vertebrate	0.8833	0.8959	0.1159	N/A	0.8714	0.8881	0.3556	N/A	0.8773	0.8920	0.1749	N/A
Pseudonaja_textilis	vertebrate	0.6653	0.7376	0.1136	N/A	0.8870	0.9064	0.2363	N/A	0.7603	0.8133	0.1535	N/A
Falco_naumanni	vertebrate	0.8926	0.9011	0.0841	N/A	0.8955	0.9163	0.2165	N/A	0.8940	0.9086	0.1211	N/A
Chiroxiphia_lanceolata	vertebrate	0.8977	0.9072	0.0125	N/A	0.8999	0.9201	0.0207	N/A	0.8988	0.9136	0.0156	N/A
Myotis_davidii	vertebrate	0.8286	0.8380	0.1138	N/A	0.8716	0.8886	0.3426	N/A	0.8496	0.8625	0.1709	N/A
Lagopus_leucura	vertebrate	0.8877	0.9063	0.1767	N/A	0.9075	0.9285	0.2366	N/A	0.8975	0.9173	0.2023	N/A
Rhincodon_typus	vertebrate	0.7472	0.7623	0.0605	0.6611	0.8339	0.8497	0.0661	0.6150	0.7881	0.8036	0.0632	0.6372
Opisthocomus_hoazin	vertebrate	0.8705	0.8783	0.0976	N/A	0.8728	0.8935	0.1672	N/A	0.8716	0.8859	0.1233	N/A
Bombus_impatiens	invertebrate	0.8928	0.9104	0.7345	0.8080	0.9476	0.9576	0.9199	0.7422	0.9194	0.9334	0.8168	0.7737
Tribolium_castaneum	invertebrate	0.8610	0.8733	0.7488	0.8499	0.9186	0.9234	0.9299	0.8124	0.8889	0.8977	0.8296	0.8307
Drosophila_virilis	invertebrate	0.9038	0.9102	0.7056	N/A	0.9627	0.9674	0.9192	N/A	0.9324	0.9379	0.7983	N/A
Apis_dorsata	invertebrate	0.9079	0.9150	0.6698	0.7214	0.9590	0.9679	0.9419	0.7558	0.9328	0.9407	0.7829	0.7382
Frankliniella_occidentalis	invertebrate	0.7877	0.8037	0.5602	N/A	0.9226	0.9294	0.3636	N/A	0.8498	0.8620	0.4409	N/A
Trachymyrmex_cornetzi	invertebrate	0.7776	0.8452	0.2853	N/A	0.8985	0.8911	0.7536	N/A	0.8337	0.8676	0.4139	N/A
Drosophila_albomicans	invertebrate	0.8878	0.8915	0.7273	N/A	0.9679	0.9722	0.9261	N/A	0.9261	0.9301	0.8147	N/A
Ctenocephalides_felis	invertebrate	0.7028	0.7162	0.4768	N/A	0.8776	0.8774	0.9157	N/A	0.7805	0.7886	0.6271	N/A
Atta_cephalotes	invertebrate	0.9238	0.9306	0.5763	N/A	0.9488	0.9568	0.7768	N/A	0.9361	0.9435	0.6617	N/A
Rhopalosiphum_maidis	invertebrate	0.8828	0.8920	0.7092	N/A	0.9419	0.9457	0.8692	N/A	0.9114	0.9181	0.7810	N/A
Culex_pipiens	invertebrate	0.6913	0.7148	0.5067	0.7343	0.9307	0.9255	0.9082	0.8559	0.7934	0.8066	0.6505	0.7904
Hyposmocoma_kahamanoa	invertebrate	0.7057	0.7444	0.4439	N/A	0.8940	0.8937	0.9029	N/A	0.7888	0.8123	0.5952	N/A
Caenorhabditis_remanei	invertebrate	0.9092	0.9176	0.9481	N/A	0.8789	0.8909	0.9259	N/A	0.8938	0.9040	0.9369	N/A
Schistosoma_mansoni	invertebrate	0.7951	0.7821	0.8211	0.8781	0.7256	0.7397	0.8584	0.7761	0.7588	0.7603	0.8393	0.8240
Opisthorchis_viverrini	invertebrate	0.8288	0.8674	0.8576	N/A	0.4020	0.3918	0.6966	N/A	0.5414	0.5398	0.7687	N/A

Table S6: Subgenic F1 for test species.

species	group	Precision				Recall				F1			
		hel. BW	hel. post	genemark	augustus	hel. BW	hel. post	genemark	augustus	hel. BW	hel. post	genemark	augustus
Neurospora_crassa	fungi	0.9476	0.9603	0.9683	0.9800	0.9447	0.9433	0.9175	0.8075	0.9462	0.9517	0.9422	0.8854
Coccidioides_immitis	fungi	0.9527	0.9607	0.9714	0.9128	0.9070	0.9037	0.8792	0.8644	0.9293	0.9313	0.9230	0.8879
Fusarium_graminearum	fungi	0.9668	0.9681	0.9716	0.9674	0.9415	0.9395	0.9192	0.8735	0.9540	0.9536	0.9446	0.9181
Debaryomyces_hansenii	fungi	0.9966	0.9969	0.9959	0.9939	0.9909	0.9906	0.9802	0.9340	0.9937	0.9937	0.9880	0.9630
Aspergillus_terreus	fungi	0.9743	0.9738	0.9725	0.9729	0.9495	0.9483	0.9057	0.8899	0.9617	0.9609	0.9379	0.9296
Laccaria_bicolor	fungi	0.8940	0.8991	0.8971	0.8851	0.8214	0.8137	0.7667	0.7332	0.8562	0.8543	0.8268	0.8020
Papaver_somniferum	plant	0.3887	0.4368	0.2073	N/A	0.7894	0.7756	0.7315	N/A	0.5209	0.5589	0.3230	N/A
Arachis_hypogaea	plant	0.7977	0.8328	0.1779	N/A	0.7852	0.7755	0.6924	N/A	0.7914	0.8031	0.2831	N/A
Brassica_napus	plant	0.7678	0.7948	0.4029	N/A	0.8462	0.8379	0.8696	N/A	0.8051	0.8158	0.5507	N/A
Oryza_brachyantha	plant	0.8723	0.8867	0.2295	0.6441	0.9378	0.9360	0.3566	0.7991	0.9038	0.9107	0.2792	0.7133
Nicotiana_attenuata	plant	0.7522	0.8101	0.1333	0.4147	0.8076	0.7992	0.4741	0.6634	0.7789	0.8046	0.2081	0.5104
Solanum_pennellii	plant	0.8358	0.8596	0.3030	0.4155	0.8960	0.8918	0.6692	0.7719	0.8648	0.8754	0.4171	0.5402
Setaria_viridis	plant	0.8099	0.8306	0.1778	N/A	0.9042	0.9007	0.4082	N/A	0.8544	0.8642	0.2477	N/A
Vitis_riparia	plant	0.8993	0.9079	0.4499	N/A	0.8985	0.8932	0.6181	N/A	0.8989	0.9005	0.5207	N/A
Triticum_dicoccoides	plant	0.3794	0.4677	0.0276	0.2961	0.8087	0.7981	0.2784	0.5580	0.5165	0.5898	0.0503	0.3869
Phoenix_dactylifera	plant	0.8576	0.8707	0.1946	N/A	0.9111	0.9046	0.2690	N/A	0.8836	0.8873	0.2258	N/A
Coffea_arabica	plant	0.7930	0.8130	0.3687	N/A	0.8339	0.8277	0.7409	N/A	0.8129	0.8203	0.4924	N/A
Cannabis_sativa	plant	0.8741	0.8931	0.2490	N/A	0.7735	0.7662	0.6856	N/A	0.8207	0.8248	0.3653	N/A
Hibiscus_syriacus	plant	0.5568	0.5806	0.2568	N/A	0.7153	0.7098	0.6458	N/A	0.6262	0.6387	0.3675	N/A
Balaenoptera_musculus	vertebrate	0.7910	0.8719	0.2757	N/A	0.9452	0.9369	0.1185	N/A	0.8613	0.9032	0.1657	N/A
Xiphias_gladius	vertebrate	0.8720	0.8974	0.6800	N/A	0.9584	0.9531	0.6217	N/A	0.9132	0.9244	0.6495	N/A
Sparus_aurata	vertebrate	0.8580	0.8820	0.6176	N/A	0.9027	0.8943	0.5851	N/A	0.8797	0.8881	0.6009	N/A
Desmodus_rotundus	vertebrate	0.8307	0.8735	0.3228	N/A	0.9355	0.9272	0.1374	N/A	0.8800	0.8996	0.1927	N/A
Pseudonaja_textilis	vertebrate	0.8603	0.8805	0.2901	N/A	0.9189	0.9098	0.1380	N/A	0.8886	0.8949	0.1870	N/A
Falco_naumanni	vertebrate	0.7675	0.8350	0.2861	N/A	0.9631	0.9565	0.1450	N/A	0.8542	0.8916	0.1925	N/A
Chiroxiphia_lanceolata	vertebrate	0.8568	0.9017	0.1567	N/A	0.9469	0.9368	0.0496	N/A	0.8996	0.9189	0.0753	N/A
Myotis_davidii	vertebrate	0.8058	0.8400	0.2861	N/A	0.9101	0.9016	0.1437	N/A	0.8548	0.8697	0.1913	N/A
Lagopus_leucura	vertebrate	0.7983	0.8533	0.3684	N/A	0.9630	0.9568	0.0940	N/A	0.8730	0.9021	0.1498	N/A
Rhincodon_typus	vertebrate	0.7327	0.7857	0.1328	0.8165	0.9067	0.8902	0.0466	0.4332	0.8105	0.8347	0.0690	0.5661
Opisthocomus_hoazin	vertebrate	0.8246	0.8523	0.4399	N/A	0.9041	0.9004	0.2111	N/A	0.8625	0.8757	0.2853	N/A
Bombus_impatiens	invertebrate	0.8549	0.8833	0.6961	0.6721	0.8681	0.8432	0.3317	0.4489	0.8615	0.8628	0.4493	0.5383
Tribolium_castaneum	invertebrate	0.8736	0.8869	0.7136	0.6759	0.8547	0.8372	0.5338	0.6475	0.8640	0.8613	0.6107	0.6614
Drosophila_virilis	invertebrate	0.8471	0.8658	0.6052	N/A	0.8816	0.8638	0.4725	N/A	0.8640	0.8648	0.5307	N/A
Apis_dorsata	invertebrate	0.8298	0.8677	0.6017	0.6359	0.8616	0.8370	0.4055	0.3582	0.8454	0.8521	0.4845	0.4583
Frankliniella_occidentalis	invertebrate	0.7344	0.7714	0.4893	N/A	0.8693	0.8522	0.3505	N/A	0.7962	0.8097	0.4084	N/A
Trachymyrmex_cornetzi	invertebrate	0.8302	0.8670	0.3522	N/A	0.8078	0.7819	0.3874	N/A	0.8188	0.8223	0.3689	N/A
Drosophila_albomicans	invertebrate	0.8539	0.8684	0.6176	N/A	0.9021	0.8814	0.5182	N/A	0.8774	0.8748	0.5636	N/A
Ctenocephalides_felis	invertebrate	0.6529	0.7032	0.5736	N/A	0.8014	0.7793	0.3749	N/A	0.7196	0.7393	0.4535	N/A
Atta_cephalotes	invertebrate	0.9134	0.9268	0.6888	N/A	0.8336	0.8215	0.4352	N/A	0.8717	0.8710	0.5334	N/A
Rhopalosiphum_maidis	invertebrate	0.9146	0.9231	0.6695	N/A	0.8873	0.8691	0.4429	N/A	0.9008	0.8953	0.5331	N/A
Culex_pipiens	invertebrate	0.7610	0.8116	0.4724	0.6991	0.8535	0.8124	0.4253	0.4673	0.8046	0.8120	0.4476	0.5602
Hyposmocoma_kahamanoa	invertebrate	0.8429	0.8661	0.6238	N/A	0.7796	0.7563	0.4106	N/A	0.8100	0.8075	0.4953	N/A
Caenorhabditis_remanei	invertebrate	0.9304	0.9334	0.9466	N/A	0.8902	0.8792	0.9008	N/A	0.9098	0.9055	0.9231	N/A
Schistosoma_mansoni	invertebrate	0.9013	0.9065	0.9264	0.8991	0.7285	0.7089	0.7619	0.7918	0.8057	0.7956	0.8361	0.8421
Opisthorchis_viverrini	invertebrate	0.8728	0.8806	0.9242	N/A	0.3589	0.3333	0.4811	N/A	0.5086	0.4836	0.6328	N/A

Table S7: Genic F1 for test species.

species	group	Precision				Recall				F1			
		hel. BW	hel. post	genemark	augustus	hel. BW	hel. post	genemark	augustus	hel. BW	hel. post	genemark	augustus
Neurospora_crassa	fungi	0.9224	0.9342	0.9683	0.9800	0.8553	0.8539	0.6650	0.5853	0.8876	0.8922	0.7885	0.7329
Coccidioides_immitis	fungi	0.8877	0.8953	0.9714	0.9128	0.8676	0.8646	0.7074	0.6955	0.8775	0.8797	0.8186	0.7895
Fusarium_graminearum	fungi	0.9250	0.9267	0.9716	0.9674	0.9328	0.9307	0.8674	0.8243	0.9289	0.9287	0.9165	0.8902
Debaryomyces_hansenii	fungi	0.9944	0.9950	0.9959	0.9939	0.9909	0.9906	0.9802	0.9340	0.9926	0.9928	0.9880	0.9630
Aspergillus_terreus	fungi	0.9618	0.9615	0.9725	0.9729	0.9495	0.9483	0.9057	0.8899	0.9556	0.9549	0.9379	0.9296
Laccaria_bicolor	fungi	0.8611	0.8672	0.8971	0.8851	0.8145	0.8069	0.7483	0.7156	0.8372	0.8360	0.8160	0.7914
Papaver_somniferum	plant	0.3915	0.4381	0.2073	N/A	0.7715	0.7584	0.6425	N/A	0.5194	0.5554	0.3134	N/A
Arachis_hypogaea	plant	0.7696	0.8039	0.1779	N/A	0.7792	0.7690	0.6131	N/A	0.7744	0.7861	0.2758	N/A
Brassica_napus	plant	0.7496	0.7754	0.4029	N/A	0.7625	0.7551	0.6841	N/A	0.7560	0.7651	0.5072	N/A
Oryza_brachyantha	plant	0.8302	0.8454	0.2295	0.6380	0.9278	0.9252	0.3159	0.7388	0.8763	0.8835	0.2658	0.6847
Nicotiana_attenuata	plant	0.7366	0.7917	0.1333	0.4079	0.8048	0.7963	0.4354	0.6270	0.7692	0.7940	0.2041	0.4942
Solanum_pennellii	plant	0.8096	0.8322	0.3030	0.4137	0.8883	0.8837	0.6109	0.7217	0.8471	0.8572	0.4051	0.5259
Setaria_viridis	plant	0.7708	0.7921	0.1778	N/A	0.8861	0.8818	0.3509	N/A	0.8244	0.8346	0.2360	N/A
Vitis_riparia	plant	0.8495	0.8594	0.4499	N/A	0.8939	0.8879	0.5774	N/A	0.8712	0.8734	0.5057	N/A
Triticum_dicoccoides	plant	0.3678	0.4522	0.0276	0.2847	0.8013	0.7902	0.2499	0.5337	0.5042	0.5752	0.0498	0.3713
Phoenix_dactylifera	plant	0.8322	0.8460	0.1946	N/A	0.8956	0.8885	0.2495	N/A	0.8627	0.8667	0.2186	N/A
Coffea_arabica	plant	0.7541	0.7737	0.3687	N/A	0.8307	0.8239	0.6662	N/A	0.7905	0.7980	0.4747	N/A
Cannabis_sativa	plant	0.8431	0.8623	0.2490	N/A	0.7791	0.7715	0.6206	N/A	0.8098	0.8144	0.3554	N/A
Hibiscus_syriacus	plant	0.5179	0.5400	0.2568	N/A	0.7220	0.7162	0.6069	N/A	0.6032	0.6157	0.3609	N/A
Balaenoptera_musculus	vertebrate	0.7869	0.8658	0.2757	N/A	0.9405	0.9322	0.1159	N/A	0.8568	0.8978	0.1632	N/A
Xiphias_gladius	vertebrate	0.8494	0.8742	0.6800	N/A	0.9488	0.9435	0.5850	N/A	0.8964	0.9075	0.6289	N/A
Sparus_aurata	vertebrate	0.8455	0.8697	0.6176	N/A	0.8930	0.8847	0.5417	N/A	0.8686	0.8771	0.5772	N/A
Desmodus_rotundus	vertebrate	0.8262	0.8682	0.3228	N/A	0.9284	0.9202	0.1337	N/A	0.8743	0.8934	0.1891	N/A
Pseudonaja_textilis	vertebrate	0.8456	0.8657	0.2901	N/A	0.9148	0.9057	0.1347	N/A	0.8788	0.8852	0.1840	N/A
Falco_naumanni	vertebrate	0.7618	0.8267	0.2861	N/A	0.9523	0.9455	0.1386	N/A	0.8464	0.8821	0.1868	N/A
Chiroxiphia_lanceolata	vertebrate	0.8475	0.8907	0.1567	N/A	0.9395	0.9295	0.0475	N/A	0.8912	0.9097	0.0729	N/A
Myotis_davidii	vertebrate	0.7999	0.8338	0.2861	N/A	0.9063	0.8978	0.1407	N/A	0.8498	0.8646	0.1886	N/A
Lagopus_leucura	vertebrate	0.7896	0.8425	0.3684	N/A	0.9562	0.9499	0.0904	N/A	0.8650	0.8930	0.1451	N/A
Rhincodon_typus	vertebrate	0.7230	0.7758	0.1328	0.8165	0.9037	0.8872	0.0459	0.4268	0.8033	0.8278	0.0682	0.5606
Opisthocomus_hoazin	vertebrate	0.8115	0.8389	0.4399	N/A	0.9037	0.9000	0.2098	N/A	0.8551	0.8684	0.2841	N/A
Bombus_impatiens	invertebrate	0.8396	0.8670	0.6961	0.6721	0.8529	0.8284	0.3094	0.4187	0.8462	0.8472	0.4284	0.5160
Tribolium_castaneum	invertebrate	0.8600	0.8739	0.7136	0.6721	0.8424	0.8251	0.5075	0.6226	0.8511	0.8488	0.5931	0.6464
Drosophila_virilis	invertebrate	0.8285	0.8467	0.6052	N/A	0.8701	0.8529	0.4396	N/A	0.8488	0.8498	0.5093	N/A
Apis_dorsata	invertebrate	0.8156	0.8521	0.6017	0.6359	0.8521	0.8279	0.3885	0.3431	0.8335	0.8398	0.4721	0.4457
Frankliniella_occidentalis	invertebrate	0.7280	0.7629	0.4893	N/A	0.8584	0.8411	0.3191	N/A	0.7878	0.8001	0.3863	N/A
Trachymyrmex_cornetzi	invertebrate	0.8032	0.8381	0.3522	N/A	0.8062	0.7806	0.3739	N/A	0.8047	0.8083	0.3627	N/A
Drosophila_albomicans	invertebrate	0.8270	0.8412	0.6176	N/A	0.8934	0.8734	0.4829	N/A	0.8589	0.8570	0.5420	N/A
Ctenocephalides_felis	invertebrate	0.6446	0.6938	0.5736	N/A	0.8002	0.7783	0.3712	N/A	0.7140	0.7336	0.4507	N/A
Atta_cephalotes	invertebrate	0.9030	0.9170	0.6888	N/A	0.8332	0.8211	0.4334	N/A	0.8667	0.8664	0.5320	N/A
Rhopalosiphum_maidis	invertebrate	0.9031	0.9120	0.6695	N/A	0.8777	0.8598	0.4261	N/A	0.8902	0.8851	0.5208	N/A
Culex_pipiens	invertebrate	0.7472	0.7967	0.4724	0.6980	0.8476	0.8072	0.4109	0.4544	0.7942	0.8019	0.4395	0.5505
Hyposmocoma_kahamanoa	invertebrate	0.8294	0.8527	0.6238	N/A	0.7750	0.7519	0.4012	N/A	0.8013	0.7992	0.4884	N/A
Caenorhabditis_remanei	invertebrate	0.9181	0.9229	0.9466	N/A	0.8902	0.8792	0.9008	N/A	0.9040	0.9005	0.9231	N/A
Schistosoma_mansoni	invertebrate	0.8949	0.9008	0.9264	0.8965	0.7244	0.7049	0.7550	0.7857	0.8007	0.7909	0.8320	0.8374
Opisthorchis_viverrini	invertebrate	0.8684	0.8768	0.9242	N/A	0.3571	0.3316	0.4772	N/A	0.5060	0.4812	0.6294	N/A

Table S8: Gffcompare exon level results for test species. N/A indicates that an annotation was not possible with this species/tool combination.

species	group	precision				recall				F1			
		helixer post	genemark	augustus	augustus masked	helixer post	genemark	augustus	augustus masked	helixer post	genemark	augustus	augustus masked
Neurospora_crassa	fungi	0.799000	0.854000	0.756000	0.803000	0.811000	0.783000	0.634000	0.743000	0.804955	0.816960	0.689646	0.771836
Coccidioides_immitis	fungi	0.771000	0.853000	0.754000	0.761000	0.720000	0.708000	0.626000	0.634000	0.744628	0.773766	0.684064	0.691719
Fusarium_graminearum	fungi	0.608000	0.747000	0.732000	0.733000	0.605000	0.683000	0.635000	0.667000	0.606496	0.713568	0.680059	0.698444
Debaryomyces_hansenii	fungi	0.793000	0.843000	0.839000	0.887000	0.888000	0.850000	0.785000	0.856000	0.837816	0.846486	0.811102	0.871224
Aspergillus_terreus	fungi	0.538000	0.656000	0.653000	0.655000	0.623000	0.679000	0.659000	0.677000	0.577388	0.667302	0.655986	0.665818
Laccaria_bicolor	fungi	0.397000	0.529000	0.549000	0.581000	0.482000	0.523000	0.503000	0.464000	0.435390	0.525983	0.524994	0.515950
Papaver_somniferum	plant	0.304000	0.142000	NaN	NaN	0.789000	0.552000	NaN	NaN	0.438895	0.225890	NaN	NaN
Arachis_hypogaea	plant	0.703000	0.098000	NaN	NaN	0.813000	0.459000	NaN	NaN	0.754009	0.161515	NaN	NaN
Brassica_napus	plant	0.713000	0.379000	NaN	NaN	0.869000	0.806000	NaN	NaN	0.783308	0.515568	NaN	NaN
Oryza_brachyantha	plant	0.806000	0.015000	0.668000	0.713000	0.913000	0.035000	0.621000	0.655000	0.856170	0.021000	0.643643	0.682770
Nicotiana_attenuata	plant	0.641000	0.035000	0.323000	0.696000	0.790000	0.217000	0.635000	0.377000	0.707743	0.060278	0.428194	0.489081
Solanum_pennellii	plant	0.717000	0.136000	0.457000	0.717000	0.883000	0.570000	0.675000	0.731000	0.791389	0.219603	0.545009	0.723932
Setaria_viridis	plant	0.735000	0.020000	NaN	NaN	0.891000	0.058000	NaN	NaN	0.805517	0.029744	NaN	NaN
Vitis_riparia	plant	0.732000	0.205000	NaN	NaN	0.866000	0.571000	NaN	NaN	0.793382	0.301688	NaN	NaN
Triticum_dicoccoides	plant	0.340000	0.002000	0.303000	0.441000	0.850000	0.033000	0.591000	0.642000	0.485714	0.003771	0.400611	0.522848
Phoenix_dactylifera	plant	0.672000	0.007000	NaN	NaN	0.865000	0.028000	NaN	NaN	0.756383	0.011200	NaN	NaN
Coffea_arabica	plant	0.627000	0.201000	NaN	NaN	0.821000	0.536000	NaN	NaN	0.711004	0.292364	NaN	NaN
Cannabis_sativa	plant	0.810000	0.123000	NaN	NaN	0.831000	0.552000	NaN	NaN	0.820366	0.201173	NaN	NaN
Hibiscus_syriacus	plant	0.450000	0.199000	NaN	NaN	0.822000	0.691000	NaN	NaN	0.581604	0.309009	NaN	NaN
Balaenoptera_musculus	vertebrate	0.722000	0.001000	NaN	NaN	0.834000	0.008000	NaN	NaN	0.773969	0.001778	NaN	NaN
Xiphias_gladius	vertebrate	0.756000	0.441000	NaN	NaN	0.888000	0.749000	NaN	NaN	0.816701	0.555141	NaN	NaN
Sparus_aurata	vertebrate	0.713000	0.465000	NaN	NaN	0.887000	0.777000	NaN	NaN	0.790539	0.581812	NaN	NaN
Desmodus_rotundus	vertebrate	0.687000	0.010000	NaN	NaN	0.830000	0.038000	NaN	NaN	0.751760	0.015833	NaN	NaN
Pseudonaja_textilis	vertebrate	0.624000	0.005000	NaN	NaN	0.838000	0.015000	NaN	NaN	0.715338	0.007500	NaN	NaN
Falco_naumanni	vertebrate	0.714000	0.001000	NaN	NaN	0.865000	0.004000	NaN	NaN	0.782280	0.001600	NaN	NaN
Chiroxiphia_lanceolata	vertebrate	0.748000	0.000000	NaN	NaN	0.866000	0.000000	NaN	NaN	0.802686	0.000000	NaN	NaN
Myotis_davidii	vertebrate	0.557000	0.007000	NaN	NaN	0.780000	0.029000	NaN	NaN	0.649903	0.011278	NaN	NaN
Lagopus_leucura	vertebrate	0.734000	0.005000	NaN	NaN	0.874000	0.010000	NaN	NaN	0.797905	0.006667	NaN	NaN
Rhincodon_typus	vertebrate	0.483000	0.000000	0.564000	0.608000	0.768000	0.002000	0.473000	0.512000	0.593036	0.000000	0.514507	0.555886
Opisthocomus_hoazin	vertebrate	0.588000	0.003000	NaN	NaN	0.781000	0.011000	NaN	NaN	0.670896	0.004714	NaN	NaN
Bombus_impatiens	invertebrate	0.745000	0.606000	0.611000	0.709000	0.845000	0.754000	0.560000	0.690000	0.791855	0.671947	0.584389	0.699371
Tribolium_castaneum	invertebrate	0.487000	0.574000	0.639000	0.651000	0.561000	0.757000	0.571000	0.566000	0.521387	0.652920	0.603089	0.605532
Drosophila_virilis	invertebrate	0.783000	0.473000	NaN	NaN	0.862000	0.728000	NaN	NaN	0.820603	0.573429	NaN	NaN
Apis_dorsata	invertebrate	0.767000	0.481000	0.524000	0.523000	0.842000	0.778000	0.590000	0.734000	0.802752	0.594469	0.555045	0.610791
Frankliniella_occidentalis	invertebrate	0.690000	0.270000	NaN	NaN	0.845000	0.228000	NaN	NaN	0.759674	0.247229	NaN	NaN
Trachymyrmex_cornetzi	invertebrate	0.655000	0.107000	NaN	NaN	0.796000	0.423000	NaN	NaN	0.718649	0.170796	NaN	NaN
Drosophila_albomicans	invertebrate	0.749000	0.458000	NaN	NaN	0.859000	0.723000	NaN	NaN	0.800238	0.560769	NaN	NaN
Ctenocephalides_felis	invertebrate	0.537000	0.340000	NaN	NaN	0.802000	0.692000	NaN	NaN	0.643277	0.455969	NaN	NaN
Atta_cephalotes	invertebrate	0.692000	0.130000	NaN	NaN	0.819000	0.460000	NaN	NaN	0.750163	0.202712	NaN	NaN
Rhopalosiphum_maidis	invertebrate	0.760000	0.465000	NaN	NaN	0.884000	0.616000	NaN	NaN	0.817324	0.529954	NaN	NaN
Culex_pipiens	invertebrate	0.584000	0.177000	0.545000	0.628000	0.790000	0.592000	0.604000	0.622000	0.671557	0.272520	0.572985	0.624986
Hyposmocoma_kahamanoa	invertebrate	0.579000	0.394000	NaN	NaN	0.808000	0.711000	NaN	NaN	0.674596	0.507030	NaN	NaN
Caenorhabditis_remanei	invertebrate	0.408000	0.730000	NaN	NaN	0.501000	0.795000	NaN	NaN	0.449743	0.761115	NaN	NaN
Schistosoma_mansoni	invertebrate	0.361000	0.591000	0.646000	0.667000	0.463000	0.587000	0.522000	0.568000	0.405687	0.588993	0.577418	0.613532
Opisthorchis_viverrini	invertebrate	0.389000	0.583000	NaN	NaN	0.225000	0.467000	NaN	NaN	0.285098	0.518592	NaN	NaN

Table S9: Gffcompare intron level results for test species. N/A indicates that an annotation was not possible with this species/tool combination.

species	group	precision				recall				F1			
		helixer post	genemark	augustus	augustus masked	helixer post	genemark	augustus	augustus masked	helixer post	genemark	augustus	augustus masked
Neurospora_crassa	fungi	0.823000	0.895000	0.800000	0.827000	0.820000	0.800000	0.678000	0.786000	0.821497	0.844838	0.733965	0.805979
Coccidioides_immitis	fungi	0.802000	0.904000	0.781000	0.788000	0.741000	0.744000	0.697000	0.703000	0.770294	0.816233	0.736613	0.743077
Fusarium_graminearum	fungi	0.574000	0.775000	0.756000	0.753000	0.570000	0.701000	0.672000	0.700000	0.571993	0.736145	0.711529	0.725533
Debaryomyces_hansenii	fungi	0.292000	0.415000	0.492000	0.570000	0.719000	0.700000	0.764000	0.811000	0.415327	0.521076	0.598548	0.669471
Aspergillus_terreus	fungi	0.540000	0.701000	0.696000	0.694000	0.624000	0.724000	0.715000	0.729000	0.578969	0.712314	0.705372	0.711070
Laccaria_bicolor	fungi	0.442000	0.606000	0.616000	0.650000	0.522000	0.596000	0.587000	0.543000	0.478680	0.600958	0.601150	0.591702
Papaver_somniferum	plant	0.336000	0.152000	NaN	NaN	0.822000	0.581000	NaN	NaN	0.477016	0.240960	NaN	NaN
Arachis_hypogaea	plant	0.710000	0.103000	NaN	NaN	0.834000	0.486000	NaN	NaN	0.767021	0.169976	NaN	NaN
Brassica_napus	plant	0.742000	0.404000	NaN	NaN	0.900000	0.868000	NaN	NaN	0.813398	0.551371	NaN	NaN
Oryza_brachyantha	plant	0.815000	0.011000	0.657000	0.707000	0.911000	0.027000	0.618000	0.648000	0.860330	0.015632	0.636904	0.676215
Nicotiana_attenuata	plant	0.654000	0.032000	0.342000	0.711000	0.798000	0.205000	0.685000	0.403000	0.718860	0.055359	0.456222	0.514422
Solanum_pennellii	plant	0.712000	0.130000	0.474000	0.733000	0.871000	0.563000	0.732000	0.769000	0.783515	0.211227	0.575403	0.750569
Setaria_viridis	plant	0.755000	0.017000	NaN	NaN	0.897000	0.054000	NaN	NaN	0.819897	0.025859	NaN	NaN
Vitis_riparia	plant	0.717000	0.205000	NaN	NaN	0.851000	0.584000	NaN	NaN	0.778274	0.303473	NaN	NaN
Triticum_dicoccoides	plant	0.379000	0.002000	0.341000	0.492000	0.857000	0.023000	0.589000	0.641000	0.525571	0.003680	0.431933	0.556703
Phoenix_dactylifera	plant	0.634000	0.004000	NaN	NaN	0.827000	0.017000	NaN	NaN	0.717752	0.006476	NaN	NaN
Coffea_arabica	plant	0.619000	0.210000	NaN	NaN	0.816000	0.554000	NaN	NaN	0.703978	0.304555	NaN	NaN
Cannabis_sativa	plant	0.825000	0.125000	NaN	NaN	0.859000	0.586000	NaN	NaN	0.841657	0.206048	NaN	NaN
Hibiscus_syriacus	plant	0.461000	0.209000	NaN	NaN	0.836000	0.729000	NaN	NaN	0.594288	0.324864	NaN	NaN
Balaenoptera_musculus	vertebrate	0.671000	0.000000	NaN	NaN	0.787000	0.002000	NaN	NaN	0.724385	0.000000	NaN	NaN
Xiphias_gladius	vertebrate	0.721000	0.441000	NaN	NaN	0.851000	0.713000	NaN	NaN	0.780625	0.544945	NaN	NaN
Sparus_aurata	vertebrate	0.693000	0.476000	NaN	NaN	0.859000	0.754000	NaN	NaN	0.767122	0.583584	NaN	NaN
Desmodus_rotundus	vertebrate	0.633000	0.007000	NaN	NaN	0.776000	0.024000	NaN	NaN	0.697243	0.010839	NaN	NaN
Pseudonaja_textilis	vertebrate	0.577000	0.002000	NaN	NaN	0.773000	0.007000	NaN	NaN	0.660772	0.003111	NaN	NaN
Falco_naumanni	vertebrate	0.665000	0.000000	NaN	NaN	0.814000	0.001000	NaN	NaN	0.731995	0.000000	NaN	NaN
Chiroxiphia_lanceolata	vertebrate	0.706000	0.000000	NaN	NaN	0.823000	0.000000	NaN	NaN	0.760024	0.000000	NaN	NaN
Myotis_davidii	vertebrate	0.497000	0.004000	NaN	NaN	0.703000	0.016000	NaN	NaN	0.582318	0.006400	NaN	NaN
Lagopus_leucura	vertebrate	0.696000	0.003000	NaN	NaN	0.833000	0.006000	NaN	NaN	0.758362	0.004000	NaN	NaN
Rhincodon_typus	vertebrate	0.422000	0.000000	0.575000	0.628000	0.686000	0.000000	0.437000	0.488000	0.522549	0.000000	0.496591	0.549219
Opisthocomus_hoazin	vertebrate	0.540000	0.001000	NaN	NaN	0.723000	0.004000	NaN	NaN	0.618242	0.001600	NaN	NaN
Bombus_impatiens	invertebrate	0.739000	0.667000	0.662000	0.753000	0.832000	0.773000	0.573000	0.703000	0.782747	0.716099	0.614293	0.727141
Tribolium_castaneum	invertebrate	0.415000	0.616000	0.676000	0.688000	0.470000	0.787000	0.671000	0.667000	0.440791	0.691079	0.673491	0.677337
Drosophila_virilis	invertebrate	0.775000	0.487000	NaN	NaN	0.854000	0.752000	NaN	NaN	0.812584	0.591161	NaN	NaN
Apis_dorsata	invertebrate	0.784000	0.539000	0.595000	0.613000	0.852000	0.802000	0.599000	0.745000	0.816587	0.644710	0.596993	0.672585
Frankliniella_occidentalis	invertebrate	0.712000	0.284000	NaN	NaN	0.860000	0.231000	NaN	NaN	0.779033	0.254773	NaN	NaN
Trachymyrmex_cornetzi	invertebrate	0.659000	0.117000	NaN	NaN	0.788000	0.448000	NaN	NaN	0.717750	0.185543	NaN	NaN
Drosophila_albomicans	invertebrate	0.757000	0.470000	NaN	NaN	0.856000	0.750000	NaN	NaN	0.803462	0.577869	NaN	NaN
Ctenocephalides_felis	invertebrate	0.545000	0.377000	NaN	NaN	0.812000	0.729000	NaN	NaN	0.652233	0.496986	NaN	NaN
Atta_cephalotes	invertebrate	0.693000	0.144000	NaN	NaN	0.810000	0.479000	NaN	NaN	0.746946	0.221432	NaN	NaN
Rhopalosiphum_maidis	invertebrate	0.761000	0.521000	NaN	NaN	0.877000	0.655000	NaN	NaN	0.814893	0.580366	NaN	NaN
Culex_pipiens	invertebrate	0.589000	0.167000	0.565000	0.652000	0.771000	0.615000	0.649000	0.660000	0.667822	0.262673	0.604094	0.655976
Hyposmocoma_kahamanoa	invertebrate	0.536000	0.425000	NaN	NaN	0.737000	0.684000	NaN	NaN	0.620632	0.524256	NaN	NaN
Caenorhabditis_remanei	invertebrate	0.347000	0.782000	NaN	NaN	0.429000	0.872000	NaN	NaN	0.383668	0.824551	NaN	NaN
Schistosoma_mansoni	invertebrate	0.293000	0.617000	0.656000	0.682000	0.393000	0.611000	0.561000	0.608000	0.335711	0.613985	0.604792	0.642878
Opisthorchis_viverrini	invertebrate	0.327000	0.593000	NaN	NaN	0.201000	0.474000	NaN	NaN	0.248966	0.526864	NaN	NaN

Table S10: Gffcompare intron chain level results for test species. N/A indicates that an annotation was not possible with this species/tool combination.

species	group	precision				recall				F1			
		helixer post	genemark	augustus	augustus masked	helixer post	genemark	augustus	augustus masked	helixer post	genemark	augustus	augustus masked
Neurospora_crassa	fungi	0.740000	0.776000	0.629000	0.705000	0.723000	0.706000	0.502000	0.621000	0.731401	0.739347	0.558370	0.660339
Coccidioides_immitis	fungi	0.644000	0.729000	0.572000	0.583000	0.575000	0.587000	0.393000	0.405000	0.607547	0.650339	0.465898	0.477966
Fusarium_graminearum	fungi	0.405000	0.600000	0.585000	0.577000	0.396000	0.543000	0.481000	0.500000	0.400449	0.570079	0.527927	0.535747
Debaryomyces_hansenii	fungi	0.334000	0.413000	0.506000	0.567000	0.668000	0.633000	0.723000	0.789000	0.445333	0.499864	0.595343	0.659827
Aspergillus_terreus	fungi	0.335000	0.466000	0.470000	0.473000	0.362000	0.452000	0.427000	0.440000	0.347977	0.458893	0.447469	0.455904
Laccaria_bicolor	fungi	0.112000	0.208000	0.204000	0.224000	0.143000	0.207000	0.152000	0.144000	0.125616	0.207499	0.174202	0.175304
Papaver_somniferum	plant	0.154000	0.038000	NaN	NaN	0.450000	0.185000	NaN	NaN	0.229470	0.063049	NaN	NaN
Arachis_hypogaea	plant	0.465000	0.020000	NaN	NaN	0.510000	0.115000	NaN	NaN	0.486462	0.034074	NaN	NaN
Brassica_napus	plant	0.503000	0.177000	NaN	NaN	0.634000	0.413000	NaN	NaN	0.560953	0.247800	NaN	NaN
Oryza_brachyantha	plant	0.550000	0.003000	0.253000	0.289000	0.635000	0.005000	0.219000	0.258000	0.589451	0.003750	0.234775	0.272622
Nicotiana_attenuata	plant	0.366000	0.008000	0.111000	0.322000	0.424000	0.055000	0.221000	0.152000	0.392871	0.013968	0.147777	0.206515
Solanum_pennellii	plant	0.443000	0.037000	0.147000	0.333000	0.542000	0.150000	0.187000	0.300000	0.487525	0.059358	0.164605	0.315640
Setaria_viridis	plant	0.492000	0.005000	NaN	NaN	0.614000	0.013000	NaN	NaN	0.546271	0.007222	NaN	NaN
Vitis_riparia	plant	0.411000	0.041000	NaN	NaN	0.470000	0.122000	NaN	NaN	0.438524	0.061374	NaN	NaN
Triticum_dicoccoides	plant	0.187000	0.000000	0.121000	0.193000	0.552000	0.006000	0.284000	0.323000	0.279361	0.000000	0.169699	0.241624
Phoenix_dactylifera	plant	0.334000	0.001000	NaN	NaN	0.405000	0.003000	NaN	NaN	0.366089	0.001500	NaN	NaN
Coffea_arabica	plant	0.338000	0.049000	NaN	NaN	0.424000	0.152000	NaN	NaN	0.376147	0.074109	NaN	NaN
Cannabis_sativa	plant	0.569000	0.035000	NaN	NaN	0.545000	0.148000	NaN	NaN	0.556741	0.056612	NaN	NaN
Hibiscus_syriacus	plant	0.254000	0.061000	NaN	NaN	0.467000	0.239000	NaN	NaN	0.329037	0.097193	NaN	NaN
Balaenoptera_musculus	vertebrate	0.192000	0.000000	NaN	NaN	0.202000	0.000000	NaN	NaN	0.196873	0.000000	NaN	NaN
Xiphias_gladius	vertebrate	0.241000	0.037000	NaN	NaN	0.270000	0.095000	NaN	NaN	0.254677	0.053258	NaN	NaN
Sparus_aurata	vertebrate	0.243000	0.063000	NaN	NaN	0.309000	0.154000	NaN	NaN	0.272054	0.089419	NaN	NaN
Desmodus_rotundus	vertebrate	0.148000	0.000000	NaN	NaN	0.160000	0.001000	NaN	NaN	0.153766	0.000000	NaN	NaN
Pseudonaja_textilis	vertebrate	0.126000	0.000000	NaN	NaN	0.165000	0.000000	NaN	NaN	0.142887	0.000000	NaN	NaN
Falco_naumanni	vertebrate	0.200000	0.000000	NaN	NaN	0.220000	0.000000	NaN	NaN	0.209524	0.000000	NaN	NaN
Chiroxiphia_lanceolata	vertebrate	0.239000	0.000000	NaN	NaN	0.256000	0.000000	NaN	NaN	0.247208	0.000000	NaN	NaN
Myotis_davidii	vertebrate	0.067000	0.000000	NaN	NaN	0.086000	0.000000	NaN	NaN	0.075320	0.000000	NaN	NaN
Lagopus_leucura	vertebrate	0.216000	0.000000	NaN	NaN	0.244000	0.000000	NaN	NaN	0.229148	0.000000	NaN	NaN
Rhincodon_typus	vertebrate	0.061000	0.000000	0.061000	0.081000	0.082000	0.000000	0.071000	0.089000	0.069958	0.000000	0.065621	0.084812
Opisthocomus_hoazin	vertebrate	0.058000	0.000000	NaN	NaN	0.069000	0.000000	NaN	NaN	0.063024	0.000000	NaN	NaN
Bombus_impatiens	invertebrate	0.348000	0.189000	0.125000	0.238000	0.403000	0.313000	0.119000	0.240000	0.373486	0.235685	0.121926	0.238996
Tribolium_castaneum	invertebrate	0.127000	0.255000	0.205000	0.203000	0.152000	0.385000	0.098000	0.093000	0.138380	0.306797	0.132607	0.127561
Drosophila_virilis	invertebrate	0.562000	0.250000	NaN	NaN	0.606000	0.406000	NaN	NaN	0.583171	0.309451	NaN	NaN
Apis_dorsata	invertebrate	0.399000	0.140000	0.099000	0.155000	0.457000	0.339000	0.160000	0.338000	0.426035	0.198163	0.122317	0.212535
Frankliniella_occidentalis	invertebrate	0.313000	0.040000	NaN	NaN	0.402000	0.044000	NaN	NaN	0.351961	0.041905	NaN	NaN
Trachymyrmex_cornetzi	invertebrate	0.252000	0.009000	NaN	NaN	0.322000	0.046000	NaN	NaN	0.282732	0.015055	NaN	NaN
Drosophila_albomicans	invertebrate	0.524000	0.243000	NaN	NaN	0.610000	0.391000	NaN	NaN	0.563739	0.299726	NaN	NaN
Ctenocephalides_felis	invertebrate	0.197000	0.072000	NaN	NaN	0.306000	0.185000	NaN	NaN	0.239690	0.103658	NaN	NaN
Atta_cephalotes	invertebrate	0.282000	0.012000	NaN	NaN	0.350000	0.059000	NaN	NaN	0.312342	0.019944	NaN	NaN
Rhopalosiphum_maidis	invertebrate	0.378000	0.063000	NaN	NaN	0.463000	0.112000	NaN	NaN	0.416205	0.080640	NaN	NaN
Culex_pipiens	invertebrate	0.371000	0.081000	0.266000	0.336000	0.492000	0.213000	0.307000	0.361000	0.423017	0.117367	0.285033	0.348052
Hyposmocoma_kahamanoa	invertebrate	0.145000	0.057000	NaN	NaN	0.211000	0.159000	NaN	NaN	0.171882	0.083917	NaN	NaN
Caenorhabditis_remanei	invertebrate	0.088000	0.394000	NaN	NaN	0.103000	0.402000	NaN	NaN	0.094911	0.397960	NaN	NaN
Schistosoma_mansoni	invertebrate	0.021000	0.097000	0.100000	0.133000	0.022000	0.103000	0.061000	0.091000	0.021488	0.099910	0.075776	0.108063
Opisthorchis_viverrini	invertebrate	0.018000	0.070000	NaN	NaN	0.007000	0.054000	NaN	NaN	0.010080	0.060968	NaN	NaN

Table S11: Gffcompare transcript/gene level results for test species. N/A indicates that an annotation was not possible with this species/tool combination.

		precision				recall				F1			
species	group	helixer post	genemark	augustus	augustus masked	helixer post	genemark	augustus	augustus masked	helixer post	genemark	augustus	augustus masked
Neurospora_crassa	fungi	0.719000	0.769000	0.647000	0.733000	0.752000	0.733000	0.533000	0.650000	0.735130	0.750569	0.584493	0.689009
Coccidioides_immitis	fungi	0.624000	0.710000	0.607000	0.615000	0.596000	0.600000	0.423000	0.434000	0.609679	0.650382	0.498565	0.508885
Fusarium_graminearum	fungi	0.478000	0.631000	0.627000	0.623000	0.479000	0.588000	0.519000	0.547000	0.478499	0.608742	0.567911	0.582532
Debaryomyces_hansenii	fungi	0.874000	0.905000	0.908000	0.935000	0.891000	0.867000	0.808000	0.871000	0.882418	0.885593	0.855086	0.901866
Aspergillus_terreus	fungi	0.364000	0.473000	0.482000	0.485000	0.422000	0.491000	0.467000	0.482000	0.390860	0.481832	0.474381	0.483495
Laccaria_bicolor	fungi	0.117000	0.211000	0.210000	0.232000	0.160000	0.212000	0.159000	0.148000	0.135162	0.211499	0.180976	0.180716
Papaver_somniferum	plant	0.154000	0.064000	NaN	NaN	0.479000	0.263000	NaN	NaN	0.233068	0.102948	NaN	NaN
Arachis_hypogaea	plant	0.491000	0.040000	NaN	NaN	0.534000	0.186000	NaN	NaN	0.511598	0.065841	NaN	NaN
Brassica_napus	plant	0.522000	0.223000	NaN	NaN	0.646000	0.455000	NaN	NaN	0.577418	0.299307	NaN	NaN
Oryza_brachyantha	plant	0.579000	0.038000	0.355000	0.381000	0.695000	0.052000	0.313000	0.353000	0.631719	0.043911	0.332680	0.366466
Nicotiana_attenuata	plant	0.350000	0.021000	0.143000	0.378000	0.448000	0.119000	0.258000	0.170000	0.392982	0.035700	0.184010	0.234526
Solanum_pennellii	plant	0.463000	0.069000	0.191000	0.389000	0.588000	0.241000	0.226000	0.347000	0.518067	0.107284	0.207031	0.366802
Setaria_viridis	plant	0.510000	0.027000	NaN	NaN	0.664000	0.053000	NaN	NaN	0.576899	0.035775	NaN	NaN
Vitis_riparia	plant	0.457000	0.080000	NaN	NaN	0.532000	0.203000	NaN	NaN	0.491656	0.114770	NaN	NaN
Triticum_dicoccoides	plant	0.183000	0.006000	0.136000	0.207000	0.621000	0.052000	0.380000	0.424000	0.282694	0.010759	0.200310	0.278187
Phoenix_dactylifera	plant	0.392000	0.022000	NaN	NaN	0.475000	0.057000	NaN	NaN	0.429527	0.031747	NaN	NaN
Coffea_arabica	plant	0.378000	0.086000	NaN	NaN	0.480000	0.237000	NaN	NaN	0.422937	0.126204	NaN	NaN
Cannabis_sativa	plant	0.582000	0.064000	NaN	NaN	0.562000	0.232000	NaN	NaN	0.571825	0.100324	NaN	NaN
Hibiscus_syriacus	plant	0.278000	0.090000	NaN	NaN	0.522000	0.306000	NaN	NaN	0.362790	0.139091	NaN	NaN
Balaenoptera_musculus	vertebrate	0.226000	0.001000	NaN	NaN	0.224000	0.007000	NaN	NaN	0.224996	0.001750	NaN	NaN
Xiphias_gladius	vertebrate	0.261000	0.046000	NaN	NaN	0.292000	0.115000	NaN	NaN	0.275631	0.065714	NaN	NaN
Sparus_aurata	vertebrate	0.259000	0.072000	NaN	NaN	0.328000	0.177000	NaN	NaN	0.289445	0.102361	NaN	NaN
Desmodus_rotundus	vertebrate	0.182000	0.003000	NaN	NaN	0.190000	0.013000	NaN	NaN	0.185914	0.004875	NaN	NaN
Pseudonaja_textilis	vertebrate	0.142000	0.002000	NaN	NaN	0.197000	0.008000	NaN	NaN	0.165038	0.003200	NaN	NaN
Falco_naumanni	vertebrate	0.222000	0.000000	NaN	NaN	0.243000	0.001000	NaN	NaN	0.232026	0.000000	NaN	NaN
Chiroxiphia_lanceolata	vertebrate	0.262000	0.000000	NaN	NaN	0.280000	0.000000	NaN	NaN	0.270701	0.000000	NaN	NaN
Myotis_davidii	vertebrate	0.094000	0.002000	NaN	NaN	0.120000	0.009000	NaN	NaN	0.105421	0.003273	NaN	NaN
Lagopus_leucura	vertebrate	0.235000	0.001000	NaN	NaN	0.266000	0.003000	NaN	NaN	0.249541	0.001500	NaN	NaN
Rhincodon_typus	vertebrate	0.079000	0.003000	0.071000	0.090000	0.107000	0.008000	0.098000	0.117000	0.090892	0.004364	0.082343	0.101739
Opisthocomus_hoazin	vertebrate	0.077000	0.000000	NaN	NaN	0.094000	0.002000	NaN	NaN	0.084655	0.000000	NaN	NaN
Bombus_impatiens	invertebrate	0.350000	0.188000	0.116000	0.217000	0.414000	0.327000	0.140000	0.260000	0.379319	0.238742	0.126875	0.236562
Tribolium_castaneum	invertebrate	0.158000	0.268000	0.230000	0.228000	0.195000	0.404000	0.109000	0.102000	0.174561	0.322238	0.147906	0.140945
Drosophila_virilis	invertebrate	0.581000	0.298000	NaN	NaN	0.638000	0.456000	NaN	NaN	0.608167	0.360446	NaN	NaN
Apis_dorsata	invertebrate	0.404000	0.146000	0.096000	0.141000	0.469000	0.350000	0.177000	0.357000	0.434080	0.206048	0.124484	0.202157
Frankliniella_occidentalis	invertebrate	0.306000	0.043000	NaN	NaN	0.406000	0.044000	NaN	NaN	0.348978	0.043494	NaN	NaN
Trachymyrmex_cornetzi	invertebrate	0.245000	0.013000	NaN	NaN	0.326000	0.061000	NaN	NaN	0.279755	0.021432	NaN	NaN
Drosophila_albomicans	invertebrate	0.535000	0.287000	NaN	NaN	0.639000	0.437000	NaN	NaN	0.582394	0.346461	NaN	NaN
Ctenocephalides_felis	invertebrate	0.212000	0.093000	NaN	NaN	0.322000	0.241000	NaN	NaN	0.255670	0.134210	NaN	NaN
Atta_cephalotes	invertebrate	0.287000	0.016000	NaN	NaN	0.367000	0.075000	NaN	NaN	0.322107	0.026374	NaN	NaN
Rhopalosiphum_maidis	invertebrate	0.386000	0.084000	NaN	NaN	0.478000	0.148000	NaN	NaN	0.427102	0.107172	NaN	NaN
Culex_pipiens	invertebrate	0.343000	0.100000	0.267000	0.336000	0.509000	0.239000	0.266000	0.311000	0.409829	0.141003	0.266499	0.323017
Hyposmocoma_kahamanoa	invertebrate	0.165000	0.073000	NaN	NaN	0.247000	0.213000	NaN	NaN	0.197840	0.108734	NaN	NaN
Caenorhabditis_remanei	invertebrate	0.106000	0.401000	NaN	NaN	0.126000	0.395000	NaN	NaN	0.115138	0.397977	NaN	NaN
Schistosoma_mansoni	invertebrate	0.035000	0.116000	0.128000	0.165000	0.034000	0.118000	0.070000	0.105000	0.034493	0.116991	0.090505	0.128333
Opisthorchis_viverrini	invertebrate	0.050000	0.117000	NaN	NaN	0.021000	0.093000	NaN	NaN	0.029577	0.103629	NaN	NaN

Table S12: Fraction of complete BUSCOs for test species across the reference annotation and gene calling tools. N/A indicates that an annotation was not possible with this species/tool combination.

species	group	reference	helixer_post	genemark	augustus
Neurospora_crassa	fungi	1.000000	0.998681	1.000000	0.984169
Coccidioides_immitis	fungi	0.996042	0.997361	0.990765	0.967018
Fusarium_graminearum	fungi	0.982850	0.994723	0.990765	0.990765
Debaryomyces_hansenii	fungi	0.978892	0.982850	0.973615	0.961741
Aspergillus_terreus	fungi	0.932718	0.996042	0.990765	0.980211
Laccaria_bicolor	fungi	0.905013	0.972296	0.963061	0.912929
Papaver_somniferum	plant	1.000000	0.997647	0.851765	N/A
Arachis_hypogaea	plant	1.000000	0.992941	0.738824	N/A
Brassica_napus	plant	0.997647	0.997647	0.995294	N/A
Oryza_brachyantha	plant	0.997647	0.997647	0.098824	0.720000
Nicotiana_attenuata	plant	0.997647	0.971765	0.296471	0.844706
Solanum_pennellii	plant	0.995294	0.978824	0.743529	0.868235
Setaria_viridis	plant	0.995294	0.995294	0.131765	N/A
Vitis_riparia	plant	0.995294	0.952941	0.687059	N/A
Triticum_dicoccoides	plant	0.990588	0.992941	0.093723	0.736471
Phoenix_dactylifera	plant	0.985882	0.945882	0.103529	N/A
Coffea_arabica	plant	0.983529	0.960000	0.771765	N/A
Cannabis_sativa	plant	0.971765	0.957647	0.776471	N/A
Hibiscus_syriacus	plant	0.964706	0.962353	0.931765	N/A
Balaenoptera_musculus	vertebrate	0.997904	0.961216	0.105870	N/A
Xiphias_gladius	vertebrate	0.991614	0.977987	0.905660	N/A
Sparus_aurata	vertebrate	0.991614	0.959119	0.920335	N/A
Desmodus_rotundus	vertebrate	0.988470	0.959119	0.164570	N/A
Pseudonaja_textilis	vertebrate	0.980084	0.930818	0.078616	N/A
Falco_naumanni	vertebrate	0.952830	0.916143	0.066038	N/A
Chiroxiphia_lanceolata	vertebrate	0.946541	0.920335	0.000000	N/A
Myotis_davidii	vertebrate	0.943396	0.903564	0.162474	N/A
Lagopus_leucura	vertebrate	0.937107	0.903564	0.085954	N/A
Rhincodon_typus	vertebrate	0.857442	0.784067	0.011530	0.578616
Opisthocomus_hoazin	vertebrate	0.779874	0.734801	0.035639	N/A
Bombus_impatiens	invertebrate	0.996855	0.986373	0.950734	0.706499
Tribolium_castaneum	invertebrate	0.994759	0.903564	0.938155	0.765199
Drosophila_virilis	invertebrate	0.993711	0.977987	0.932914	N/A
Apis_dorsata	invertebrate	0.992662	0.985325	0.970650	0.723270
Frankliniella_occidentalis	invertebrate	0.989518	0.973795	0.029350	N/A
Trachymyrmex_cornetzi	invertebrate	0.987421	0.961216	0.658281	N/A
Drosophila_albomicans	invertebrate	0.973795	0.961216	0.930818	N/A
Ctenocephalides_felis	invertebrate	0.969602	0.942348	0.909853	N/A
Atta_cephalotes	invertebrate	0.968553	0.939203	0.661426	N/A
Rhopalosiphum_maidis	invertebrate	0.967505	0.943396	0.828092	N/A
Culex_pipiens	invertebrate	0.963312	0.942348	0.921384	0.910901
Hyposmocoma_kahamanoa	invertebrate	0.923480	0.829140	0.831237	N/A
Caenorhabditis_remanei	invertebrate	0.754717	0.711740	0.729560	N/A
Schistosoma_mansoni	invertebrate	0.709644	0.396226	0.533543	0.459119
Opisthorchis_viverrini	invertebrate	0.647799	0.179245	0.573375	N/A

Table S13: Feature F1 statistics for three test species, at exon, intron, intron chain and transcript levels, summarized as the mean value per group and level.

species	group	precision				recall				F1			
		helixer vertebrate	helixer mammal	tiberius	tiberius softmasked	helixer vertebrate	helixer mammal	tiberius	tiberius softmasked	helixer vertebrate	helixer mammal	tiberius	tiberius softmasked
Exon													
Bos_taurus	mammal	0.671	0.728	0.858	**0.875**	0.821	0.855	0.857	**0.869**	0.738	0.786	0.857	**0.872**
Delphinapterus_leucas	mammal	0.711	0.770	0.899	**0.909**	0.834	0.854	0.857	**0.865**	0.768	0.810	0.877	**0.886**
Homo_sapiens	mammal	0.673	0.730	0.866	**0.879**	0.840	0.867	**0.872**	0.870	0.747	0.793	0.869	**0.874**
Intron													
Bos_taurus	mammal	0.616	0.692	0.870	**0.885**	0.774	0.823	0.862	**0.877**	0.686	0.752	0.866	**0.881**
Delphinapterus_leucas	mammal	0.660	0.737	0.909	**0.919**	0.786	0.822	0.863	**0.872**	0.718	0.777	0.885	**0.895**
Homo_sapiens	mammal	0.624	0.697	0.876	**0.888**	0.797	0.836	**0.876**	0.874	0.700	0.760	0.876	**0.881**
Intron Chain													
Bos_taurus	mammal	0.174	0.256	0.460	**0.510**	0.179	0.274	0.463	**0.513**	0.176	0.265	0.461	**0.511**
Delphinapterus_leucas	mammal	0.184	0.257	0.509	**0.539**	0.192	0.269	0.495	**0.525**	0.188	0.263	0.502	**0.532**
Homo_sapiens	mammal	0.176	0.244	0.473	**0.495**	0.198	0.282	0.512	**0.533**	0.186	0.262	0.492	**0.513**
Transcript/Gene													
Bos_taurus	mammal	0.217	0.308	0.472	**0.534**	0.208	0.330	0.501	**0.539**	0.212	0.319	0.486	**0.536**
Delphinapterus_leucas	mammal	0.212	0.281	0.505	**0.544**	0.215	0.291	0.503	**0.529**	0.213	0.286	0.504	**0.536**
Homo_sapiens	mammal	0.210	0.283	0.478	**0.512**	0.212	0.308	0.507	**0.523**	0.211	0.295	0.492	**0.517**

Table S14: Mapman4 annotation statistics (from the Mercator functional annotation tool) for test plant species. N/A indicates that an annotation was not possible with this species/tool combination.

%Annotated	%Classified	%Bins occupied	harmonic	annotation	species
70.91	11.06	88.67	78.80	genemark	Arachis_hypogaea
85.23	27.86	96.63	90.57	genemark	Brassica_napus
77.29	13.67	86.79	81.76	genemark	Cannabis_sativa
76.39	20.08	87.64	81.63	genemark	Coffea_arabica
87.02	25.66	95.54	91.08	genemark	Hibiscus_syriacus
77.68	6.10	67.66	72.32	genemark	Nicotiana_attenuata
54.23	18.89	31.45	39.81	genemark	Oryza_brachyantha
75.20	12.13	90.30	82.06	genemark	Papaver_somniferum
39.62	9.74	32.30	35.59	genemark	Phoenix_dactylifera
49.81	14.22	36.69	42.26	genemark	Setaria_viridis
86.60	15.71	87.72	87.16	genemark	Solanum_pennellii
3.27	3.27	34.40	5.97	genemark	Triticum_dicoccoides
82.93	24.62	88.79	85.76	genemark	Vitis_riparia
92.82	48.92	97.11	94.92	helixer	Arachis_hypogaea
92.70	47.74	97.75	95.16	helixer	Brassica_napus
94.73	54.03	93.58	94.15	helixer	Cannabis_sativa
89.91	46.45	95.75	92.74	helixer	Coffea_arabica
93.49	46.66	96.75	95.09	helixer	Hibiscus_syriacus
86.37	38.39	96.25	91.04	helixer	Nicotiana_attenuata
89.48	52.68	94.66	92.00	helixer	Oryza_brachyantha
68.48	17.53	96.54	80.12	helixer	Papaver_somniferum
90.41	52.86	95.18	92.73	helixer	Phoenix_dactylifera
86.45	46.06	95.02	90.53	helixer	Setaria_viridis
90.69	48.44	95.83	93.19	helixer	Solanum_pennellii
71.53	18.53	94.88	81.57	helixer	Triticum_dicoccoides
94.60	53.13	96.23	95.41	helixer	Vitis_riparia
96.50	50.73	94.67	95.58	reference	Arachis_hypogaea
97.62	53.49	97.94	97.78	reference	Brassica_napus
98.35	50.31	91.65	94.88	reference	Cannabis_sativa
97.27	52.55	92.24	94.69	reference	Coffea_arabica
98.96	59.32	95.37	97.13	reference	Hibiscus_syriacus
93.48	46.75	94.05	93.76	reference	Nicotiana_attenuata
96.43	60.15	91.44	93.87	reference	Oryza_brachyantha
93.11	44.73	94.40	93.75	reference	Papaver_somniferum
97.93	60.07	88.50	92.98	reference	Phoenix_dactylifera
95.70	55.63	90.92	93.25	reference	Setaria_viridis
97.68	57.33	93.33	95.46	reference	Solanum_pennellii
93.94	52.39	93.55	93.74	reference	Triticum_dicoccoides
98.59	57.53	92.69	95.55	reference	Vitis_riparia

S3.2 Data

S3.2.1 Full Datasets

Table S15: Number of species used for model creation (training/validation) and for model evaluation (testing).

Split	Fungi	Invertebrates	Plants	Vertebrates
training/validation	298	201	77	314
testing (used)	6	15	13	11

Table S16: Configuration and metadata used for the automated training species selection process.

	fungi	plants	vertebrates	invertebrates
total species	298	77	314	201
seeds per split	32	12	24	24
training species (per split)	64	16	16	16

Table S17: Overview of assessed fungal species, including their scientific names, identification, and version. The "Split" column indicates whether each species was designated for model training/validation or model evaluation purposes.

scientific	ID	version	split
Aaosphaeria arxii	Aaosphaeria_arxii	GCF_010015735.1_Aaoar1	train_val
Acaromyces ingoldii	Acaromyces_ingoldii	GCF_003144295.1_Acain1	test
Agaricus bisporus	Agaricus_bisporus	GCF_000300575.1_Agabi_varbisH97_2	train_val
Alternaria alternata	Alternaria_alternata	GCF_001642055.1_Altal1	test
Alternaria arborescens	Alternaria_arborescens	GCF_004154835.1_ASM415483v1	train_val
Alternaria atra	Alternaria_atra	GCF_907166805.1_ALTATR162	train_val
Alternaria burnsii	Alternaria_burnsii	GCF_013036055.1_ASM1303605v1	train_val
Alternaria rosae	Alternaria_rosae	GCF_020736505.1_Altro1	train_val
Amorphotheca resinae	Amorphotheca_resinae	GCF_003019875.1_Amore1	train_val
Apiotrichum porosum	Apiotrichum_porosum	GCF_003942205.1_ASM394220v1	train_val
Aplosporella prunicola	Aplosporella_prunicola	GCF_010093885.1_Aplpr1	test
Arthroderma uncinatum	Arthroderma_uncinatum	GCF_011692745.1_ASM1169274v1	test
Ascochyta rabiei	Ascochyta_rabiei	GCF_004011695.1_Arabiei_Me14	train_val
Ascoidea rubescens	Ascoidea_rubescens	GCF_001661345.1_Ascru1	train_val
Aspergillus aculeatinus	Aspergillus_aculeatinus	GCF_003184765.1_Aspacu1	train_val
Aspergillus aculeatus	Aspergillus_aculeatus	GCF_001890905.1_Aspac1	train_val
Aspergillus alliaceus	Aspergillus_alliaceus	GCF_009176365.1_Aspalli1	train_val
Aspergillus bombycis	Aspergillus_bombycis	GCF_001792695.1_ASM179269v1	train_val
Aspergillus brunneoviolaceus	Aspergillus_brunneoviolaceus	GCF_003184695.1_Aspbru1	train_val
Aspergillus caelatus	Aspergillus_caelatus	GCF_009193585.1_Aspcae1	train_val
Aspergillus campestris	Aspergillus_campestris	GCF_002847485.1_Aspcam1	train_val
Aspergillus candidus	Aspergillus_candidus	GCF_002847045.1_Aspcand1	train_val
Aspergillus chevalieri	Aspergillus_chevalieri	GCF_016861735.1_AchevalieriM1_assembly01	train_val
Aspergillus clavatus	Aspergillus_clavatus	GCF_000002715.2_ASM271v1	train_val
Aspergillus costaricensis	Aspergillus_costaricensis	GCF_003184835.1_Aspcos1	test
Aspergillus eucalypticola	Aspergillus_eucalypticola	GCF_003184535.1_Aspeuc1	test
Aspergillus fijiensis	Aspergillus_fijiensis	GCF_003184825.1_Aspfij1	test
Aspergillus fischeri	Aspergillus_fischeri	GCF_000149645.2_ASM14964v3	test
Aspergillus flavus	Aspergillus_flavus	GCF_014117465.1_ASM1411746v1	train_val
Aspergillus fumigatus	Aspergillus_fumigatus	GCF_000002655.1_ASM265v1	train_val
Aspergillus glaucus	Aspergillus_glaucus	GCF_001890805.1_Aspgl1	test
Aspergillus heteromorphus	Aspergillus_heteromorphus	GCF_003184545.1_Asphet1	train_val
Aspergillus homomorphus	Aspergillus_homomorphus	GCF_003184865.1_Asphom1	train_val
Aspergillus ibericus	Aspergillus_ibericus	GCF_003184845.1_Aspibe1	train_val
Aspergillus japonicus	Aspergillus_japonicus	GCF_003184785.1_Aspjap1	train_val
Aspergillus lentulus	Aspergillus_lentulus	GCF_010724455.1_ASM1072445v1	test
Aspergillus luchuensis	Aspergillus_luchuensis	GCF_016861625.1_AkawachiiIFO4308_assembly01	train_val
Aspergillus melleus	Aspergillus_melleus	GCF_016097325.1_ASM1609732v1	train_val
Aspergillus mulundensis	Aspergillus_mulundensis	GCF_003369625.1_ASM336962v1	test
Aspergillus neoniger	Aspergillus_neoniger	GCF_003184625.1_Aspneo1	test
Aspergillus nidulans	Aspergillus_nidulans	GCF_000149205.2_ASM14920v2	train_val
Aspergillus niger	Aspergillus_niger	GCF_000002855.3_ASM285v2	test
Aspergillus nomiae	Aspergillus_nomiae	GCF_001204775.2_ASM120477v2	train_val
Aspergillus novofumigatus	Aspergillus_novofumigatus	GCF_002847465.1_Aspnov1	train_val
Aspergillus ochraceoroseus	Aspergillus_ochraceoroseus	GCF_002846915.1_Aspergillus_ochraceoroseus_IBT_...	test
Aspergillus oryzae	Aspergillus_oryzae	GCF_000184455.2_ASM18445v3	train_val
Aspergillus piperis	Aspergillus_piperis	GCF_003184755.1_Asppip1	test
Aspergillus pseudonomiae	Aspergillus_pseudonomiae	GCF_009193645.1_Asppsen1	train_val
Aspergillus pseudotamarii	Aspergillus_pseudotamarii	GCF_009193445.1_Asppset1	train_val
Aspergillus pseudoviridinutans	Aspergillus_pseudoviridinutans	GCF_018340605.1_Asppvi_assembly01	train_val
Aspergillus puulaauensis	Aspergillus_puulaauensis	GCF_016861865.1_ApuulaauensisMK2_assembly01	test
Aspergillus ruber	Aspergillus_ruber	GCF_000600275.1_Eurhe1	train_val
Aspergillus saccharolyticus	Aspergillus_saccharolyticus	GCF_003184585.1_Aspsac1	test
Aspergillus sclerotioniger	Aspergillus_sclerotioniger	GCF_003184525.1_Aspscl1	train_val
Aspergillus steynii	Aspergillus_steynii	GCF_002849105.1_Aspste1	train_val
Aspergillus sydowii	Aspergillus_sydowii	GCF_001890705.1_Aspsy1	test
Aspergillus tanneri	Aspergillus_tanneri	GCF_003426965.1_ASM342696v1	train_val
Aspergillus terreus	Aspergillus_terreus	GCF_000149615.1_ASM14961v1	test (used)
Aspergillus thermomutatus	Aspergillus_thermomutatus	GCF_002237265.1_ASM223726v2	train_val
Aspergillus tubingensis	Aspergillus_tubingensis	GCF_013340325.1_ASM1334032v1	train_val
Aspergillus udagawae	Aspergillus_udagawae	GCF_001078395.1_Aud_assembly02	train_val
Aspergillus uvarum	Aspergillus_uvarum	GCF_003184745.1_Aspuva1	train_val
Aspergillus vadensis	Aspergillus_vadensis	GCF_003184925.1_Aspvad1	train_val
Aspergillus versicolor	Aspergillus_versicolor	GCF_001890125.1_Aspve1	train_val
Aspergillus viridinutans	Aspergillus_viridinutans	GCF_018404265.1_Aspvir_assembly01	train_val
Aspergillus welwitschiae	Aspergillus_welwitschiae	GCF_003344945.1_Aspwel1	train_val
Aspergillus wentii	Aspergillus_wentii	GCF_001890725.1_Aspwe1	train_val
Aureobasidium melanogenum	Aureobasidium_melanogenum	GCF_000721775.1_Aureobasidium_pullulans_var._me...	test
Aureobasidium namibiae	Aureobasidium_namibiae	GCF_000721765.1_Aureobasidium_pullulans_var._na...	train_val
Aureobasidium pullulans	Aureobasidium_pullulans	GCF_000721785.1_Aureobasidium_pullulans_var._pu...	train_val
Aureobasidium subglaciale	Aureobasidium_subglaciale	GCF_000721755.1_Aureobasidium_pullulans_var._su...	train_val
Babjeviella inositovora	Babjeviella_inositovora	GCF_001661335.1_Babin1	train_val
Bacidia gigantensis	Bacidia_gigantensis	GCF_019456465.1_ASM1945646v1	train_val
Batrachochytrium dendrobatidis	Batrachochytrium_dendrobatidis	GCF_000203795.1_v1.0	train_val
Baudoinia panamericana	Baudoinia_panamericana	GCF_000338955.1_Bauco1	test
Beauveria bassiana	Beauveria_bassiana	GCF_000280675.1_ASM28067v1	train_val
Bipolaris maydis	Bipolaris_maydis	GCF_000354255.1_CocheC4_1	train_val
Bipolaris oryzae	Bipolaris_oryzae	GCF_000523455.1_Cochliobolus_miyabeanus_v1.0	train_val
Bipolaris sorokiniana	Bipolaris_sorokiniana	GCF_000338995.1_Cocsa1	train_val
Bipolaris victoriae	Bipolaris_victoriae	GCF_000527765.1_Cochliobolus_victoriae_v1.0	train_val

scientific	ID	version	split
Bipolaris zeicola	Bipolaris_zeicola	GCF_000523435.1_Cochliobolus_carbonum_v1.0	test
Blastomyces dermatitidis	Blastomyces_dermatitidis	GCF_000003525.1_BD_ER3_V1	train_val
Blastomyces gilchristii	Blastomyces_gilchristii	GCF_000003855.2_BD_SLH14081_V1	train_val
Boeremia exigua	Boeremia_exigua	GCF_020726555.1_Boeex1	train_val
Botrytis byssoidea	Botrytis_byssoidea	GCF_014898295.1_ASM1489829v1	test
Botrytis cinerea	Botrytis_cinerea	GCF_000143535.2_ASM14353v4	train_val
Botrytis deweyae	Botrytis_deweyae	GCF_014898535.1_ASM1489853v1	test
Botrytis fragariae	Botrytis_fragariae	GCF_013461495.1_Bfra_R1V1	train_val
Botrytis porri	Botrytis_porri	GCF_014898465.1_ASM1489846v1	train_val
Botrytis sinoallii	Botrytis_sinoallii	GCF_014898435.1_ASM1489843v1	test
Brettanomyces bruxellensis	Brettanomyces_bruxellensis	GCF_011074885.1_ASM1107488v2	test
Brettanomyces nanus	Brettanomyces_nanus	GCF_011074865.1_ASM1107486v2	train_val
Candida albicans	Candida_albicans	GCF_000182965.3_ASM18296v3	train_val
Candida auris	_Candida_auris	GCF_002775015.1_Cand_auris_B11221_V1	train_val
Candida dubliniensis	Candida_dubliniensis	GCF_000026945.1_ASM2694v1	train_val
Candida duobushaemulonis	_Candida_duobushaemulonis	GCF_002926085.2_CanDuoHae_v1.0	test
Candida glabrata	_Candida_glabrata	GCF_000002545.3_ASM254v2	test
Candida haemuloni	_Candida_haemuloni	GCF_002926055.2_CanHae_1.0	train_val
Candida orthopsilosis	Candida_orthopsilosis	GCF_000315875.1_ASM31587v1	train_val
Candida parapsilosis	Candida_parapsilosis	GCF_000182765.1_ASM18276v2	train_val
Candida pseudohaemulonii	_Candida_pseudohaemulonii	GCF_003013735.1_Cand_pseudohaemulonii_B12108	test
Candida tropicalis	Candida_tropicalis	GCF_000006335.3_ASM633v3	train_val
Cantharellus anzutake	Cantharellus_anzutake	GCF_015039405.1_Cananz1	train_val
Capronia coronata	Capronia_coronata	GCF_000585585.1_Capr_coro_CBS_617_96_V1	train_val
Capronia epimyces	Capronia_epimyces	GCF_000585565.1_Capr_epim_CBS_606_96_V1	train_val
Ceraceosorus guamensis	Ceraceosorus_guamensis	GCF_003144195.1_Cersp1	train_val
Cercospora beticola	Cercospora_beticola	GCF_002742065.1_CB0940_V2	test
Cercospora kikuchii	Cercospora_kikuchii	GCF_019650295.1_Ck_assembly01	test
Chaetomium globosum	Chaetomium_globosum	GCF_000143365.1_ASM14336v1	train_val
Chaetomium thermophilum	Chaetomium_thermophilum	GCF_000221225.1_CTHT_3.0	train_val
Cladophialophora bantiana	Cladophialophora_bantiana	GCF_000835475.1_Clad_bant_CBS_173_52_V1	test
Cladophialophora carrionii	Cladophialophora_carrionii	GCF_000365165.1_Clad_carr_CBS_160_54_V1	train_val
Cladophialophora immunda	Cladophialophora_immunda	GCF_000835495.1_Clad_immu_CBS83496_V1	test
Cladophialophora psammophila	Cladophialophora_psammophila	GCF_000585535.1_Clad_psam_CBS_110553_V1	test
Cladophialophora yegresii	Cladophialophora_yegresii	GCF_000585515.1_Clad_yegr_CBS_114405_V1	test
Clavispora lusitaniae	Clavispora_lusitaniae	GCF_000003835.1_ASM383v1	train_val
Coccidioides immitis	Coccidioides_immitis	GCF_000149335.2_ASM14933v2	test (used)
Coccidioides posadasii	Coccidioides_posadasii	GCF_000151335.2_JCVI-cpa1-1.0	train_val
Colletotrichum aenigma	Colletotrichum_aenigma	GCF_013390185.1_ASM1339018v1	train_val
Colletotrichum fructicola	Colletotrichum_fructicola	GCF_009771025.1_ASM977102v1	train_val
Colletotrichum gloeosporioides	Colletotrichum_gloeosporioides	GCF_011800055.1_NFU_CgLc1_1.0	test
Colletotrichum graminicola	Colletotrichum_graminicola	GCF_000149035.1_C_graminicola_M1_001_V1	train_val
Colletotrichum higginsianum	Colletotrichum_higginsianum	GCF_001672515.1_ASM167251v1	train_val
Colletotrichum karsti	Colletotrichum_karsti	GCF_011947395.1_ASM1194739v2	train_val
Colletotrichum orchidophilum	Colletotrichum_orchidophilum	GCF_001831195.1_CORC01	test
Colletotrichum scovillei	Colletotrichum_scovillei	GCF_011075155.1_ASM1107515v1	test
Colletotrichum siamense	Colletotrichum_siamense	GCF_013390195.1_ASM1339019v1	train_val
Colletotrichum truncatum	Colletotrichum_truncatum	GCF_014235925.1_CTRU02	test
Coniophora puteana	Coniophora_puteana	GCF_000271625.1_Conpu1	train_val
Coniosporium apollinis	Coniosporium_apollinis	GCF_000281105.1_Coni_apol_CBS100218_V1	train_val
Coprinopsis cinerea	Coprinopsis_cinerea	GCF_000182895.1_CC3	train_val
Cordyceps fumosorosea	Cordyceps_fumosorosea	GCF_001636725.1_ISF_1.0	test
Cordyceps militaris	Cordyceps_militaris	GCF_000225605.1_CmilitarisCM01_v01	train_val
Cryphonectria parasitica	Cryphonectria_parasitica	GCF_011745365.1_Crypa2	test
Cryptococcus amylolentus	Cryptococcus_amylolentus	GCF_001720205.1_Cryp_amyl_CBS6039_V3	train_val
Cryptococcus gattii VGI	Cryptococcus_gattii_VGI	GCF_000185945.1_ASM18594v1	test
Cryptococcus neoformans	Cryptococcus_neoformans	GCF_000091045.1_ASM9104v1	train_val
Cryptococcus wingfieldii	Cryptococcus_wingfieldii	GCF_001720155.1_Tsuc_wing_CBS7118_V1	test
Cucurbitaria berberidis	Cucurbitaria_berberidis	GCF_010015615.1_Cucbe1	train_val
Cutaneotrichosporon oleaginosum	Cutaneotrichosporon_oleaginosum	GCF_001027345.1_Triol1	train_val
Cyberlindnera jadinii	Cyberlindnera_jadinii	GCF_001661405.1_Cybja1	train_val
Cyphellophora europaea	Cyphellophora_europaea	GCF_000365145.1_Phia_euro_CBS_101466_V1	train_val
Dacryopinax primogenitus	Dacryopinax_primogenitus	GCF_000292625.1_Dacryopinax_sp._DJM_731_SSP1_v1.0	train_val
Daldinia childiae	Daldinia_childiae	GCF_008694065.1_Dalch_JS-1345	test
Debaryomyces fabryi	Debaryomyces_fabryi	GCF_001447935.2_debFab1.1	train_val
Debaryomyces hansenii	Debaryomyces_hansenii	GCF_000006445.2_ASM644v2	test (used)
Diaporthe batatas	Diaporthe_batatas	GCF_019321695.1_ASM1932169v1	train_val
Diaporthe citri	Diaporthe_citri	GCF_014595645.1_ASM1459564v1	train_val
Dichomitus squalens	Dichomitus_squalens	GCF_000275845.1_Dichomitus_squalens_v1.0	train_val
Didymella exigua	Didymella_exigua	GCF_010094145.1_Didex1	test
Diplodia corticola	Diplodia_corticola	GCF_001883845.1_ASM188384v1	train_val
Dissoconium aciculare	Dissoconium_aciculare	GCF_010015565.1_Disac1	test
Diutina rugosa	Diutina_rugosa	GCF_008704595.1_ASM870459v1	train_val
Dothidotthia symphoricarpi	Dothidotthia_symphoricarpi	GCF_010015815.1_Dotsy1	train_val
Drechmeria coniospora	Drechmeria_coniospora	GCF_001625195.1_ASM162519v1	train_val
Drepanopeziza brunnea	Drepanopeziza_brunnea	GCF_000298775.1_ASM29877v1	test
Emericellopsis atlantica	Emericellopsis_atlantica	GCF_019669845.1_AcreTS7_1	train_val
Encephalitozoon cuniculi	Encephalitozoon_cuniculi	GCF_000091225.1_ASM9122v1	train_val
Encephalitozoon hellem	Encephalitozoon_hellem	GCF_000277815.2_ASM27781v3	train_val
Encephalitozoon intestinalis	Encephalitozoon_intestinalis	GCF_000146465.1_ASM14646v1	test

scientific	ID	version	split
Encephalitozoon romaleae	Encephalitozoon_romaleae	GCF_000280035.1_ASM28003v2	train_val
Endocarpon pusillum	Endocarpon_pusillum	GCF_000464535.1_EPUS	train_val
Eremomyces bilateralis	Eremomyces_bilateralis	GCF_010015585.1_Erebi1	train_val
Eremothecium cymbalariae	Eremothecium_cymbalariae	GCF_000235365.1_ASM23536v1	test
Eremothecium gossypii	Eremothecium_gossypii	GCF_000091025.4_ASM9102v4	train_val
Eremothecium sinecaudum	Eremothecium_sinecaudum	GCF_001548555.1_ASM154855v1	train_val
Exophiala aquamarina	Exophiala_aquamarina	GCF_000709125.1_Exop_aqua_CBS_119918_V1	train_val
Exophiala dermatitidis	Exophiala_dermatitidis	GCF_000230625.1_Exop_derm_V1	test
Exophiala mesophila	Exophiala_mesophila	GCF_000836275.1_Exop_meso_CBS40295_V1	test
Exophiala oligosperma	Exophiala_oligosperma	GCF_000835515.1_Exop_olig_CBS72588_V1	train_val
Exophiala spinifera	Exophiala_spinifera	GCF_000836115.1_Exop_spin_CBS89968_V1	train_val
Exophiala xenobiotica	Exophiala_xenobiotica	GCF_000835505.1_Exop_xeno_CBS118157_V1	train_val
Exserohilum turcicum	Exserohilum_turcicum	GCF_000359705.1_Setospaeria_trucica_Et28A_v1.0	train_val
Fibroporia radiculosa	Fibroporia_radiculosa	GCF_000313525.1_ASM31352v1	train_val
Filobasidium floriforme	Filobasidium_floriforme	GCF_021052385.1_Filflo1	train_val
Fomitiporia mediterranea	Fomitiporia_mediterranea	GCF_000271605.1_Fomme1	train_val
Fonsecaea erecta	Fonsecaea_erecta	GCF_001651985.1_ASM165198v1	train_val
Fonsecaea monophora	Fonsecaea_monophora	GCF_001642475.1_ASM164247v1	train_val
Fonsecaea multimorphosa	Fonsecaea_multimorphosa	GCF_000836435.1_Fons_mult_CBS_102226_V1	test
Fonsecaea nubica	Fonsecaea_nubica	GCF_001646965.1_ASM164696v1	train_val
Fonsecaea pedrosoi	Fonsecaea_pedrosoi	GCF_000835455.1_Fons_pedr_CBS_271_37_V1	train_val
Fusarium coffeatum	Fusarium_coffeatum	GCF_003316985.1_ASM331698v1	train_val
Fusarium flagelliforme	Fusarium_flagelliforme	GCF_020744385.1_Fuseq1	train_val
Fusarium fujikuroi	Fusarium_fujikuroi	GCF_900079805.1_Fusarium_fujikuroi_IMI58289_V2	train_val
Fusarium graminearum	Fusarium_graminearum	GCF_000240135.3_ASM24013v3	test (used)
Fusarium mangiferae	Fusarium_mangiferae	GCF_900044065.1_Genome_assembly_version_1	train_val
Fusarium musae	Fusarium_musae	GCF_019915245.1_ASM1991524v1	test
Fusarium odoratissimum	Fusarium_odoratissimum	GCF_000260195.1_FO_II5_V1	train_val
Fusarium oxysporum	Fusarium_oxysporum	GCF_000271745.1_FO_FOSC_3_a_V1	train_val
Fusarium poae	Fusarium_poae	GCF_019609905.1_ASM1960990v1	test
Fusarium proliferatum	Fusarium_proliferatum	GCF_900067095.1_F._proliferatum_ET1_version_1	train_val
Fusarium pseudograminearum	Fusarium_pseudograminearum	GCF_000303195.2_FP7	train_val
Fusarium redolens	Fusarium_redolens	GCF_020744475.1_Fusre1	train_val
Fusarium solani	Fusarium_solani	GCF_020744495.1_Fusso1	test
Fusarium subglutinans	Fusarium_subglutinans	GCF_013396075.1_ASM1339607v1	test
Fusarium tjaetaba	Fusarium_tjaetaba	GCF_013396195.1_ASM1339619v1	test
Fusarium vanettenii	Fusarium_vanettenii	GCF_000151355.1_v2.0	test
Fusarium venenatum	Fusarium_venenatum	GCF_900007375.1_ASM90000737v1	train_val
Fusarium verticillioides	Fusarium_verticillioides	GCF_000149555.1_ASM14955v1	test
Gaeumannomyces tritici	Gaeumannomyces_tritici	GCF_000145635.1_Gae_graminis_V2	test
Geosmithia morbida	Geosmithia_morbida	GCF_012550715.1_ASM1255071v1	train_val
Glarea lozoyensis	Glarea_lozoyensis	GCF_000409485.1_GLAREA	test
Gloeophyllum trabeum	Gloeophyllum_trabeum	GCF_000344685.1_Glotr1_1	test
Grosmannia clavigera	Grosmannia_clavigera	GCF_000143105.1_Sanger-454-IlluminaPA_2.0	train_val
Guyanagaster necrorhizus	Guyanagaster_necrorhizus	GCF_019112545.1_Guyne1	train_val
Heterobasidion irregulare	Heterobasidion_irregulare	GCF_000320585.1_Heterobasidion_irregulare_v2.0	train_val
Hirsutella rhossiliensis	Hirsutella_rhossiliensis	GCF_020360975.1_ASM2036097v1	train_val
Histoplasma capsulatum	Histoplasma_capsulatum	GCF_000150115.1_ASM15011v1	train_val
Histoplasma mississippiense nom inval	Histoplasma_mississippiense_nom._inval._	GCF_000149585.1_ASM14958v1	train_val
Hyaloscypha bicolor	Hyaloscypha_bicolor	GCF_002865645.1_Melbi2	train_val
Hyphopichia burtonii	Hyphopichia_burtonii	GCF_001661395.1_Hypbu1	train_val
Ilyonectria robusta	Ilyonectria_robusta	GCF_021365365.1_Ilyrob1	test
Jaminaea rosea	Jaminaea_rosea	GCF_003144245.1_Jamsp1	train_val
Kalmanozyma brasiliensis	Kalmanozyma_brasiliensis	GCF_000497045.1_PSEUBRA1	test
Kazachstania africana	Kazachstania_africana	GCF_000304475.1_Ka_CBS2517	test
Kazachstania barnettii	Kazachstania_barnettii	GCF_903064755.1_KABA2	test
Kazachstania naganishii	Kazachstania_naganishii	GCF_000348985.1_ASM34898v1	test
Kluyveromyces lactis	Kluyveromyces_lactis	GCF_000002515.2_ASM251v1	train_val
Kluyveromyces marxianus	Kluyveromyces_marxianus	GCF_001417885.1_Kmar_1.0	test
Kockovaella imperatae	Kockovaella_imperatae	GCF_002102565.1_Kocim1	train_val
Komagataella phaffii	Komagataella_phaffii	GCF_000027005.1_ASM2700v1	train_val
Kuraishia capsulata	Kuraishia_capsulata	GCF_000576695.1_AUH_PRJEB4427_v1	train_val
Kwoniella bestiolae	Kwoniella_bestiolae	GCF_000512585.1_Cryp_best_CBS10118_V1	train_val
Kwoniella dejecticola	Kwoniella_dejecticola	GCF_000512565.1_Cryp_deje_CBS10117_V1	train_val
Kwoniella mangrovensis	Kwoniella_mangrovensis	GCF_000507465.1_Kwon_mang_CBS8507_V2	train_val
Kwoniella pini	Kwoniella_pini	GCF_000512605.1_Cryp_pinu_CBS10737_V1	train_val
Kwoniella shandongensis	Kwoniella_shandongensis	GCF_008629635.1_Kwon_shan_CBS_12478_V1	train_val
Laccaria bicolor	Laccaria_bicolor	GCF_000143565.1_V1.0	test (used)
Lachancea lanzarotensis	Lachancea_lanzarotensis	GCF_000938715.1_LALA0	test
Lachancea thermotolerans	Lachancea_thermotolerans	GCF_000142805.1_ASM14280v1	train_val
Lachnellula hyalina	Lachnellula_hyalina	GCF_007821495.1_CFIA_Lhya_EG2017	test
Laetiporus sulphureus	Laetiporus_sulphureus	GCF_001632365.1_Laesu1	train_val
Lasiodiplodia theobromae	Lasiodiplodia_theobromae	GCF_012971845.1_ASM1297184v1	train_val
Lentinula edodes	Lentinula_edodes	GCF_021015755.1_Lenedo1	train_val
Leptosphaeria maculans	Leptosphaeria_maculans	GCF_000230375.1_ASM23037v1	train_val
Letharia columbiana	Letharia_columbiana	GCF_014066305.1_Lecol_v1.0	train_val
Letharia lupina	Letharia_lupina	GCF_014066315.1_Lelup_v1.1	test
Linderina pennispora	Linderina_pennispora	GCF_002104995.1_Linpe1	test
Lindgomyces ingoldianus	Lindgomyces_ingoldianus	GCF_010093535.1_Linin1	train_val
Lobosporangium transversale	Lobosporangium_transversale	GCF_002105155.1_Lobtra1	test

scientific	ID	version	split
Lodderomyces elongisporus	Lodderomyces_elongisporus	GCF_000149685.1_ASM14968v1	train_val
Macroventuria anomochaeta	Macroventuria_anomochaeta	GCF_010093625.1_Macan1	train_val
Malassezia globosa	Malassezia_globosa	GCF_000181695.1_ASM18169v1	train_val
Malassezia pachydermatis	Malassezia_pachydermatis	GCF_001278385.1_MalaPachy	train_val
Malassezia restricta	Malassezia_restricta	GCF_003290485.1_ASM329048v1	test
Malassezia sympodialis	Malassezia_sympodialis	GCF_000349305.1_ASM34930v2	train_val
Marasmius oreades	Marasmius_oreades	GCF_018924745.1_UU_Maror_2	test
Meira miltonrushii	Meira_miltonrushii	GCF_003144205.1_Meimi1	train_val
Melampsora larici-populina	Melampsora_larici-populina	GCF_000204055.1_v1.0	train_val
Metarhizium acridum	Metarhizium_acridum	GCF_000187405.1_MetAcr_May2010	test
Metarhizium album	Metarhizium_album	GCF_000804445.1_MAM_1.0_for_version_1_of_the_Me...	train_val
Metarhizium brunneum	Metarhizium_brunneum	GCF_000814965.1_MBR_1.0	train_val
Metarhizium robertsii	Metarhizium_robertsii	GCF_000187425.2_MAA_2.0	test
Metschnikowia bicuspidata	Metschnikowia_bicuspidata	GCF_001664035.1_Metbi1	train_val
Meyerozyma guilliermondii	Meyerozyma_guilliermondii	GCF_000149425.1_ASM14942v1	train_val
Microdochium trichocladiopsis	Microdochium_trichocladiopsis	GCF_020744255.1_Mictri1	test
Microsporum canis	Microsporum_canis	GCF_000151145.1_ASM15114v1	train_val
Mitosporidium daphniae	Mitosporidium_daphniae	GCF_000760515.2_UGP1.1	test
Mixia osmundae	Mixia_osmundae	GCF_000708205.1_Mixia_osmundae_v1.0	train_val
Moesziomyces antarcticus	Moesziomyces_antarcticus	GCF_000747765.1_ASM74776v1	train_val
Mollisia scopiformis	Mollisia_scopiformis	GCF_001500285.1_Phisc1	train_val
Morchella importuna	Morchella_importuna	GCF_003444635.1_ASM344463v2	train_val
Morchella sextelata	Morchella_sextelata	GCF_020137385.1_ASM2013738v1	train_val
Mycena indigotica	Mycena_indigotica	GCF_014461135.1_ASM1446113v1	train_val
Mytilinidion resinicola	Mytilinidion_resinicola	GCF_010093595.1_Mytre1	train_val
Nannizzia gypsea	Nannizzia_gypsea	GCF_000150975.2_MS_CBS118893	test
Naumovozyma castellii	Naumovozyma_castellii	GCF_000237345.1_ASM23734v1	train_val
Naumovozyma dairenensis	Naumovozyma_dairenensis	GCF_000227115.2_ASM22711v2	train_val
Nematocida parisii	Nematocida_parisii	GCF_000250985.1_Nema_parisii_ERTm1_V3	train_val
Neohortaea acidophila	Neohortaea_acidophila	GCF_010093505.1_Horac1	train_val
Neurospora crassa	Neurospora_crassa	GCF_000182925.2_NC12	test (used)
Neurospora tetrasperma	Neurospora_tetrasperma	GCF_000213175.1_v2.0	train_val
Nosema ceranae	Nosema_ceranae	GCF_000988165.1_ASM98816v1	train_val
Ogataea angusta	Ogataea_angusta	GCF_019207475.1_ASM1920747v1	test
Ogataea haglerorum	Ogataea_haglerorum	GCF_019207285.1_ASM1920728v1	train_val
Ogataea parapolymorpha	Ogataea_parapolymorpha	GCF_000187245.1_Hansenula_2	train_val
Ogataea philodendri	Ogataea_philodendri	GCF_020536065.1_ASM2053606v1	test
Ogataea polymorpha	Ogataea_polymorpha	GCF_001664045.1_Hanpo2	train_val
Orbilia oligospora	Orbilia_oligospora	GCF_000225545.1_AOL24927_1.0	test
Ordospora colligata	Ordospora_colligata	GCF_000803265.1_ASM80326v1	test
Paecilomyces variotii	Paecilomyces_variotii	GCF_004022145.1_Paevar1	test
Paracoccidioides brasiliensis	Paracoccidioides_brasiliensis	GCF_000150735.1_Paracocci_br_Pb18_V2	test
Paracoccidioides lutzii	Paracoccidioides_lutzii	GCF_000150705.2_Paracocci_br_Pb01_V2	test
Paraphaeosphaeria sporulosa	Paraphaeosphaeria_sporulosa	GCF_001642045.1_Parsp1	train_val
Parastagonospora nodorum	Parastagonospora_nodorum	GCF_000146915.1_ASM14691v2	train_val
Penicilliopsis zonata	Penicilliopsis_zonata	GCF_001890105.1_Aspzo1	train_val
Penicillium arizonense	Penicillium_arizonense	GCF_001773325.1_ASM177332v1	test
Penicillium digitatum	Penicillium_digitatum	GCF_000315645.1_PdigPd1_v1	train_val
Penicillium expansum	Penicillium_expansum	GCF_000769745.1_ASM76974v1	test
Penicillium griseofulvum	Penicillium_griseofulvum	GCF_001561935.1_ASM156193v1	train_val
Penicillium roqueforti	Penicillium_roqueforti	GCF_015533775.1_ASM1553377v1	train_val
Penicillium rubens	Penicillium_rubens	GCF_000226395.1_PenChr_Nov2007	train_val
Penicillium solitum	Penicillium_solitum	GCF_002072235.1_ASM207223v1	train_val
Pestalotiopsis fici	Pestalotiopsis_fici	GCF_000516985.1_PFICI	train_val
Phaeoacremonium minimum	Phaeoacremonium_minimum	GCF_000392275.1_UCRPA7V03	train_val
Phanerochaete carnosa	Phanerochaete_carnosa	GCF_000300595.1_Phanerochaete_carnosa_HHB-10118...	test
Phialophora attinorum	Phialophora_attinorum	GCF_001299255.1_ASM129925v1	test
Phycomyces blakesleeanus	Phycomyces_blakesleeanus	GCF_001638985.1_Phybl2	train_val
Pichia kudriavzevii	Pichia_kudriavzevii	GCF_003054445.1_ASM305444v1	train_val
Pichia membranifaciens	Pichia_membranifaciens	GCF_001661235.1_Picme2	train_val
Pleurotus ostreatus	Pleurotus_ostreatus	GCF_014466165.1_ASM1446616v1	train_val
Pneumocystis carinii	Pneumocystis_carinii	GCF_001477545.1_Pneu_cari_B80_V3	test
Pneumocystis jirovecii	Pneumocystis_jirovecii	GCF_001477535.1_Pneu_jiro_RU7_V2	train_val
Pneumocystis murina	Pneumocystis_murina	GCF_000349005.2_Pneumo_murina_B123_V4	train_val
Pochonia chlamydosporia	Pochonia_chlamydosporia	GCF_001653235.2_ASM165323v2	train_val
Podospora anserina	Podospora_anserina	GCF_000226545.1_ASM22654v1	train_val
Postia placenta	Postia_placenta	GCF_002117355.1_PosplRSB12_1	train_val
Protomyces lactucae-debilis	Protomyces_lactucae-debilis	GCF_002105105.1_Prola1	train_val
Pseudocercospora fijiensis	Pseudocercospora_fijiensis	GCF_000340215.1_Mycfi2	test
Pseudogymnoascus destructans	Pseudogymnoascus_destructans	GCF_001641265.1_ASM164126v1	train_val
Pseudogymnoascus verrucosus	Pseudogymnoascus_verrucosus	GCF_001662655.1_ASM166265v1	train_val
Pseudomassariella vexata	Pseudomassariella_vexata	GCF_002105095.1_Pseve2	train_val
Pseudomicrostroma glucosiphilum	Pseudomicrostroma_glucosiphilum	GCF_003144135.1_Rhodsp1	train_val
Pseudovirgaria hyperparasitica	Pseudovirgaria_hyperparasitica	GCF_010093815.1_Psehy1	train_val
Pseudozyma flocculosa	Pseudozyma_flocculosa	GCF_000417875.1_Pflocc_1.0	train_val
Pseudozyma hubeiensis	Pseudozyma_hubeiensis	GCF_000403515.1_ASM40351v1	train_val
Puccinia graminis	Puccinia_graminis	GCF_000149925.1_ASM14992v1	test
Punctularia strigosozonata	Punctularia_strigosozonata	GCF_000264995.1_Punctularia_strigosozonata_v1.0	train_val
Purpureocillium lilacinum	Purpureocillium_lilacinum	GCF_001653265.1_ASM165326v1	test
Pyrenophora tritici-repentis	Pyrenophora_tritici-repentis	GCF_000149985.1_ASM14998v1	train_val

scientific	ID	version	split
Pyricularia grisea	Pyricularia_grisea	GCF_004355905.1_ASM435590v1	train_val
Pyricularia oryzae	Pyricularia_oryzae	GCF_000002495.2_MG8	train_val
Pyricularia pennisetigena	Pyricularia_pennisetigena	GCF_004337985.1_ASM433798v1	train_val
Ramularia collo-cygni	Ramularia_collo-cygni	GCF_900074925.1_version_1	train_val
Rasamsonia emersonii	Rasamsonia_emersonii	GCF_000968595.1_ASM96859v1	test
Rhinocladiella mackenziei	Rhinocladiella_mackenziei	GCF_000835555.1_Rhin_mack_CBS_650_93_V1	train_val
Rhizoctonia solani	Rhizoctonia_solani	GCF_016906535.1_ASM1690653v1	test
Rhizophagus irregularis	Rhizophagus_irregularis	GCF_000439145.1_ASM43914v3	train_val
Rhizopus microsporus	Rhizopus_microsporus	GCF_002708625.1_Rhimi1_1	test
Rhodotorula graminis	Rhodotorula_graminis	GCF_001329695.1_Rhoba1_1	test
Rhodotorula toruloides	Rhodotorula_toruloides	GCF_000320785.1_RHOziaDV1.0	train_val
Saccharomyces cerevisiae	Saccharomyces_cerevisiae	GCF_000146045.2_R64	train_val
Saccharomyces eubayanus	Saccharomyces_eubayanus	GCF_001298625.1_SEUB3.0	test
Saccharomyces paradoxus	Saccharomyces_paradoxus	GCF_002079055.1_ASM207905v1	train_val
Saccharomycodes ludwigii	Saccharomycodes_ludwigii	GCF_020623625.1_UHD_SCDLUD_16	train_val
Saitoella complicata	Saitoella_complicata	GCF_001661265.1_Saico1	train_val
Saprochaete ingens	Saprochaete_ingens	GCF_902498895.1_sapIngB	train_val
Scedosporium apiospermum	Scedosporium_apiospermum	GCF_000732125.1_ScApio1.0	train_val
Scheffersomyces spartinae	Scheffersomyces_spartinae	GCF_019049425.1_ASM1904942v1	train_val
Scheffersomyces stipitis	Scheffersomyces_stipitis	GCF_000209165.1_ASM20916v1	train_val
Schizophyllum commune	Schizophyllum_commune	GCF_000143185.1_v1.0	train_val
Schizosaccharomyces cryophilus	Schizosaccharomyces_cryophilus	GCF_000004155.1_SCY4	train_val
Schizosaccharomyces japonicus	Schizosaccharomyces_japonicus	GCF_000149845.2_SJ5	train_val
Schizosaccharomyces octosporus	Schizosaccharomyces_octosporus	GCF_000150505.1_SO6	test
Schizosaccharomyces pombe	Schizosaccharomyces_pombe	GCF_000002945.1_ASM294v2	train_val
Sclerotinia sclerotiorum	Sclerotinia_sclerotiorum	GCF_000146945.2_ASM14694v2	train_val
Serpula lacrymans	Serpula_lacrymans	GCF_000218685.1_v1.0	test
Sodiomyces alkalinus	Sodiomyces_alkalinus	GCF_003711515.1_Sodal1	test
Sordaria macrospora	Sordaria_macrospora	GCF_000182805.2_ASM18280v2	train_val
Sparassis crispa	Sparassis_crispa	GCF_003851025.1_SCP_1.1	train_val
Spathaspora passalidarum	Spathaspora_passalidarum	GCF_000223485.1_Spathaspora_passalidarum_v2.0	train_val
Sphaerulina musiva	Sphaerulina_musiva	GCF_000320565.1_Septoria_musiva_SO2202_v1.0	train_val
Spizellomyces punctatus	Spizellomyces_punctatus	GCF_000182565.1_S_punctatus_V1	train_val
Sporisorium graminicola	Sporisorium_graminicola	GCF_005498985.1_PGRAM_IIB_1.0	train_val
Sporothrix brasiliensis	Sporothrix_brasiliensis	GCF_000820605.1_S_brasiliensis_5110_v1	train_val
Sporothrix schenckii	Sporothrix_schenckii	GCF_000961545.1_S_schenckii_v1	train_val
Stereum hirsutum	Stereum_hirsutum	GCF_000264905.1_Stehi1	train_val
Sugiyamaella lignohabitans	Sugiyamaella_lignohabitans	GCF_001640025.1_ASM164002v2	train_val
Suhomyces tanzawaensis	Suhomyces_tanzawaensis	GCF_001661415.1_Canta1	test
Suillus bovinus	Suillus_bovinus	GCF_016758785.1_Suibov1	train_val
Suillus clintonianus	Suillus_clintonianus	GCF_016758775.1_Suicli1	train_val
Suillus discolor	Suillus_discolor	GCF_016758755.1_Suidis1	train_val
Suillus fuscotomentosus	Suillus_fuscotomentosus	GCF_016647785.1_Suifus1	test
Suillus paluster	Suillus_paluster	GCF_016628075.1_Suipal1	train_val
Suillus plorans	Suillus_plorans	GCF_016647745.1_Suiplo1	train_val
Suillus subalutaceus	Suillus_subalutaceus	GCF_016647625.1_Suisu1	test
Suillus subaureus	Suillus_subaureus	GCF_016647635.1_Suisub1	train_val
Synchytrium microbalum	Synchytrium_microbalum	GCF_006535985.1_ASM653598v1	train_val
Talaromyces amestolkiae	Talaromyces_amestolkiae	GCF_001896365.1_ASM189636v1	train_val
Talaromyces atroroseus	Talaromyces_atroroseus	GCF_001907595.1_ASM190759v1	train_val
Talaromyces marneffei	Talaromyces_marneffei	GCF_000001985.1_JCVI-PMFA1-2.0	train_val
Talaromyces proteolyticus	Talaromyces_proteolyticus	GCF_021365285.1_Talpro1	test
Talaromyces rugulosus	Talaromyces_rugulosus	GCF_013368755.1_ASM1336875v1	train_val
Talaromyces stipitatus	Talaromyces_stipitatus	GCF_000003125.1_JCVI-TSTA1-3.0	train_val
Tetrapisispora blattae	Tetrapisispora_blattae	GCF_000315915.1_ASM31591v1	train_val
Tetrapisispora phaffii	Tetrapisispora_phaffii	GCF_000236905.1_ASM23690v1	test
Thermothelomyces thermophilus	Thermothelomyces_thermophilus	GCF_000226095.1_ASM22609v1	train_val
Thermothielavioides terrestris	Thermothielavioides_terrestris	GCF_000226115.1_ASM22611v1	train_val
Thyridium curvatum	Thyridium_curvatum	GCF_004353045.1_ASM435304v1	train_val
Tilletiaria anomala	Tilletiaria_anomala	GCF_000711695.1_Tilletiaria_anomala_UBC_951_v1.0	train_val
Tilletiopsis washingtonensis	Tilletiopsis_washingtonensis	GCF_003144115.1_Tilwa1	train_val
Torulaspora delbrueckii	Torulaspora_delbrueckii	GCF_000243375.1_ASM24337v1	test
Torulaspora globosa	Torulaspora_globosa	GCF_014133895.1_ASM1413389v1	test
Trametes versicolor	Trametes_versicolor	GCF_000271585.1_Trametes_versicolor_v1.0	train_val
Trematosphaeria pertusa	Trematosphaeria_pertusa	GCF_010094035.1_Trepe1	test
Tremella mesenterica	Tremella_mesenterica	GCF_000271645.1_Treme1	train_val
Trichoderma asperellum	Trichoderma_asperellum	GCF_003025105.1_Trias_v._1.0	test
Trichoderma atroviride	Trichoderma_atroviride	GCF_000171015.1_TRIAT_v2.0	train_val
Trichoderma citrinoviride	Trichoderma_citrinoviride	GCF_003025115.1_Trici_v4.0	train_val
Trichoderma gamsii	Trichoderma_gamsii	GCF_001481775.2_TGAM01v2	train_val
Trichoderma harzianum	Trichoderma_harzianum	GCF_003025095.1_Triha_v1.0	train_val
Trichoderma reesei	Trichoderma_reesei	GCF_000167675.1_v2.0	train_val
Trichoderma virens	Trichoderma_virens	GCF_000170995.1_TRIVI_v2.0	train_val
Trichophyton benhamiae	Trichophyton_benhamiae	GCF_000151125.1_ASM15112v2	train_val
Trichophyton rubrum	Trichophyton_rubrum	GCF_000151425.1_ASM15142v1	test
Trichophyton verrucosum	Trichophyton_verrucosum	GCF_000151505.1_ASM15150v1	test
Trichosporon asahii	Trichosporon_asahii	GCF_000293215.1_Trichosporon_asahii_1	test
Truncatella angustata	Truncatella_angustata	GCF_020726525.1_Truan1	test
Tuber melanosporum	Tuber_melanosporum	GCF_000151645.1_ASM15164v1	train_val
Uncinocarpus reesii	Uncinocarpus_reesii	GCF_000003515.1_ASM351v2	train_val

scientific	ID	version	split
Ustilaginoidea virens	Ustilaginoidea_virens	GCF_000687475.1_ASM68747v2	test
Ustilago hordei	Ustilago_hordei	GCF_900519145.1_Uho2_v1	train_val
Ustilago maydis	Ustilago_maydis	GCF_000328475.2_Umaydis521_2.0	train_val
Vanderwaltozyma polyspora	Vanderwaltozyma_polyspora	GCF_000150035.1_ASM15003v1	train_val
Vavraia culicis	Vavraia_culicis	GCF_000192795.1_Vavr_culi_floridensis_V1	train_val
Venustampulla echinocandica	Venustampulla_echinocandica	GCF_003357145.1_ASM335714v1	train_val
Verruconis gallopava	Verruconis_gallopava	GCF_000836295.1_O_gall_CBS43764	test
Verticillium alfalfae	Verticillium_alfalfae	GCF_000150825.1_ASM15082v1	train_val
Verticillium dahliae	Verticillium_dahliae	GCF_000150675.1_ASM15067v2	train_val
Verticillium nonalfalfae	Verticillium_nonalfalfae	GCF_003724135.2_ASM372413v2	test
Vittaforma corneae	Vittaforma_corneae	GCF_000231115.1_Vitt_corn_V1	train_val
Wallemia ichthyophaga	Wallemia_ichthyophaga	GCF_000400465.1_Wallemia_ichthyophaga_version_1.0	train_val
Wallemia mellicola	Wallemia_mellicola	GCF_000263375.1_Wallemia_sebi_v1.0	train_val
Westerdykella ornata	Westerdykella_ornata	GCF_010094085.1_Wesor1	test
Wickerhamiella sorbophila	Wickerhamiella_sorbophila	GCF_002251995.1_ASM225199v2	train_val
Wickerhamomyces anomalus	Wickerhamomyces_anomalus	GCF_001661255.1_Wican1	train_val
Wickerhamomyces ciferrii	Wickerhamomyces_ciferrii	GCF_000313485.1_ASM31348v1	train_val
Xylona heveae	Xylona_heveae	GCF_001619985.1_Xylona_heveae_TC161_v1.0	train_val
Yamadazyma tenuis	Yamadazyma_tenuis	GCF_000223465.1_Candida_tenuis_v1.0	train_val
Yarrowia lipolytica	Yarrowia_lipolytica	GCF_000002525.2_ASM252v1	train_val
Zasmidium cellare	Zasmidium_cellare	GCF_010093935.1_Zasce1	train_val
Zygosaccharomyces rouxii	Zygosaccharomyces_rouxii	GCF_000026365.1_ASM2636v1	train_val
Zygotorulaspora mrakii	Zygotorulaspora_mrakii	GCF_013402915.1_ASM1340291v1	test
Zymoseptoria tritici	Zymoseptoria_tritici	GCF_000219625.1_MYCGR_v2.0	train_val

Table S18: Overview of assessed plant species, including their scientific names, identification, and version. The "Split" column indicates whether each species was designated for model training/validation or model evaluation purposes.

scientific	ID	version	split
Ananas comosus	Acomosus	v3	train_val
Amaranthus hypochondriacus	Ahypochondriacus	v2.1	train_val
Arabidopsis lyrata	Alyrata	v2.1	train_val
Asparagus officinalis	Aofficinalis	V1.1	train_val
Arabidopsis thaliana	Athaliana	TAIR10	train_val
Amborella trichopoda	Atrichopoda	v1.0	train_val
Brachypodium distachyon	Bdistachyon	v3.1	train_val
Brachypodium hybridum	Bhybridum	v1.1	train_val
Brassica oleracea	Boleraceacapitata	v1.0	train_val
Brassica rapa	BrapaFPsc	v1.3	train_val
Beta vulgaris	Bvulgaris	EL10_1.0	train_val
Cicer arietinum	Carietinum	v1.0	train_val
Citrus clementina	Cclementina	v1.0	train_val
Capsella grandiflora	Cgrandiflora	v1.1	train_val
Cinnamomum kanehirae	Ckanehirae	v3	train_val
Carica papaya	Cpapaya	ASGPBv0.4	train_val
Chenopodium quinoa	Cquinoa	v1.0	train_val
Chlamydomonas reinhardtii	Creinhardtii	v5.6	train_val
Capsella rubella	Crubella	v1.1	train_val
Cucumis sativus	Csativus	v1.0	train_val
Citrus sinensis	Csinensis	v1.1	train_val
Coccomyxa subellipsoidea C-169	CsubellipsoideaC169	v2.0	train_val
Chromochloris zofingiensis	Czofingiensis	v5.2.3.2	train_val
Dioscorea alata	Dalata	v2.1	train_val
Daucus carota	Dcarota	v2.0	train_val
Dunaliella salina	Dsalina	v1.0	train_val
Eucalyptus grandis	Egrandis	v2.0	train_val
Eutrema salsugineum	Esalsugineum	v1.0	train_val
Fragaria vesca	Fvesca	v4.0.a2	train_val
Glycine max	Gmax	Wm82.a4.v1	train_val
Gossypium raimondii	Graimondii	v2.1	train_val
Glycine soja	Gsoja	v1.1	train_val
Helianthus annuus	Hannuus	r1.2	train_val
Hordeum vulgare	Hvulgare	r1	train_val
Kalanchoe fedtschenkoi	Kfedtschenkoi	v1.1	train_val
Lupinus albus	Lalbus	v1	train_val
Lotus japonicus	Ljaponicus	Lj1.0v1	train_val
Lactuca sativa	Lsativa	v5	train_val
Linum usitatissimum	Lusitatissimum	v1.0	train_val
Musa acuminata	Macuminata	v1	train_val
Malus domestica	Mdomestica	v1.1	train_val
Manihot esculenta	Mesculenta	v8.1	train_val
Mimulus guttatus	Mguttatus	v2.0	train_val
Marchantia polymorpha	Mpolymorpha	v3.1	train_val
Micromonas pusilla	MpusillaCCMP1545	v3.0	train_val
Micromonas sp. RCC299	MspRCC299	v3.0	train_val
Medicago truncatula	Mtruncatula	Mt4.0v1	train_val
Nymphaea colorata	Ncolorata	v1.2	train_val
Olea europaea	Oeuropaea	v1.0	train_val
Ostreococcus lucimarinus	Olucimarinus	v2.0	train_val
Oryza sativa	Osativa	v7.0	train_val
Oropetium thomaeum	Othomaeum	v1.0	train_val
Phaseolus acutifolius	Pacutifolius	v1.0	train_val
Panicum hallii	Phallii	v3.2	train_val
Physcomitrella patens	Ppatens	v3.3	train_val
Prunus persica	Ppersica	v2.1	train_val
Populus trichocarpa	Ptrichocarpa	v4.1	train_val
Poncirus trifoliata	Ptrifoliata	v1.3.1	train_val
Porphyra umbilicalis	Pumbilicalis	v1.5	train_val
Panicum virgatum	Pvirgatum	v5.1	train_val
Ricinus communis	Rcommunis	v0.1	train_val
Sorghum bicolor	Sbicolor	v3.1.1	train_val
Setaria italica	Sitalica	v2.2	train_val
Solanum lycopersicum	Slycopersicum	ITAG3.2	train_val
Selaginella moellendorffii	Smoellendorffii	v1.0	train_val
Schrenkiella parvula	Sparvula	v2.2	train_val
Spirodela polyrhiza	Spolyrhiza	v2	train_val
Salix purpurea	Spurpurea	v1.0	train_val
Solanum tuberosum	Stuberosum	v4.03	train_val
Triticum aestivum	Taestivum	v2.2	train_val
Theobroma cacao	Tcacao	v2.1	train_val
Trifolium pratense	Tpratense	v2	train_val
Volvox carteri	Vcarteri	v2.1	train_val
Vigna unguiculata	Vunguiculata	v1.2	train_val
Vitis vinifera	Vvinifera	v2.1	train_val
Zostera marina	Zmarina	v3.1	train_val
Zea mays	Zmays	RefGen_V4	train_val
Arachis hypogaea	Arachis_hypogaea	GCF_003086295.2_arahy.Tifrunner.gnm1.KYV3	test (used)
Brassica napus	Brassica_napus	GCF_020379485.1_Da-Ae	test (used)
Cannabis sativa	Cannabis_sativa	GCF_900626175.2_cs10	test (used)

scientific	ID	version	split
Coffea arabica	Coffea_arabica	GCF_003713225.1_Cara_1.0	test (used)
Hibiscus syriacus	Hibiscus_syriacus	GCF_006381635.1_ASM638163v2	test (used)
Nicotiana attenuata	Nicotiana_attenuata	GCF_001879085.1_NIATTr2	test (used)
Oryza brachyantha	Oryza_brachyantha	GCF_000231095.2_ObraRS2	test (used)
Papaver somniferum	Papaver_somniferum	GCF_003573695.1_ASM357369v1	test (used)
Phoenix dactylifera	Phoenix_dactylifera	GCF_009389715.1_palm_55x_up_171113_PBpolish2nd_...	test (used)
Setaria viridis	Setaria_viridis	GCF_005286985.1_Setaria_viridis_v2.0	test (used)
Solanum pennellii	Solanum_pennellii	GCF_001406875.1_SPENNV200	test (used)
Triticum dicoccoides	Triticum_dicoccoides	GCF_002162155.2_WEW_v2.1	test (used)
Vitis riparia	Vitis_riparia	GCF_004353265.1_EGFV_Vit.rip_1.0	test (used)

Table S19: Overview of assessed vertebrate genomes, including their scientific names, identification, and version. The "Split" column indicates whether each species was designated for model training/validation or model evaluation purposes.

scientific	ID	version	split
Acinonyx jubatus	Acinonyx_jubatus	GCF_003709585.1_Aci_jub_2	train_val
Ailuropoda melanoleuca	Ailuropoda_melanoleuca	GCF_002007445.1_ASM200744v2	train_val
Aotus nancymaae	Aotus_nancymaae	GCF_000952055.2_Anan_2.0	train_val
Artibeus jamaicensis	Artibeus_jamaicensis	GCF_014825515.1_WHU_Ajam_v2	test
Arvicanthis niloticus	Arvicanthis_niloticus	GCF_011762505.1_mArvNil1.pat.X	test
Arvicola amphibius	Arvicola_amphibius	GCF_903992535.2_mArvAmp1.2	train_val
Balaenoptera acutorostrata	Balaenoptera_acutorostrata	GCF_000493695.1_BalAcu1.0	test
Balaenoptera musculus	Balaenoptera_musculus	GCF_009873245.2_mBalMus1.pri.v3	test (used)
Bison bison	Bison_bison	GCF_000754665.1_Bison_UMD1.0	train_val
Bos indicus	Bos_indicus	GCF_000247795.1_Bos_indicus_1.0	train_val
Bos indicus x Bos taurus	Bos_indicus_x_Bos_taurus	GCF_003369695.1_UOA_Brahman_1	test
Bos mutus	Bos_mutus	GCF_000298355.1_BosGru_v2.0	train_val
Bos taurus	Bos_taurus	GCF_002263795.1_ARS-UCD1.2	train_val
Bubalus bubalis	Bubalus_bubalis	GCF_019923935.1_NDDB_SH_1	train_val
Callithrix jacchus	Callithrix_jacchus	GCF_009663435.1_Callithrix_jacchus_cj1700_1.1	train_val
Callorhinus ursinus	Callorhinus_ursinus	GCF_003265705.1_ASM326570v1	train_val
Camelus bactrianus	Camelus_bactrianus	GCF_000767855.1_Ca_bactrianus_MBC_1.0	test
Camelus dromedarius	Camelus_dromedarius	GCF_000803125.2_CamDro3	train_val
Camelus ferus	Camelus_ferus	GCF_009834535.1_BCGSAC_Cfer_1.0	test
Canis lupus dingo	Canis_lupus_dingo	GCF_012295265.1_UNSW_AlpineDingo_1.0	train_val
Canis lupus familiaris	Canis_lupus_familiaris	GCF_014441545.1_ROS_Cfam_1.0	train_val
Capra hircus	Capra_hircus	GCF_001704415.1_ARS1	train_val
Carlito syrichta	Carlito_syrichta	GCF_000164805.1_Tarsius_syrichta-2.0.1	train_val
Castor canadensis	Castor_canadensis	GCF_001984765.1_C.can_genome_v1.0	train_val
Cavia porcellus	Cavia_porcellus	GCF_000151735.1_Cavpor3.0	train_val
Cebus imitator	Cebus_imitator	GCF_001604975.1_Cebus_imitator-1.0	train_val
Ceratotherium simum	Ceratotherium_simum	GCF_000283155.1_CerSimSim1.0	train_val
Cercocebus atys	Cercocebus_atys	GCF_000955945.1_Caty_1.0	train_val
Cervus canadensis	Cervus_canadensis	GCF_019320065.1_ASM1932006v1	test
Cervus elaphus	Cervus_elaphus	GCF_910594005.1_mCerEla1.1	train_val
Chinchilla lanigera	Chinchilla_lanigera	GCF_000276665.1_ChiLan1.0	train_val
Chlorocebus sabaeus	Chlorocebus_sabaeus	GCF_015252025.1_Vero_WHO_p1.0	train_val
Choloepus didactylus	Choloepus_didactylus	GCF_015220235.1_mChoDid1.pri	test
Chrysochloris asiatica	Chrysochloris_asiatica	GCF_000296735.1_ChrAsi1.0	train_val
Colobus angolensis	Colobus_angolensis	GCF_000951035.1_Cang.pa_1.0	train_val
Condylura cristata	Condylura_cristata	GCF_000260355.1_ConCri1.0	test
Cricetulus griseus	Cricetulus_griseus	GCF_000223135.1_CriGri_1.0	train_val
Dasypus novemcinctus	Dasypus_novemcinctus	GCF_000208655.1_Dasnov3.0	train_val
Delphinapterus leucas	Delphinapterus_leucas	GCF_002288925.2_ASM228892v3	train_val
Desmodus rotundus	Desmodus_rotundus	GCF_002940915.1_ASM294091v2	test (used)
Dipodomys ordii	Dipodomys_ordii	GCF_000151885.1_Dord_2.0	train_val
Dipodomys spectabilis	Dipodomys_spectabilis	GCF_019054845.1_ASM1905484v1	test
Dromiciops gliroides	Dromiciops_gliroides	GCF_019393635.1_mDroGli1.pri	train_val
Echinops telfairi	Echinops_telfairi	GCF_000313985.2_ASM31398v2	train_val
Elephantulus edwardii	Elephantulus_edwardii	GCF_000299155.1_EleEdw1.0	test
Enhydra lutris	Enhydra_lutris	GCF_002288905.1_ASM228890v2	test
Eptesicus fuscus	Eptesicus_fuscus	GCF_000308155.1_EptFus1.0	test
Equus asinus	Equus_asinus	GCF_016077325.2_ASM1607732v2	train_val
Equus caballus	Equus_caballus	GCF_002863925.1_EquCab3.0	train_val
Equus przewalskii	Equus_przewalskii	GCF_000696695.1_Burgud	test
Erinaceus europaeus	Erinaceus_europaeus	GCF_000296755.1_EriEur2.0	train_val
Eumetopias jubatus	Eumetopias_jubatus	GCF_004028035.1_ASM402803v1	test
Felis catus	Felis_catus	GCF_018350175.1_F.catus_Fca126_mat1.0	train_val
Fukomys damarensis	Fukomys_damarensis	GCF_012274545.1_DMR_v1.0_HiC	train_val
Galeopterus variegatus	Galeopterus_variegatus	GCF_000696425.1_G_variegatus-3.0.2	test
Globicephala melas	Globicephala_melas	GCF_006547405.1_ASM654740v1	test
Gorilla gorilla	Gorilla_gorilla	GCF_008122165.1_Kamilah_GGO_v0	train_val
Gracilinanus agilis	Gracilinanus_agilis	GCF_016433145.1_AgileGrace	test
Grammomys surdaster	Grammomys_surdaster	GCF_004785775.1_NIH_TR_1.0	test
Halichoerus grypus	Halichoerus_grypus	GCF_012393455.1_Tufts_HGry_1.1	test
Heterocephalus glaber	Heterocephalus_glaber	GCF_000247695.1_HetGla_female_1.0	train_val
Hipposideros armiger	Hipposideros_armiger	GCF_001890085.1_ASM189008v1	train_val
Homo sapiens	Homo_sapiens	GCF_000001405.39_GRCh38.p13	train_val
Hyaena hyaena	Hyaena_hyaena	GCF_003009895.1_ASM300989v1	train_val
Hylobates moloch	Hylobates_moloch	GCF_009828535.2_HMol_V2	train_val
Ictidomys tridecemlineatus	Ictidomys_tridecemlineatus	GCF_016881025.1_HiC_Itri_2	train_val
Jaculus jaculus	Jaculus_jaculus	GCF_020740685.1_mJacJac1.mat.Y.cur	train_val
Lagenorhynchus obliquidens	Lagenorhynchus_obliquidens	GCF_003676395.1_ASM367639v1	test
Lemur catta	Lemur_catta	GCF_020740605.2_mLemCat1.pri	train_val
Leopardus geoffroyi	Leopardus_geoffroyi	GCF_018350155.1_O.geoffroyi_Oge1_pat1.0	test
Leptonychotes weddellii	Leptonychotes_weddellii	GCF_000349705.1_LepWed1.0	train_val
Lipotes vexillifer	Lipotes_vexillifer	GCF_000442215.1_Lipotes_vexillifer_v1	train_val
Lontra canadensis	Lontra_canadensis	GCF_010015895.1_GSC_riverotter_1.0	train_val
Loxodonta africana	Loxodonta_africana	GCF_000001905.1_Loxafr3.0	train_val
Lynx canadensis	Lynx_canadensis	GCF_007474595.2_mLynCan4.pri.v2	train_val
Macaca fascicularis	Macaca_fascicularis	GCF_012559485.2_MFA1912RKSv2	train_val
Macaca mulatta	Macaca_mulatta	GCF_003339765.1_Mmul_10	train_val
Macaca nemestrina	Macaca_nemestrina	GCF_000956065.1_Mnem_1.0	train_val
Mandrillus leucophaeus	Mandrillus_leucophaeus	GCF_000951045.1_Mleu.le_1.0	train_val
Manis javanica	Manis_javanica	GCF_014570535.1_YNU_ManJav_2.0	test

scientific	ID	version	split
Manis pentadactyla	Manis_pentadactyla	GCF_014570555.1_YNU_ManPten_2.0	test
Marmota flaviventris	Marmota_flaviventris	GCF_003676075.2_GSC_YBM_2.0	test
Marmota marmota	Marmota_marmota	GCF_001458135.1_marMar2.1	train_val
Marmota monax	Marmota_monax	GCF_021218885.1_Marmota_monax_Labrador192_V1.0	test
Mastomys coucha	Mastomys_coucha	GCF_008632895.1_UCSF_Mcou_1	test
Meles meles	Meles_meles	GCF_922984935.1_mMelMel3.1_paternal_haplotype	train_val
Meriones unguiculatus	Meriones_unguiculatus	GCF_002204375.1_MunDraft-v1.0	train_val
Mesocricetus auratus	Mesocricetus_auratus	GCF_017639785.1_BCM_Maur_2.0	train_val
Microcebus murinus	Microcebus_murinus	GCF_000165445.2_Mmur_3.0	train_val
Microtus ochrogaster	Microtus_ochrogaster	GCF_000317375.1_MicOch1.0	train_val
Microtus oregoni	Microtus_oregoni	GCF_018167655.1_Mior012	train_val
Miniopterus natalensis	Miniopterus_natalensis	GCF_001595765.1_Mnat.v1	test
Mirounga angustirostris	Mirounga_angustirostris	GCF_021288785.1_ASM2128878v2	test
Mirounga leonina	Mirounga_leonina	GCF_011800145.1_KU_Mleo_1.0	train_val
Molossus molossus	Molossus_molossus	GCF_014108415.1_mMolMol1.p	test
Monodelphis domestica	Monodelphis_domestica	GCF_000002295.2_MonDom5	train_val
Monodon monoceros	Monodon_monoceros	GCF_005190385.1_NGI_Narwhal_1	test
Mus caroli	Mus_caroli	GCF_900094665.1_CAROLI_EIJ_v1.1	train_val
Mus musculus	Mus_musculus	GCF_000001635.27_GRCm39	train_val
Mus pahari	Mus_pahari	GCF_900095145.1_PAHARI_EIJ_v1.1	train_val
Mustela erminea	Mustela_erminea	GCF_009829155.1_mMusErm1.Pri	test
Mustela putorius	Mustela_putorius	GCF_011764305.1_ASM1176430v1.1	train_val
Myotis brandtii	Myotis_brandtii	GCF_000412655.1_ASM41265v1	train_val
Myotis davidii	Myotis_davidii	GCF_000327345.1_ASM32734v1	test (used)
Myotis lucifugus	Myotis_lucifugus	GCF_000147115.1_Myoluc2.0	train_val
Myotis myotis	Myotis_myotis	GCF_014108235.1_mMyoMyo1.p	train_val
Nannospalax galili	Nannospalax_galili	GCF_000622305.1_S.galili_v1.0	train_val
Neogale vison	Neogale_vison	GCF_020171115.1_ASM_NN_V1	train_val
Neomonachus schauinslandi	Neomonachus_schauinslandi	GCF_002201575.2_ASM220157v2	train_val
Neophocaena asiaeorientalis	Neophocaena_asiaeorientalis	GCF_003031525.2_Neophocaena_asiaeorientalis_V1	test
Nomascus leucogenys	Nomascus_leucogenys	GCF_006542625.1_Asia_NLE_v1	train_val
Ochotona curzoniae	Ochotona_curzoniae	GCF_017591425.1_NIBS_Ocur_1.0	train_val
Ochotona princeps	Ochotona_princeps	GCF_014633375.1_OchPri4.0	train_val
Octodon degus	Octodon_degus	GCF_000260255.1_OctDeg1.0	train_val
Odobenus rosmarus	Odobenus_rosmarus	GCF_000321225.1_Oros_1.0	train_val
Odocoileus virginianus	Odocoileus_virginianus	GCF_002102435.1_Ovir.te_1.0	test
Onychomys torridus	Onychomys_torridus	GCF_903995425.1_mOncTor1.1	test
Orcinus orca	Orcinus_orca	GCF_000331955.2_Oorc_1.1	train_val
Ornithorhynchus anatinus	Ornithorhynchus_anatinus	GCF_004115215.2_mOrnAna1.pri.v4	train_val
Orycteropus afer	Orycteropus_afer	GCF_000298275.1_OryAfe1.0	test
Oryctolagus cuniculus	Oryctolagus_cuniculus	GCF_000003625.3_OryCun2.0	train_val
Oryx dammah	Oryx_dammah	GCF_014754425.2_SCBI_Odam_1.1	test
Otolemur garnettii	Otolemur_garnettii	GCF_000181295.1_OtoGar3	train_val
Ovis aries	Ovis_aries	GCF_016772045.1_ARS-UI_Ramb_v2.0	train_val
Pan paniscus	Pan_paniscus	GCF_013052645.1_Mhudiblu_PPA_v0	train_val
Panthera leo	Panthera_leo	GCF_018350215.1_P.leo_Ple1_pat1.1	train_val
Panthera pardus	Panthera_pardus	GCF_001857705.1_PanPar1.0	train_val
Panthera tigris	Panthera_tigris	GCF_018350195.1_P.tigris_Pti1_mat1.1	train_val
Pan troglodytes	Pan_troglodytes	GCF_002880755.1_Clint_PTRv2	train_val
Papio anubis	Papio_anubis	GCF_008728515.1_Panubis1.0	train_val
Peromyscus leucopus	Peromyscus_leucopus	GCF_004664715.2_UCI_PerLeu_2.1	test
Peromyscus maniculatus	Peromyscus_maniculatus	GCF_003704035.1_HU_Pman_2.1.3	train_val
Phascolarctos cinereus	Phascolarctos_cinereus	GCF_002099425.1_phaCin_unsw_v4.1	train_val
Phoca vitulina	Phoca_vitulina	GCF_004348235.1_GSC_HSeal_1.0	train_val
Phocoena sinus	Phocoena_sinus	GCF_008692025.1_mPhoSin1.pri	train_val
Phyllostomus discolor	Phyllostomus_discolor	GCF_004126475.2_mPhyDis1.pri.v3	train_val
Phyllostomus hastatus	Phyllostomus_hastatus	GCF_019186645.2_TTU_PhHast_1.1	train_val
Physeter catodon	Physeter_catodon	GCF_002837175.2_ASM283717v2	test
Piliocolobus tephrosceles	Piliocolobus_tephrosceles	GCF_002776525.3_ASM277652v3	train_val
Pipistrellus kuhlii	Pipistrellus_kuhlii	GCF_014108245.1_mPipKuh1.p	train_val
Pongo abelii	Pongo_abelii	GCF_002880775.1_Susie_PABv2	train_val
Prionailurus bengalensis	Prionailurus_bengalensis	GCF_016509475.1_Fcat_Pben_1.1_paternal_pri	test
Propithecus coquereli	Propithecus_coquereli	GCF_000956105.1_Pcoq_1.0	train_val
Pteropus alecto	Pteropus_alecto	GCF_000325575.1_ASM32557v1	train_val
Pteropus giganteus	Pteropus_giganteus	GCF_902729225.1_Ma_sr-lr_union100	test
Pteropus vampyrus	Pteropus_vampyrus	GCF_000151845.1_Pvam_2.0	train_val
Puma concolor	Puma_concolor	GCF_003327715.1_PumCon1.0	train_val
Puma yagouaroundi	Puma_yagouaroundi	GCF_014898765.1_PumYag	train_val
Rattus norvegicus	Rattus_norvegicus	GCF_015227675.2_mRatBN7.2	train_val
Rattus rattus	Rattus_rattus	GCF_011064425.1_Rrattus_CSIRO_v1	train_val
Rhinolophus ferrumequinum	Rhinolophus_ferrumequinum	GCF_004115265.1_mRhiFer1_v1.p	test
Rhinopithecus bieti	Rhinopithecus_bieti	GCF_001698545.1_ASM169854v1	train_val
Rhinopithecus roxellana	Rhinopithecus_roxellana	GCF_007565055.1_ASM756505v1	train_val
Rousettus aegyptiacus	Rousettus_aegyptiacus	GCF_014176215.1_mRouAeg1.p	test
Saimiri boliviensis	Saimiri_boliviensis	GCF_016699345.1_BCM_Sbol_2.0	train_val
Sapajus apella	Sapajus_apella	GCF_009761245.1_GSC_monkey_1.0	test
Sarcophilus harrisii	Sarcophilus_harrisii	GCF_902635505.1_mSarHar1.11	train_val
Sorex araneus	Sorex_araneus	GCF_000181275.1_SorAra2.0	train_val
Sturnira hondurensis	Sturnira_hondurensis	GCF_014824575.2_WHU_Shon_v2.1	train_val
Suricata suricatta	Suricata_suricatta	GCF_006229205.1_meerkat_22Aug2017_6uvM2_HiC	test

scientific	ID	version	split
Sus scrofa	Sus_scrofa	GCF_000003025.6_Sscrofa11.1	train_val
Tachyglossus aculeatus	Tachyglossus_aculeatus	GCF_015852505.1_mTacAcu1.pri	train_val
Talpa occidentalis	Talpa_occidentalis	GCF_014898055.1_MPIMG_talOcc4	train_val
Theropithecus gelada	Theropithecus_gelada	GCF_003255815.1_Tgel_1.0	train_val
Trachypithecus francoisi	Trachypithecus_francoisi	GCF_009764315.1_Tfra_2.0	test
Trichechus manatus	Trichechus_manatus	GCF_000243295.1_TriManLat1.0	test
Trichosurus vulpecula	Trichosurus_vulpecula	GCF_011100635.1_mTriVul1.pri	test
Tupaia chinensis	Tupaia_chinensis	GCF_000334495.1_TupChi_1.0	train_val
Tursiops truncatus	Tursiops_truncatus	GCF_011762595.1_mTurTru1.mat.Y	train_val
Urocitellus parryii	Urocitellus_parryii	GCF_003426925.1_ASM342692v1	train_val
Ursus americanus	Ursus_americanus	GCF_020975775.1_gsc_jax_bbear_1.0	train_val
Ursus arctos	Ursus_arctos	GCF_003584765.2_ASM358476v2	train_val
Ursus maritimus	Ursus_maritimus	GCF_017311325.1_ASM1731132v1	train_val
Vicugna pacos	Vicugna_pacos	GCF_000164845.3_VicPac3.1	train_val
Vombatus ursinus	Vombatus_ursinus	GCF_900497805.2_bare-nosed_wombat_genome_assembly	train_val
Vulpes lagopus	Vulpes_lagopus	GCF_018345385.1_ASM1834538v1	test
Vulpes vulpes	Vulpes_vulpes	GCF_003160815.1_VulVul2.2	train_val
Zalophus californianus	Zalophus_californianus	GCF_009762305.2_mZalCal1.pri.v2	test
Acanthisitta chloris	Acanthisitta_chloris	GCF_000695815.1_ASM69581v1	test
Acanthochromis polyacanthus	Acanthochromis_polyacanthus	GCF_002109545.1_ASM210954v1	train_val
Acanthopagrus latus	Acanthopagrus_latus	GCF_904848185.1_fAcaLat1.1	train_val
Acipenser ruthenus	Acipenser_ruthenus	GCF_010645085.1_ASM1064508v1	test
Alligator mississippiensis	Alligator_mississippiensis	GCF_000281125.3_ASM28112v4	train_val
Alligator sinensis	Alligator_sinensis	GCF_000455745.1_ASM45574v1	test
Alosa sapidissima	Alosa_sapidissima	GCF_018492685.1_fAloSap1.pri	train_val
Amblyraja radiata	Amblyraja_radiata	GCF_010909765.2_sAmbRad1.1.pri	test
Amphiprion ocellaris	Amphiprion_ocellaris	GCF_002776465.1_AmpOce1.0	train_val
Anabas testudineus	Anabas_testudineus	GCF_900324465.2_fAnaTes1.2	train_val
Anarrhichthys ocellatus	Anarrhichthys_ocellatus	GCF_004355925.1_GSC_Weel_1.0	train_val
Anas platyrhynchos	Anas_platyrhynchos	GCF_015476345.1_ZJU1.0	train_val
Anguilla anguilla	Anguilla_anguilla	GCF_013347855.1_fAngAng1.pri	test
Anolis carolinensis	Anolis_carolinensis	GCF_000090745.1_AnoCar2.0	train_val
Anser cygnoides	Anser_cygnoides	GCF_000971095.1_AnsCyg_PRJNA183603_v1.0	train_val
Antrostomus carolinensis	Antrostomus_carolinensis	GCF_000700745.1_ASM70074v1	train_val
Apaloderma vittatum	Apaloderma_vittatum	GCF_000703405.1_ASM70340v1	test
Aptenodytes forsteri	Aptenodytes_forsteri	GCF_000699145.1_ASM69914v1	test
Apteryx mantelli	Apteryx_mantelli	GCF_001039765.1_AptMant0	test
Apteryx rowi	Apteryx_rowi	GCF_003343035.1_aptRow1	train_val
Aquila chrysaetos	Aquila_chrysaetos	GCF_900496995.4_bAquChr1.4	train_val
Archocentrus centrarchus	Archocentrus_centrarchus	GCF_007364275.1_fArcCen1	train_val
Astatotilapia calliptera	Astatotilapia_calliptera	GCF_900246225.1_fAstCal1.2	train_val
Astyanax mexicanus	Astyanax_mexicanus	GCF_000372685.2_Astyanax_mexicanus-2.0	train_val
Athene cunicularia	Athene_cunicularia	GCF_003259725.1_athCun1	train_val
Austrofundulus limnaeus	Austrofundulus_limnaeus	GCF_001266775.1_Austrofundulus_limnaeus-1.0	train_val
Aythya fuligula	Aythya_fuligula	GCF_009819795.1_bAytFul2.pri	test
Balearica regulorum	Balearica_regulorum	GCF_000709895.1_ASM70989v1	test
Betta splendens	Betta_splendens	GCF_900634795.3_fBetSpl5.3	train_val
Boleophthalmus pectinirostris	Boleophthalmus_pectinirostris	GCF_000788275.1_BP.fa	train_val
Buceros rhinoceros	Buceros_rhinoceros	GCF_000710305.1_ASM71030v1	test
Bufo bufo	Bufo_bufo	GCF_905171765.1_aBufBuf1.1	test
Bufo gargarizans	Bufo_gargarizans	GCF_014858855.1_ASM1485885v1	test
Calidris pugnax	Calidris_pugnax	GCF_001431845.1_ASM143184v1	train_val
Callorhinchus milii	Callorhinchus_milii	GCF_018977255.1_IMCB_Cmil_1.0	train_val
Calypte anna	Calypte_anna	GCF_003957555.1_bCalAnn1_v1.p	train_val
Camarhynchus parvulus	Camarhynchus_parvulus	GCF_901933205.1_STF_HiC	train_val
Carassius auratus	Carassius_auratus	GCF_003368295.1_ASM336829v1	train_val
Carcharodon carcharias	Carcharodon_carcharias	GCF_017639515.1_sCarCar2.pri	test
Cariama cristata	Cariama_cristata	GCF_000690535.1_ASM69053v1	train_val
Catharus ustulatus	Catharus_ustulatus	GCF_009819885.2_bCatUst1.pri.v2	train_val
Centrocercus urophasianus	Centrocercus_urophasianus	GCF_019232065.1_USGS_Curo_1.0	train_val
Chaetura pelagica	Chaetura_pelagica	GCF_000747805.1_ChaPel_1.0	train_val
Chanos chanos	Chanos_chanos	GCF_902362185.1_fChaCha1.1	test
Charadrius vociferus	Charadrius_vociferus	GCF_000708025.1_ASM70802v2	train_val
Cheilinus undulatus	Cheilinus_undulatus	GCF_018320785.1_ASM1832078v1	train_val
Chelmon rostratus	Chelmon_rostratus	GCF_017976325.1_fCheRos1.pri	test
Chelonia mydas	Chelonia_mydas	GCF_015237465.2_rCheMyd1.pri.v2	test
Chelonoidis abingdonii	Chelonoidis_abingdonii	GCF_003597395.1_ASM359739v1	train_val
Chiloscyllium plagiosum	Chiloscyllium_plagiosum	GCF_004010195.1_ASM401019v2	train_val
Chiroxiphia lanceolata	Chiroxiphia_lanceolata	GCF_009829145.1_bChiLan1.pri	test (used)
Chlamydotis macqueenii	Chlamydotis_macqueenii	GCF_000695195.1_ASM69519v1	train_val
Chrysemys picta	Chrysemys_picta	GCF_000241765.4_Chrysemys_picta_BioNano-3.0.4	train_val
Clupea harengus	Clupea_harengus	GCF_900700415.2_Ch_v2.0.2	train_val
Colius striatus	Colius_striatus	GCF_000690715.1_ASM69071v1	test
Colossoma macropomum	Colossoma_macropomum	GCF_904425465.1_Colossoma_macropomum	test
Columba livia	Columba_livia	GCF_000337935.1_Cliv_1.0	train_val
Corapipo altera	Corapipo_altera	GCF_003945725.1_ASM394572v1	test
Coregonus clupeaformis	Coregonus_clupeaformis	GCF_020615455.1_ASM2061545v1	test
Corvus brachyrhynchos	Corvus_brachyrhynchos	GCF_000691975.1_ASM69197v1	test
Corvus cornix	Corvus_cornix	GCF_000738735.5_ASM73873v5	test
Corvus kubaryi	Corvus_kubaryi	GCF_017639235.1_C.kubaryi_AGA036_p1.0	test

scientific	ID	version	split
Corvus moneduloides	Corvus_moneduloides	GCF_009650955.1_bCorMon1.pri	train_val
Cottoperca gobio	Cottoperca_gobio	GCF_900634415.1_fCotGob3.1	train_val
Coturnix japonica	Coturnix_japonica	GCF_001577835.2_Coturnix_japonica_2.1	train_val
Crocodylus porosus	Crocodylus_porosus	GCF_001723895.1_CroPor_comp1	train_val
Crotalus tigris	Crotalus_tigris	GCF_016545835.1_ASM1654583v1	test
Cuculus canorus	Cuculus_canorus	GCF_000709325.1_ASM70932v1	test
Cyanistes caeruleus	Cyanistes_caeruleus	GCF_002901205.1_cyaCae2	train_val
Cyclopterus lumpus	Cyclopterus_lumpus	GCF_009769545.1_fCycLum1.pri	train_val
Cygnus atratus	Cygnus_atratus	GCF_013377495.1_Cygnus_atratus_primary_v1.0	train_val
Cygnus olor	Cygnus_olor	GCF_009769625.2_bCygOlo1.pri.v2	train_val
Cynoglossus semilaevis	Cynoglossus_semilaevis	GCF_000523025.1_Cse_v1.0	train_val
Cyprinodon tularosa	Cyprinodon_tularosa	GCF_016077235.1_ASM1607723v1	train_val
Cyprinodon variegatus	Cyprinodon_variegatus	GCF_000732505.1_C_variegatus-1.0	train_val
Cyprinus carpio	Cyprinus_carpio	GCF_018340385.1_ASM1834038v1	train_val
Danio rerio	Danio_rerio	GCF_000002035.6_GRCz11	train_val
Denticeps clupeoides	Denticeps_clupeoides	GCF_900700375.1_fDenClu1.1	train_val
Dermochelys coriacea	Dermochelys_coriacea	GCF_009764565.3_rDerCor1.pri.v4	test
Dromaius novaehollandiae	Dromaius_novaehollandiae	GCF_003342905.1_droNov1	train_val
Dryobates pubescens	Dryobates_pubescens	GCF_000699005.1_ASM69900v1	test
Echeneis naucrates	Echeneis_naucrates	GCF_900963305.1_fEcheNa1.1	train_val
Egretta garzetta	Egretta_garzetta	GCF_000687185.1_ASM68718v1	test
Electrophorus electricus	Electrophorus_electricus	GCF_013358815.1_fEleEle1.pri	train_val
Empidonax traillii	Empidonax_traillii	GCF_003031625.1_ASM303162v1	test
Epinephelus lanceolatus	Epinephelus_lanceolatus	GCF_005281545.1_ASM528154v1	test
Erpetoichthys calabaricus	Erpetoichthys_calabaricus	GCF_900747795.1_fErpCal1.1	train_val
Esox lucius	Esox_lucius	GCF_011004845.1_fEsoLuc1.pri	train_val
Etheostoma cragini	Etheostoma_cragini	GCF_013103735.1_CSU_Ecrag_1.0	test
Etheostoma spectabile	Etheostoma_spectabile	GCF_008692095.1_UIUC_Espe_1.0	train_val
Eurypyga helias	Eurypyga_helias	GCF_000690775.1_ASM69077v1	train_val
Falco cherrug	Falco_cherrug	GCF_000337975.1_F_cherrug_v1.0	test
Falco naumanni	Falco_naumanni	GCF_017639655.2_bFalNau1.pat	test (used)
Falco peregrinus	Falco_peregrinus	GCF_000337955.1_F_peregrinus_v1.0	train_val
Falco rusticolus	Falco_rusticolus	GCF_015220075.1_bFalRus1.pri	test
Ficedula albicollis	Ficedula_albicollis	GCF_000247815.1_FicAlb1.5	train_val
Fulmarus glacialis	Fulmarus_glacialis	GCF_000690835.1_ASM69083v1	train_val
Fundulus heteroclitus	Fundulus_heteroclitus	GCF_011125445.2_MU-UCD_Fhet_4.1	train_val
Gadus morhua	Gadus_morhua	GCF_902167405.1_gadMor3.0	train_val
Gallus gallus	Gallus_gallus	GCF_016699485.2_bGalGal1.mat.broiler.GRCg7b	train_val
Gambusia affinis	Gambusia_affinis	GCF_019740435.1_SWU_Gaff_1.0	train_val
Gasterosteus aculeatus	Gasterosteus_aculeatus	GCF_016920845.1_GAculeatus_UGA_version5	train_val
Gavialis gangeticus	Gavialis_gangeticus	GCF_001723915.1_GavGan_comp1	train_val
Gavia stellata	Gavia_stellata	GCF_000690875.1_ASM69087v1	test
Gekko japonicus	Gekko_japonicus	GCF_001447785.1_Gekko_japonicus_V1.1	test
Geospiza fortis	Geospiza_fortis	GCF_000277835.1_GeoFor_1.0	train_val
Geotrypetes seraphini	Geotrypetes_seraphini	GCF_902459505.1_aGeoSer1.1	train_val
Gopherus evgoodei	Gopherus_evgoodei	GCF_007399415.2_rGopEvg1_v1.p	train_val
Gouania willdenowi	Gouania_willdenowi	GCF_900634775.1_fGouWil2.1	train_val
Gymnodraco acuticeps	Gymnodraco_acuticeps	GCF_902827175.1_fGymAcu1.1	test
Haliaeetus albicilla	Haliaeetus_albicilla	GCF_000691405.1_ASM69140v1	train_val
Haliaeetus leucocephalus	Haliaeetus_leucocephalus	GCF_000737465.1_Haliaeetus_leucocephalus-4.0	test
Haplochromis burtoni	Haplochromis_burtoni	GCF_018398535.1_NCSU_Asbu1	train_val
Hippocampus comes	Hippocampus_comes	GCF_001891065.1_H_comes_QL1_v1	train_val
Hippoglossus hippoglossus	Hippoglossus_hippoglossus	GCF_009819705.1_fHipHip1.pri	test
Hippoglossus stenolepis	Hippoglossus_stenolepis	GCF_013339905.1_IPHC_HiSten_1.0	train_val
Hirundo rustica	Hirundo_rustica	GCF_015227805.1_bHirRus1.pri.v2	train_val
Ictalurus punctatus	Ictalurus_punctatus	GCF_001660625.1_IpCoco_1.2	train_val
Kryptolebias marmoratus	Kryptolebias_marmoratus	GCF_001649575.2_ASM164957v2	train_val
Labrus bergylta	Labrus_bergylta	GCF_900080235.1_BallGen_V1	train_val
Lacerta agilis	Lacerta_agilis	GCF_009819535.1_rLacAgi1.pri	train_val
Lagopus leucura	Lagopus_leucura	GCF_019238085.1_USGS_WTPT01	test (used)
Larimichthys crocea	Larimichthys_crocea	GCF_000972845.2_L_crocea_2.0	train_val
Lates calcarifer	Lates_calcarifer	GCF_001640805.1_ASM164080v1	train_val
Latimeria chalumnae	Latimeria_chalumnae	GCF_000225785.1_LatCha1	train_val
Lepidothrix coronata	Lepidothrix_coronata	GCF_001604755.1_Lepidothrix_coronata-1.0	train_val
Lepisosteus oculatus	Lepisosteus_oculatus	GCF_000242695.1_LepOcu1	train_val
Leptosomus discolor	Leptosomus_discolor	GCF_000691785.1_ASM69178v1	train_val
Lonchura striata	Lonchura_striata	GCF_005870125.1_lonStrDom2	train_val
Manacus vitellinus	Manacus_vitellinus	GCF_001715985.3_ASM171598v3	train_val
Mastacembelus armatus	Mastacembelus_armatus	GCF_900324485.2_fMasArm1.2	train_val
Mauremys mutica	Mauremys_mutica	GCF_020497125.1_ASM2049712v1	train_val
Mauremys reevesii	Mauremys_reevesii	GCF_016161935.1_ASM1616193v1	train_val
Maylandia zebra	Maylandia_zebra	GCF_000238955.4_M_zebra_UMD2a	train_val
Megalops cyprinoides	Megalops_cyprinoides	GCF_013368585.1_fMegCyp1.pri	train_val
Melanotaenia boesemani	Melanotaenia_boesemani	GCF_017639745.1_fMelBoe1.pri	train_val
Meleagris gallopavo	Meleagris_gallopavo	GCF_000146605.3_Turkey_5.1	train_val
Melopsittacus undulatus	Melopsittacus_undulatus	GCF_012275295.1_bMelUnd1.mat.Z	train_val
Merops nubicus	Merops_nubicus	GCF_000691845.1_ASM69184v1	test
Mesitornis unicolor	Mesitornis_unicolor	GCF_000695765.1_ASM69576v1	train_val
Microcaecilia unicolor	Microcaecilia_unicolor	GCF_901765095.1_aMicUni1.1	train_val
Micropterus dolomieu	Micropterus_dolomieu	GCF_021292245.1_ASM2129224v1	train_val

scientific	ID	version	split
Micropterus salmoides	Micropterus_salmoides	GCF_014851395.1_ASM1485139v1	train_val
Molothrus ater	Molothrus_ater	GCF_012460135.1_BPBGC_Mater_1.0	train_val
Monopterus albus	Monopterus_albus	GCF_001952655.1_M_albus_1.0	train_val
Morone saxatilis	Morone_saxatilis	GCF_004916995.1_NCSU_SB_2.0	train_val
Motacilla alba	Motacilla_alba	GCF_015832195.1_Motacilla_alba_V1.0_pri	train_val
Myripristis murdjan	Myripristis_murdjan	GCF_902150065.1_fMyrMur1.1	train_val
Nanorana parkeri	Nanorana_parkeri	GCF_000935625.1_ASM93562v1	test
Nematolebias whitei	Nematolebias_whitei	GCF_014905685.2_NemWhi1	train_val
Neolamprologus brichardi	Neolamprologus_brichardi	GCF_000239395.1_NeoBri1.0	train_val
Neopelma chrysocephalum	Neopelma_chrysocephalum	GCF_003984885.1_ASM398488v2	test
Nestor notabilis	Nestor_notabilis	GCF_000696875.1_ASM69687v1	train_val
Nipponia nippon	Nipponia_nippon	GCF_000708225.1_ASM70822v1	train_val
Notechis scutatus	Notechis_scutatus	GCF_900518725.1_TS10Xv2-PRI	train_val
Nothobranchius furzeri	Nothobranchius_furzeri	GCF_001465895.1_Nfu_20140520	train_val
Nothoprocta perdicaria	Nothoprocta_perdicaria	GCF_003342845.1_notPer1	train_val
Notolabrus celidotus	Notolabrus_celidotus	GCF_009762535.1_fNotCel1.pri	test
Notothenia coriiceps	Notothenia_coriiceps	GCF_000735185.1_NC01	test
Numida meleagris	Numida_meleagris	GCF_002078875.1_NumMel1.0	train_val
Oncorhynchus gorbuscha	Oncorhynchus_gorbuscha	GCF_021184085.1_OgorEven_v1.0	test
Oncorhynchus keta	Oncorhynchus_keta	GCF_012931545.1_Oket_V1	train_val
Oncorhynchus kisutch	Oncorhynchus_kisutch	GCF_002021735.2_Okis_V2	test
Oncorhynchus mykiss	Oncorhynchus_mykiss	GCF_013265735.2_USDA_OmykA_1.1	test
Oncorhynchus nerka	Oncorhynchus_nerka	GCF_006149115.1_Oner_1.0	train_val
Oncorhynchus tshawytscha	Oncorhynchus_tshawytscha	GCF_018296145.1_Otsh_v2.0	test
Onychostruthus taczanowskii	Onychostruthus_taczanowskii	GCF_017590055.1_ASM1759005v1	test
Opisthocomus hoazin	Opisthocomus_hoazin	GCF_000692075.1_ASM69207v1	test (used)
Oreochromis aureus	Oreochromis_aureus	GCF_013358895.1_ZZ_aureus	test
Oreochromis niloticus	Oreochromis_niloticus	GCF_001858045.2_O_niloticus_UMD_NMBU	train_val
Oryzias latipes	Oryzias_latipes	GCF_002234675.1_ASM223467v1	train_val
Oryzias melastigma	Oryzias_melastigma	GCF_002922805.2_ASM292280v2	train_val
Oxyura jamaicensis	Oxyura_jamaicensis	GCF_011077185.1_BPBGC_Ojam_1.0	train_val
Pangasianodon hypophthalmus	Pangasianodon_hypophthalmus	GCF_009078355.1_GENO_Phyp_1.0	train_val
Pantherophis guttatus	Pantherophis_guttatus	GCF_001185365.1_UNIGE_PanGut_3.0	train_val
Paralichthys olivaceus	Paralichthys_olivaceus	GCF_001970005.1_Flounder_ref_guided_V1.0	test
Parambassis ranga	Parambassis_ranga	GCF_900634625.1_fParRan2.1	train_val
Paramormyrops kingsleyae	Paramormyrops_kingsleyae	GCF_002872115.1_PKINGS_0.1	train_val
Parus major	Parus_major	GCF_001522545.3_Parus_major1.1	train_val
Passer montanus	Passer_montanus	GCF_014805655.1_ASM1480565v1	train_val
Pelecanus crispus	Pelecanus_crispus	GCF_000687375.1_ASM68737v1	train_val
Pelodiscus sinensis	Pelodiscus_sinensis	GCF_000230535.1_PelSin_1.0	train_val
Perca flavescens	Perca_flavescens	GCF_004354835.1_PFLA_1.0	train_val
Perca fluviatilis	Perca_fluviatilis	GCF_010015445.1_GENO_Pfluv_1.0	train_val
Periophthalmus magnuspinnatus	Periophthalmus_magnuspinnatus	GCF_009829125.1_fPerMag1.pri	train_val
Petromyzon marinus	Petromyzon_marinus	GCF_010993605.1_kPetMar1.pri	train_val
Phaethon lepturus	Phaethon_lepturus	GCF_000687285.1_ASM68728v1	train_val
Phalacrocorax carbo	Phalacrocorax_carbo	GCF_000708925.1_ASM70892v1	train_val
Phasianus colchicus	Phasianus_colchicus	GCF_004143745.1_ASM414374v1	test
Pimephales promelas	Pimephales_promelas	GCF_016745375.1_EPA_FHM_2.0	test
Pipra filicauda	Pipra_filicauda	GCF_003945595.2_ASM394559v2	train_val
Plectropomus leopardus	Plectropomus_leopardus	GCF_008729295.1_YSFRI_Pleo_2.0	test
Podarcis muralis	Podarcis_muralis	GCF_004329235.1_PodMur_1.0	test
Poecilia formosa	Poecilia_formosa	GCF_000485575.1_Poecilia_formosa-5.1.2	train_val
Poecilia latipinna	Poecilia_latipinna	GCF_001443285.1_P_latipinna-1.0	train_val
Poecilia mexicana	Poecilia_mexicana	GCF_001443325.1_P_mexicana-1.0	train_val
Poecilia reticulata	Poecilia_reticulata	GCF_000633615.1_Guppy_female_1.0_MT	train_val
Pogona vitticeps	Pogona_vitticeps	GCF_900067755.1_pvi1.1	train_val
Polyodon spathula	Polyodon_spathula	GCF_017654505.1_ASM1765450v1	test
Polypterus senegalus	Polypterus_senegalus	GCF_016835505.1_ASM1683550v1	train_val
Protobothrops mucrosquamatus	Protobothrops_mucrosquamatus	GCF_001527695.2_P.Mucros_1.0	train_val
Protopterus annectens	Protopterus_annectens	GCF_019279795.1_PAN1.0	train_val
Pseudochaenichthys georgianus	Pseudochaenichthys_georgianus	GCF_902827115.1_fPseGeo1.1	train_val
Pseudonaja textilis	Pseudonaja_textilis	GCF_900518735.1_EBS10Xv2-PRI	test (used)
Pseudopodoces humilis	Pseudopodoces_humilis	GCF_000331425.1_PseHum1.0	train_val
Pterocles gutturalis	Pterocles_gutturalis	GCF_000699245.1_ASM69924v1	train_val
Pundamilia nyererei	Pundamilia_nyererei	GCF_000239375.1_PunNye1.0	train_val
Pungitius pungitius	Pungitius_pungitius	GCF_902500615.1_NSP_V7	test
Puntigrus tetrazona	Puntigrus_tetrazona	GCF_018831695.1_ASM1883169v1	train_val
Pygocentrus nattereri	Pygocentrus_nattereri	GCF_015220715.1_fPygNat1.pri	train_val
Pygoscelis adeliae	Pygoscelis_adeliae	GCF_000699105.1_ASM69910v1	train_val
Pyrgilauda ruficollis	Pyrgilauda_ruficollis	GCF_017590135.1_ASM1759013v1	train_val
Python bivittatus	Python_bivittatus	GCF_000186305.1_Python_molurus_bivittatus-5.0.2	test
Rana temporaria	Rana_temporaria	GCF_905171775.1_aRanTem1.1	train_val
Rhinatrema bivittatum	Rhinatrema_bivittatum	GCF_901001135.1_aRhiBiv1.1	test
Rhincodon typus	Rhincodon_typus	GCF_001642345.1_ASM164234v2	test (used)
Salarias fasciatus	Salarias_fasciatus	GCF_902148845.1_fSalaFa1.1	train_val
Salmo salar	Salmo_salar	GCF_905237065.1_Ssal_v3.1	train_val
Salmo trutta	Salmo_trutta	GCF_901001165.1_fSalTru1.1	train_val
Salvelinus alpinus	Salvelinus_alpinus	GCF_002910315.2_ASM291031v2	test
Salvelinus namaycush	Salvelinus_namaycush	GCF_016432855.1_SaNama_1.0	train_val
Salvelinus sp IW2-2015	Salvelinus_sp._IW2-2015	GCF_002910315.2_ASM291031v2	train_val

scientific	ID	version	split
Sander lucioperca	Sander_lucioperca	GCF_008315115.2_SLUC_FBN_1.2	train_val
Scatophagus argus	Scatophagus_argus	GCF_020382885.2_fScaArg1.pri	test
Sceloporus undulatus	Sceloporus_undulatus	GCF_019175285.1_SceUnd_v1.1	train_val
Scleropages formosus	Scleropages_formosus	GCF_900964775.1_fSclFor1.1	train_val
Scophthalmus maximus	Scophthalmus_maximus	GCF_013347765.1_ASM1334776v1	train_val
Scyliorhinus canicula	Scyliorhinus_canicula	GCF_902713615.1_sScyCan1.1	train_val
Sebastes umbrosus	Sebastes_umbrosus	GCF_015220745.1_fSebUmb1.pri	train_val
Serinus canaria	Serinus_canaria	GCF_007115625.1_cibio_Scana_2019	train_val
Seriola dumerili	Seriola_dumerili	GCF_002260705.1_Sdu_1.0	train_val
Seriola lalandi	Seriola_lalandi	GCF_002814215.1_Sedor1	train_val
Silurus meridionalis	Silurus_meridionalis	GCF_014805685.1_ASM1480568v1	test
Simochromis diagramma	Simochromis_diagramma	GCF_900408965.1_fSimDia1.1	train_val
Siniperca chuatsi	Siniperca_chuatsi	GCF_020085105.1_ASM2008510v1	train_val
Sinocyclocheilus anshuiensis	Sinocyclocheilus_anshuiensis	GCF_001515605.1_SAMN03320099.WGS_v1.1	test
Sinocyclocheilus grahami	Sinocyclocheilus_grahami	GCF_001515645.1_SAMN03320097.WGS_v1.1	train_val
Sinocyclocheilus rhinocerous	Sinocyclocheilus_rhinocerous	GCF_001515625.1_SAMN03320098_v1.1	train_val
Solea senegalensis	Solea_senegalensis	GCF_019176455.1_IFAPA_SoseM_1	train_val
Sparus aurata	Sparus_aurata	GCF_900880675.1_fSpaAur1.1	test (used)
Sphaeramia orbicularis	Sphaeramia_orbicularis	GCF_902148855.1_fSphaOr1.1	test
Stegastes partitus	Stegastes_partitus	GCF_000690725.1_Stegastes_partitus-1.0.2	train_val
Strigops habroptila	Strigops_habroptila	GCF_004027225.2_bStrHab1.2.pri	test
Struthio camelus	Struthio_camelus	GCF_000698965.1_ASM69896v1	train_val
Sturnus vulgaris	Sturnus_vulgaris	GCF_001447265.1_Sturnus_vulgaris-1.0	train_val
Syngnathus acus	Syngnathus_acus	GCF_901709675.1_fSynAcu1.2	test
Tachysurus fulvidraco	Tachysurus_fulvidraco	GCF_003724035.1_ASM372403v1	train_val
Taeniopygia guttata	Taeniopygia_guttata	GCF_003957565.2_bTaeGut1.4.pri	train_val
Takifugu rubripes	Takifugu_rubripes	GCF_901000725.2_fTakRub1.2	train_val
Tauraco erythrolophus	Tauraco_erythrolophus	GCF_000709365.1_ASM70936v1	train_val
Terrapene carolina	Terrapene_carolina	GCF_002925995.2_T_m_triunguis-2.0	train_val
Thalassophryne amazonica	Thalassophryne_amazonica	GCF_902500255.1_fThaAma1.1	train_val
Thamnophis elegans	Thamnophis_elegans	GCF_009769535.1_rThaEle1.pri	test
Thamnophis sirtalis	Thamnophis_sirtalis	GCF_001077635.1_Thamnophis_sirtalis-6.0	test
Thunnus albacares	Thunnus_albacares	GCF_914725855.1_fThuAlb1.1	train_val
Thunnus maccoyii	Thunnus_maccoyii	GCF_910596095.1_fThuMac1.1	train_val
Tinamus guttatus	Tinamus_guttatus	GCF_000705375.1_ASM70537v2	train_val
Toxotes jaculatrix	Toxotes_jaculatrix	GCF_017976425.1_fToxJac2.pri	train_val
Trachemys scripta	Trachemys_scripta	GCF_013100865.1_CAS_Tse_1.0	test
Trematomus bernacchii	Trematomus_bernacchii	GCF_902827165.1_fTreBer1.1	train_val
Tyto alba	Tyto_alba	GCF_018691265.1_T.alba_DEE_v4.0	test
Varanus komodoensis	Varanus_komodoensis	GCF_004798865.1_ASM479886v1	train_val
Xenopus laevis	Xenopus_laevis	GCF_017654675.1_Xenopus_laevis_v10.1	train_val
Xenopus tropicalis	Xenopus_tropicalis	GCF_000004195.4_UCB_Xtro_10.0	train_val
Xiphias gladius	Xiphias_gladius	GCF_016859285.1_ASM1685928v1	test (used)
Xiphophorus couchianus	Xiphophorus_couchianus	GCF_001444195.1_X_couchianus-1.0	train_val
Xiphophorus hellerii	Xiphophorus_hellerii	GCF_003331165.1_Xiphophorus_hellerii-4.1	test
Xiphophorus maculatus	Xiphophorus_maculatus	GCF_002775205.1_X_maculatus-5.0-male	train_val
Zonotrichia albicollis	Zonotrichia_albicollis	GCF_000385455.1_Zonotrichia_albicollis-1.0.1	train_val
Zootoca vivipara	Zootoca_vivipara	GCF_011800845.1_UG_Zviv_1	test

Table S20: Overview of assessed invertebrate genomes, including their scientific names, identification, and version. The "Split" column indicates whether each species was designated for model training/validation or model evaluation purposes.

scientific	ID	version	split
Acanthaster planci	Acanthaster_planci	GCF_001949145.1_OKI-Apl_1.0	train_val
Acromyrmex echinatior	Acromyrmex_echinatior	GCF_000204515.1_Aech_3.9	train_val
Acropora digitifera	Acropora_digitifera	GCF_000222465.1_Adig_1.1	test
Acropora millepora	Acropora_millepora	GCF_013753865.1_Amil_v2.1	train_val
Actinia tenebrosa	Actinia_tenebrosa	GCF_009602425.1_ASM960242v1	train_val
Acyrthosiphon pisum	Acyrthosiphon_pisum	GCF_005508785.1_pea_aphid_22Mar2018_4r6ur	train_val
Aedes aegypti	Aedes_aegypti	GCF_002204515.2_AaegL5.0	train_val
Aedes albopictus	Aedes_albopictus	GCF_006496715.1_Aalbo_primary.1	test
Aethina tumida	Aethina_tumida	GCF_001937115.1_Atum_1.0	train_val
Agrilus planipennis	Agrilus_planipennis	GCF_000699045.2_Apla_2.0	train_val
Amphibalanus amphitrite	Amphibalanus_amphitrite	GCF_019059575.1_NRLGWU_Aamphi_draft	train_val
Amphimedon queenslandica	Amphimedon_queenslandica	GCF_000090795.1_v1.0	train_val
Amyelois transitella	Amyelois_transitella	GCF_001186105.1_ASM118610v1	train_val
Anneissia japonica	Anneissia_japonica	GCF_011630105.1_ASM1163010v1	test
Anopheles albimanus	Anopheles_albimanus	GCF_013758885.1_VT_AalbS3_pri_1.0	train_val
Anopheles arabiensis	Anopheles_arabiensis	GCF_016920715.1_AaraD3	train_val
Anopheles coluzzii	Anopheles_coluzzii	GCF_016920705.1_AcolMOP1	train_val
Anopheles gambiae	Anopheles_gambiae	GCF_000005575.2_AgamP3	test
Anopheles merus	Anopheles_merus	GCF_017562075.2_AmerM5.1	train_val
Anopheles stephensi	Anopheles_stephensi	GCF_013141755.1_UCI_ANSTEP_V1.0	train_val
Anoplophora glabripennis	Anoplophora_glabripennis	GCF_000390285.2_Agla_2.0	train_val
Aphidius gifuensis	Aphidius_gifuensis	GCF_014905175.1_ASM1490517v1	train_val
Aphis gossypii	Aphis_gossypii	GCF_004010815.1_ASM401081v1	train_val
Apis cerana	Apis_cerana	GCF_001442555.1_ACSNU-2.0	train_val
Apis dorsata	Apis_dorsata	GCF_000469605.1_Apis_dorsata_1.3	test (used)
Apis florea	Apis_florea	GCF_000184785.3_Aflo_1.1	train_val
Apis laboriosa	Apis_laboriosa	GCF_014066325.1_ASM1406632v1	train_val
Apis mellifera	Apis_mellifera	GCF_003254395.2_Amel_HAv3.1	train_val
Aplysia californica	Aplysia_californica	GCF_000002075.1_AplCal3.0	train_val
Aricia agestis	Aricia_agestis	GCF_905147365.1_ilAriAges1.1	test
Asterias rubens	Asterias_rubens	GCF_902459465.1_eAstRub1.3	train_val
Athalia rosae	Athalia_rosae	GCF_000344095.2_Aros_2.0	test
Atta cephalotes	Atta_cephalotes	GCF_000143395.1_Attacep1.0	test (used)
Atta colombica	Atta_colombica	GCF_001594045.1_Acol1.0	train_val
Bactrocera dorsalis	Bactrocera_dorsalis	GCF_000789215.1_ASM78921v2	test
Bactrocera latifrons	Bactrocera_latifrons	GCF_001853355.1_ASM185335v1	test
Bactrocera oleae	Bactrocera_oleae	GCF_001188975.3_MU_Boleae_v2	test
Bactrocera tryoni	Bactrocera_tryoni	GCF_016617805.1_CSIRO_BtryS06_freeze2	train_val
Belonocnema kinseyi	Belonocnema_kinseyi	GCF_010883055.1_B_treatae_v1	train_val
Belonocnema treatae	Belonocnema_treatae	GCF_010883055.1_B_treatae_v1	train_val
Bemisia tabaci	Bemisia_tabaci	GCF_001854935.1_ASM185493v1	train_val
Bicyclus anynana	Bicyclus_anynana	GCF_900239965.1_Bicyclus_anynana_v1.2	train_val
Biomphalaria glabrata	Biomphalaria_glabrata	GCF_000457365.1_ASM45736v1	train_val
Bombus bifarius	Bombus_bifarius	GCF_011952205.1_Bbif_JDL3187	train_val
Bombus impatiens	Bombus_impatiens	GCF_000188095.3_BIMP_2.2	test (used)
Bombus pyrosoma	Bombus_pyrosoma	GCF_014825855.1_ASM1482585v1	train_val
Bombus terrestris	Bombus_terrestris	GCF_000214255.1_Bter_1.0	train_val
Bombus vancouverensis	Bombus_vancouverensis	GCF_011952275.1_Bvanc_JDL1245	train_val
Bombus vosnesenskii	Bombus_vosnesenskii	GCF_011952255.1_Bvos_JDL3184-5_v1.1	test
Bombyx mandarina	Bombyx_mandarina	GCF_003987935.1_ASM398793v1	train_val
Bombyx mori	Bombyx_mori	GCF_014905235.1_Bmori_2016v1.0	train_val
Bradysia coprophila	Bradysia_coprophila	GCF_014529535.1_BU_Bcop_v1	train_val
Branchiostoma belcheri	Branchiostoma_belcheri	GCF_001625305.1_Haploidv18h27	test
Branchiostoma floridae	Branchiostoma_floridae	GCF_000003815.2_Bfl_VNyyK	test
Brugia malayi	Brugia_malayi	GCF_000002995.4_B_malayi-4.0	train_val
Caenorhabditis briggsae	Caenorhabditis_briggsae	GCF_000004555.2_CB4	train_val
Caenorhabditis elegans	Caenorhabditis_elegans	GCF_000002985.6_WBcel235	train_val
Caenorhabditis remanei	Caenorhabditis_remanei	GCF_000149515.1_ASM14951v1	test (used)
Camponotus floridanus	Camponotus_floridanus	GCF_003227725.1_Cflo_v7.5	train_val
Capsaspora owczarzaki	Capsaspora_owczarzaki	GCF_000151315.2_C_owczarzaki_V2	train_val
Centruroides sculpturatus	Centruroides_sculpturatus	GCF_000671375.1_Cexi_2.0	train_val
Cephus cinctus	Cephus_cinctus	GCF_000341935.1_Ccin1	test
Ceratina calcarata	Ceratina_calcarata	GCF_001652005.1_ASM165200v1	test
Ceratitis capitata	Ceratitis_capitata	GCF_000347755.3_Ccap_2.1	train_val
Ceratosolen solmsi	Ceratosolen_solmsi	GCF_000503995.1_CerSol_1.0	test
Chelonus insularis	Chelonus_insularis	GCF_013357705.1_ASM1335770v1	train_val
Chrysoperla carnea	Chrysoperla_carnea	GCF_905475395.1_inChrCarn1.1	test
Cimex lectularius	Cimex_lectularius	GCF_000648675.2_Clec_2.1	train_val
Ciona intestinalis	Ciona_intestinalis	GCF_000224145.3_KH	train_val
Coccinella septempunctata	Coccinella_septempunctata	GCF_907165205.1_icCocSept1.1	train_val
Colias croceus	Colias_croceus	GCF_905220415.1_ilColCroc2.1	test
Colletes gigas	Colletes_gigas	GCF_013123115.1_ASM1312311v1	train_val
Contarinia nasturtii	Contarinia_nasturtii	GCF_009176525.2_AAFC_CNas_1.1	train_val
Copidosoma floridanum	Copidosoma_floridanum	GCF_000648655.2_Cflo_2.0	test
Cotesia glomerata	Cotesia_glomerata	GCF_020080835.1_MPM_Cglom_v2.3	train_val
Crassostrea gigas	Crassostrea_gigas	GCF_902806645.1_cgigas_uk_roslin_v1	train_val
Crassostrea virginica	Crassostrea_virginica	GCF_002022765.2_C_virginica-3.0	test
Cryptotermes secundus	Cryptotermes_secundus	GCF_002891405.2_Csec_1.0	train_val
Ctenocephalides felis	Ctenocephalides_felis	GCF_003426905.1_ASM342690v1	test (used)

scientific	ID	version	split
Culex pipiens	Culex_pipiens	GCF_016801865.1_TS_Cpip_V1	test (used)
Culex quinquefasciatus	Culex_quinquefasciatus	GCF_015732765.1_VPISU_Cqui_1.0_pri_paternal	test
Cyphomyrmex costatus	Cyphomyrmex_costatus	GCF_001594065.1_Ccosl1.0	test
Danaus plexippus	Danaus_plexippus	GCF_009731565.1_Dplex_v4	test
Daphnia magna	Daphnia_magna	GCF_020631705.1_ASM2063170v1.1	train_val
Daphnia pulex	Daphnia_pulex	GCF_021134715.1_ASM2113471v1	test
Daphnia pulicaria	Daphnia_pulicaria	GCF_021234035.1_SC_F0-13Bv2	train_val
Dendroctonus ponderosae	Dendroctonus_ponderosae	GCF_000355655.1_DendPond_male_1.0	train_val
Dendronephthya gigantea	Dendronephthya_gigantea	GCF_004324835.1_DenGig_1.0	train_val
Dermacentor silvarum	Dermacentor_silvarum	GCF_013339745.1_ASM1333974v1	train_val
Dermatophagoides pteronyssinus	Dermatophagoides_pteronyssinus	GCF_001901225.1_ASM190122v2	train_val
Diabrotica virgifera	Diabrotica_virgifera	GCF_003013835.1_Dvir_v2.0	train_val
Diachasma alloeum	Diachasma_alloeum	GCF_001412515.2_Dall2.0	train_val
Diaphorina citri	Diaphorina_citri	GCF_000475195.1_Diaci_psyllid_genome_assembly_v...	train_val
Dinoponera quadriceps	Dinoponera_quadriceps	GCF_001313825.1_ASM131382v1	test
Diprion similis	Diprion_similis	GCF_021155765.1_iyDipSimi1.1	test
Diuraphis noxia	Diuraphis_noxia	GCF_001186385.1_Dnoxia_1.0	train_val
Drosophila albomicans	Drosophila_albomicans	GCF_009650485.1_drosAlbom15112-1751.03v1	test (used)
Drosophila ananassae	Drosophila_ananassae	GCF_017639315.1_ASM1763931v2	train_val
Drosophila arizonae	Drosophila_arizonae	GCF_001654025.1_ASM165402v1	test
Drosophila biarmipes	Drosophila_biarmipes	GCF_018148935.1_ASM1814893v1	train_val
Drosophila bipectinata	Drosophila_bipectinata	GCF_018153845.1_ASM1815384v1	train_val
Drosophila busckii	Drosophila_busckii	GCF_011750605.1_ASM1175060v1	train_val
Drosophila elegans	Drosophila_elegans	GCF_018152505.1_ASM1815250v1	train_val
Drosophila erecta	Drosophila_erecta	GCF_003286155.1_DereRS2	train_val
Drosophila eugracilis	Drosophila_eugracilis	GCF_018153835.1_ASM1815383v1	train_val
Drosophila ficusphila	Drosophila_ficusphila	GCF_018152265.1_ASM1815226v1	test
Drosophila grimshawi	Drosophila_grimshawi	GCF_018153295.1_ASM1815329v1	train_val
Drosophila guanche	Drosophila_guanche	GCF_900245975.1_DGUA_6	test
Drosophila hydei	Drosophila_hydei	GCF_003285905.1_DhydRS2	train_val
Drosophila innubila	Drosophila_innubila	GCF_004354385.1_UK_Dinn_1.0	train_val
Drosophila kikkawai	Drosophila_kikkawai	GCF_018152535.1_ASM1815253v1	train_val
Drosophila mauritiana	Drosophila_mauritiana	GCF_004382145.1_ASM438214v1	train_val
Drosophila melanogaster	Drosophila_melanogaster	GCF_000001215.4_Release_6_plus_ISO1_MT	train_val
Drosophila miranda	Drosophila_miranda	GCF_003369915.1_D.miranda_PacBio2.1	train_val
Drosophila mojavensis	Drosophila_mojavensis	GCF_018153725.1_ASM1815372v1	train_val
Drosophila navojoa	Drosophila_navojoa	GCF_001654015.2_UFRJ_Dnav_4.2	train_val
Drosophila novamexicana	Drosophila_novamexicana	GCF_003285875.2_DnovRS2.1	train_val
Drosophila obscura	Drosophila_obscura	GCF_018151105.1_ASM1815110v1	test
Drosophila persimilis	Drosophila_persimilis	GCF_003286085.1_DperRS2	train_val
Drosophila pseudoobscura	Drosophila_pseudoobscura	GCF_009870125.1_UCI_Dpse_MV25	train_val
Drosophila rhopaloa	Drosophila_rhopaloa	GCF_018152115.1_ASM1815211v1	train_val
Drosophila santomea	Drosophila_santomea	GCF_016746245.2_Prin_Dsan_1.1	train_val
Drosophila sechellia	Drosophila_sechellia	GCF_004382195.1_ASM438219v1	test
Drosophila serrata	Drosophila_serrata	GCF_002093755.1_Dser1.0	train_val
Drosophila simulans	Drosophila_simulans	GCF_016746395.2_Prin_Dsim_3.1	train_val
Drosophila subobscura	Drosophila_subobscura	GCF_008121235.1_UCBerk_Dsub_1.0	train_val
Drosophila subpulchrella	Drosophila_subpulchrella	GCF_014743375.2_RU_Dsub_v1.1	test
Drosophila suzukii	Drosophila_suzukii	GCF_013340165.1_LBDM_Dsuz_2.1.pri	train_val
Drosophila takahashii	Drosophila_takahashii	GCF_018152695.1_ASM1815269v1	test
Drosophila teissieri	Drosophila_teissieri	GCF_016746235.2_Prin_Dtei_1.1	train_val
Drosophila virilis	Drosophila_virilis	GCF_003285735.1_DvirRS2	test (used)
Drosophila willistoni	Drosophila_willistoni	GCF_000005925.1_dwil_caf1	test
Drosophila yakuba	Drosophila_yakuba	GCF_016746365.2_Prin_Dyak_Tai18E2_2.1	test
Dufourea novaeangliae	Dufourea_novaeangliae	GCF_001272555.1_ASM127255v1	test
Echinococcus granulosus	Echinococcus_granulosus	GCF_000524195.1_ASM52419v1	test
Eufriesea mexicana	Eufriesea_mexicana	GCF_001483705.1_ASM148370v1	test
Eurytemora affinis	Eurytemora_affinis	GCF_000591075.1_Eaff_2.0	test
Exaiptasia diaphana	Exaiptasia_diaphana	GCF_001417965.1_Aiptasia_genome_1.1	train_val
Folsomia candida	Folsomia_candida	GCF_002217175.1_ASM221717v1	train_val
Fonticula alba	Fonticula_alba	GCF_000388065.1_Font_alba_ATCC_38817_V2	train_val
Fopius arisanus	Fopius_arisanus	GCF_000806365.1_ASM80636v1	train_val
Formica exsecta	Formica_exsecta	GCF_003651465.1_ASM365146v1	test
Frankliniella occidentalis	Frankliniella_occidentalis	GCF_000697945.2_Focc_2.1	test (used)
Frieseomelitta varia	Frieseomelitta_varia	GCF_011392965.1_Fvar_1.2	test
Galendromus occidentalis	Galendromus_occidentalis	GCF_000255335.1_Mocc_1.0	train_val
Galleria mellonella	Galleria_mellonella	GCF_003640425.2_ASM364042v2	train_val
Gigantopelta aegis	Gigantopelta_aegis	GCF_016097555.1_Gae_host_genome	train_val
Glossina fuscipes	Glossina_fuscipes	GCF_014805625.1_Yale_Gfus_2	train_val
Habropoda laboriosa	Habropoda_laboriosa	GCF_001263275.1_ASM126327v1	train_val
Haliotis rubra	Haliotis_rubra	GCF_003918875.1_ASM391887v1	train_val
Haliotis rufescens	Haliotis_rufescens	GCF_003343065.1_H.ruf_v1.0	test
Halyomorpha halys	Halyomorpha_halys	GCF_000696795.2_Hhal_2.0	train_val
Harmonia axyridis	Harmonia_axyridis	GCF_914767665.1_icHarAxyr1.1	test
Harpegnathos saltator	Harpegnathos_saltator	GCF_003227715.1_Hsal_v8.5	train_val
Helicoverpa armigera	Helicoverpa_armigera	GCF_002156985.1_Harm_1.0	test
Helobdella robusta	Helobdella_robusta	GCF_000326865.1_Helobdella_robusta_v1.0	test
Hermetia illucens	Hermetia_illucens	GCF_905115235.1_iHerIll2.2.curated.20191125	train_val
Homalodisca vitripennis	Homalodisca_vitripennis	GCF_021130785.1_UT_GWSS_2.1	train_val
Homarus americanus	Homarus_americanus	GCF_018991925.1_GMGI_Hamer_2.0	train_val

scientific	ID	version	split
Hyalella azteca	Hyalella_azteca	GCF_000764305.1_Hazt_2.0	test
Hydra vulgaris	Hydra_vulgaris	GCF_000004095.1_Hydra_RP_1.0	test
Hyposmocoma kahamanoa	Hyposmocoma_kahamanoa	GCF_003589595.1_ASM358959v1	test (used)
Ischnura elegans	Ischnura_elegans	GCF_921293095.1_ioIscEleg1.1	train_val
Ixodes scapularis	Ixodes_scapularis	GCF_016920785.2_ASM1692078v2	train_val
Lepeophtheirus salmonis	Lepeophtheirus_salmonis	GCF_016086655.3_UVic_Lsal_1.2	train_val
Leptinotarsa decemlineata	Leptinotarsa_decemlineata	GCF_000500325.1_Ldec_2.0	train_val
Leptopilina heterotoma	Leptopilina_heterotoma	GCF_015476425.1_ASM1547642v1	train_val
Limulus polyphemus	Limulus_polyphemus	GCF_000517525.1_Limulus_polyphemus-2.1.2	test
Linepithema humile	Linepithema_humile	GCF_000217595.1_Lhum_UMD_V04	train_val
Lingula anatina	Lingula_anatina	GCF_001039355.2_LinAna2.0	train_val
Loa loa	Loa_loa	GCF_000183805.2_Loa_loa_V3.1	train_val
Lottia gigantea	Lottia_gigantea	GCF_000327385.1_Helro1	train_val
Lucilia cuprina	Lucilia_cuprina	GCF_000699065.1_Lcup_2.0	test
Lucilia sericata	Lucilia_sericata	GCF_015586225.1_ASM1558622v1	train_val
Lytechinus variegatus	Lytechinus_variegatus	GCF_018143015.1_Lvar_3.0	test
Manduca sexta	Manduca_sexta	GCF_014839805.1_JHU_Msex_v1.0	train_val
Maniola hyperantus	Maniola_hyperantus	GCF_902806685.1_iAphHyp1.1	test
Maniola jurtina	Maniola_jurtina	GCF_905333055.1_ilManJurt1.1	train_val
Megachile rotundata	Megachile_rotundata	GCF_000220905.1_MROT_1.0	train_val
Megalopta genalis	Megalopta_genalis	GCF_011865705.1_USU_MGEN_1.2	train_val
Melanaphis sacchari	Melanaphis_sacchari	GCF_002803265.2_SCAv2.0	train_val
Melitaea cinxia	Melitaea_cinxia	GCF_905220565.1_ilMelCinx1.1	train_val
Mercenaria mercenaria	Mercenaria_mercenaria	GCF_014805675.1_ASM1480567v1.1	train_val
Microplitis demolitor	Microplitis_demolitor	GCF_000572035.2_Mdem2	train_val
Mizuhopecten yessoensis	Mizuhopecten_yessoensis	GCF_002113885.1_ASM211388v2	test
Monomorium pharaonis	Monomorium_pharaonis	GCF_013373865.1_ASM1337386v2	test
Monosiga brevicollis	Monosiga_brevicollis	GCF_000002865.3_V1.0	train_val
Musca domestica	Musca_domestica	GCF_000371365.1_Musca_domestica-2.0.2	train_val
Myzus persicae	Myzus_persicae	GCF_001856785.1_MPER_G0061.0	train_val
Nasonia vitripennis	Nasonia_vitripennis	GCF_009193385.2_Nvit_psr_1.1	train_val
Necator americanus	Necator_americanus	GCF_000507365.1_N_americanus_v1	train_val
Nematostella vectensis	Nematostella_vectensis	GCF_000209225.1_ASM20922v1	train_val
Neodiprion fabricii	Neodiprion_fabricii	GCF_021155785.1_iyNeoFabr1.1	train_val
Neodiprion lecontei	Neodiprion_lecontei	GCF_021901455.1_iyNeoLeco1.1	train_val
Neodiprion pinetum	Neodiprion_pinetum	GCF_021155775.1_iyNeoPine1.1	train_val
Neodiprion virginiana	Neodiprion_virginiana	GCF_021901495.1_iyNeoVirg1.1	train_val
Nicrophorus vespilloides	Nicrophorus_vespilloides	GCF_001412225.1_Nicve_v1.0	train_val
Nilaparvata lugens	Nilaparvata_lugens	GCF_014356525.1_ASM1435652v1	train_val
Nomia melanderi	Nomia_melanderi	GCF_003710045.1_USU_Nmel_1.2	train_val
Nylanderia fulva	Nylanderia_fulva	GCF_005281655.1_TAMU_Nfulva_1.0	train_val
Octopus bimaculoides	Octopus_bimaculoides	GCF_001194135.1_Octopus_bimaculoides_v2_0	train_val
Octopus sinensis	Octopus_sinensis	GCF_006345805.1_ASM634580v1	train_val
Octopus vulgaris	Octopus_vulgaris	GCF_006345805.1_ASM634580v1	train_val
Odontomachus brunneus	Odontomachus_brunneus	GCF_010583005.1_Obru_v1	train_val
Onthophagus taurus	Onthophagus_taurus	GCF_000648695.1_Otau_2.0	test
Ooceraea biroi	Ooceraea_biroi	GCF_003672135.1_Obir_v5.4	train_val
Opisthorchis viverrini	Opisthorchis_viverrini	GCF_000715545.1_OpiViv1.0	test (used)
Orbicella faveolata	Orbicella_faveolata	GCF_002042975.1_ofav_dov_v1	train_val
Orussus abietinus	Orussus_abietinus	GCF_000612105.2_Oabi_2.0	train_val
Osmia bicornis	Osmia_bicornis	GCF_907164935.1_iOsmBic2.1	train_val
Osmia lignaria	Osmia_lignaria	GCF_012274295.1_USDA_OLig_1.0	train_val
Ostrinia furnacalis	Ostrinia_furnacalis	GCF_004193835.1_ASM419383v1	train_val
Papilio machaon	Papilio_machaon	GCF_912999745.1_ilPapMach1.1	train_val
Papilio polytes	Papilio_polytes	GCF_000836215.1_Ppol_1.0	train_val
Papilio xuthus	Papilio_xuthus	GCF_000836235.1_Pxut_1.0	train_val
Pararge aegeria	Pararge_aegeria	GCF_905163445.1_ilParAegt1.1	train_val
Parasteatoda tepidariorum	Parasteatoda_tepidariorum	GCF_000365465.3_Ptep_3.0	train_val
Patiria miniata	Patiria_miniata	GCF_015706575.1_ASM1570657v1	train_val
Pecten maximus	Pecten_maximus	GCF_902652985.1_xPecMax1.1	train_val
Pediculus humanus	Pediculus_humanus	GCF_000006295.1_JCVI_LOUSE_1.0	test
Penaeus japonicus	Penaeus_japonicus	GCF_017312705.1_Mj_TUMSAT_v1.0	train_val
Penaeus monodon	Penaeus_monodon	GCF_015228065.1_NSTDA_Pmon_1	train_val
Penaeus vannamei	Penaeus_vannamei	GCF_003789085.1_ASM378908v1	test
Photinus pyralis	Photinus_pyralis	GCF_008802855.1_Ppyr1.3	train_val
Pieris brassicae	Pieris_brassicae	GCF_905147105.1_ilPieBrab1.1	train_val
Pieris rapae	Pieris_rapae	GCF_905147795.1_ilPieRapa1.1	train_val
Plutella xylostella	Plutella_xylostella	GCF_905116875.1_Haplomerged_assembly	train_val
Pocillopora damicornis	Pocillopora_damicornis	GCF_003704095.1_ASM370409v1	train_val
Pogonomyrmex barbatus	Pogonomyrmex_barbatus	GCF_000187915.1_Pbar_UMD_V03	train_val
Polistes canadensis	Polistes_canadensis	GCF_001313835.1_ASM131383v1	train_val
Polistes dominula	Polistes_dominula	GCF_001465965.1_Pdom_r1.2	test
Polistes fuscatus	Polistes_fuscatus	GCF_010416935.1_CU_Pfus_HIC	test
Pollicipes pollicipes	Pollicipes_pollicipes	GCF_011947565.2_Ppol_2	train_val
Pomacea canaliculata	Pomacea_canaliculata	GCF_003073045.1_ASM307304v1	train_val
Portunus trituberculatus	Portunus_trituberculatus	GCF_017591435.1_ASM1759143v1	train_val
Priapulus caudatus	Priapulus_caudatus	GCF_000485595.1_Priapulus_caudatus-5.0.1	test
Procambarus clarkii	Procambarus_clarkii	GCF_020424385.1_ASM2042438v2	test
Pseudomyrmex gracilis	Pseudomyrmex_gracilis	GCF_002006095.1_ASM200609v1	train_val
Rhagoletis pomonella	Rhagoletis_pomonella	GCF_013731165.1_Rhpom_1.0	test

scientific	ID	version	split
Rhagoletis zephyria	Rhagoletis_zephyria	GCF_001687245.1_Rhagoletis_zephyria_1.0	train_val
Rhipicephalus microplus	Rhipicephalus_microplus	GCF_013339725.1_ASM1333972v1	train_val
Rhipicephalus sanguineus	Rhipicephalus_sanguineus	GCF_013339695.1_ASM1333969v1	train_val
Rhopalosiphum maidis	Rhopalosiphum_maidis	GCF_003676215.2_ASM367621v3	test (used)
Saccoglossus kowalevskii	Saccoglossus_kowalevskii	GCF_000003605.2_Skow_1.1	train_val
Salpingoeca rosetta	Salpingoeca_rosetta	GCF_000188695.1_Proterospongia_sp_ATCC50818	train_val
Scaptodrosophila lebanonensis	Scaptodrosophila_lebanonensis	GCF_003285725.1_SlebRS2	train_val
Schistosoma haematobium	Schistosoma_haematobium	GCF_000699445.2_SchHae_2.0	train_val
Schistosoma mansoni	Schistosoma_mansoni	GCF_000237925.1_ASM23792v2	test (used)
Sipha flava	Sipha_flava	GCF_003268045.1_YSA_version1	test
Sitophilus oryzae	Sitophilus_oryzae	GCF_002938485.1_Soryzae_2.0	train_val
Solenopsis invicta	Solenopsis_invicta	GCF_016802725.1_UNIL_Sinv_3.0	train_val
Sphaeroforma arctica	Sphaeroforma_arctica	GCF_001186125.1_Spha_arctica_JP610_V1	train_val
Spodoptera frugiperda	Spodoptera_frugiperda	GCF_011064685.1_ZJU_Sfru_1.0	train_val
Spodoptera litura	Spodoptera_litura	GCF_002706865.1_ASM270686v1	train_val
Stegodyphus dumicola	Stegodyphus_dumicola	GCF_010614865.1_ASM1061486v1	train_val
Stomoxys calcitrans	Stomoxys_calcitrans	GCF_001015335.1_Stomoxys_calcitrans-1.0.1	train_val
Strongylocentrotus purpuratus	Strongylocentrotus_purpuratus	GCF_000002235.5_Spur_5.0	train_val
Strongyloides ratti	Strongyloides_ratti	GCF_001040885.1_S_ratti_ED321	test
Styela clava	Styela_clava	GCF_013122585.1_ASM1312258v2	test
Stylophora pistillata	Stylophora_pistillata	GCF_002571385.1_Stylophora_pistillata_v1	train_val
Teleopsis dalmanni	Teleopsis_dalmanni	GCF_002237135.1_ASM223713v2	test
Temnothorax curvispinosus	Temnothorax_curvispinosus	GCF_003070985.1_ASM307098v1	test
Tetranychus urticae	Tetranychus_urticae	GCF_000239435.1_ASM23943v1	train_val
Thrips palmi	Thrips_palmi	GCF_012932325.1_TpBJ-2018v1	train_val
Trachymyrmex cornetzi	Trachymyrmex_cornetzi	GCF_001594075.1_Tcor1.0	test (used)
Trachymyrmex septentrionalis	Trachymyrmex_septentrionalis	GCF_001594115.1_Tsep1.0	train_val
Trachymyrmex zeteki	Trachymyrmex_zeteki	GCF_001594055.1_Tzet1.0	test
Tribolium castaneum	Tribolium_castaneum	GCF_000002335.3_Tcas5.2	test (used)
Tribolium madens	Tribolium_madens	GCF_015345945.1_Tmad_KSU_1.1	train_val
Trichinella spiralis	Trichinella_spiralis	GCF_000181795.1_Trichinella_spiralis-3.7.1	train_val
Trichogramma pretiosum	Trichogramma_pretiosum	GCF_000599845.2_Tpre_2.0	test
Trichoplax adhaerens	Trichoplax_adhaerens	GCF_000150275.1_v1.0	train_val
Trichoplusia ni	Trichoplusia_ni	GCF_003590095.1_tn1	test
Vanessa tameamea	Vanessa_tameamea	GCF_002938995.1_ASM293899v1	test
Varroa destructor	Varroa_destructor	GCF_002443255.1_Vdes_3.0	train_val
Varroa jacobsoni	Varroa_jacobsoni	GCF_002532875.1_vjacob_1.0	train_val
Venturia canescens	Venturia_canescens	GCF_019457755.1_ASM1945775v1	train_val
Vespa crabro	Vespa_crabro	GCF_910589235.1_iyVesCrab1.2	train_val
Vespa mandarinia	Vespa_mandarinia	GCF_014083535.2_V.mandarinia_Nanaimo_p1.0	train_val
Vespula pensylvanica	Vespula_pensylvanica	GCF_014466175.1_ASM1446617v1	train_val
Vollenhovia emeryi	Vollenhovia_emeryi	GCF_000949405.1_V.emery_V1.0	train_val
Wasmannia auropunctata	Wasmannia_auropunctata	GCF_000956235.1_wasmannia.A_1.0	test
Xenia sp Carnegie-2017	Xenia_sp._Carnegie-2017	GCF_021976095.1_XeniaSp_v1	train_val
Zerene cesonia	Zerene_cesonia	GCF_012273895.1_Zerene_cesonia_1.1	train_val
Zeugodacus cucurbitae	Zeugodacus_cucurbitae	GCF_000806345.1_ASM80634v1	train_val
Zootermopsis nevadensis	Zootermopsis_nevadensis	GCF_000696155.1_ZooNev1.0	train_val

Table S21: Overview of assessed mammal genomes, including their scientific names, identification, and version. The "Split" column indicates whether each species was designated for model training/validation or model evaluation purposes.

scientific	ID	version	split
Aotus nancymaae	Aotus_nancymaae	GCF_000952055.2_Anan_2.0	train
Bos taurus	Bos_taurus	GCF_000003205.7_Btau_5.0.1	test
Camelus bactrianus	Camelus_bactrianus	GCF_000767855.1_Ca_bactrianus_MBC_1.0	test
Canis lupus familiaris	Canis_lupus_familiaris	GCF_000002285.3_CanFam3.1	train
Cavia porcellus	Cavia_porcellus	GCF_000151735.1_Cavpor3.0	train
Cebus imitator	Cebus_imitator	GCF_001604975.1_Cebus_imitator-1.0	train
Ceratotherium simum	Ceratotherium_simum	GCF_000283155.1_CerSimSim1.0	train
Chinchilla lanigera	Chinchilla_lanigera	GCF_000276665.1_ChiLan1.0	train
Condylura cristata	Condylura_cristata	GCF_000260355.1_ConCri1.0	train
Delphinapterus leucas	Delphinapterus_leucas	GCF_002288925.1_ASM294091v2	test
Desmodus rotundus	Desmodus_rotundus	GCF_022682495.1_HLdesRot8A	train
Dipodomys ordii	Dipodomys_ordii	GCF_000151885.1_Dord_2.0	train
Enhydra lutris	Enhydra_lutris	GCF_002288905.1_ASM228890v2	train
Eptesicus fuscus	Eptesicus_fuscus	GCF_000308155.1_EptFus1.0	train
Equus caballus	Equus_caballus	GCF_000002305.2_EquCab2.0	train
Heterocephalus glaber	Heterocephalus_glaber	GCF_000247695.1_HetGla_female_1.0	train
Homo sapiens	Homo_sapiens	GCF_000001405.40_GRCh38.p14	test
Ictidomys tridecemlineatus	Ictidomys_tridecemlineatus	GCF_000236235.1_SpeTri2.0	train
Jaculus jaculus	Jaculus_jaculus	GCF_000280705.1_JacJac1.0	train
Loxodonta africana	Loxodonta_africana	GCF_000001905.1_Loxafr3.0	train
Marmota marmota	Marmota_marmota	GCF_001458135.1_marMar2.1	train
Microcebus murinus	Microcebus_murinus	GCF_000165445.2_Mmur_3.0	train
Microtus ochrogaster	Microtus_ochrogaster	GCF_000317375.1_MicOch1.0	train
Mus musculus	Mus_musculus	GCF_000001635.26_GRCm38.p6	train
Mus pahari	Mus_pahari	GCF_900095145.1_PAHARI_EIJ_v1.1	train
Neomonachus schauinslandi	Neomonachus_schauinslandi	GCF_002201575.2_ASM220157v2	train
Ochotona princeps	Ochotona_princeps	GCF_000292845.1_OchPri3.0	train
Octodon degus	Octodon_degus	GCF_000260255.1_OctDeg1.0	train
Odobenus rosmarus	Odobenus_rosmarus	GCF_000321225.1_Oros_1.0	train
Otolemur garnettii	Otolemur_garnettii	GCF_000181295.1_OtoGar3	train
Panthera pardus	Panthera_pardus	GCF_001857705.1_PanPar1.0	val
Propithecus coquereli	Propithecus_coquereli	GCF_000956105.1_Pcoq_1.0	trai
Puma concolor	Puma_concolor	GCF_003327715.1_PumCon1.0	train
Rattus norvegicus	Rattus_norvegicus	GCF_000001895.5_Rnor_6.0	val
Saimiri boliviensis	Saimiri_boliviensis	GCF_000235385.1_SaiBol1.0	train
Sorex araneus	Sorex_araneus	GCF_000181275.1_SorAra2.0	train
Trichechus manatus	Trichechus_manatus	GCF_000243295.1_TriManLat1.0	train

S3.2.2 Training species sets

Table S22: Enumeration of the fungus species incorporated into distinct training sets.

training set name	seed_005_059	seed_006_059	seed_005_033	Seed_006_033
selection method	automated selection	automated selection	automated selection	automated selection
training species	Agaricus_bisporus	Aaosphaeria_arxii	Agaricus_bisporus	Aaosphaeria_arxii
	Alternaria_arborescens	Alternaria_arborescens	Alternaria_arborescens	Alternaria_arborescens
	Alternaria_burnsii	Alternaria_atra	Amorphotheca_resinae	Alternaria_atra
	Amorphotheca_resinae	Alternaria_burnsii	Apiotrichum_porosum	Alternaria_rosae
	Apiotrichum_porosum	Alternaria_rosae	Ascoidea_rubescens	Apiotrichum_porosum
	Aspergillus_alliaceus	Apiotrichum_porosum	Aspergillus_aculeatus	Ascoidea_rubescens
	Aspergillus_campestris	Aspergillus_aculeatinus	Aspergillus_alliaceus	Aspergillus_aculeatinus
	Aspergillus_chevalieri	Aspergillus_alliaceus	Aspergillus_brunneoviolaceus	Aspergillus_aculeatus
	Aspergillus_clavatus	Aspergillus_homomorphus	Aspergillus_campestris	Aspergillus_alliaceus
	Aspergillus_homomorphus	Aspergillus_melleus	Aspergillus_candidus	Aspergillus_brunneoviolaceus
	Aspergillus_japonicus	Aspergillus_nomiae	Aspergillus_chevalieri	Aspergillus_candidus
	Aspergillus_luchuensis	Aspergillus_tanneri	Aspergillus_clavatus	Aspergillus_flavus
	Aspergillus_nomiae	Aspergillus_tubingensis	Aspergillus_flavus	Aspergillus_homomorphus
	Aspergillus_tanneri	Aspergillus_udagawae	Aspergillus_homomorphus	Aspergillus_melleus
	Aspergillus_tubingensis	Aspergillus_uvarum	Aspergillus_japonicus	Aspergillus_nomiae
	Aspergillus_udagawae	Aspergillus_viridinutans	Aspergillus_luchuensis	Aspergillus_oryzae
	Aspergillus_uvarum	Aureobasidium_pullulans	Aspergillus_nomiae	Aspergillus_tanneri
	Aspergillus_viridinutans	Babjeviella_inositovora	Aspergillus_oryzae	Aspergillus_tubingensis
	Aspergillus_welwitschiae	Bacidia_gigantensis	Aspergillus_tanneri	Aspergillus_udagawae
	Aureobasidium_pullulans	Batrachochytrium_dendrobatidis	Aspergillus_tubingensis	Aspergillus_viridinutans
	Batrachochytrium_dendrobatidis	Bipolaris_victoriae	Aspergillus_udagawae	Aureobasidium_namibiae
	Bipolaris_sorokiniana	Blastomyces_dermatitidis	Aspergillus_viridinutans	Aureobasidium_pullulans
	Bipolaris_victoriae	Blastomyces_gilchristii	Aspergillus_welwitschiae	Babjeviella_inositovora
	Blastomyces_dermatitidis	Boeremia_exigua	Aureobasidium_namibiae	Bacidia_gigantensis
	Boeremia_exigua	Botrytis_cinerea	Aureobasidium_pullulans	Batrachochytrium_dendrobatidis
	Botrytis_cinerea	Botrytis_porri	Batrachochytrium_dendrobatidis	Blastomyces_dermatitidis
	Botrytis_fragariae	_Candida_auris	Bipolaris_sorokiniana	Blastomyces_gilchristii
	Botrytis_porri	Candida_dubliniensis	Blastomyces_dermatitidis	Boeremia_exigua
	_Candida_auris	Candida_parapsilosis	Boeremia_exigua	Botrytis_cinerea
	_Candida_haemuloni	Candida_tropicalis	Botrytis_cinerea	Brettanomyces_nanus
	Candida_parapsilosis	Colletotrichum_higginsianum	Botrytis_fragariae	Candida_albicans
	Candida_tropicalis	Cordyceps_militaris	Brettanomyces_nanus	Candida_dubliniensis
	Capronia_epimyces	Cryptococcus_amylolentus	Candida_albicans	Capronia_coronata
	Ceraceosorus_guamensis	Cryptococcus_neoformans	_Candida_haemuloni	Colletotrichum_fructicola
	Chaetomium_globosum	Cutaneotrichosporon_oleaginosum	Capronia_coronata	Coniophora_puteana
	Coccidioides_posadasii	Cyberlindnera_jadinii	Capronia_epimyces	Cordyceps_militaris
	Colletotrichum_higginsianum	Debaryomyces_fabryi	Ceraceosorus_guamensis	Cryptococcus_amylolentus
	Coniosporium_apollinis	Dichomitus_squalens	Chaetomium_globosum	Cryptococcus_neoformans
	Cordyceps_militaris	Diutina_rugosa	Coccidioides_posadasii	Cutaneotrichosporon_oleaginosum
	Cucurbitaria_berberidis	Dothidotthia_symphoricarpi	Colletotrichum_fructicola	Cyberlindnera_jadinii
	Cutaneotrichosporon_oleaginosum	Drechmeria_coniospora	Coniophora_puteana	Dacryopinax_primogenitus
	Cyberlindnera_jadinii	Encephalitozoon_cuniculi	Coniosporium_apollinis	Debaryomyces_fabryi
	Debaryomyces_fabryi	Encephalitozoon_romaleae	Cordyceps_militaris	Dichomitus_squalens
	Diaporthe_citri	Endocarpon_pusillum	Cucurbitaria_berberidis	Dothidotthia_symphoricarpi
	Dichomitus_squalens	Exophiala_aquamarina	Cutaneotrichosporon_oleaginosum	Encephalitozoon_romaleae
	Diutina_rugosa	Exophiala_oligosperma	Cyberlindnera_jadinii	Endocarpon_pusillum
	Drechmeria_coniospora	Exserohilum_turcicum	Dacryopinax_primogenitus	Exophiala_oligosperma
	Emericellopsis_atlantica	Fibroporia_radiculosa	Debaryomyces_fabryi	Exophiala_xenobiotica
	Encephalitozoon_cuniculi	Filobasidium_floriforme	Diaporthe_citri	Exserohilum_turcicum
	Endocarpon_pusillum	Fomitiporia_mediterranea	Dichomitus_squalens	Fibroporia_radiculosa
	Eremomyces_bilateralis	Fonsecaea_erecta	Emericellopsis_atlantica	Fonsecaea_erecta
	Exophiala_aquamarina	Fonsecaea_pedrosoi	Endocarpon_pusillum	Fonsecaea_nubica
	Exophiala_oligosperma	Fusarium_flagelliforme	Eremomyces_bilateralis	Fonsecaea_pedrosoi
	Exserohilum_turcicum	Fusarium_fujikuroi	Exophiala_oligosperma	Fusarium_odoratissimum
	Filobasidium_floriforme	Fusarium_mangiferae	Exophiala_xenobiotica	Fusarium_oxysporum
	Fomitiporia_mediterranea	Fusarium_odoratissimum	Exserohilum_turcicum	Fusarium_proliferatum
	Fonsecaea_pedrosoi	Fusarium_oxysporum	Fonsecaea_nubica	Fusarium_pseudograminearum
	Fusarium_flagelliforme	Fusarium_proliferatum	Fonsecaea_pedrosoi	Geosmithia_morbida
	Fusarium_fujikuroi	Fusarium_pseudograminearum	Fusarium_odoratissimum	Heterobasidion_irregulare
	Fusarium_mangiferae	Geosmithia_morbida	Fusarium_proliferatum	Histoplasma_capsulatum
	Fusarium_odoratissimum	Grosmannia_clavigera	Fusarium_pseudograminearum	Hyphopichia_burtonii
	Fusarium_proliferatum	Heterobasidion_irregulare	Geosmithia_morbida	Komagataella_phaffii
	Fusarium_pseudograminearum	Histoplasma_capsulatum	Heterobasidion_irregulare	Kwoniella_bestiolae
	Geosmithia_morbida	Hyaloscypha_bicolor	Kluyveromyces_lactis	Kwoniella_mangrovensis
	Grosmannia_clavigera	Hyphopichia_burtonii	Kwoniella_bestiolae	Kwoniella_shandongensis
	Heterobasidion_irregulare	Komagataella_phaffii	Kwoniella_mangrovensis	Lachancea_thermotolerans
	Hyaloscypha_bicolor	Kwoniella_shandongensis	Kwoniella_pini	Lasiodiplodia_theobromae
	Kluyveromyces_lactis	Laetiporus_sulphureus	Lachancea_thermotolerans	Leptosphaeria_maculans

training set name	seed_005_059	seed_006_059	seed_005_033	Seed_006_033
training species (cont.)	Kwoniella_pini	Leptosphaeria_maculans	Lasiodiplodia_theobromae	Lindgomyces_ingoldianus
	Laetiporus_sulphureus	Letharia_columbiana	Lentinula_edodes	Malassezia_pachydermatis
	Lentinula_edodes	Malassezia_sympodialis	Lindgomyces_ingoldianus	Meira_miltonrushii
	Letharia_columbiana	Melampsora_larici-populina	Malassezia_pachydermatis	Metarhizium_album
	Malassezia_sympodialis	Metschnikowia_bicuspidata	Meira_miltonrushii	Metschnikowia_bicuspidata
	Melampsora_larici-populina	Microsporum_canis	Metarhizium_album	Microsporum_canis
	Metarhizium_brunneum	Mixia_osmundae	Metarhizium_brunneum	Mollisia_scopiformis
	Microsporum_canis	Mollisia_scopiformis	Microsporum_canis	Morchella_importuna
	Mixia_osmundae	Morchella_importuna	Morchella_importuna	Morchella_sextelata
	Morchella_importuna	Morchella_sextelata	Morchella_sextelata	Mytilinidion_resinicola
	Morchella_sextelata	Mytilinidion_resinicola	Mytilinidion_resinicola	Nematocida_parisii
	Mytilinidion_resinicola	Neurospora_tetrasperma	Nematocida_parisii	Neurospora_tetrasperma
	Neurospora_tetrasperma	Penicillium_digitatum	Neurospora_tetrasperma	Ogataea_haglerorum
	Nosema_ceranae	Pestalotiopsis_fici	Nosema_ceranae	Parastagonospora_nodorum
	Ogataea_polymorpha	Pichia_kudriavzevii	Ogataea_haglerorum	Penicillium_digitatum
	Pestalotiopsis_fici	Pleurotus_ostreatus	Ogataea_polymorpha	Penicillium_rubens
	Pneumocystis_murina	Pneumocystis_jirovecii	Parastagonospora_nodorum	Pestalotiopsis_fici
	Pochonia_chlamydosporia	Pneumocystis_murina	Penicillium_rubens	Pichia_kudriavzevii
	Podospora_anserina	Pseudogymnoascus_verrucosus	Pestalotiopsis_fici	Pleurotus_ostreatus
	Postia_placenta	Pseudomassariella_vexata	Pochonia_chlamydosporia	Pneumocystis_jirovecii
	Pseudogymnoascus_destructans	Pseudomicrostroma_glucosiphilum	Podospora_anserina	Protomyces_lactucae-debilis
	Pseudogymnoascus_verrucosus	Pseudozyma_hubeiensis	Postia_placenta	Pseudogymnoascus_verrucosus
	Pseudomassariella_vexata	Punctularia_strigosozonata	Protomyces_lactucae-debilis	Pseudomassariella_vexata
	Pseudozyma_hubeiensis	Pyrenophora_tritici-repentis	Pseudogymnoascus_destructans	Pseudomicrostroma_glucosiphilum
	Punctularia_strigosozonata	Rhizophagus_irregularis	Pseudogymnoascus_verrucosus	Pseudozyma_flocculosa
	Pyrenophora_tritici-repentis	Saccharomyces_cerevisiae	Pseudomassariella_vexata	Pseudozyma_hubeiensis
	Rhinocladiella_mackenziei	Saccharomyces_paradoxus	Pseudozyma_flocculosa	Punctularia_strigosozonata
	Rhizophagus_irregularis	Saprochaete_ingens	Pseudozyma_hubeiensis	Pyricularia_oryzae
	Saccharomyces_cerevisiae	Scheffersomyces_stipitis	Punctularia_strigosozonata	Pyricularia_pennisetigena
	Saccharomyces_paradoxus	Schizosaccharomyces_cryophilus	Pyricularia_oryzae	Rhodotorula_toruloides
	Saprochaete_ingens	Schizosaccharomyces_japonicus	Pyricularia_pennisetigena	Saccharomyces_cerevisiae
	Scheffersomyces_stipitis	Schizosaccharomyces_pombe	Rhinocladiella_mackenziei	Saccharomycodes_ludwigii
	Schizosaccharomyces_cryophilus	Sclerotinia_sclerotiorum	Rhodotorula_toruloides	Saitoella_complicata
	Schizosaccharomyces_japonicus	Sordaria_macrospora	Saccharomyces_cerevisiae	Sordaria_macrospora
	Schizosaccharomyces_pombe	Sphaerulina_musiva	Saccharomycodes_ludwigii	Spizellomyces_punctatus
	Sclerotinia_sclerotiorum	Spizellomyces_punctatus	Saitoella_complicata	Sporisorium_graminicola
	Sordaria_macrospora	Sporisorium_graminicola	Sordaria_macrospora	Sporothrix_brasiliensis
	Sparassis_crispa	Sugiyamaella_lignohabitans	Sparassis_crispa	Stereum_hirsutum
	Spathaspora_passalidarum	Suillus_discolor	Spathaspora_passalidarum	Sugiyamaella_lignohabitans
	Sphaerulina_musiva	Suillus_plorans	Spizellomyces_punctatus	Suillus_discolor
	Spizellomyces_punctatus	Suillus_subaureus	Sporothrix_brasiliensis	Suillus_plorans
	Sugiyamaella_lignohabitans	Synchytrium_microbalum	Stereum_hirsutum	Suillus_subaureus
	Suillus_clintonianus	Talaromyces_amestolkiae	Sugiyamaella_lignohabitans	Synchytrium_microbalum
	Suillus_paluster	Talaromyces_rugulosus	Suillus_clintonianus	Talaromyces_amestolkiae
	Suillus_plorans	Tetrapisispora_blattae	Suillus_paluster	Talaromyces_rugulosus
	Talaromyces_rugulosus	Thermothelomyces_thermophilus	Suillus_plorans	Tetrapisispora_blattae
	Thermothelomyces_thermophilus	Thermothielavioides_terrestris	Talaromyces_rugulosus	Thermothelomyces_thermophilus
	Thermothielavioides_terrestris	Tilletiopsis_washingtonensis	Thermothelomyces_thermophilus	Thermothielavioides_terrestris
	Tilletiaria_anomala	Tremella_mesenterica	Thermothielavioides_terrestris	Tilletiopsis_washingtonensis
	Tremella_mesenterica	Trichoderma_gamsii	Tilletiaria_anomala	Trametes_versicolor
	Trichoderma_reesei	Tuber_melanosporum	Trametes_versicolor	Trichoderma_citrinoviride
	Tuber_melanosporum	Uncinocarpus_reesii	Trichoderma_citrinoviride	Trichoderma_gamsii
	Uncinocarpus_reesii	Ustilago_hordei	Trichoderma_reesei	Venustampulla_echinocandica
	Ustilago_hordei	Venustampulla_echinocandica	Vavraia_culicis	Verticillium_alfalfae
	Vavraia_culicis	Wallemia_ichthyophaga	Venustampulla_echinocandica	Wallemia_ichthyophaga
	Venustampulla_echinocandica	Wickerhamiella_sorbophila	Verticillium_alfalfae	Wickerhamiella_sorbophila
	Wickerhamomyces_anomalus	Wickerhamomyces_anomalus	Wickerhamomyces_anomalus	Wickerhamomyces_anomalus
	Xylona_heveae	Xylona_heveae	Yarrowia_lipolytica	Yarrowia_lipolytica
	Yarrowia_lipolytica	Yarrowia_lipolytica	Zygosaccharomyces_rouxii	Zygosaccharomyces_rouxii
	Zygosaccharomyces_rouxii	Zygosaccharomyces_rouxii	Zymoseptoria_tritici	Zymoseptoria_tritici
validation species	Each fungus species marked as train_val in the table S17 and not listed under training species was used for validation. Only a random selection of 800 subsequences per species were used.			

Table S23: Enumeration of the plant species incorporated into distinct training sets.

training set name	Fullmoon_211117_17	ori9	seed_002_016	seed_009_017	seed_002_017	seed_009_016
selection method	manual selection	manual selection	automated selection	automated selection	automated selection	automated selection
training species	Creinhardtii<br>Dalata<br>Esalsugineum<br>Graimondii<br>Mguttatus<br>Mpolymorpha<br>MspRCC299<br>Ncolorata<br>Phallii<br>Ppatens<br>Ppersica<br>Ptrifoliata<br>Sitalica<br>Athaliana<br>Bdistachyon<br>Ptrichocarpa<br>Vunguiculata	Creinhardtii<br>Mguttatus<br>Mpolymorpha<br>Sitalica<br>Athaliana<br>Bdistachyon<br>Ptrichocarpa<br>Zmays<br>Gmax	Ahypochondriacus<br>Alyrata<br>Boleraceacapitata<br>Carietinum<br>Cclementina<br>Crubella<br>Csativus<br>Csinensis<br>CsubellipsoideaC169<br>Czofingiensis<br>Dalata<br>Dsalina<br>Esalsugineum<br>Graimondii<br>Lusitatissimum<br>Mesculenta<br>Mguttatus<br>MpusillaCCMP1545<br>Ncolorata<br>Osativa<br>Pacutifolius<br>Phallii<br>Ppatens<br>Ptrichocarpa<br>Ptrifoliata<br>Pvirgatum<br>Slycopersicum<br>Stuberosum<br>Vcarteri<br>Vunguiculata<br>Zmarina<br>Zmays	Acomosus<br>Athaliana<br>Bdistachyon<br>Carietinum<br>Cgrandiflora<br>Creinhardtii<br>Crubella<br>Esalsugineum<br>Fvesca<br>Gmax<br>Ljaponicus<br>Lusitatissimum<br>Macuminata<br>Mesculenta<br>Mguttatus<br>Mpolymorpha<br>MpusillaCCMP1545<br>Mtruncatula<br>Ncolorata<br>Olucimarinus<br>Osativa<br>Othomaeum<br>Pacutifolius<br>Ppersica<br>Pumbilicalis<br>Sbicolor<br>Slycopersicum<br>Sparvula<br>Taestivum<br>Tpratense<br>Vcarteri<br>Zmays	Acomosus<br>Athaliana<br>Boleraceacapitata<br>Carietinum<br>Cgrandiflora<br>Creinhardtii<br>Crubella<br>Csativus<br>Dalata<br>Esalsugineum<br>Fvesca<br>Graimondii<br>Ljaponicus<br>Lusitatissimum<br>Macuminata<br>Mesculenta<br>Mguttatus<br>Mpolymorpha<br>MpusillaCCMP1545<br>Ncolorata<br>Osativa<br>Othomaeum<br>Pacutifolius<br>Ppatens<br>Ptrichocarpa<br>Pvirgatum<br>Slycopersicum<br>Stuberosum<br>Tpratense<br>Vcarteri<br>Zmarina<br>Zmays	Ahypochondriacus<br>Alyrata<br>Bdistachyon<br>Carietinum<br>Cclementina<br>Crubella<br>Csinensis<br>CsubellipsoideaC169<br>Czofingiensis<br>Dsalina<br>Esalsugineum<br>Gmax<br>Lusitatissimum<br>Mesculenta<br>Mguttatus<br>MpusillaCCMP1545<br>Mtruncatula<br>Ncolorata<br>Olucimarinus<br>Osativa<br>Pacutifolius<br>Phallii<br>Ppersica<br>Ptrifoliata<br>Pumbilicalis<br>Sbicolor<br>Slycopersicum<br>Sparvula<br>Taestivum<br>Vcarteri<br>Vunguiculata<br>Zmays
skipped validators	Crubella<br>Dsalina<br>Gmax<br>Hannuus<br>Mdomestica<br>Sbicolor<br>Zmays	Crubella<br>Dalata<br>Dsalina<br>Esalsugineum<br>Graimondii<br>Hannuus<br>Mdomestica<br>MspRCC299<br>Ncolorata<br>Phallii<br>Ppatens<br>Ppersica<br>Ptrifoliata<br>Sbicolor<br>Vunguiculata				
validation species	Each plant species marked as train_val in the table S18 and not listed under training species was used for validation. Only a random selection of 800 subsequences per species were used.					

Table S24: Enumeration of the vertebrate species / mixed vertebrate and invertebrate species incorporated into distinct training sets.

training set name	seed_020_036	seed_020_043_ori0_up0_xtr1	ori6 (mixed)	seed_004_043	seed_020_043
selection method	automated selection	automated selection + tuning	manual selection	automated selection	automated selection
training species	Antrostomus_carolinensis	Alligator_mississippiensis	Anabas_testudineus	Alligator_mississippiensis	Alligator_mississippiensis
	Athene_cunicularia	Antrostomus_carolinensis	Drosophila_melanogaster	Calidris_pugnax	Antrostomus_carolinensis
	Calidris_pugnax	Calidris_pugnax	Gallus_gallus	Camelus_dromedarius	Calidris_pugnax
	Canis_lupus_dingo	Camelus_dromedarius	Mus_musculus	Canis_lupus_dingo	Camelus_dromedarius
	Castor_canadensis	Castor_canadensis	Oryzias_latipes	Castor_canadensis	Canis_lupus_dingo
	Chelonoidis_abingdonii	Cavia_porcellus	Theropithecus_gelada	Cervus_elaphus	Castor_canadensis
	Chinchilla_lanigera	Chelonoidis_abingdonii		Chlamydotis_macqueenii	Chelonoidis_abingdonii
	Chrysochloris_asiatica	Gouania_willdenowi		Clupea_harengus	Chinchilla_lanigera
	Cottoperca_gobio	Lacerta_agilis		Dasypus_novemcinctus	Gouania_willdenowi
	Gouania_willdenowi	Leptosomus_discolor		Etheostoma_spectabile	Lacerta_agilis
	Lates_calcarifer	Macaca_fascicularis		Lacerta_agilis	Leptonychotes_weddellii
	Leptonychotes_weddellii	Mustela_putorius		Latimeria_chalumnae	Leptosomus_discolor
	Megalops_cyprinoides	Myripristis_murdjan		Leptosomus_discolor	Macaca_fascicularis
	Meles_meles	Neogale_vison		Macaca_fascicularis	Mustela_putorius
	Mustela_putorius	Neomonachus_schauinslandi		Monopterus_albus	Myripristis_murdjan
	Neogale_vison	Nothoprocta_perdicaria		Myripristis_murdjan	Neogale_vison
	Nothoprocta_perdicaria	Odobenus_rosmarus		Neogale_vison	Odobenus_rosmarus
	Ochotona_princeps	Oxyura_jamaicensis		Oncorhynchus_keta	Oxyura_jamaicensis
	Odobenus_rosmarus	Panthera_leo		Oxyura_jamaicensis	Panthera_leo
	Panthera_leo	Panthera_tigris		Panthera_tigris	Panthera_tigris
	Pantherophis_guttatus	Pipra_filicauda		Paramormyrops_kingsleyae	Pantherophis_guttatus
	Pan_troglodytes	Protobothrops_mucrosquamatus		Petromyzon_marinus	Paramormyrops_kingsleyae
	Parambassis_ranga	Pygocentrus_nattereri		Pygocentrus_nattereri	Pipra_filicauda
	Piliocolobus_tephrosceles	Sander_lucioperca		Serinus_canaria	Pygocentrus_nattereri
	Pipra_filicauda	Scleropages_formosus		Sorex_araneus	Sander_lucioperca
	Pygoscelis_adeliae	Serinus_canaria		Struthio_camelus	Serinus_canaria
	Rattus_norvegicus	Struthio_camelus		Tachyglossus_aculeatus	Sorex_araneus
	Sander_lucioperca	Tachyglossus_aculeatus		Talpa_occidentalis	Struthio_camelus
	Tachyglossus_aculeatus	Tachysurus_fulvidraco		Theropithecus_gelada	Tachyglossus_aculeatus
	Tachysurus_fulvidraco	Takifugu_rubripes		Tinamus_guttatus	Tachysurus_fulvidraco
	Takifugu_rubripes	Talpa_occidentalis		Trematomus_bernacchii	Takifugu_rubripes
	Thunnus_albacares			Varanus_komodoensis	Tinamus_guttatus
validation species	For model 'ori6' each vertebrate and invertebrate species marked as train_val in the tables S19 and S20 and not listed under training species was used for validation. Only a random selection of 800 subsequences per species were used.<br>For all other models each vertebrate species marked as train_val in the table S19 and not listed under training species were used for validation. Only a random selection of 800 subsequences per species were used.				

Table S25: Enumeration of the invertebrate species incorporated into distinct training sets.

training set name	seed_015_045	seed_001_045	seed_022_048_booster<br>_001_039_ori1_up1_xtr0	seed_001_039	seed_015_039
selection method	automated selection	automated selection	automated selection + tuning	automated selection	automated selection
training species	Actinia_tenebrosa<br>Amphimedon_queenslandica<br>Anopheles_coluzzii<br>Anoplophora_glabripennis<br>Caenorhabditis_elegans<br>Drosophila_grimshawi<br>Drosophila_miranda<br>Drosophila_teissieri<br>Haliotis_rubra<br>Homalodisca_vitripennis<br>Ischnura_elegans<br>Loa_loa<br>Nasonia_vitripennis<br>Necator_americanus<br>Neodiprion_fabricii<br>Neodiprion_virginiana<br>Nicrophorus_vespilloides<br>Nomia_melanderi<br>Orussus_abietinus<br>Pogonomyrmex_barbatus<br>Pomacea_canaliculata<br>Portunus_trituberculatus<br>Rhagoletis_zephyria<br>Salpingoeca_rosetta<br>Schistosoma_haematobium<br>Sphaeroforma_arctica<br>Spodoptera_frugiperda<br>Strongylocentrotus_purpuratus<br>Tetranychus_urticae<br>Varroa_jacobsoni<br>Xenia_sp._Carnegie-2017<br>Zerene_cesonia	Acyrthosiphon_pisum<br>Anopheles_albimanus<br>Anopheles_coluzzii<br>Anoplophora_glabripennis<br>Aphis_gossypii<br>Bemisia_tabaci<br>Bicyclus_anynana<br>Chelonus_insularis<br>Drosophila_elegans<br>Drosophila_grimshawi<br>Drosophila_rhopaloa<br>Drosophila_simulans<br>Drosophila_teissieri<br>Fonticula_alba<br>Haliotis_rubra<br>Homalodisca_vitripennis<br>Ischnura_elegans<br>Loa_loa<br>Lottia_gigantea<br>Lucilia_sericata<br>Nasonia_vitripennis<br>Neodiprion_virginiana<br>Nicrophorus_vespilloides<br>Pogonomyrmex_barbatus<br>Pseudomyrmex_gracilis<br>Rhagoletis_zephyria<br>Salpingoeca_rosetta<br>Schistosoma_haematobium<br>Spodoptera_frugiperda<br>Strongylocentrotus_purpuratus<br>Zerene_cesonia<br>Zootermopsis_nevadensis	Acromyrmex_echinatior<br>Actinia_tenebrosa<br>Acyrthosiphon_pisum<br>Anopheles_albimanus<br>Anopheles_coluzzii<br>Anopheles_stephensi<br>Aphis_gossypii<br>Apis_mellifera<br>Bemisia_tabaci<br>Bicyclus_anynana<br>Bombyx_mori<br>Bradysia_coprophila<br>Brugia_malayi<br>Caenorhabditis_elegans<br>Ceratitis_capitata<br>Chelonus_insularis<br>Ciona_intestinalis<br>Cryptotermes_secundus<br>Daphnia_pulicaria<br>Dendroctonus_ponderosae<br>Dermacentor_silvarum<br>Diachasma_alloeum<br>Diuraphis_noxia<br>Drosophila_biarmipes<br>Drosophila_busckii<br>Drosophila_elegans<br>Drosophila_kikkawai<br>Drosophila_melanogaster<br>Drosophila_navojoa<br>Drosophila_rhopaloa<br>Drosophila_santomea<br>Drosophila_serrata<br>Drosophila_simulans<br>Drosophila_teissieri<br>Exaiptasia_diaphana<br>Fonticula_alba<br>Gigantopelta_aegis<br>Haliotis_rubra<br>Hermetia_illucens<br>Ixodes_scapularis<br>Leptopilina_heterotoma<br>Lottia_gigantea<br>Lucilia_sericata<br>Megalopta_genalis<br>Melanaphis_sacchari<br>Neodiprion_pinetum<br>Nylanderia_fulva<br>Octopus_bimaculoides<br>Odontomachus_brunneus<br>Ooceraea_biroi<br>Papilio_machaon<br>Parasteatoda_tepidariorum<br>Penaeus_japonicus<br>Pieris_rapae<br>Plutella_xylostella<br>Pogonomyrmex_barbatus<br>Pomacea_canaliculata<br>Pseudomyrmex_gracilis<br>Salpingoeca_rosetta<br>Thrips_palmi<br>Trichoplax_adhaerens<br>Varroa_destructor<br>Vespa_crabro<br>Vespula_pensylvanica<br>Zootermopsis_nevadensis	Acromyrmex_echinatior<br>Acyrthosiphon_pisum<br>Anopheles_albimanus<br>Aphis_gossypii<br>Bemisia_tabaci<br>Bicyclus_anynana<br>Bombyx_mori<br>Chelonus_insularis<br>Cryptotermes_secundus<br>Diachasma_alloeum<br>Drosophila_biarmipes<br>Drosophila_elegans<br>Drosophila_kikkawai<br>Drosophila_rhopaloa<br>Drosophila_santomea<br>Drosophila_simulans<br>Exaiptasia_diaphana<br>Fonticula_alba<br>Gigantopelta_aegis<br>Haliotis_rubra<br>Lottia_gigantea<br>Lucilia_sericata<br>Megalopta_genalis<br>Neodiprion_pinetum<br>Odontomachus_brunneus<br>Penaeus_japonicus<br>Plutella_xylostella<br>Pogonomyrmex_barbatus<br>Pseudomyrmex_gracilis<br>Salpingoeca_rosetta<br>Trichoplax_adhaerens<br>Zootermopsis_nevadensis	Acromyrmex_echinatior<br>Actinia_tenebrosa<br>Amphimedon_queenslandica<br>Bombyx_mori<br>Caenorhabditis_elegans<br>Cryptotermes_secundus<br>Diachasma_alloeum<br>Drosophila_biarmipes<br>Drosophila_kikkawai<br>Drosophila_miranda<br>Drosophila_santomea<br>Exaiptasia_diaphana<br>Gigantopelta_aegis<br>Haliotis_rubra<br>Megalopta_genalis<br>Necator_americanus<br>Neodiprion_fabricii<br>Neodiprion_pinetum<br>Nomia_melanderi<br>Odontomachus_brunneus<br>Orussus_abietinus<br>Penaeus_japonicus<br>Plutella_xylostella<br>Pogonomyrmex_barbatus<br>Pomacea_canaliculata<br>Portunus_trituberculatus<br>Salpingoeca_rosetta<br>Sphaeroforma_arctica<br>Tetranychus_urticae<br>Trichoplax_adhaerens<br>Varroa_jacobsoni<br>Xenia_sp._Carnegie-2017
validation species	Each invertebrate species marked as train_val in the table S20 and not listed under training species was used for validation. Only a random selection of 800 subsequences per species were used.				

S3.2.3 Encoding

Table S26: Modified one-hot base encoding as used by Helixer. Ambiguous bases are assumed to be equally probable.

Base	Encoding
C	[1., 0., 0., 0.]
A	[0., 1., 0., 0.]
T	[0., 0., 1., 0.]
G	[0., 0., 0., 1.]
Y	[0.5, 0., 0.5, 0.]
R	[0., 0.5, 0., 0.5]
W	[0., 0.5, 0.5, 0.]
S	[0.5, 0., 0., 0.5]
K	[0., 0., 0.5, 0.5]
M	[0.5, 0.5, 0., 0.]
D	[0., 0.33, 0.33, 0.33]
V	[0.33, 0.33, 0., 0.33]
H	[0.33, 0.33, 0.33, 0.]
B	[0.33, 0., 0.33, 0.33]
N	[0.25, 0.25, 0.25, 0.25]

Table S27: Additional length parameters used for Helixer inference on test species

	subsequence length	overlap offset	overlap core length
fungi	21384	10692	16038
land_plant	106920	53460	80190
invertebrate	213840	106920	160380
vertebrate	213840	106920	160380

S3.3 Training Parameters

S3.3.1 Model parameters

Table S28: Details of parameters utilised in the development of various Helixer fungus models that have been released. For performance metrics of each model, please refer to Figures S4 and S8.

parameter	fungi_v0.3_a_0100	fungi_v0.3_a_0200	fungi_v0.3_a_0300	fungi_v0.3_a_0400
data_dir	seed_005_059	seed_006_059	seed_005_033	seed_006_033
data_helixer_commit	ac7ba57	ac7ba57	ac7ba57	ac7ba57
data_geenuff_commit	46418ef	46418ef	46418ef	46418ef
training_helixer_commit	ac7ba57	ac7ba57	ac7ba57	ac7ba57
pool_size	9	9	9	9
batch_size	140	140	140	140
val_test_batch_size	280	280	280	280
class_weights	[0.7,1.6,1.2,1.2]	[0.7,1.6,1.2,1.2]	[0.7,1.6,1.2,1.2]	[0.7,1.6,1.2,1.2]
transition_weights	[1,12,3,1,12,3]	[1,12,3,1,12,3]	[1,12,3,1,12,3]	[1,12,3,1,12,3]
save_every_check	True	True	True	True
predict_phase	True	True	True	True
lstm_layers	3	3	3	3
cnn_layers	4	4	4	4
units	128	128	128	128
filter_depth	96	96	96	96
kernel_size	9	9	9	9
check_every_nth_batch	1000	1000	1000	1000
patience	6	6	6	6

Table S29: Details of parameters utilised in the development of various Helixer plant models that have been released. For performance metrics of each model, please refer to Figures S3 and S7.

parameter	land_plant_v0.3_a_0080	land_plant_v0.3_a_0090	land_plant_v0.3_a_0100	land_plant_v0.3_a_0200
data_dir	seed_009_017	seed_002_017	seed_009_017	seed_009_016
data_helixer_commit	ac7ba57	ac7ba57	02e5e94	02e5e94
data_geenuff_commit	46418ef	46418ef	46418ef	46418ef
training_helixer_commit	ac7ba57	ac7ba57	02e5e94	02e5e94
kernel_size	12	10	10	10
learning_rate	0.0003	0.0003	N/A	N/A
pool_size	9	9	9	9
batch_size	140	140	50	50
val_test_batch_size	280	280	100	100
class_weights	[0.7,1.6,1.2,1.2]	[0.7,1.6,1.2,1.2]	[0.7,1.6,1.2,1.2]	[0.7,1.6,1.2,1.2]
transition_weights	[1,12,3,1,12,3]	[1,12,3,1,12,3]	[1,12,3,1,12,3]	[1,12,3,1,12,3]
save_every_check	True	True	True	True
predict_phase	True	True	True	True
lstm_layers	3	3	3	3
cnn_layers	4	4	4	4
units	128	128	128	128
filter_depth	96	96	96	96
check_every_nth_batch	1000	1000	5000	5000
patience	9	9	7	7
save_every_epoch	N/A	N/A	N/A	N/A

parameter	land_plant_v0.3_a_0300	land_plant_v0.3_a_0400	land_plant_v0.3_m_0100	land_plant_v0.3_m_0200
data_dir	seed_002_016	seed_002_017	fullmoon_211117_17	ori9
data_helixer_commit	02e5e94	02e5e94	bb840b4	bb840b4
data_geenuff_commit	46418ef	46418ef	1f6cffb	1f6cffb
training_helixer_commit	02e5e94	02e5e94	bb840b4	bb840b4
kernel_size	10	10	10	10
learning_rate	N/A	N/A	N/A	N/A
pool_size	9	9	9	9
batch_size	50	50	50	50
val_test_batch_size	100	100	100	100
class_weights	[0.7,1.6,1.2,1.2]	[0.7,1.6,1.2,1.2]	[0.7,1.6,1.2,1.2]	[0.7,1.6,1.2,1.2]
transition_weights	[1,12,3,1,12,3]	[1,12,3,1,12,3]	[1,12,3,1,12,3]	[1,12,3,1,12,3]
save_every_check	True	True	N/A	N/A
predict_phase	True	True	True	True
lstm_layers	3	3	3	3
cnn_layers	4	4	4	4
units	128	128	128	128
filter_depth	96	96	96	96
check_every_nth_batch	5000	5000	N/A	N/A
patience	7	7	N/A	N/A
save_every_epoch	N/A	N/A	True	True

Table S30: Details of parameters utilised in the development of various Helixer vertebrate models that have been released. For performance metrics of each model, please refer to Figures S1 and S5.

parameter	vertebrate_v0.3_a_0200	vertebrate_v0.3_a_0300	vertebrate_v0.3_a_0400
data_dir	seed_020_043	seed_020_036	seed_004_043
data_helixer_commit	ac7ba57	ac7ba57	ac7ba57
data_geenuff_commit	46418ef	46418ef	46418ef
training_helixer_commit	ac7ba57	ac7ba57	ac7ba57
pool_size	9	9	9
batch_size	140	140	140
val_test_batch_size	280	280	280
class_weights	[0.7,1.6,1.2,1.2]	[0.7,1.6,1.2,1.2]	[0.7,1.6,1.2,1.2]
transition_weights	[1,12,3,1,12,3]	[1,12,3,1,12,3]	[1,12,3,1,12,3]
save_every_check	True	True	True
predict_phase	True	True	True
lstm_layers	3	3	3
cnn_layers	4	4	4
units	128	128	128
filter_depth	96	96	96
kernel_size	9	9	9
check_every_nth_batch	1000	1000	1000
patience	6	6	6
manual_checkpoint_selection	N/A	N/A	N/A

parameter	vertebrate_v0.3_m0100	vertebrate_v0.3_m_0080	vertebrate_v0.3_m_0090
data_dir	ori6	seed_020_043_ori0_up0_xtr1	seed_020_043_ori0_up0_xtr1
data_helixer_commit	02e5e94	ac7ba57	ac7ba57
data_geenuff_commit	46418ef	46418ef	46418ef
training_helixer_commit	02e5e94	82bf1ca	82bf1ca
pool_size	9	9	9
batch_size	240	120	120
val_test_batch_size	480	240	240
class_weights	[0.7,1.6,1.2,1.2]	[0.7,1.6,1.2,1.2]	[0.7,1.6,1.2,1.2]
transition_weights	[1,12,3,1,12,3]	[1,12,3,1,12,3]	[1,12,3,1,12,3]
save_every_check	True	True	True
predict_phase	True	True	True
lstm_layers	3	4	4
cnn_layers	4	4	4
units	128	128	128
filter_depth	96	128	128
kernel_size	10	10	10
check_every_nth_batch	1000	1300	1300
patience	7	9	9
manual_checkpoint_selection	N/A	model_e2_b003899.h5 -> model_e0_b002599.h5	model_e2_b003899.h5 -> model_e0_b003899.h5

Table S31: Details of parameters utilised in the development of various Helixer invertebrate models that have been released. For performance metrics of each model, please refer to Figures S2 and S6.

parameter	invertebrate_v0.3_a_0300	invertebrate_v0.3_a_0400	invertebrate_v0.3_a_0500
data_dir	seed_001_045	seed_001_039	seed_015_045
data_helixer_commit	ac7ba57	ac7ba57	ac7ba57
data_geenuff_commit	46418ef	46418ef	46418ef
training_helixer_commit	ac7ba57	ac7ba57	ac7ba57
pool_size	9	9	9
batch_size	140	140	140
val_test_batch_size	280	280	280
class_weights	[0.7,1.6,1.2,1.2]	[0.7,1.6,1.2,1.2]	[0.7,1.6,1.2,1.2]
transition_weights	[1,12,3,1,12,3]	[1,12,3,1,12,3]	[1,12,3,1,12,3]
save_every_check	True	True	True
predict_phase	True	True	True
lstm_layers	3	3	3
cnn_layers	4	4	4
units	128	128	128
filter_depth	96	96	96
kernel_size	9	9	9
check_every_nth_batch	1000	1000	1000
patience	6	6	6
manual_checkpoint_selection	N/A	N/A	N/A

parameter	invertebrate_v0.3_a_0600	invertebrate_v0.3_m_0100	invertebrate_v0.3_m_0200
data_dir	seed_015_039	seed_022_048_booster_001_039_ori1_up1_xtr0	seed_022_048_booster_001_039_ori1_up1_xtr0
data_helixer_commit	ac7ba57	ac7ba57	ac7ba57
data_geenuff_commit	46418ef	46418ef	46418ef
training_helixer_commit	ac7ba57	82bf1ca	82bf1ca
pool_size	9	9	9
batch_size	140	120	120
val_test_batch_size	280	240	240
class_weights	[0.7,1.6,1.2,1.2]	[0.7,1.6,1.2,1.2]	[0.7,1.6,1.2,1.2]
transition_weights	[1,12,3,1,12,3]	[1,12,3,1,12,3]	[1,12,3,1,12,3]
save_every_check	True	True	True
predict_phase	True	True	True
lstm_layers	3	4	4
cnn_layers	4	4	4
units	128	128	128
filter_depth	96	128	128
kernel_size	9	10	10
check_every_nth_batch	1000	1300	1300
patience	6	9	9
manual_checkpoint_selection	N/A	model_e3_b006499.h5	model_e3_b014299.h5

Table S32: Details of parameters utilised in the development of the Helixer mammal model that has been released.

parameter	mammal_v0.3_a_0400
data_dir	training
data_helixer_commit	73e6aca
data_geenuff_commit	46418ef
training_helixer_commit	73e6aca
pool_size	9
batch_size	480
val_test_batch_size	960
class_weights	[0.7,1.6,1.2,1.2]
transition_weights	[1,12,3,1,12,3]
save_every_check	True
predict_phase	True
lstm_layers	4
cnn_layers	4
units	128
filter_depth	128
kernel_size	10
check_every_nth_batch	1300
patience	9
manual_checkpoint_selection	N/A

S3.3.2 Additional Information

Table S33: Species used for model evaluation, indicating the AUGUSTUS model used (closest available), and the corresponding Helixer model

Species	AUGUSTUS closest	Helixer lineage
Rhincodon_typus	rhincodon	vertebrate
Schistosoma_mansoni	(schistosoma2)	invertebrate
Apis_dorsata	adorsata	invertebrate
Bombus_impatiens	bombus_impatiens1	invertebrate
Tribolium_castaneum	tribolium2012	invertebrate
Culex_pipiens	culex	invertebrate
Neurospora_crassa	(neurospora)	fungi
Laccaria_bicolor	laccaria_bicolor	fungi
Fusarium_graminearum	(fusarium)	fungi
Debaryomyces_hansenii	debaryomyces_hansenii	fungi
Coccidioides_immitis	coccidioides_immitis	fungi
Aspergillus_terreus	aspergillus_terreus	fungi
Nicotiana_attenuata	coyote_tobacco	land_plant
Triticum_dicoccoides	wheat	land_plant
Solanum_pennellii	tomato	land_plant
Oryza_brachyantha	rice	land_plant

Table S34: Non default and result affecting parameters used for various bioinformatics tools unless otherwise specified.

tool	parameter	value
HelixerPost		
helixer_post_bin	windowSize	100
helixer_post_bin	edgeThresh	0.1
helixer_post_bin	peakThresh	0.8
helixer_post_bin	minCodingLength	60
BUSCO		
busco	mode	prot
busco	lineage	metazoa_odb10 (vertebrates and invertebrates)
busco	lineage	viridiplantae_odb10 (land_plants)
busco	lineage	fungi_odb10 (fungi)

Table S35: Parameters changed in training ablation models as compared to best land_plant model (also shown, v0.3_a_0080)

name	architecture	predict phase	transition weights
land_plant_v0.3_a_0080	HybridModel	True	[1, 12, 3, 1, 12, 3]
transition_weights_medium	HybridModel	True	[1, 8, 2, 1, 8, 2]
transition_weights_low	HybridModel	True	[1, 4, 1.5, 1, 4, 1.5]
transition_weights_none	HybridModel	True	[1, 1, 1, 1, 1, 1]
phase_none	HybridModel	False	[1, 12, 3, 1, 12, 3]
LSTM	LSTMModel	True	[1, 12, 3, 1, 12, 3]

Table S36: The overlapping parameters during inference in the ablation analysis differ slightly from other analyses, but were constant for all models and species.

parameter	value
subsequence length	106920
overlap	TRUE
overlap offset	13365
core length	53460

S4 Supplemental Discussion

While running the evaluation on the test set, it became evident that current best Helixer models have more issues with over-prediction (precision), while Helixer excelled at recall. Moreover, the over-prediction was not random, but concentrated in more fragmented genomes, and particularly relevant here over-prediction was occurring in large part on the small contigs below the subsequence length, i.e. those with padding.

We hypothesize that this is due to an early decision we made on how to handle small contigs without genes, which could either result from no-gene calling having been performed on the small contigs (not uncommon), or of course, no genes being present. At the time, we elected to avoid including likely erroneous data and filtered completely unannotated contigs from the training data, ignoring the bias this created in the remaining small contigs. This decision appears to have become problematic once the training species sets were expanded to include highly fragmented genomes. Thus, we have given the network a shortcut, which–like the cautionary example of a medical model making predictions based on a hospital-specific token instead of the lung image (https://doi.org/10.1371/journal.pmed.1002683)–lets the model achieve higher training accuracy by over predicting genes on contigs when it sees padding, at the expense of relying on biological features.

We report this while it is still under development for three reasons, 1) allow the earlier documentation of models that are, despite this issue, state-of-the-art, 2) inform users of current best models about likely error types, and 3) as a genomics-field example for potential Deep Learning shortcuts that modellers might face.

Both less naïve filters and data augmentation (shift and pad subsequences from well-assembled genomic regions) are being considered and tested to address this problem and the next generation of models should be available soon.

References

Stephen F Altschul, Thomas L Madden, Alejandro A Schäffer, Jinghui Zhang, Zheng Zhang, Webb Miller, and David J Lipman. Gapped blast and psi-blast: a new generation of protein database search programs. *Nucleic acids research*, 25(17):3389–3402, 1997.

Gary Benson. Tandem repeats finder: a program to analyze dna sequences. *Nucleic acids research*, 27(2):573–580, 1999.

Tomáš Brůna, Katharina J Hoff, Alexandre Lomsadze, Mario Stanke, and Mark Borodovsky. Braker2: automatic eukaryotic genome annotation with genemark-ep+ and augustus supported by a protein database. *NAR genomics and bioinformatics*, 3(1):lqaa108, 2021.

Jullien M Flynn, Robert Hubley, Clément Goubert, Jeb Rosen, Andrew G Clark, Cédric Feschotte, and Arian F Smit. Repeatmodeler2 for automated genomic discovery of transposable element families. *Proceedings of the National Academy of Sciences*, 117(17):9451–9457, 2020.

Lars Gabriel, Felix Becker, Katharina J Hoff, and Mario Stanke. Tiberius: end-to-end deep learning with an hmm for gene prediction. *Bioinformatics*, 40(12):btae685, 2024.

Geo Pertea and Mihaela Pertea. Gff utilities: Gffread and gffcompare. *F1000Research*, 9:304, 2020. URL `https://doi.org/10.12688/f1000research.23297.2`.

Aaron R Quinlan and Ira M Hall. Bedtools: a flexible suite of utilities for comparing genomic features. *Bioinformatics*, 26(6):841–842, 2010.

AFA Smit, R Hubley, and P Green. RepeatMasker 4.1.7-p1. `http://www.repeatmasker.org`, 2013–2025. Downloaded on 2025-03-19.

Felix Stiehler, Marvin Steinborn, Stephan Scholz, Daniela Dey, Andreas PM Weber, and Alisandra K Denton. Helixer: cross-species gene annotation of large eukaryotic genomes using deep learning. *Bioinformatics*, 36(22-23):5291–5298, 2020.

Roman L Tatusov, Eugene V Koonin, and David J Lipman. A genomic perspective on protein families. *Science*, 278 (5338):631–637, 1997.

Andrew Viterbi. Error bounds for convolutional codes and an asymptotically optimum decoding algorithm. *IEEE transactions on Information Theory*, 13(2):260–269, 1967.
