# Supplementary material for: Helixer: ab initio prediction of primary eukaryotic gene models combining deep learning and a hidden Markov model
Source: Nat Methods. 2025 Nov 24;23(4):732–9. doi: 10.1038/s41592-025-02939-1 (PMC13076211; doi:10.1038/s41592-025-02939-1)
Supplement: Supplementary file 2 — Reporting Summary [file 41592_2025_2939_MOESM2_ESM.pdf]

nature portfolio

Corresponding author(s): Marie Bolger

Last updated by author(s): Oct 22, 2025

Reporting Summary

Nature Portfolio wishes to improve the reproducibility of the work that we publish. This form provides structure for consistency and transparency in reporting. For further information on Nature Portfolio policies, see our Editorial Policies and the Editorial Policy Checklist.

Statistics

For all statistical analyses, confirm that the following items are present in the figure legend, table legend, main text, or Methods section.

n/a	Confirmed	
☒	☐	The exact sample size (*n*) for each experimental group/condition, given as a discrete number and unit of measurement
☒	☐	A statement on whether measurements were taken from distinct samples or whether the same sample was measured repeatedly
☒	☐	The statistical test(s) used AND whether they are one- or two-sided<br>*Only common tests should be described solely by name; describe more complex techniques in the Methods section.*
☒	☐	A description of all covariates tested
☒	☐	A description of any assumptions or corrections, such as tests of normality and adjustment for multiple comparisons
☒	☐	A full description of the statistical parameters including central tendency (e.g. means) or other basic estimates (e.g. regression coefficient) AND variation (e.g. standard deviation) or associated estimates of uncertainty (e.g. confidence intervals)
☒	☐	For null hypothesis testing, the test statistic (e.g. *F*, *t*, *r*) with confidence intervals, effect sizes, degrees of freedom and *P* value noted<br>*Give P values as exact values whenever suitable.*
☒	☐	For Bayesian analysis, information on the choice of priors and Markov chain Monte Carlo settings
☒	☐	For hierarchical and complex designs, identification of the appropriate level for tests and full reporting of outcomes
☒	☐	Estimates of effect sizes (e.g. Cohen's *d*, Pearson's *r*), indicating how they were calculated

Our web collection on statistics for biologists contains articles on many of the points above.

Software and code

Policy information about availability of computer code

Data collection

No physical experimental data was generated for this study. The genome assemblies and annotations were downloaded from public sources. Restricted data (e.g. genomes released before their corresponding publication) were removed.

Data analysis

All code developed for this project is available, either from our public Github repositories or from others in the community.

The most important code versions are listed below for clarity.
Main:

Helixer v0.3.0
HelixerPost v0.3.0

Helixer's code is available on GitHub https://github.com/usadellab/Helixer and the specific release of Version 0.3.0 is also available on Zenodo https://doi.org/10.5281/zenodo.17404831. All software requirements for Helixer can also be accessed via these links. HelixerPost is available on GitHub https://github.com/usadellab/HelixerPost and the specific release of Version 0.3.0 is also available on Zenodo https://doi.org/10.5281/zenodo.17414354.

Software versions:
GeenuFF v0.3.0 (GitHub tag)
HelixerPost v0.3.0
GffCompare v0.12.8
BUSCO v5.2.2

Mercator4 v5.0
Orthofinder v2.5.4
AUGUSTUS v3.3.2 and v3.5.0
GeneMark-ES v4.71_lic
Tiberius v1.1.4

Software for plotting (only major python packages; Python v3.12.3 was used):
h5py v3.13.0
jupyter v1.1.1
matplotlib v3.10.3
matplotlib-venn v1.1.2
notebook v7.4.2
numpy v2.2.6
pandas v2.2.3
seaborn v0.13.2

For RNA-seq processing the following tools were used:
FastQC v0.11.5
Trimmomatic v0.36; extra parameters: ILLUMINACLIP:TruSeq3-PE-2.fa:3:30:10:1:true MAXINFO:36:0.7 MINLEN:36,
Hisat v22.1.0; extra parameters: –max-seeds 8 –dta –pen-canintronlen G,-8,1.5 –pen-noncanintronlen G,-8,1.5
Samtools v1.6
PicardTools v52.0; extra parameters: STRAND=SECOND READ TRANSCRIPTION STRAND
MultiQC v1.8

For manuscripts utilizing custom algorithms or software that are central to the research but not yet described in published literature, software must be made available to editors and reviewers. We strongly encourage code deposition in a community repository (e.g. GitHub). See the Nature Portfolio guidelines for submitting code & software for further information.

Data

Policy information about availability of data

All manuscripts must include a data availability statement. This statement should provide the following information, where applicable:

- Accession codes, unique identifiers, or web links for publicly available datasets
- A description of any restrictions on data availability
- For clinical datasets or third party data, please ensure that the statement adheres to our policy

The pre-trained models are available via GitHub https://github.com/usadellab/Helixer or on Zenodo https://zenodo.org/records/10836346.

Fungus training, validation, and test genomes were acquired from RefSeq on March 4th, 2022; exact accessions can be found in Supplementary Table 17
Plant training and validation genomes were acquired from Phytozome13 on June 7th 2021, test plant genomes were acquired from RefSeq on July 14th 2022; exact accessions can be found in Supplementary Table 18
Vertebrate training, validation, and test genomes were acquired from RefSeq on May 6th, 2022; exact accessions can be found in Supplementary Table 19
Invertebrate training, validation, and test genomes were acquired from RefSeq on May 6th, 2022; exact accessions can be found in Supplementary Table 20
Mammal training, validation and test genomes were acquired from RefSeq on March 13th, 2025; exact accessions can be found in Supplementary Table 21
RNA-seq coverage of Arabidopsis thaliana shown in Extended Data Figure 3 was collected from these SRA accessions:
SRS3032258
ERS1647356
SRS1605924
ERS3438334
SRS2705778
ERS3438336
ERS2617740

Research involving human participants, their data, or biological material

Policy information about studies with human participants or human data. See also policy information about sex, gender (identity/presentation), and sexual orientation and race, ethnicity and racism.

Reporting on sex and gender	NA
Reporting on race, ethnicity, or other socially relevant groupings	NA
Population characteristics	NA
Recruitment	NA
Ethics oversight	NA

Note that full information on the approval of the study protocol must also be provided in the manuscript.

Field-specific reporting

Please select the one below that is the best fit for your research. If you are not sure, read the appropriate sections before making your selection.

☒ Life sciences ☐ Behavioural & social sciences ☐ Ecological, evolutionary & environmental sciences

For a reference copy of the document with all sections, see nature.com/documents/nr-reporting-summary-flat.pdf

Life sciences study design

All studies must disclose on these points even when the disclosure is negative.

Sample size	Sampling was used to a very limited extent in this study. During the training process, for computational reasons, 800 blocks were randomly selected from validation genomes to provide estimates of accuracy metrics to select candidate models. These estimates are included in supplementary figures and tables to illustrate the consistency of prediction metrics across the relevant clades, but since they are based on the genomes used during training, should not considered as definitive metrics in any case.<br><br>Independent test metrics were calculated with the full independent test genomes (and annotations).
Data exclusions	Genome assemblies and associated annotations were quality assessed using automated approaches such as BUSCO. Only the primary (longest) transcript was used from each gene model. During training, gene models which were incomplete (e.g lacking UTRs) or invalid (overlapping another model, invalid splicing, incorrect coding phase, missing start or stop codons etc) were masked.
Replication	Multiple training runs were performed using different random splits of the genomes into training and validation sets. The resulting models were assessed using the separate test data set and showed similar performance.
Randomization	Samples were not assigned to experimental groups in the traditional sense in this study. However, multiple training runs were performed using different random splits of the genomes into training and validation sets.
Blinding	Blinding was not relevant to this study since assessments of the various existing tools and newly trained models used the same deterministic, objective and fully automated process on pre-determined datasets. Random partitioning of genomes into training, validation and test datasets ensured data leakage during the training process thus preventing bias.

Reporting for specific materials, systems and methods

We require information from authors about some types of materials, experimental systems and methods used in many studies. Here, indicate whether each material, system or method listed is relevant to your study. If you are not sure if a list item applies to your research, read the appropriate section before selecting a response.

Materials & experimental systems

n/a	Involved in the study	
☒	☐	Antibodies
☒	☐	Eukaryotic cell lines
☒	☐	Palaeontology and archaeology
☒	☐	Animals and other organisms
☒	☐	Clinical data
☒	☐	Dual use research of concern
☒	☐	Plants

Methods

n/a	Involved in the study	
☒	☐	ChIP-seq
☒	☐	Flow cytometry
☒	☐	MRI-based neuroimaging

Plants

Seed stocks	NA
Novel plant genotypes	NA
Authentication	NA
